# Enhancing the Mitochondrial Uptake of Phosphonium Cations by Carboxylic Acid Incorporation

**DOI:** 10.3389/fchem.2020.00783

**Published:** 2020-09-09

**Authors:** Laura Pala, Hans M. Senn, Stuart T. Caldwell, Tracy A. Prime, Stefan Warrington, Thomas P. Bright, Hiran A. Prag, Claire Wilson, Michael P. Murphy, Richard C. Hartley

**Affiliations:** ^1^School of Chemistry, University of Glasgow, Glasgow, United Kingdom; ^2^MRC Mitochondrial Biology Unit, University of Cambridge, Cambridge, United Kingdom; ^3^Department of Medicine, University of Cambridge, Cambridge, United Kingdom

**Keywords:** mitochondria, phosphonium, mitochondria-targeting, membrane permeation, membrane potential, pH gradient, computational chemistry

## Abstract

There is considerable interest in developing drugs and probes targeted to mitochondria in order to understand and treat the many pathologies associated with mitochondrial dysfunction. The large membrane potential, negative inside, across the mitochondrial inner membrane enables delivery of molecules conjugated to lipophilic phosphonium cations to the organelle. Due to their combination of charge and hydrophobicity, quaternary triarylphosphonium cations rapidly cross biological membranes without the requirement for a carrier. Their extent of uptake is determined by the magnitude of the mitochondrial membrane potential, as described by the Nernst equation. To further enhance this uptake here we explored whether incorporation of a carboxylic acid into a quaternary triarylphosphonium cation would enhance its mitochondrial uptake in response to both the membrane potential and the mitochondrial pH gradient (alkaline inside). Accumulation of arylpropionic acid derivatives depended on both the membrane potential and the pH gradient. However, acetic or benzoic derivatives did not accumulate, due to their lowered pK_a_. Surprisingly, despite not being taken up by mitochondria, the phenylacetic or phenylbenzoic derivatives were not retained within mitochondria when generated within the mitochondrial matrix by hydrolysis of their cognate esters. Computational studies, supported by crystallography, showed that these molecules passed through the hydrophobic core of mitochondrial inner membrane as a neutral dimer. This finding extends our understanding of the mechanisms of membrane permeation of lipophilic cations and suggests future strategies to enhance drug and probe delivery to mitochondria.

## Introduction

Mitochondrial dysfunction contributes to a wide range of pathologies, consequently they are an important therapeutic target (Nunnari and Suomalainen, 2012; Gorman et al., 2016; Murphy and Hartley, 2018). Furthermore, the delivery of probe molecules to the organelle *in vivo* is essential in understanding how mitochondrial dysfunction arises (Yousif et al., 2009; Smith et al., 2012; Logan et al., 2014; Jean et al., 2016). There are a number of strategies to deliver molecules to mitochondria *in vivo*, with conjugation to the alkyltriphenylphosphonium (TPP) cation being the most widespread (Smith et al., 2003, 2012; Yousif et al., 2009). TPP is used because it readily crosses biological membranes without the requirement for a protein carrier, and is chemically tractable making it easy to introduce synthetically into the molecule to be targeted (Smith et al., 2011, 2012), as well as having a good safety and toxicity profile. An important aspect of the TPP targeting system is that its positive charge and the large mitochondrial membrane potential lead to these molecules accumulating ~1,000-fold within energized mitochondria, as described by the Nernst equation (Ross et al., 2006; Smith et al., 2011). Even so, improving mitochondria-targeting head groups to enhance mitochondrial accumulation would lead to improved therapies and probes.

**Figure 1:**
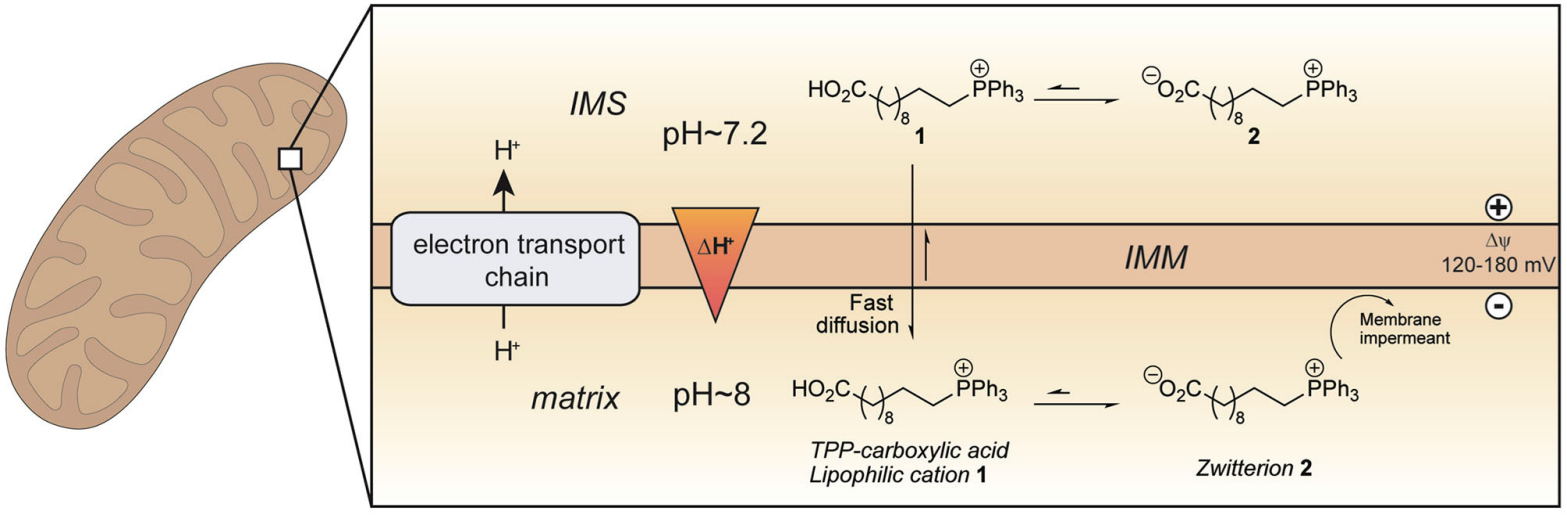
Enhanced mitochondrial uptake by a TPP cation linked to a carboxylic acid. Model showing how a TPP long chain carboxylic acid **1** crosses the mitochondrial inner membrane and accumulates within the mitochondrial matrix in its zwitterionic form **2** due to the higher pH (~ 8) in mitochondria compared to that in the cytosol (~ 7.2). IMM, inner mitochondrial membrane; IMS, intermembrane space.

To develop an improved mitochondria-targeting head group, we started from the finding that a TPP cation conjugated to a long alkyl chain carboxylic acid 1 accumulates within mitochondria to a greater extent than related compounds without an acid moiety (Figure 1) (Finichiu et al., 2013). This greater uptake occurs because of the equilibrium between the protonated **(1)** and deprotonated forms **(2)** determined by the pK_a_ of the carboxylic acid (~4.9) (Kanicky and Shah, 2003). The higher pH of the mitochondrial matrix (~8) relative to the cytosol (~7.2) and the inability of the zwitterionic TPP carboxylate **2** to cross the mitochondrial inner membrane means that the overall accumulation of the molecule is enhanced 4–10-fold compared to a comparable TPP without a carboxylic acid (Finichiu et al., 2013). Hence, we set out to develop a generic TPP-carboxylic acid targeting group so as to enhance mitochondrial uptake 4–10-fold over current TPP groups (Figure 1). The simplest way to do this is to incorporate a carboxylic acid group on one of the TPP phenyls. Both protonated and deprotonated forms of the carboxylic acid would be present in a ratio depending on the pK_a_ of the acid and the local pH (Figure 1). Only the protonated form of the TPP-carboxylic acid targeting group should cross the mitochondrial membrane and once within the matrix, the higher pH should increase accumulation of the membrane-impermeant deprotonated form. Thus, this approach should generate a generic TPP-carboxylic acid targeting group to increase the delivery of the same cargo targeted with a TPP group (Figure 1). Furthermore, this combined TPP-carboxylic acid targeting group could easily be attached to any cargo using a simple phosphine-carboxylic acid precursor. Here we set out to develop such a generic TPP-carboxylic acid targeting group to enhance the delivery of drugs and probes to mitochondria *in vivo*.

## Materials and Methods

### Synthetic Chemistry

**Scheme 1:**
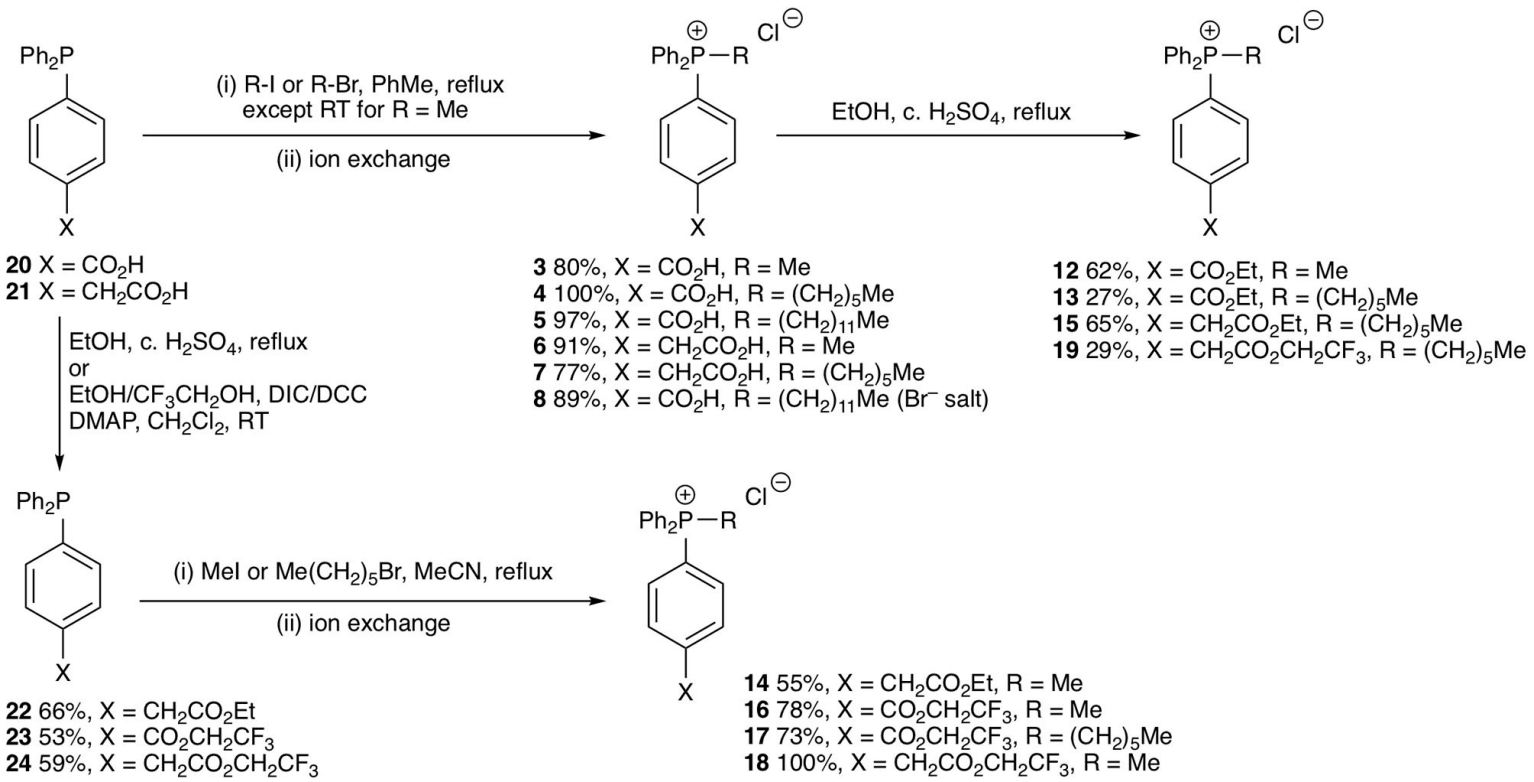
Synthesis of test phosphonium salts **3**–**8** and **12**–**19**.

**Scheme 2:**
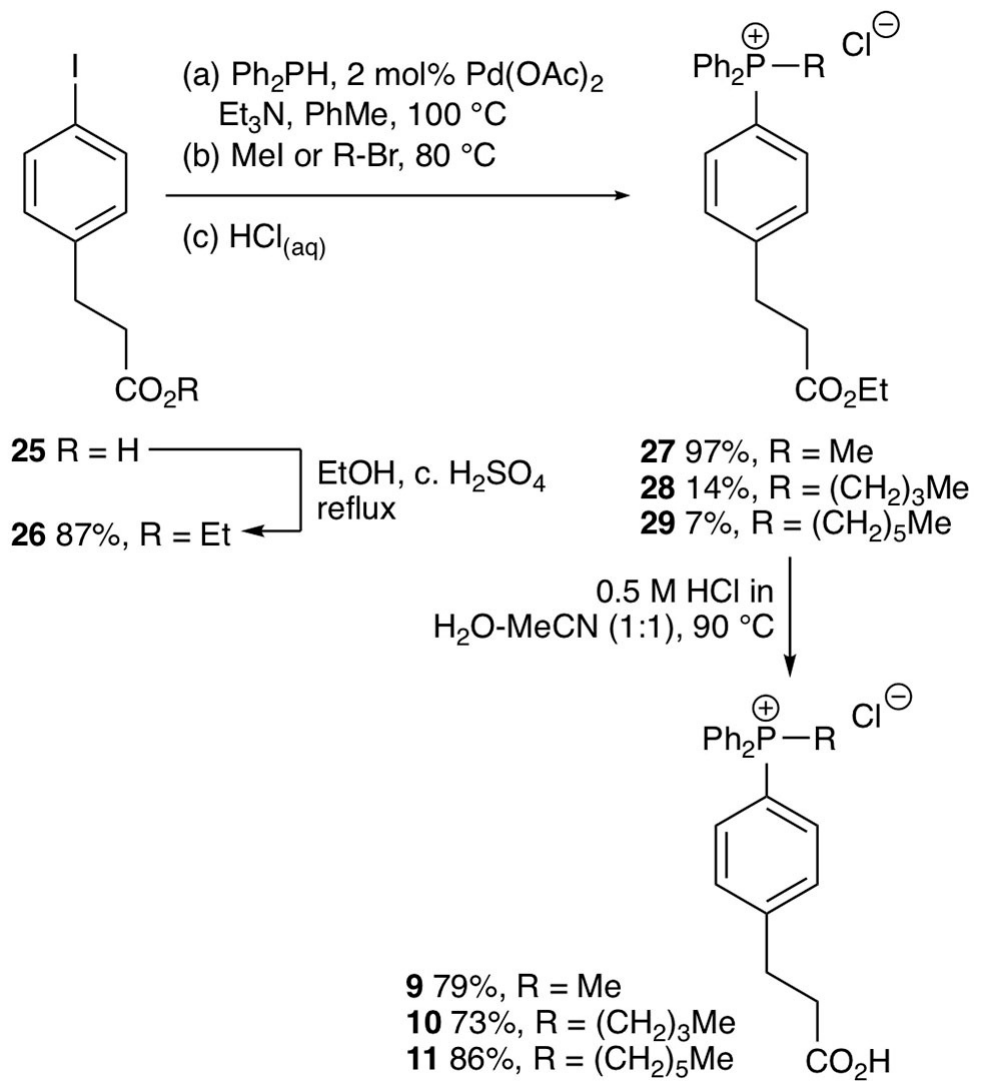
Synthesis of test phosphonium salts **9**–**11**.

The test compounds **3–19** were prepared as shown in Scheme 1. Phosphines **20** and **21** were alkylated with iodomethane, 1-bromohexane, or 1-bromododecane to give the corresponding salts **3–8** (Scheme 1). Carboxylic acids **3**, **4**, **7** were converted into the corresponding ethyl esters **12**, **13**, and **15** by stirring in ethanol with catalytic concentrated sulfuric acid. The trifluoroethyl ester **19** was prepared from carboxylic acid **7** in a similar way using trifluoroethanol as solvent. However, a better approach to trifluoroethyl esters was to esterify the carboxylic acid group of the parent phosphines **20** and **21** with trifluoroethanol using diisopropylcarbodiimide (DIC) coupling with dimethylaminopyridine (DMAP) as a nucleophilic catalyst to give the corresponding esters **23** and **24**, and then alkylate the phosphine to give the phosphonium salts **16–18**. Ethyl ester **14** was prepared in a similar way using dicyclohexylcarbodiimide (DCC) coupling to make ester **22** followed by alkylation with iodomethane. The synthesis of the phenylpropionic acid derivatives **9–11** began with the preparation of iodoarene **25** by the method of (Qin et al., 2015) and protection of the acid as ester **26** (Scheme 2). Adapting the procedure reported by (Dydio et al., 2014), which we had used to prepare phosphine **21**, iodoarene **26** was cross-coupled with diphenylphosphine and then alkylated *in situ* with iodomethane, 1-bromobutane, or 1-bromohexane to give the corresponding phosphonium salts **27–29**. Acid-catalyzed hydrolysis then gave the phosphonium salts **9–11**. The details of the synthetic procedures are described in the Supplemental Information.

### Biology-Methods

#### Animals

All procedures were carried out in accordance with the UK Animals (Scientific Procedures) Act 1986 and the University of Cambridge Animal Welfare Policy. Female Wistar rats (typically 6–10 weeks old, weighing ~200 g, Charles River Laboratories, UK) were housed in pathogen-free facilities at 21 ± 2°C, humidity 57 ± 5% with a 12 h light/dark cycle and laboratory chow and water were available *ad libitum*.

#### Isolation of Rat Liver and Heart Mitochondria

Rats were killed by stunning and cervical dislocation and the liver or heart rapidly excised and transferred to ice cold mitochondrial isolation buffer. Mitochondria were isolated from rat liver tissue by differential centrifugation in STE buffer [250 mM sucrose, 5 mM Tris, 1 mM ethyleneglycol-bis(β-aminoethylether)-*N, N, N', N'*-tetraacetic acid (EGTA) (pH 7.4 at 4°C, adjusted with HCl)] (Chappell and Hansford, 1972). The liver was washed in ice-cold STE, chopped finely, rinsed in STE and homogenized 5–8 times using a loose PTFE pestle in a 55 ml Potter-Elvehjem tissue grinder (Wheaton, USA) and then with a tight pestle. The homogenate was centrifuged (1,000 × g, 3 min, 4°C), the pellets were discarded, and the supernatants centrifuged (10,000 x g, 10 min, 4°C). The supernatant was discarded and the pellet was resuspended and centrifuged again (10,000 × g, 10 min, 4°C). The final mitochondria pellet was resuspended in ~5 mL STE buffer, kept in ice and used within 4 h.

Rat hearts were washed in ice-cold STEB buffer [STE buffer supplemented with 0.1% (ws/v) fatty-acid free bovine serum albumin (BSA)], chopped finely, washed again and then homogenized 5–8 times using a loose PTFE pestle in a 55 mL Potter-Elvehjem tissue grinder (Wheaton, USA) and then a tight pestle with roughly 8 strokes. The homogenate was centrifuged (700 × g, 5 min, 4°C) and the supernatant was decanted through two layers of a pre-wetted muslin into new centrifuge tubes. The pellet was resuspended and centrifuged again (700 × g, 5 min, 4°C). This supernatant was also decanted through muslin and the combined supernatants centrifuged (10,000 × g, 10 min, 4°C). The mitochondrial pellet was resuspended in STEB and centrifuged again (10,000 × g, 10 min, 4°C). The mitochondrial pellet was resuspended in STE without BSA (~300 μL per heart), kept on ice and used within 4 h. The protein concentration of the mitochondrial suspensions was determined using the BCA assay using BSA as a standard (Thermo Fisher Scientific, UK).

#### Reversed Phase-High Performance Liquid Chromatography (RP-HPLC)

For most RP-HPLC experiments, the stationary phase used was a C18 column (Jupiter 300 Å, Phenomenex) with a Widepore C18 guard column (Phenomenex). The column was connected to a 321 pump, a UV/Vis 151 system (Gilson, UK), a RIC20 Remote Controlled Chilling/Heating Dry bath (Torrey Pine Scientific) and a GX-241 liquid handler (Gilson) with Trilution LC 3.0 software (Gilson). The mobile phase was a gradient of water + 0.1% (v/v) trifluoroacetic acid (TFA) (HPLC buffer A) and acetonitrile [HPLC grade acetonitrile (Fisher Scientific, UK)] + 0.1% (v/v) TFA (HPLC buffer B) at a flow rate of 1 mL/min. Samples were prepared in 1 mL, containing 25% acetonitrile + 0.1% (v/v) TFA. Prior to analysis, they were filtered under vacuum using Protein Precipitation Plates (Porvair, 100 μm pre-filter frit, <10 μm secondary frit). The filtrate was transferred to centrifuge tubes and placed in the chilled sample holder. 800 μL of sample were loaded into a 2 mL sample loop and injected onto the column for analysis. Compounds were eluted using the following gradient: 0–2 min: 5% buffer B, 2–17 min: 5-100% buffer B, 17–19 min: 100% buffer B, 19–22 min: 100-5% buffer B. For some RP-HPLC experiments samples were prepared in 850 μL, containing 20% acetonitrile +0.1% (v/v) TFA. This was transferred to centrifuge tubes and placed in the chilled sample holder. 800 μL of sample were loaded into a 2 mL sample loop and injected onto the column for analysis. Compounds were eluted using the following gradient: 0–2 min: 5% buffer B, 2–4 min: 55% buffer B, 4–16 min: 70% buffer B, 16–18 min: 70-100% buffer B, 18–21 min: 100% buffer B, 21–23 min: 100–5% buffer B. All samples were detected at 220 nm and peaks identified by comparing the retention times of the known standards.

#### Mitochondrial Incubation of Compounds

Rat liver or heart mitochondria (0.5 mg protein/mL) were incubated in 2 mL KCl buffer (120 mM KCl, 10 mM HEPES, 1 mM EGTA, adjusted with KOH to pH 7.2, 37°C) supplemented with compound and internal standard (5 μM each, unless otherwise stated) in a shaking heat block (37°C, 1,000 rpm). Mitochondria were energized with succinate/rotenone (10 mM/10 μM) or glutamate/malate (10 mM each). To abolish the mitochondrial proton motive force either FCCP (1 μM), or a mixture of oligomycin, antimycin A and valinomycin (OAV) (5 μg/mL, 100 nM, 1 nM, respectively) were used. After the appropriate incubation time, the mitochondrial suspensions were rapidly cooled on ice and the mitochondria pelleted by centrifugation (10,000 × g, 5 min, 4°C). The mitochondrial pellet was dried by aspirating the supernatant, snap frozen and stored at −80°C until RP-HPLC analysis. For this, the pellet was thawed and extracted by resuspension in 250 μL HPLC buffer B with vortexing, and then centrifuged (16,000 × g, 10 min, 4°C). The resulting supernatant was removed and 750 μL HPLC buffer A was added.

#### Mitochondrial Respiration Assay

Respiration of isolated mitochondria was assessed using the Oroboros Oxygraph-2K (O2K) high resolution respirometer (Oroboros Instruments, Austria), equipped with two stirred (200 rpm), and temperature controlled (37°C) chambers. Before starting experiments, 2 mL KCl buffer was left to equilibrate for approximately 1 h and the Oroboros was calibrated. Rat liver mitochondria (2 mg protein/mL) were then added to the chamber with glutamate and malate (10 mM each). The lid was closed, and respiration assessed. Compounds were added in increasing concentrations using gastight syringes (Hamilton Robotics, UK), with equal volumes of control compound being used in parallel experiments. Additions did not exceed 10 μL per addition.

## Quantum-Chemical Calculations

### Computational Details

Free energies of transfer and of ion-pairing/dimerisation were calculated using density-functional theory (DFT) with the TPSS exchange-correlation functional (Tao et al., 2003) (which is a pure meta-GGA functional) supplemented by Grimme's D3 correction (Grimme et al., 2010, 2011) to account for dispersive interactions. Structures and vibrational frequencies were calculated with the def2-SVP (cations) and def2-SVP+ (ion pairs and anions) basis sets, final energies with def2-TZVP+ in all cases (Weigend and Ahlrichs, 2005; Weigend, 2006). The “+” indicates augmentation of the published sets with one diffuse Gaussian function per valence angular momentum on all atoms; the exponents of the diffuse functions were derived such as to form a geometric series with the existing two outermost functions (even-tempered).

Structure optimisations and frequency calculations were performed with Turbomole 6.4 (Ahlrichs et al., 1989; Häser and Ahlrichs, 1989; Treutler and Ahlrichs, 1995; von Arnim and Ahlrichs, 1998, 1999), making use of the efficient MARI-J technique (Sierka et al., 2003). SCF energies were converged to 10^−8^ *E*_h_; structures to 10^−6^ *E*_h_ in the energy and 10^−4^ *E*_h_ $a_{0}^{-1}$ in the gradient. The COSMO continuum solvent model (Klamt and Schüürmann, 1993; Klamt and Jonas, 1996) was applied throughout, with default solvation radii and relative permittivity ε_r_ = 78.3553 and refractive index *n* = 1.3334 for water and ε_r_ = 1.8819, *n* = 1.3749 for hexane. Frequencies were obtained by numerical differentiation (NumForce), using only the “slow” component of solvent polarization (Klamt, 1996). All structures were confirmed by the absence of imaginary frequencies to be minima on the potential-energy surface in the respective solvent.

Final energies were calculated with Gaussian 09 (Frisch et al., 2013), using the SMD solvation model, which combines the IEF-PCM polarisable continuum model for electrostatic solvation (Tomasi et al., 2005) with the SMD non-electrostatic terms (Marenich et al., 2009). The same values of ε_r_ and *n* were used as above (which are G09 default values, noting that *n* = ε_∞_^1/2^). Default solvation radii were used, except for Cl, whose solvation radius was reduced from 2.38 Å to 1.8 Å in order to reproduce better the experimental free energy of hydration (Hünenberger and Reif, 2011) of Cl^−^, Δ_solv_G^*^(Cl^−^, aq) = −316.55 kJ mol^−1^; the default radius yielded −271 kJ mol^−1^, the adjusted radius −307 kJ mol^−1^. The solvation radius of Na was similarly reduced from 2.27 Å to 1.6 Å; experimental Δ_solv_G^*^(Cl^−^, aq) = −427.41 kJ mol^−1^, calculated using default radius: −302 kJ mol^−1^, calculated using adjusted radius: −429 kJ mol^−1^.

The thermal contributions to the free energy (including the zero-point vibrational energy) were calculated according to the standard ideal-gas/rigid-rotor/harmonic-oscillator approximation at *T* = 298.15 K, *p*° = 100 kPa. Frequencies were not scaled; however, to avoid spurious large entropy contributions from low-frequency vibrational modes, for which the harmonic-oscillator approximation is unreliable, frequencies below 100 cm^−1^ were raised (Ribeiro et al., 2011) to 100 cm^−1^.

p*K*_a_ values were calculated from free energies obtained at the M06-2X/def2-TZVP+ level (Zhao and Truhlar, 2008) using the SMD continuum model for water in Gaussian 09. Frequencies were scaled (Alecu et al., 2010; Kanchanakungwankul et al., 2018) by a factor of 0.972 and raised (Ribeiro et al., 2011) to ≥100 cm^−1^ using the GoodVibes program (Funes-Ardoiz and Paton, 2018) for the calculation of free energies. The p*K*_a_ value of an acid HA was then computed with respect to a reference acid HRef with experimentally known p*K*_a_ as follows:

$$\text{p}K_{\text{a}}\left(\text{HA}\right)=\mathrm{lg}\left(\text{e}\right)\Delta G_{\text{a}}\left(\text{HA}\right)/\left(RT\right)+\text{p}K_{\text{a}}^{\text{expt}}\left(\text{HRef}\right)-\text{p}K_{\text{a}}^{\text{calc}}\left(\text{HRef}\right)$$

where lg(e) is the decadic logarithm of Euler's number, Δ*G*_a_(HA) = G^*^(A^−^) – G^*^(HA), and $\text{p}K_{\text{a}}^{\text{calc}}\left(\text{HRef}\right)=\mathrm{lg}\left(\text{e}\right)\Delta G_{a}\left(\text{HRef}\right)$.

### Choice of Standard States

We express all thermodynamic quantities in solution with reference to a fixed-solute equal-concentrations standard state as defined by Ben-Naim (Ben-Naim, 1987, 1992), indicated by ^*^, with a concentration of c^*^ = 1 mol L^−1^. The usual gas-phase standard state with *p*° = 100 kPa is indicated by °; the standard temperature is *T* = 298.15 K in both cases. The free energy of solute *S* in solution contains a liberational term, which replaces the gas-phase translational free energy. In the ^*^ standard, these terms are exactly equal,

$$\begin{array}{r} G_{\text{lib}}^{*}\left(S,\text{sln}\right)=G_{\text{trans}}^{*}\left(S,\text{g}\right). \end{array}$$

The liberational free energy can therefore be calculated as

$$\begin{array}{r} G_{\text{lib}}^{*}\left(S,\text{sln}\right)=G_{\text{trans}}^{{}^{\circ}}\left(S,\text{g}\right)+\Delta G^{{}^{\circ}\to *}, \end{array}$$

where Δ*G*^° → *^ = *RT* ln(RTc^*^/*p*°) = 8.0 kJ mol^−1^ is the conversion between standard states, associated with the isothermal compression of 1 mol of ideal gas from its concentration at *p*° to c^*^. For a process in solution where the number of particles changes, e.g., ion-pairing, the free-energy change $\Delta G_{\text{sln}}^{\;*}$ therefore includes a term Δ*G*^° → *^ × Σ_*i*_*v*_i_, where ν_*i*_ are the (signed) stoichiometric coefficients of reactants and products.

## Results and Discussion

### Enhanced Mitochondria-Targeting With a Triphenylphosphonium Carboxylic Acid

**Figure 2:**
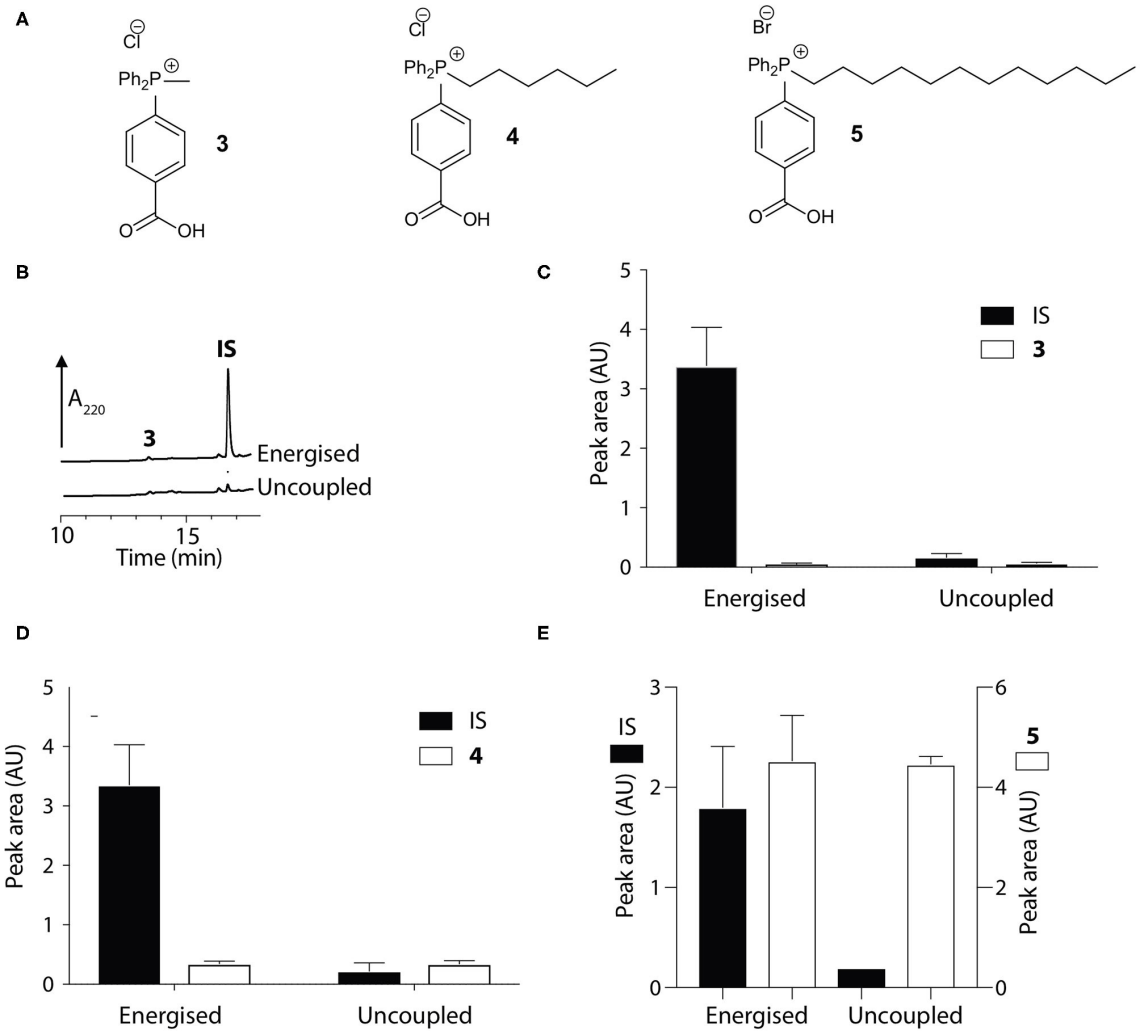
Mitochondrial uptake of TPP benzoic acids. **(A)** Structure of TPP benzoic acids **3**, **4**, and **5**. **(B)** Liver mitochondria (0.5 mg protein/ml) supplemented with succinate and rotenone, were incubated with **3** and an internal standard (IS; isoamyl TPP) at 37°C for 5 min ± FCCP. Mitochondria were pelleted by centrifugation and extracted for analysis by RP-HPLC. **(C–E)** Quantification of mitochondrial uptake of **3**, **4**, and **5**. **(C)**, **3**, IS = isoamyl TPP; **(D)**, **4**, IS = TPMP; **(E)**, **5**, IS = propylTPP). Data are mean ± S.E.M. of peak areas from three biological replicates.

To develop an enhanced mitochondria-targeting head group, we first made a series of alkyltriphenylphosphonium (TPP) salts with a carboxylic acid group on one phenyl group *para* to the phosphorus (Figure 2A). The uptake of these compounds by energized mitochondria, which will have a large membrane potential, was studied alongside TPP-containing internal standards (IS). Uptake of the IS confirmed that the test compound did not significantly disrupt mitochondrial membrane potential and showed that the mitochondria continued to respond as expected to different additives known to affect the membrane potential. Energized mitochondria were incubated with carboxylic acid **3** and IS and uptake of the compounds into mitochondria measured by RP-HPLC (Figure 2B) and quantified (Figure 2C). The IS was taken up by energized mitochondria and abolishing the membrane potential with the uncoupler FCCP (Nicholls and Ferguson, 2013), blocked this uptake (Figures 2B,C). However, carboxylic acid **3** was not taken up by energized mitochondria (Figures 2B,C). A hydrophobicity threshold is required for mitochondrial uptake of alkylTPP molecules (Ross et al., 2006; Finichiu et al., 2015; Hu et al., 2017), but when hydrophobicity was increased by replacing the methyl (**3**) with a hexyl group (**4**) there was still no mitochondrial uptake compared to IS (Figure 2D). Incorporating the more hydrophobic dodecyl group (**5**) led to extensive adsorption to mitochondria that was unaffected by uncoupling with FCCP (Figure 2E). Extending incubation time to 15 min did not lead to uptake (data not shown). We conclude that alkylTPP salts with a carboxylic acid on one of the phenyl groups of TPP are not taken up by energized mitochondria.

**Figure 3:**
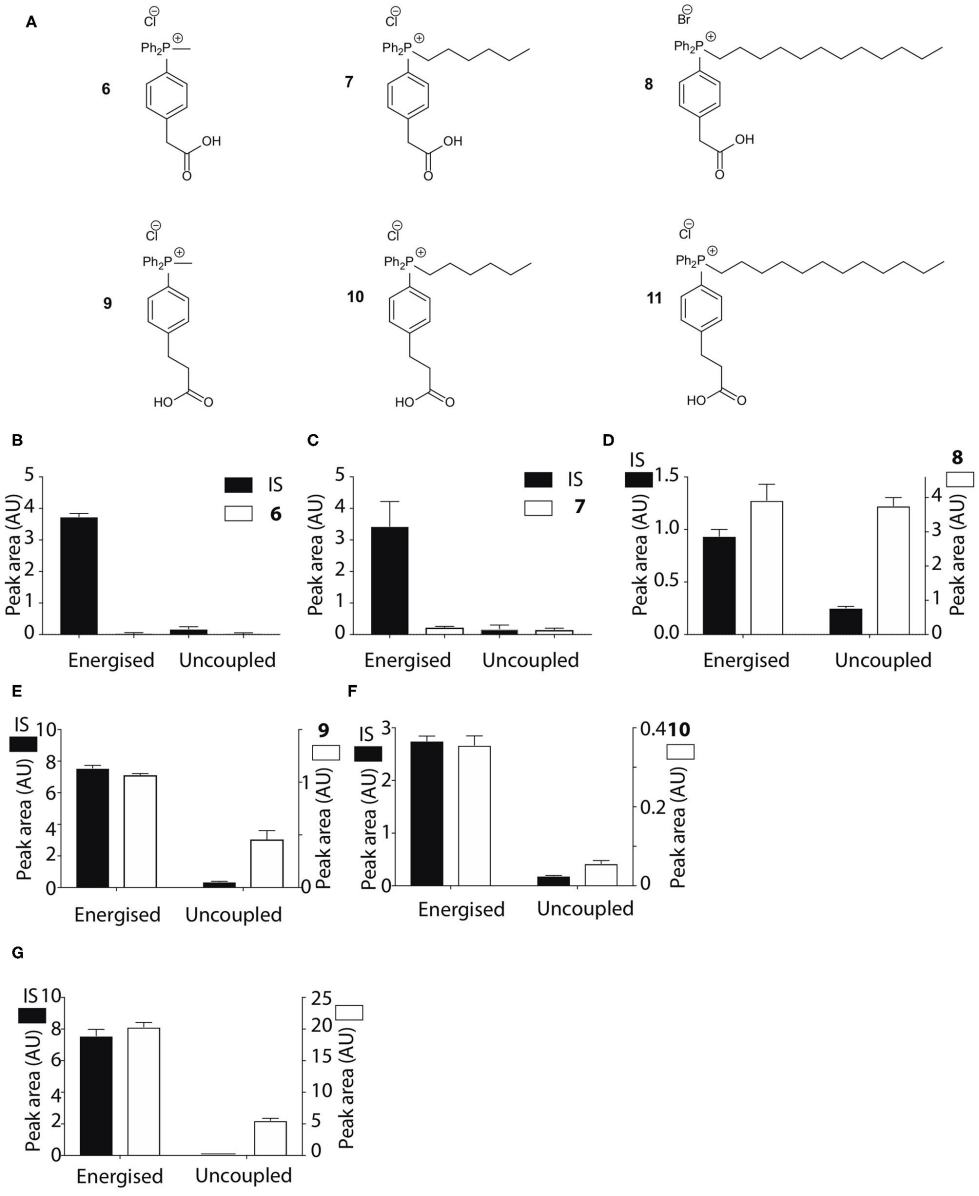
Mitochondrial uptake of TPP acetic and propionic acids. **(A)** Structure of TPP carboxylic acids **6**–**11**. **(B**–**G)** Quantification of mitochondrial uptake of **6**–**11**. Mitochondria were incubated as described in Figure 2B, except for the indicated alterations. **(B)**, **6**, IS = isoamylTPP; **(C)**, **7**, IS = TPMP; **(D)**, **8**, IS = TPMP; **(E)**, **9** (10 μM), IS = propylTPP (2.5 μM), and data shown are for incubation with heart mitochondria. The same result was obtained with liver mitochondria; **(F)**, **10**, (10 μM), IS = TPMP (5 μM), and data shown are for liver mitochondria. The same result was obtained with heart mitochondria; **(G)**, **11**, (20 μM) IS = TPMP (2.5 μM). Data shown are for incubation with heart mitochondria. The same result was obtained with liver mitochondria. Data are mean ± S.E.M. of RP-HPLC peak areas from three biological replicates except for panel G, where the +FCCP data are mean ± range (*n* = 2).

The pK_a_ values of **3**–**5** may have influenced mitochondrial uptake. The enhanced mitochondrial uptake reported previously was for a carboxylic acid attached to a TPP via an undecyl chain (**1**) with a pK_a_~4.9 (Finichiu et al., 2013). Stabilization of the deprotonated form by the phenyl and the phosphonium cation in **3**–**5** may lower the pK_a_, decreasing the proportion in the membrane permeant, protonated form (Figure 1). The pK_a_ of **3** was determined computationally by reference to experimental pK_a_ values for benzoic acid and phenylacetic acid, giving a pK_a_ of 2.5 ± 0.1 (Table 1). At intracellular pH (7.2) the TPP undecylcarboxylic acid (pK_a_ ~4.9) (Kanicky and Shah, 2003) would be ~0.5% in the protonated form, compared to 0.002% for **3**, ~300-fold lower. We next distanced the acid group from the phenyl and phosphonium, to make phenylacetic and phenylpropionic derivatives with a range of hydrophobicities (**6**–**11**) (Figure 3A). The pK_a_ values of **6** and **9** were then computationally determined by reference to the experimental values for benzoic and phenylacetic acid, yielding pK_a_ values of 2.7 ± 0.1 (**6**) and 5.2 ± 0.1 (**9**) (Table 1). So, if **3** was excluded from mitochondria by its low pK_a_ then **6**–**8** should also be excluded, while **9**–**11** should be accumulated. The phenylacetic acid derivatives **6** and **7** showed no uncoupler-sensitive mitochondrial uptake (Figures 3B,C), while the more hydrophobic **8** adsorbed to mitochondria independently of membrane potential (Figure 3D). For the propionic acid derivatives (**9**–**11**) we made methyl, butyl and hexyl versions as the hydrophobicity of the dodecyl compounds **5** and **8** made mitochondrial experiments uninformative. We found that **9**–**11** were all taken up by energized mitochondria, but not by uncoupled mitochondria (Figures 3E–G). Thus, the lack of mitochondrial uptake of the benzoic and phenylacetic derivatives was due to their low pK_a_. Note that the accumulation of the IS were significantly higher than the carboxylic acids **9**-**11** (hence two Y-axes are used in Figures), even when the least lipophilic TPP-derivative, TPMP, was used as IS (Figure 3F).

**Table 1:** Calculated p*K*_a_ values of carboxylic acids **3**, **6**, and **9**, with reference to benzoic acid and phenylacetic acid.

**Compound**	**p*K*_a_**		
	**PhCOOH ref**	**PhCH_**2**_COOH ref**	**Experiment (Rumble et al., 2018)**
PhCOOH	*4.204*	4.4	4.204
PhCH_2_COOH	4.1	*4.31*	4.31
TPMP^+^-COOH (**3**)	2.4	2.6	
TPMP^+^-CH_2_COOH (**6**)	2.6	2.8	
TPMP^+^-CH_2_CH_2_COOH (**9**)	5.1	5.3	

*Reference values are printed in italics*.

**Figure 4:**
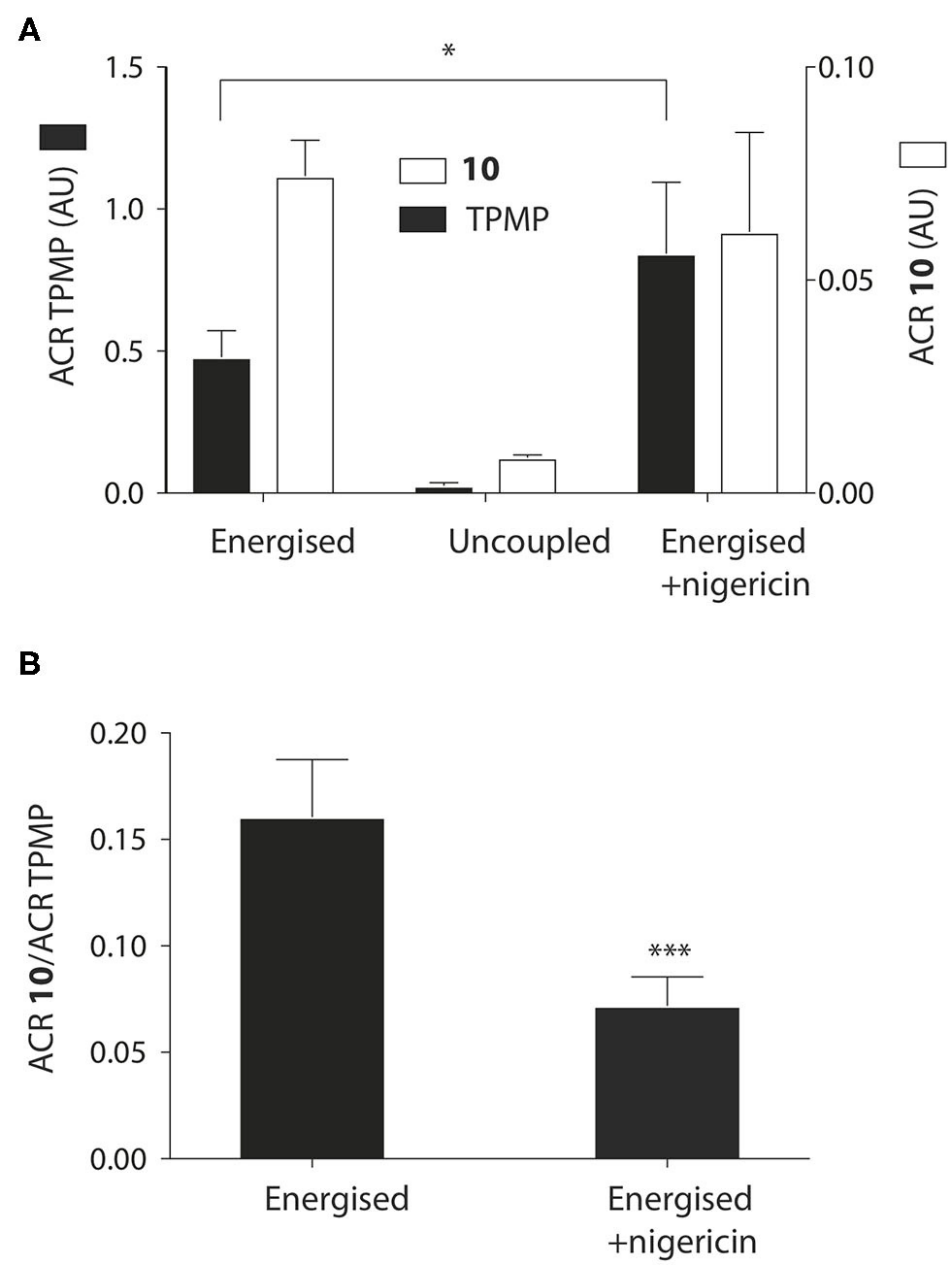
Effect of the pH gradient on mitochondrial uptake of TPP propionic acids. **(A)** Liver mitochondria (1 mg protein in 1 ml) were incubated as described in Figure 2B, with 5 μM **10** and TPMP, with FCCP or nigericin as indicated. Then the mitochondria were pelleted and the amounts of **10** and TPMP in the supernatant and pellets determined by RP-HPLC and used to calculate the ACRs for **10** and TPMP. **(B)** The ratios of the ACRs for **10** and TPMP were determined in the absence and presence of nigericin. Data are mean ± S.E.M. based on RP-HPLC peak areas from 4 to 5 replicates. *p < 0.05, ***p < 0.001 by Student's unpaired *t*-test.

We next assessed whether the mitochondrial uptake of the propionic acid TPP derivatives was enhanced by the pH gradient as shown in Figure 1. To do this we incubated **10** and TPMP with energized mitochondria and measured the accumulation ratio (ACR), that is the amount of the compound accumulated within mitochondria, normalized to the amount in the medium outside the mitochondria (Finichiu et al., 2013). The ACRs for **10** and TPMP were both substantial and were decreased by uncoupling with FCCP (Figure 4A). When the K^+^/H^+^ exchanger nigericin (Nicholls and Ferguson, 2013) was added to abolish the pH gradient and thereby increase the membrane potential, the ACR for TPMP increased as expected, but that for **10** stayed about the same (Figure 4A). This behavior is consistent with the uptake of **10** being in response to both the membrane potential and the pH gradient, while that of TPMP is solely determined by the membrane potential (Finichiu et al., 2013). Hence, the addition of nigericin will increase the uptake of TPMP due to the elevation of the membrane potential, but for **10** the abolition of the pH gradient will counteract any increased uptake due to an elevated membrane potential. To confirm this, we next compared the ratio of the ACRs for **10** and TPMP in the presence and absence of nigericin, which showed that nigericin lowered the ratio of these ACRs (Figure 4B). We conclude that the uptake of **10** into mitochondria is driven by both the membrane potential and the pH gradient as anticipated in Figure 1.

### Utilizing TPP Carboxylic Ester Head Groups to Retain Compounds Within Mitochondria

**Figure 5:**
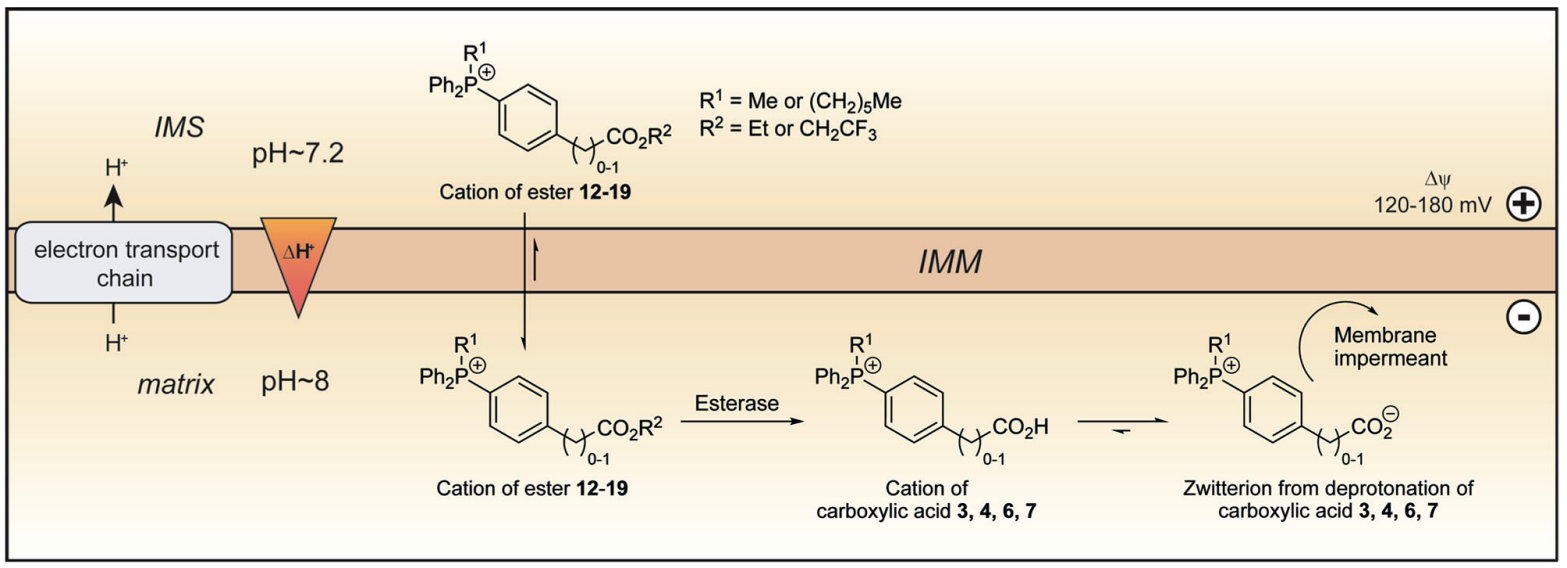
Model of ester uptake and hydrolysis within mitochondria. The lipophilic cations of the esters **12**–**19** readily cross the mitochondrial inner membrane and are accumulated in the matrix in response to the membrane potential. Within mitochondria, hydrolysis forms the cations of the corresponding carboxylic acids, which exist almost exclusively as their highly charged zwitterions at pH 8.0. The zwitterions should be unable to cross the mitochondrial inner membrane and so be trapped within the mitochondrial matrix. IMM, inner mitochondrial membrane; IMS, intermembrane space.

**Figure 6:**
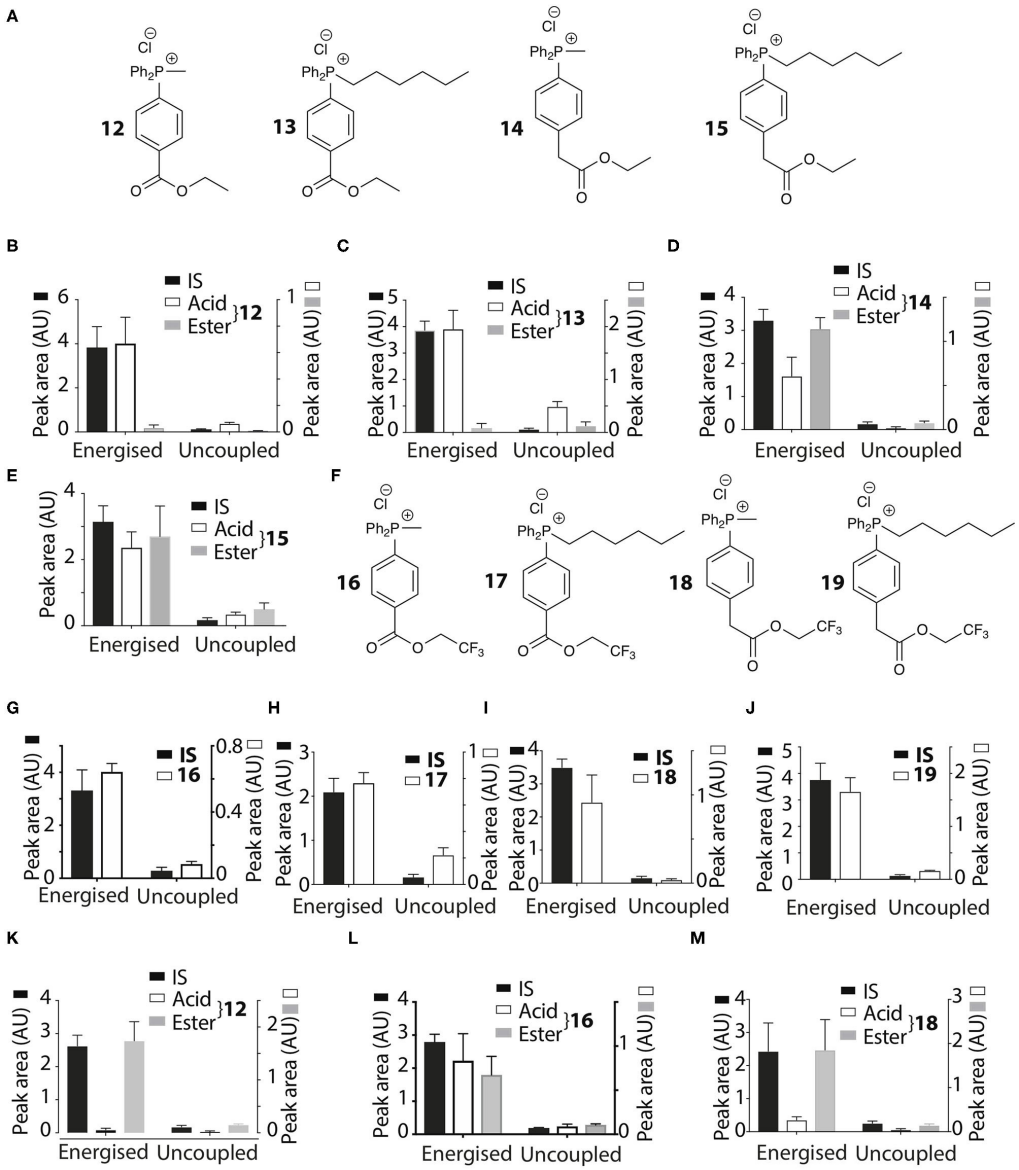
Mitochondrial uptake and hydrolysis of esters. **(A)** Structures of ethyl esters **12**–**15** of TPP carboxylic acids. **(B–E)** Quantification of mitochondrial uptake of **12–15**. Liver mitochondria were incubated as described in Figure 2B. **(B)**, **12**, IS =isoamylTPP; **(C)**, **13**, IS = TPMP; **(D)**, **14**, IS = isoamylTPP; **(E)**, **15**, IS =TPMP. **(F)** Structures of trifluoroethyl esters **16**–**19** of TPP carboxylic acids. **(G–J)** Liver mitochondria were incubated and analyzed with esters as described in Figure 2B. **(G)**, **16**, IS =isoamylTPP; **(H)**, **17**, IS = TPMP; **(I)**, **18**, IS = TPMP; **(J)**, **19**, IS =TPMP. **(K–M)** Heart mitochondria incubated as described in Figure 2B with **12**, **16** or **18** ± FCCP. **(K)**, **12**, IS = isoamylTPP; **(L)**, **16**, IS = isoamylTPP; **(M)**, **18**, IS = isoamylTPP. Data are mean ± S.E.M. of RP-HPLC peak areas from three biological replicates.

The TPP compounds containing benzoic and phenylacetic acid derivatives were not taken up by energized mitochondria because their low pK_a_ prevented passage across the mitochondrial inner membrane. However, their esters should be taken up with subsequent enzymatic hydrolysis within mitochondria then generating the membrane-impermeant acids (Figure 5). This would enable the prolonged delivery of drugs and probes to the mitochondrial matrix where enzymatic hydrolysis generates a TPP carboxylic acid “locked” within the mitochondrial matrix (Figure 5). To test this possibility we made ethyl esters (**12**–**15**) of the membrane impermeant TPP benzoic and phenylacetic acids while we also modulated the lipophilicity with methyl and hexyl alkyl groups (Figure 6A).

The esters **12**–**15** were all taken up by energized liver mitochondria and partially hydrolysed within mitochondria (Figures 6B–E), with the benzoate esters **12** and **13** hydrolysing faster than the phenylacetate esters **14** and **15**. To further accelerate ester hydrolysis we next made esters (**16–19**) incorporating the electron-withdrawing trifluoroethyl group (Figure 6F). Trifluoroethyl esters of phenylacetic acid are known to be hydrolysed 40 times more quickly than ethyl esters by the archetypical esterase, pig liver esterase (Barton et al., 1994). All these esters **16**–**19** were taken up into energized mitochondria and almost completely hydrolysed (Figures 6G–J). The methyl derivatives **12**, **16**, and **18** were also taken up by energized heart mitochondria where the lower esterase activity enabled both acid and ester to be detected (Figures 6K–M).

**Figure 7:**
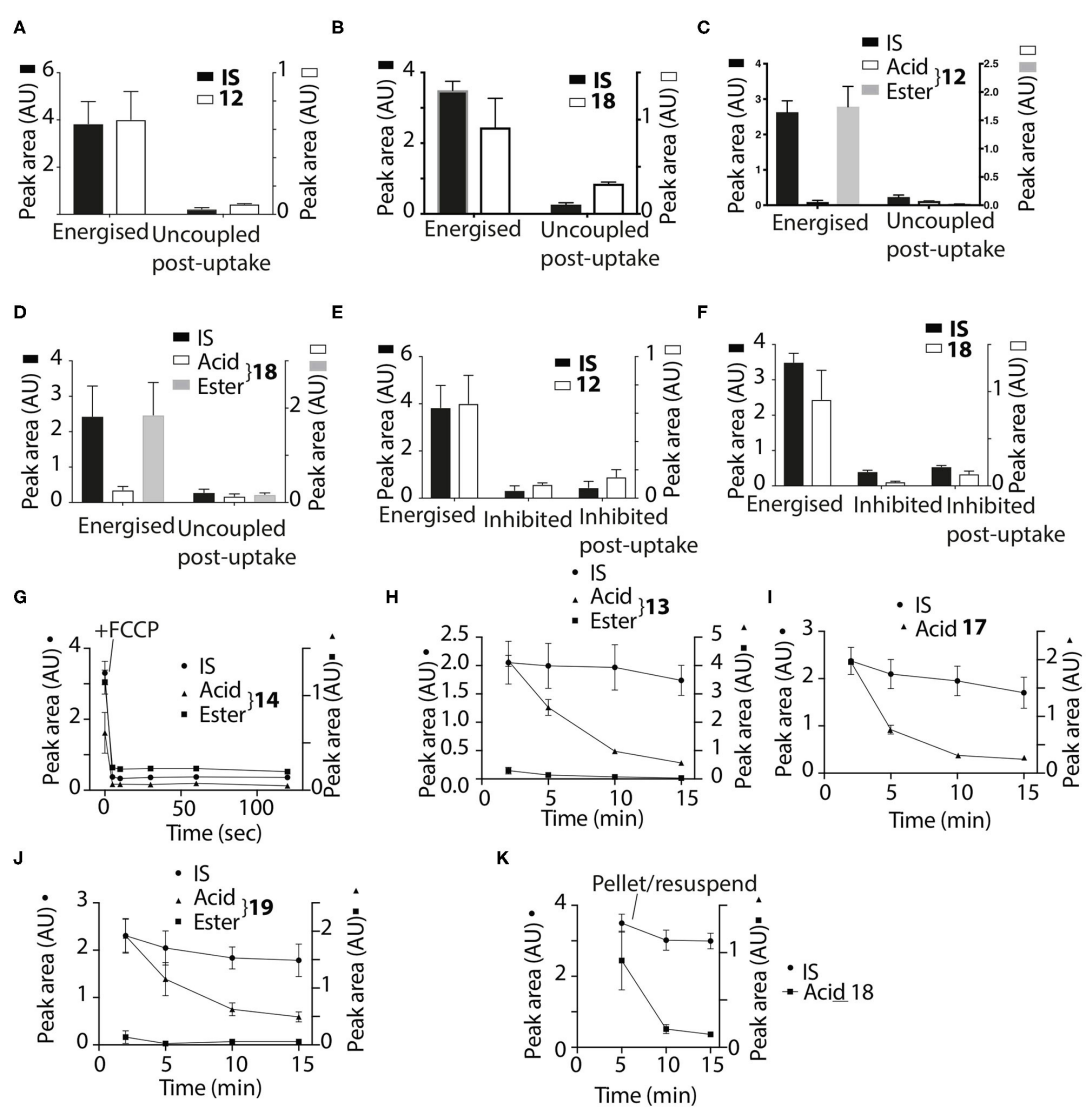
Assessment of retention of acids within mitochondria. Liver **(A,B,E,F)** or heart **(C,D)** mitochondria were incubated with **12** or **18** as described in Figure 2B for 5 min and then either incubated for a further 5 min and then pelleted, or FCCP **(B,D)** was added and after 5 min further incubation the mitochondria were then pelleted. **(A)**, **12**, IS = isoamylTPP; **(B)**, **18**, IS = isoamyl TPP; **(C)**, **12**, IS = isoamyl TPP; **(D)**, **18**, IS = isoamyl TPP. **(E,F)** Liver mitochondria were incubated as described in Figure 2B for 5 min with esters **(E)**, **12** IS = isoamylTPP, or [**(F)**, **18** IS = TPMP] for 10 min ± a mixture of oligomycin/antimycin A/valinomycin or were incubated for 5 min and then a mixture of oligomycin/antimycin A/valinomycin was added after 5 min incubation followed by 5 min further incubation. **(G)**, Liver mitochondria were incubated with **14** and isoamyl TPP as an IS for 5 min, then FCCP was added and the uptake into mitochondria analyzed at the indicated times. **(H,I)** Liver mitochondria were incubated as described in Figure 2B with the esters **17**–**19** for various times and the amounts within the mitochondria were analyzed. **(H)**, **13**, IS = TPMP; **(I)**, **17**, IS = TPMP; **(J)**, **19**, IS = TPMP. **(K)** Liver mitochondria were incubated with **18** and IS = isoamyl TPP for 5 min. Mitochondria were then pelleted by centrifugation and resuspended for 5 or 10 min and then pelleted again. Data are presented as mean ± S.E.M. of quantified peak areas from three biological replicates.

Thus, the ethyl and trifluoroethyl esters **12**–**19** are accumulated by energized mitochondria and are there hydrolysed within the matrix to the corresponding acid. To see if the acid was retained in the matrix, we incubated mitochondria with the esters for 5 min to generate the acid within the matrix, and then abolished the membrane potential with FCCP. As expected, the IS and the remaining ester were lost from the uncoupled mitochondria (Figures 7A–D). However, for **12** and **18** the acid accumulated within liver or heart mitochondria was also lost upon uncoupling to similar extents as the IS and ester (Figures 7A–D). Esters **13**–**17** and **19** showed similar behavior (data not shown). FCCP is a lipophilic anion, so to eliminate the possibility that this affected membrane permeability we also used a mixture of mitochondrial inhibitors to abolish the mitochondrial membrane potential. For **12** and **18** (Figures 7E,F) and **14**, **16** (data not shown) there was similar loss of the acid upon abolition of the membrane potential as was found with FCCP. Finally, to assess how quickly the acid left mitochondria upon uncoupling we incubated mitochondria with **14** for 5 min, then added FCCP and then isolated mitochondria at various time points subsequently (Figure 7G). The rate of efflux of the acid was indistinguishable from that of the IS and the ester at the earliest time point accessible in this experiment (Figure 7G). We conclude that these acids rapidly efflux from mitochondria upon loss of the membrane potential.

To see if the acids were retained within mitochondria by a membrane potential we next assessed their retention over time. When mitochondria were incubated with **13**, **17** and **19**, for up to 15 min IS accumulation was constant, but the amount of the acid present within mitochondria decreased over time (Figures 7H,I,J), with similar data for esters **12**, **13**, **16**, and **18** (data not shown). To further confirm that acids are lost from energized mitochondria, we incubated mitochondria with **18** for 5 min, and then pelleted the mitochondria and resuspended in fresh incubation medium. The IS was retained by the resuspended mitochondria while the acid was lost (Figure 7K). We conclude that the acids are rapidly lost from energized mitochondria.

**Figure 8:**
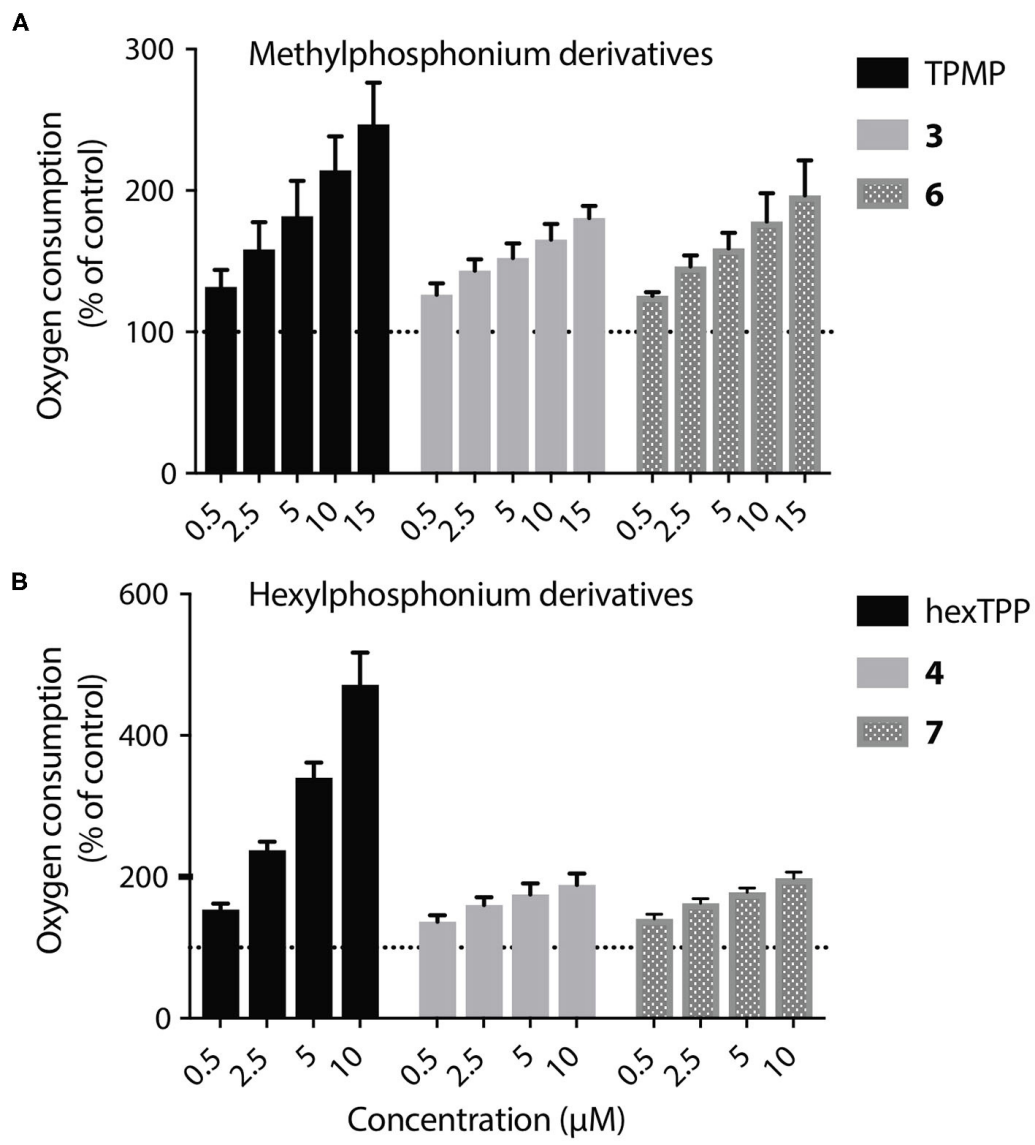
Lack of mitochondrial uncoupling by TPP acids. **(A,B)** Liver mitochondria (0.25 mg mitochondrial protein/mL) respiring on succinate were incubated in KCl buffer (pH 7.2, 37°C) were in an Oroboros O2K high resolution respirometer A range of concentrations of **3**, **4**, **6**, or **7**, or of appropriate ISs, were incubated and the effect on respiration rate, relative to controls without additions were determined. Data are percentage of initial respiration rate before addition of test compounds and are mean ± S.E.M. of three biological replicates.

The acids **3**–**8** are not taken up by energized mitochondria, but when generated within mitochondria they are not retained, whether or not there is a membrane potential. One possibility is that the acid can cross the membrane as both the protonated cation and also as the neutral zwitterion. Thus, membrane potential driven uptake of the acids **3**–**8** in their cationic protonated form, followed by deprotonation and loss of the zwitterion might explain the lack of accumulation. However, this would involve a net influx of protons into the mitochondrial matrix and consequent mitochondrial uncoupling. As increased mitochondrial oxygen consumption is diagnostic of such uncoupling, we measured the effect on this of **3**, **4**, **6**, and **7**, compared to corresponding methyl or hexyl TPPs controls (Figures 8A,B). In all cases the effect of the acid on oxygen consumption was lower than the control compound, which showed the mild uncoupling expected for TPP-compounds (Reily et al., 2013). We conclude that our data cannot be explained by the acids entering as a cation and leaching out as the neutral zwitterion, unless the membrane potential-driven uptake of the cation is very slow and matched by the efflux of the zwitterion (Figure 8A).

### Computational Analysis of Membrane Transport by TPP Derivatives

Low-p*K*_*a*_ TPP-carboxylic acids are not accumulated by energized mitochondria. It is probable that the protonated cation can cross membranes in response to the membrane potential, but that the low concentration of this species renders the flux negligible compared to efflux pathway(s). Once generated within the mitochondrial matrix, the acids are rapidly lost. As this efflux is unaffected by the membrane potential, the acid must cross the membrane as a neutral species. To explore how this might occur we next carried out computational studies to evaluate the Gibbs free energy of membrane crossing of simple salts of TPMP and some of the acids.

The thermodynamics of transfer of ions through, or their partitioning into, a phospholipid membrane can be approximated by considering the transfer of ions from aqueous solution to an apolar medium that mimics the hydrophobic membrane core; here, we chose hexane as the apolar medium. We have used this model previously in calculations to explain differences in the behavior of cations crossing membranes and accumulating in response to a membrane potential (Robb et al., 2015). It has since received some experimental validation, when (Rokitskaya et al., 2019) showed that calculations on partitioning into an alkane solvent correlate exceptionally well with experiments on transmembrane movement of lipophilic ions across black lipid membranes. The ion can transfer from water to hexane as a free ion, or together with a counter-ion such as Cl^−^ as a charge-neutral ion pair. For the transfer of a single monovalent cation from water to hexane, we write

(1) $$\begin{array}{r} {\text{M}}^{+}\left(\text{aq}\right)+{\text{X}}^{-}\left(\text{aq}\right)\to {\text{M}}^{+}\left(\text{Hex}\right)+{\text{X}}^{-}\left(\text{aq}\right) \end{array}$$

The biologically relevant counter-anions X^−^ are typically small and strongly hydrophilic, so their partitioning into the apolar phase is negligible and is only shown here to facilitate comparison with the transfer of an ion pair, as is discussed below. The cation is sufficiently lipophilic to transfer to the apolar phase. The change in free energy associated with Equation (1) is therefore the single-ion free energy of transfer, Δ_t_G^*^(M^+^, aq → Hex). The transfer free energy of a solute *S* is defined as the difference in solvation energy of *S* between the two solvents:

$$\begin{array}{r} \Delta_{\text{t}}G^{*}\left(S,\text{aq}\to \text{Hex}\right)=\Delta_{\text{solv}}G^{*}\left(S,\text{Hex}\right)-\Delta_{\text{solv}}G^{*}\left(S,\text{aq}\right) \end{array}$$

If we start from the same initial state of free solvated ions, the transfer of a neutral ion pair (IP) contains two components: firstly, the formation of the IP in water and secondly, the transfer of the IP to the apolar phase.

(2) $$\begin{array}{r} {\text{M}}^{+}\left(\text{aq}\right)+{\text{X}}^{-}\left(\text{aq}\right)\to \left[{\text{M}}^{+}\cdot {\text{X}}^{-}\right]\left(\text{aq}\right)\to \left[{\text{M}}^{+}\cdot {\text{X}}^{-}\right]\left(\text{Hex}\right) \end{array}$$

Accordingly, the free-energy change associated with Equation (2) is the sum of the free energies of ion-pairing and of transfer of the ion pair.

$$\begin{array}{l} \Delta_{\text{ip}}G^{*}\left(\text{aq}\right)+\Delta_{\text{t}}G^{*}\left(\text{IP},\text{aq}\to \text{Hex}\right) \end{array}$$

Depending on how well solvated the ion is in the respective phase and the stability of the ion pair, it will be more thermodynamically favorable for an ion to cross either as a free ion (Equation 1) or as an ion pair (Equation 2).

Consider TPMP^+^ (Ph_3_PMe^+^) and its ion pair with chloride, [TPMP^+^ · Cl^−^]: Table 2 lists the calculated free energies of transfer for the free ion and the ion pair; for the ion pair, the free energy of pairing is given together with the sum of pairing + transfer of the pair, which is the relevant free energy for crossing as a pair. Unsurprisingly, the free TPMP^+^ cation is better solvated in water than in hexane, making the transfer from water to hexane thermodynamically unfavorable, albeit far less so than for small ions with localized, concentrated charge, such as Na^+^. Larger ions, such as TPMP^+^, have smaller surface charge densities and expose apolar surface patches to the solvent. Hence, they are relatively less well stabilized in water and so the difference between stabilization in the aqueous phase and stabilization by favorable apolar interactions in hexane is less, and the energy cost of transfer from water to hexane is lower (Reichardt and Welton, 2011).

**Table 2:** Calculated free energies of transfer of solute *S* from water to hexane, Δ_t_G^*^(*S*, aq → Hex), and free energies of ion-pairing in water, Δ_ip_G^*^(aq).

**Solute *S***	Δ_t_G*(*S*, aq → Hex)	Δ_ip_G*(aq)	Δ_ip_G*(aq) + Δ_t_G*(*S*, aq → Hex)	**Ion-pair preference**
TPMP^+^	**44**			
TPMP^+^-COOH	**64**			
TPMP^+^-COO^−^	**86**			
TPMP^+^-CH_2_COOH	**59**			
TPMP^+^-CH_2_COO^−^	**88**			
Cl^−^	157			
Na^+^	229			
TPMP^+^ · Cl^−^	39	19	**58**	**14**
TPMP^+^-COOH · Cl^−^	55	18	**73**	**9**
TPMP^+^-COO^−^ · Cl^−^	160	19	**179**	**93**
TPMP^+^-COO^−^ · Na^+^	126	−3	**123**	**40**
TPMP^+^-${\text{COO}}_{2}^{-}$^*a*^	39	16	**55**	**−30**
TPMP^+^-${\text{CH}}_{2}{\text{COO}}_{2}^{-}$ (Conf 1)^*a*^	16	34	**50**	**−39**
TPMP^+^-${\text{CH}}_{2}{\text{COO}}_{2}^{-}$ (Conf 2)^*a*^	30	12	**41**	**−47**

*All free energies are in kJ mol^−1^*.

The preference for crossing as ion pair is defined as Δ_ip_G*(aq) + Δ_t_G*(IP, aq → Hex) – Δ_t_G*(free ion, aq → Hex); a negative value means that crossing as an ion pair is preferred to crossing as a free ion. The term “ion-pairing” is used here to designate both the association with a small counter-ion and the dimerisation of zwitterions.

a *Energies are per mol of monomer*.

*Comparable values for the transfer of free ions vs. ion-pairs are highlighted in bold*.

Despite any free ion being strongly solvated in water, the formation of ion pairs in water is only relatively weakly endergonic. All the ion pairs considered here are still more stable in water than in hexane, but their transfer free energies are smaller than for ions in many cases. Generally speaking, the more hydrophilic/polar the free ion is, the more it benefits from ion-pairing in terms of reducing the energetic disadvantage associated with transferring to hexane. For the example of TPMP^+^, it transfers relatively easily as a free cation; the transfer as an ion-pair [TPMP^+^ · Cl^−^] is disfavoured by another 14 kJ mol^−1^. In summary, “lipophilic” ions (low overall charge, small surface charge, large apolar surface patches) can transfer as free ions from water to hexane at modest thermodynamic cost. They do not benefit from ion-pairing. On the other hand, for hydrophilic ions (high overall charge, concentrated surface charge, little apolar surface area), it is thermodynamically prohibitive to transfer as free ions. However, when paired with a strongly hydrophilic counter ion the cost of transferring is significantly reduced (Robb et al., 2015).

We now consider derivatives of TPMP^+^ where one of the phenyl rings has been substituted in *para* position with a carboxyl or carboxymethyl group. There are therefore multiple possibilities of forming ion pairs with counter-anions or cations (Table 2). Comparing the transfer free energies of the single species, TPMP^+^, TPMP^+^-COOH, and TPMP^+^-COO^−^, it can be seen that introducing the neutral, protonated carboxyl group makes the transfer ~20 kJ mol^−1^ more unfavorable (compared to unsubstituted TPMP^+^) (Table 2). Transferring the zwitterionic carboxylate is significantly disfavored (by ~40 kJ mol^−1^) despite the overall neutral charge because the negative charge of the carboxylate is solvent-exposed and relatively localized. Depending on whether the carboxylic acid is deprotonated or neutral, the following ion pairs were considered: [TPMP^+^-COOH · Cl^−^], [TPMP^+^-COO^−^ · Cl^−^]^−^, [TPMP^+^-COO^−^ · Na^+^]^+^. None of these ion pairs transfers more easily than the corresponding single species (Table 2).

**Figure 9:**
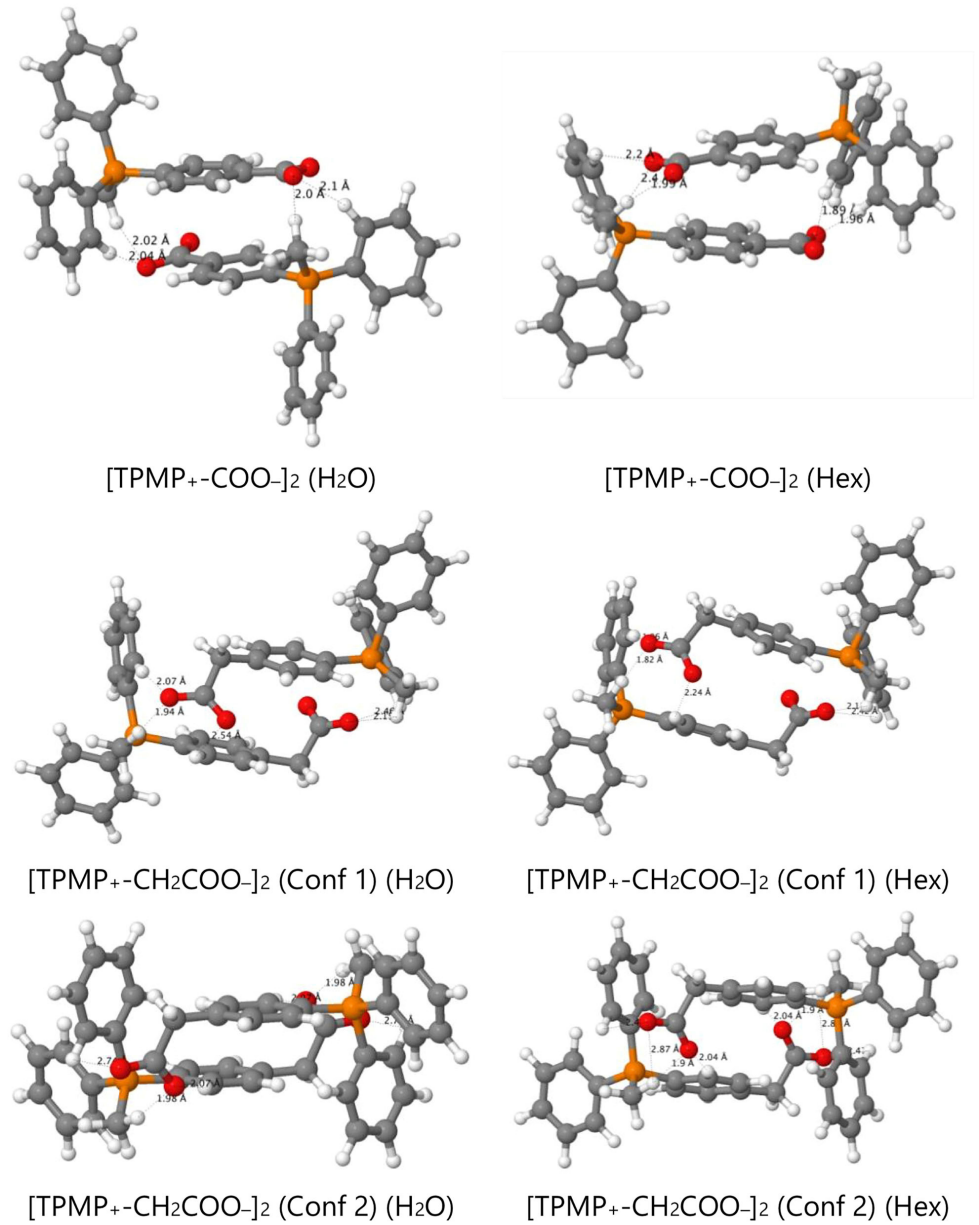
Optimized structures of self-complementary dimers of the assessed zwitterions in water and hexane.

However, the zwitterions TPMP^+^-COO^−^ and TPMP^+^-CH_2_COO^−^ have the ability to form self-complementary (“head-to-tail”) dimers (Figure 9). In these structures, the negatively charged carboxylate groups are partially buried within the pocket formed by the α- and β-hydrogens of the methyl and phenyl substituents of the phosphonium, respectively, which carry a significant fraction of the positive charge (Figure 9). These stabilizing electrostatic interactions make the dimer formation in water similarly favorable to ion-pairing with a small single ion like Cl^−^, that is, endergonic by ~15–20 kJ mol^−1^. At the same time, in the dimers, the negative charges of the carboxylates are locally compensated for and partially shielded from the solvent so that there is less stabilization from interactions with water. Therefore, transfer to hexane is less unfavorable than for the single zwitterions. For the dimer [TPMP^+^-CH_2_COO^−^]_2_, we considered two minimum energy structures. Conformer 2 is more stable than Conformer 1 by 45 kJ mol^−1^ in water, but only by 17 kJ mol^−1^ in hexane. Dimerisation in water is therefore more favorable for Conformer 2, but the transfer energy is less endergonic for Confomer 1 (see Table 2). The two effects largely compensate each other, such that transferring as dimers is clearly preferred for both conformers. Overall, therefore, the dimers transfer significantly more easily than the corresponding single species. The calculated Gibbs energies for all the species taken into consideration (cations, ion pairs, and dimers) are reported in Table 2. We conclude that the most likely way in which the TPP acid derivatives can leave mitochondria is as a neutral dimer of two zwitterionic molecules (Figure 9).

### X-ray Crystallography Support for Dimers

**Figure 10:**
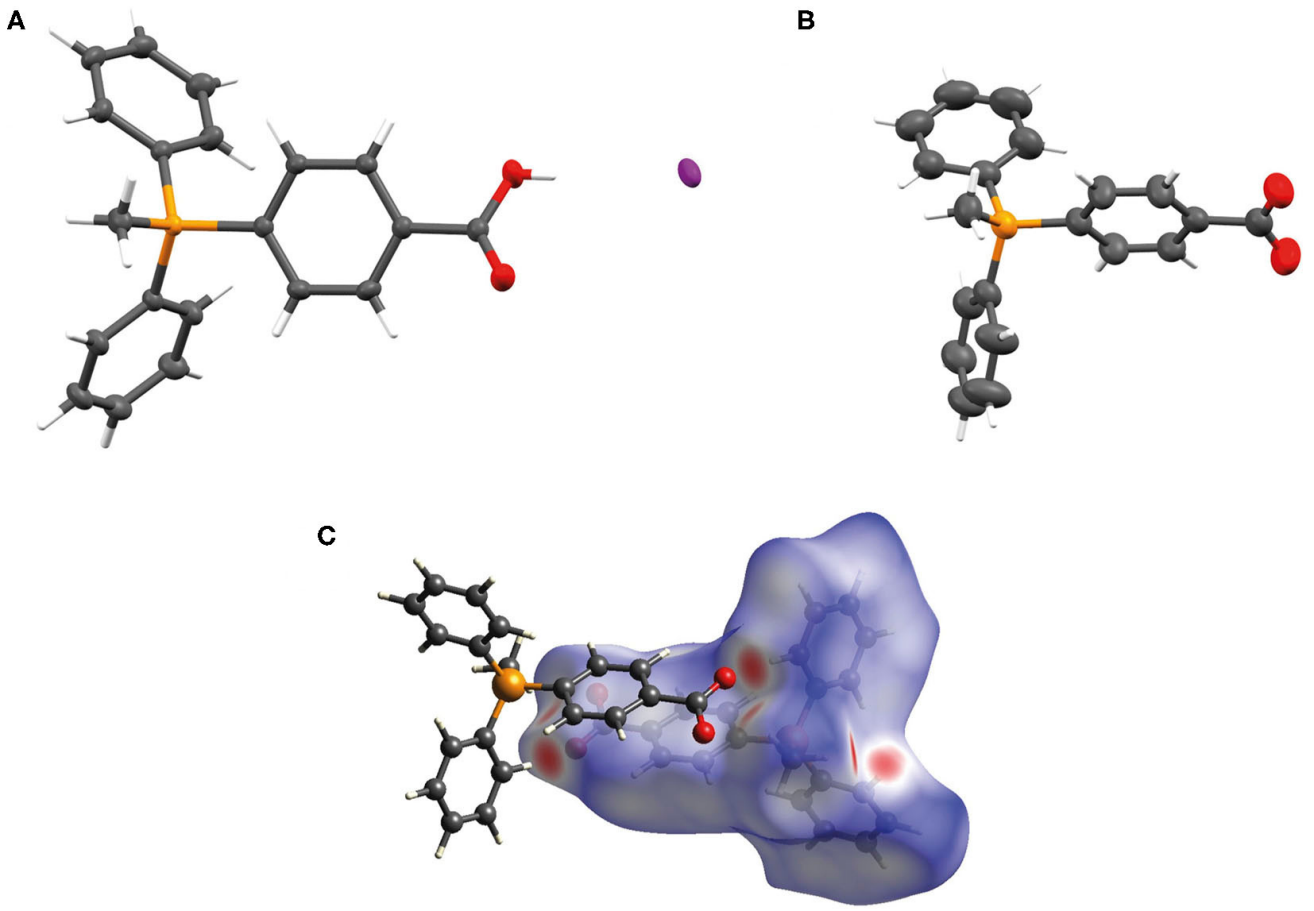
**(A)** The X-ray crystal structure of [TPMP^+^-COOH · I^−^] with atomic displacement ellipsoids drawn at 50% probability level for non-hydrogen atoms. **(B)** The X-ray crystal structure of TPMP^+^-COO^−^ with atomic displacement ellipsoids drawn at 50% probability level for non-hydrogen atoms. **(C)** View of the Hirshfeld surface of TPMP^+^-COO^−^ mapped with d_norm_ property (calculated using CrystalExplorer), contact distance < van der Waals contact are shown in red, equal to it in white and longer as blue. (Spackman and Jayatilaka, 2009).

Finally, we sought crystallographic evidence of the benefit of the interactions identified in our models of the dimer [TPMP^+^-COO^−^]_2_. To do this, we synthesized the pure zwitterionic species without any counterion. First, the iodide salt was prepared by the method used to make the chloride salt **3** of the carboxylic acid (Scheme 1), but without ion exchange. Single crystals of this compound, [TPMP^+^-COOH · I^−^], were grown. Treatment of the iodide salt with silver carbonate removed the iodide and allowed single crystals of the zwitterion, TPMP^+^-COO^−^, also to be grown. Single crystal X-ray diffraction revealed the structures of both [TPMP^+^-COOH · I^−^] and TPMP^+^-COO^−^ (Figures 10A,B). [TPMP^+^-COOH · I^−^] has a C–OH length of 1.325(2) Å and a C=O length of 1.204(2) Å, which are close to the typical C–O (1.308 Å) and C=O (1.214 Å) bond lengths in carboxylic acids (Allen et al., 2006). As expected, there is no halogen present in the structure of TPMP^+^-COO^−^ with the positive charge of the phosphonium ions balanced in the crystal by the negatively charged carboxylate ions. The C–O bond lengths of 1.233(3) and 1.246(3) Å match closely the typical bond length of 1.254 Å for a delocalised C=O carboxylate bond (Allen et al., 2006). Within the crystal lattice multiple individual molecules interact to satisfy the attractive electrostatic interactions in a range of packing motifs. Among these, evidence for the proposed “head-to-tail” dimers was observed (Figure 10C). Here, the carboxylate group of one molecule is in close proximity to the α-hydrogen of the methyl (2.440 Å) and the β-hydrogen of the phenyl (2.237 Å) of its neighbor's phosphonium group. These distances agree well with those predicted in our theoretical models of the dimer [TPMP^+^-CH_2_COO^−^]_2_ (Figure 9).

## Conclusion

**Figure 11:**
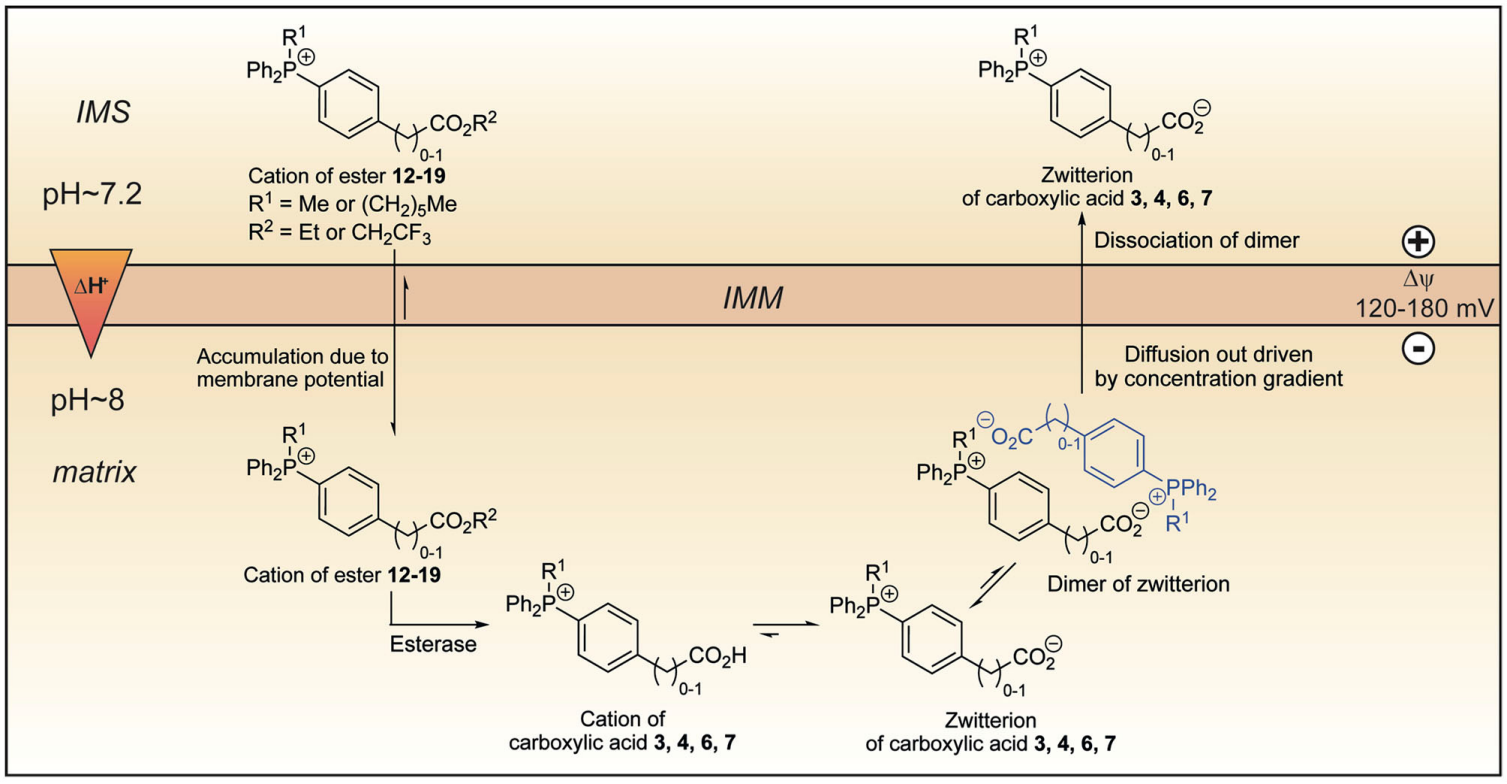
Model explaining the behavior of the esters **12**–**19** within mitochondria. Lipophilic cations of esters **12**–**19** cross the mitochondrial inner membrane and accumulate several hundred-fold due to membrane potential. Hydrolysis forms the cations of carboxylic acids **3**, **4**, **6**, and **7** which exist almost exclusively as their highly charged zwitterions at pH 8.0. Two molecules of the zwitterions dimerise forming an ion couple that diffuses out driven by the concentration gradient created by accumulation of the esters **12**–**19** and then dissociate. IMM, inner mitochondrial membrane; IMS, intermembrane space.

We set out to develop a mitochondria-targeting strategy that would enhance uptake into mitochondria using a targeting head group that would increase mitochondrial uptake of simple TPP cations by responding not only to the membrane potential but also to the pH gradient across the mitochondrial inner membrane. Here we show that incorporation of a propionic acid into a TPP moiety provides derivatives that accumulate in response to both these factors, albeit with a lower ACR than simple TPP compounds. On the other hand, we found that TPP moieties derivatised with benzoic or phenylacetic acid groups on the phosphonium were not taken up by mitochondria due to the low pK_a_ of these acids depleting the amount of cation available to cross the membrane. This suggested that this finding could be exploited to generate esters that would generate such membrane impermeant acids within the mitochondrial matrix thereby locking molecules within the mitochondrial matrix. As the ability to lock molecules within mitochondria might facilitate the development of more persistent mitochondrial probes and therapies, we explored this possibility. To our surprise, we found that despite their low pK_a_ these acids were rapidly lost from mitochondria, irrespective of the presence of a membrane potential. A computational approach suggested that this occurred by the zwitterions forming dimers and that these neutral dimers were then able to pass through the mitochondrial inner membrane. Thus, esters **12**–**19** accumulate in the matrix driven by the membrane potential, but upon hydrolysis, the corresponding carboxylic acids **3**, **4**, **6**, and **7** are not trapped (Figure 11). Instead, they diffuse out of the matrix as dimers of their zwitterions, driven by the concentration gradient. Our work suggests that making TPP carboxylic compounds in which the ability to form dimers was prevented (e.g., by incorporating bulky groups) would improve delivery and open up a new mitochondrial lock in strategy.

In summary, by investigating methods to enhance TPP delivery to mitochondria we have revealed the factors that govern uptake of TPP-carboxylic acids and exposed new potential strategies, which we will explore in future work.

## Data Availability Statement

The NMR data, the Fourier transformed NMR spectra for the synthesised compounds and xyz coordinates and energies of all optimised structures can be found in the University of Glasgow repository http://dx.doi.org/10.5525/gla.researchdata.1004. The CIF files CCDC 1998454 [TPMP^+^-COO^−^] and 1998455 (TPMP^+^-COOH.I^−^] contain the supplementary crystallographic data for this paper. These data can be obtained free of charge from The Cambridge Crystallographic Data Centre via www.ccdc.cam.ac.uk/structure.

## Ethics Statement

All experiments were conducted according to the Animals Scientific Procedures Act 1986 (UK) and directive 2010/63/EU of the European Parliament guidelines on the protection of animals used for scientific purposes. All experiments were approved by the University of Cambridge Institutional Animal Welfare and Ethical Review Body.

## Author Contributions

LP synthesized the majority of the compounds and carried out most biological testing. HS carried out the computational calculations. SC synthesized some of the compounds. SW and CW carried out and interpreted the single X-ray crystallography study. TP and TB carried out biological testing. HP helped design of biological experiments. RH and MM supervised the project and the manuscript writing. All authors contributed to the article and approved the submitted version.

## Conflict of Interest

The authors declare that the research was conducted in the absence of any commercial or financial relationships that could be construed as a potential conflict of interest.
