# Supplementary material for: Enhancing the Mitochondrial Uptake of Phosphonium Cations by Carboxylic Acid Incorporation
Source: Front Chem. 2020 Sep 9;8:783. doi: 10.3389/fchem.2020.00783 (PMC7509049; doi:10.3389/fchem.2020.00783)
Supplement: Supplementary file 1 [file Data_Sheet_1.PDF]

Supplemental Information

Enhancing the Mitochondrial Uptake of Phosphonium Cations by Carboxylic Acid Incorporation

Laura Pala[1], Hans M. Senn[1], Stuart T. Caldwell[1], Tracy A. Prime[2], Stefan Warrington[1], Thomas P. Bright[2], Hiran A. Prag[2], Claire Wilson[1], Michael P. Murphy[2,3]*, Richard C. Hartley[1,] *

[1]School of Chemistry, Joseph Black Building, University of Glasgow, University Avenue, Glasgow, G12 8QQ, United Kingdom

[2]MRC Mitochondrial Biology Unit, Wellcome Trust / MRC Building, University of Cambridge, Cambridge Biomedical Campus, Hills Road, Cambridge, CB2 0XY, United Kingdom

[3]Department of Medicine, University of Cambridge, Cambridge Biomedical Campus, Hills Road, Cambridge, CB2 0QQ, United Kingdom

*** Correspondence:**

Richard C. Hartley
Richard.Hartley@glasgow.ac.uk

Michael P. Murphy
mpm@mrc-mbu.cam.ac.uk

1 SUPPLEMENTAL MATERIALS AND METHODS

1.1 Synthetic Chemistry

1.1.1 General information

All reactions under an inert atmosphere were carried out using flame-dried glassware and solvents were added *via* syringe. Reagents were obtained from commercial suppliers and used without further purification. Anhydrous solvents were collected from a Puresolv solvent purification system or obtained from commercial suppliers. NMR spectra were obtained using a Bruker AVIII 400 spectrometer (^{1}H NMR at 400 MHz, ^{13}C NMR spectra at 101 MHz, ^{31}P NMR spectra at 162 MHz, ^{19}F spectra at 377 MHz) or a Bruker AVIII 500 spectrometer (^{1}H NMR at 500 MHz, ^{13}C NMR spectra at 126 MHz, ^{31}P NMR spectra at 202 MHz, ^{19}F spectra at 471 MHz). Signal splitting patterns were described as: singlet (s), doublet (d), triplet (t), quartet (q), quintet (qn), sextuplet (sx), septuplet (sept), multiplet (m), broad singlet (broad s), or any combination of the above. All coupling constants were recorded in Hz. In ^{1}H NMR spectra tentative assignment was carried out on the basis of the chemical shift, definitive on the basis of the COSY (when made). DEPT was used to assign the signals in ^{13}C NMR spectra as C, CH, CH_2 and CH_3. 2D techniques including COSY and HSQC were used to help assignment. Deuterated solvents contained tetramethylsilane (TMS) as a reference compound. All spectra were assigned using the following reference solvent peaks: $CDCl_3$ (7.26 ppm for ^{1}H NMR; 77.16 ppm for ^{13}C NMR), CD_3CN (1.94 ppm for ^{1}H NMR;

118.26 ppm for ^{13}C NMR), DMSO-*d*$_6$ (2.50 ppm for ^{1}H NMR, 39.52 ppm for ^{13}C NMR). Phosphonium salts entrap solvents, where this is the case the % yield is adjusted based on the presence of the solvent evaluated by ^{1}H NMR. When there was a significant amount of chlorinated solvent present, this was replaced with ethanol before biological testing and some of the definitive spectra contain this solvent. LRMS (ESI^+) and HRMS (ESI^+) spectra were collected on a Bruker MicroTOF-Q, EI spectra were collected on a Jeol JMS700 (MStation) spectrometer. IR spectra were obtained using Shimadzu FTIR-8400S. Reactions were monitored by thin layer chromatography (TLC) performed on aluminium sheets pre-coated with silica gel (Merck or Fluorochem Silica Gel 60 F254) and visualisation was performed using UV light (λ_{max} = 254 or 365 nm) or by staining with a potassium permanganate solution dip. R_f values for phosphonium salts and carboxylic acids are concentration dependent and are reported as the maximum observed R_f. Purification of products was carried out by re-crystallization, distillation under vacuum or Biotage® Isolera™ One Flash Chromatography system using Biotage® SNAP Ultra silica gel cartridges. Single crystal x-ray diffraction data were collected using Bruker D8 Venture equipped with Photon II CPAD detector, dual ImuS 3.0 Cu and Mo sources and n-Helix low temperature device.

1.1.2 Synthetic Procedures

Triphenylmethylphosphonium (TPMP) chloride. Triphenylphosphine (100 mg, 0.381 mmol, 1 eq.) and iodomethane (119 µL, 1.91 mmol, 5 eq.) were dissolved in anhydrous toluene (1 mL) under an atmosphere of argon while stirring. The reaction was conducted at RT while stirring for 16 h. The formed solid was separated by filtration, dissolved in the minimum amount of acetonitrile (2 mL) and triturated with diethyl ether (20 mL). The precipitate was collected *via* filtration and ion exchanged to the chloride form [IRA 400 resin in chloride form, loaded and eluted in MeOH-H_2O (1:1)] to afford phosphonium salt as a white-yellow hygroscopic solid (114 mg, 0.281 mmol, 74%). ν_{max} (ATR): 3053 (CH), 2990 (CH), 2932 (CH), 2877 (CH), 1587 (Ph), 1484 (Ph), 1437 (PC) cm^{-1}. δ_H (500 MHz, $CDCl_3$): 7.86-7.62 (15H, m, 15 × CH, Ar), 3.16 (3H, d, *J* = 13.2 Hz, CH_3). δ_C (126 MHz, $CDCl_3$): 135.31 (d, *J* = 2.9 Hz, CH), 133.39 (d, *J* = 10.8 Hz, CH), 130.60 (d, *J* = 13.1 Hz, CH), 118.95 (d, *J* = 88.8 Hz, C), 11.67 (d, *J* = 57.0 Hz, CH_3). δ_P (202 MHz, $CDCl_3$): 21.65 (PPh_3). LRMS (ESI^+): 277 (M^+, 100%). HRMS (ESI^+): 277.1146. $C_{19}H_{18}P^+$ requires M^+, 277.1141. ^{1}H and ^{13}C NMR data agree with literature (Le Page et al., 2015).

(4-Carboxyphenyl)diphenylmethylphosphonium chloride 3. 4-Diphenylphosphinobenzoic acid (100 mg, 0.327 mmol, 1 eq.) and iodomethane (102 µL, 1.63 mmol, 5 eq.) were dissolved in dry toluene (1 mL) under an atmosphere of argon while stirring. The reaction mixture was stirred at RT for 16 h, the formed solid was filtered under reduced pressure and purified dissolving it in the minimum amount of acetonitrile (2 mL) and precipitating it from diethyl ether (10 mL). It was then collected by filtration and ion exchanged to the chloride form [IRA 400 resin in chloride form, loaded and eluted in MeOH-H_2O (1:1)] achieving the phosphonium salt **3** as a white-yellow foam (117 mg, 0.262 mmol, 80%). ν_{max} (ATR): 3056 (CH), 2996 (CH), 2951 (CH), 2882 (CH), 1719 (C=O), 1587 (Ph), 1565 (Ph), 1438 (PC) cm^{-1}. δ_H (500 MHz, CD_3CN): 8.24 (2H, dd, *J* = 8.6, 3.0 Hz, 2 × CH, Ar), 7.91-7.86 (2H, m, 2 × CH, Ar), 7.78 (2H, dd, *J* = 8.6, 4.7 Hz, 2 × CH, Ar), 7.75-7.65 (8H, m, 8 × CH, Ar), 2.86 (3H, d, *J* = 14.0 Hz, CH_3). δ_C (126 MHz, CD_3CN): 166.45 (C), 137.39 (d, *J* = 3.7 Hz, C), 136.28 (d, *J* = 2.8 Hz, CH), 134.64 (d, *J* = 11.2 Hz, CH), 134.34 (d, *J* = 10.6 Hz, CH), 131.58 (d, *J* = 13.4 Hz, CH), 131.22 (d, *J* = 13.3 Hz, CH), 125.16 (d, *J* = 87.4 Hz, C), 119.75 (d, *J* = 89.0 Hz, C), 9.34 (d, *J* = 57.7 Hz, CH_3). δ_P (202 MHz, CD_3CN): 22.16 (PPh_2). LRMS (ESI^+): 321 (M^+, 100%). HRMS (ESI^+): 321.1037. $C_{20}H_{18}O_2P^+$ requires M^+, 321.1039.

(4-Carboxyphenyl)diphenylhexylphosphonium chloride 4. 4-Diphenylphosphinobenzoic acid **20** (200 mg, 0.653 mmol, 1.0 eq.) and 1-bromohexane (1.16 mL, 8.26 mmol, 12.7 eq.) were dissolved in anhydrous toluene (3 mL) while stirring. The reaction was conducted at reflux under an inert atmosphere of argon for 16 h while stirring. After cooling to RT, the solvent was decanted and a yellow oil was collected, washed with brine (3 × 20 mL), extracted with dichloromethane (3 × 20 mL), dried over magnesium sulfate, filtered and concentrated under reduced pressure. The crude material was purified by column chromatography [SiO_2, dichloromethane-methanol from (10:0) to (8.5:1.5)] and ion exchanged to the chloride form [IRA 400 resin in chloride form, loaded and eluted in MeOH-H_2O (1:1)] to yield the phosphonium salt **4** (279 mg, 0.653 mmol, quant. yield). R_f [SiO_2, dichloromethane-methanol (9:1)] = 0.13. ν_{max} (ATR): 2930 (CH), 2866 (CH), 1705 (C=O), 1439 (PC) cm^{-1}. δ_H (400 MHz, $CDCl_3$): 8.10 (2H, dd, *J* = 8.0, 3.0 Hz, 2 × CH, Ar), 7.93-7.66 (12H, m, 12 × CH, Ar), 3.88-3.70 (2H, m, CH_2P), 1.71-1.54 (4H, m, 2 × CH_2), 1.31-1.14 (4H, m, 2 × CH_2), 0.79 (3H, t, *J* = 7.0 Hz, CH_3). δ_C (101 MHz, $CDCl_3$): 166.09 (C), 138.74 (d, *J* = 2.4 Hz, C), 135.19 (d, *J* = 2.9 Hz, CH), 133.95 (d, *J* = 9.9 Hz, CH), 133.48 (d, *J* = 10.4 Hz, CH), 131.16 (d, *J* = 12.8 Hz, CH), 130.67 (d, *J* = 12.5 Hz, CH), 122.00 (d, *J* = 84.9 Hz, C), 118.20 (d, *J* = 85.6 Hz, C), 31.42 (d, *J* = 1.0 Hz, CH_2), 30.27 (d, *J* = 15.9 Hz, CH_2), 22.79 (d, *J* = 4.2 Hz, CH_2), 22.56 (d, *J* = 50.5 Hz, CH_2), 22.40 (CH_2), 14.10 (CH_3). δ_P (162 MHz, $CDCl_3$): 24.47 (PPh_2). LRMS (ESI^+): 391 (M^+, 100%). HRMS (ESI^+): 391.1807. $C_{25}H_{28}O_2P^+$ requires M^+, 391.1807.

(4-Carboxyphenyl)diphenyldodecylphosphonium bromide 5. 4-Diphenylphosphinobenzoic acid **20** (100 mg, 0.327 mmol, 1 eq.) and bromododecane (135 µL, 0.653 mmol, 2 eq.) were dissolved in anhydrous toluene (1 mL) under an atmosphere of argon while stirring. The reaction mixture was stirred under reflux for 7 days. After cooling to RT, the precipitate was filtered and further purified by dissolving it in the minimum amount of dichloromethane (2 mL) and triturating it from diethyl ether (10 mL). The solvent was decanted to give the phosphonium salt **5** as a yellow sticky foam (166 mg, 0.315 mmol, 97%). ν_{max} (ATR): 2924 (CH), 2853 (CH), 1717 (C=O), 1439 (PC) cm^{-1}. δ_H (500 MHz, $CDCl_3$): 8.13-7.99 (2H, m, 2 × CH, Ar), 7.99-7.66 (12H, m, 12 × CH, Ar), 3.85-3.71 (2H, m, CH_2P), 1.69-1.54 (4H, m, 2 × CH_2), 1.36-1.10 (16H, m, 8 × CH_2), 0.87 (3H, t, *J* = 6.9 Hz, CH_3). δ_C (126 MHz, $CDCl_3$): 165.56 (C), 136.97 (d, *J* = 2.9 Hz, C), 135.30 (d, *J* = 2.1 Hz, CH), 133.98 (d, *J* = 10.1 Hz, CH), 133.81 (d, *J* = 10.4 Hz, CH), 131.18 (d, *J* = 11.9 Hz, CH), 130.77 (d, *J* = 12.6 Hz, CH), 123.08 (d, *J* = 84.4 Hz, C), 117.87 (d, *J* = 85.4 Hz, C), 32.00 (CH_2), 30.62 (d, *J* = 15.9 Hz, CH_2), 21.71 (CH_2), 29.69 (CH_2), 29.65 (CH_2), 29.43 (CH_2), 29.36 (CH_2), 22.74 (d, *J* = 48.4 Hz, CH_2), 22.82 (CH_2), 22.78 (CH_2), 14.24 (CH_3). δ_P (162 MHz, $CDCl_3$): 24.57 (PPh_2). LRMS (ESI^+): 475 (M^+, 100%). HRMS (ESI^+): 475.2743. $C_{31}H_{40}O_2P^+$ requires M^+, 475.2760. ^1H and ^{13}C NMR data agree with literature (Shabana et al., 1990).

[4-(Carboxymethyl)phenyl]diphenylmethylphosphonium iodide 6. 4-(Diphenylphosphino)phenylacetic **21** (200 mg, 0.624 mmol, 1 eq.) and iodomethane (195 µL, 3.12 mmol, 5 eq.) were dissolved in anhydrous toluene (3 mL) while stirring under an atmosphere of argon. The reaction mixture was heated at RT for 72 h while stirring. It was then cooled down to RT and the white solid precipitated was filtered under reduced pressure and washed with diethyl ether (3 × 20 mL). It was then ion exchanged to the chloride form [IRA 400 resin in chloride form, loaded and eluted in MeOH-H_2O (1:1)] to yield phosphonium salt **6** as a white-yellow foam (263 mg, 0.569 mmol, 91%). ν_{max} (ATR): 3053 (OH), 2886 (CH), 1728 (C=O), 1601 (Ar), 1587 (Ar), 1485 (Ar), 1439 (PC) cm^{-1}. δ_H (400 MHz, $CDCl_3$): 7.87-7.78 (2H, m, 2 × CH, Ar), 7.78-7.67 (10H, m, 10 × CH, Ar), 7.67-7.60

(2H, m, 2 × CH, Ar), 3.82 (2H, s, CH_2), 3.14 (3H, d, J = 13.2 Hz, CH_3). δ_C (101 MHz, $CDCl_3$): 173.03 (C), 142.16 (d, J = 3.2 Hz, C), 135.42 (d, J = 3.0 Hz, CH), 133.71 (d, J = 11.2 Hz, CH), 133.41 (d, J = 10.7 Hz, CH), 131.88 (d, J = 13.3 Hz, CH), 130.71 (d, J = 12.9 Hz, CH), 119.12 (d, J = 88.7 Hz, C), 117.33 (d, J = 90.2 Hz, C), 41.39 (CH_2), 11.62 (d, J = 57.3 Hz, CH_3). δ_P (162 MHz, $CDCl_3$): 21.38 (PPh_2). LRMS (ESI$^+$): 335 (M^+, 100%). HRMS (ESI$^+$): 335.1181. $C_{21}H_{20}O_2P^+$ requires M^+, 335.1195.

[4-(Carboxymethyl)phenyl]diphenylhexylphosphonium chloride 7. 4-(Diphenylphosphino)phenylacetic **21** (700 mg, 2.19 mmol, 1.0 eq.) and 1-bromohexane (2.2 mL, 15.7 mmol, 7.2 eq.) were dissolved in anhydrous toluene (10 mL) while stirring. The reaction mixture was heated to reflux under an inert atmosphere of argon while stirring for 72 h. After cooling to RT, the solvent was decanted, the resulting yellow oil was dissolved in dichloromethane (5 mL) and triturated from diethyl ether (150 mL). The solvent was decanted and the mixture purified by column chromatography [SiO_2, dichloromethane-methanol from (10:0) to (8:2)] and ion exchanged to the chloride form [IRA 400 resin in chloride form, loaded and eluted in MeOH-H_2O (1:1)] to yield the carboxylic acid **7** as a white foam (744 mg, 1.69 mmol, 77%). R_f [SiO_2, dichloromethane-methanol (9:1)] = 0.08. ν_{max} (ATR): 2930 (CH), 2868 (CH), 1721 (C=O), 1439 (PC) cm^{-1}. δ_H (400 MHz, $CDCl_3$): 7.85-7.55 (14H, m, 14 × CH, Ar), 3.90 (2H, s, CH_2CO), 3.53-3.33 (2H, m, CH_2P), 1.70-1.47 (4H, m, 2 × CH_2), 1.32-1.14 (4H, m, 2 × CH_2), 0.79 (3H, t, J = 6.8 Hz, CH_3). δ_C (101 MHz, $CDCl_3$): 171.55 (C), 143.66 (d, J = 3.0 Hz, C), 135.23 (d, J = 2.9 Hz, CH), 133.59 (d, J = 9.9 Hz, CH), 133.45 (d, J = 10.3 Hz, CH), 132.02 (d, J = 12.9 Hz, CH), 130.64 (d, J = 12.5 Hz, CH), 118.45 (d, J = 86.0 Hz, C), 115.66 (d, J = 87.6 Hz, C), 42.10 (CH_2), 31.21 (d, J = 0.8 Hz, CH_2), 30.26 (d, J = 15.7 Hz, CH_2), 22.83 (d, J = 50.7 Hz, CH_2), 22.58 (d, J = 4.2 Hz, CH_2), 22.34 (CH_2), 14.01 (CH_3). δ_P (162 MHz, $CDCl_3$): 23.55 (PPh_2). LRMS (ESI$^+$): 405 (M^+, 100%). HRMS (ESI$^+$): 405.1966. $C_{26}H_{30}O_2P^+$ requires M^+, 405.1978.

[4-(Carboxymethyl)phenyl]diphenyldodecylphosphonium bromide 8. 4-(Diphenylphosphino)phenylacetic acid **21** (200 mg, 0.624 mmol, 1.0 eq.) and 1-bromododecane (375 µL, 1.56 mmol, 2.5 eq.) were dissolved in anhydrous toluene while stirring (3 mL) under an atmosphere of argon. The reaction mixture was heated under reflux for 72 h while stirring. After cooling to RT, the crude was concentrated at reduced pressure and purified by column chromatography [SiO_2, CH_2Cl_2-MeOH, gradient from (90:10) to (82:18)] to yield the phosphonium salt **8** as a yellow/brown oil (316 mg, 0.555 mmol, 89%). R_f [SiO_2, CH_2Cl_2-MeOH (80:10)] = 0.08. ν_{max} (ATR): 2924 (CH), 2855 (CH), 1730 (C=O), 1601 (Ar), 1466 (CH), 1439 (PC) cm^{-1}. δ_H (400 MHz, $CDCl_3$): 7.85-7.78 (2H, m, 2 × CH, Ar), 7.78-7.63 (12H, m, 12 × CH, Ar), 3.92 (2H, s, CH_2CO), 3.52-3.39 (2H, m, CH_2P), 1.69-1.52 (4H, m, 2 × CH_2), 1.33-1.13 (16H, m, 8 × CH_2), 0.86 (3H, t, J = 6.9 Hz, CH_3). δ_C (101 MHz, $CDCl_3$): 171.75 (C), 143.16 (d, J = 3.1 Hz, C), 135.23 (d, J = 2.8 Hz, CH), 133.66 (d, J = 10.4 Hz, CH), 133.59 (d, J = 10.2 Hz, CH), 132.00 (d, J = 12.9 Hz, CH), 130.66 (d, J = 12.5 Hz, CH), 118.43 (d, J = 86.1 Hz, C), 115.98 (d, J = 87.5 Hz, C), 42.02 (CH_2), 31.98 (CH_2), 30.64 (d, J = 15.6 Hz, CH_2), 29.67 (CH_2), 29.60 (CH_2), 29.40 (CH_2), 29.34 (CH_2), 29.20 (CH_2), 22.98 (d, J = 50.4 Hz, CH_2), 22.76 (CH_2), 22.67 (d, J = 4.6 Hz, CH_2), 14.21 (CH_3). δ_P (162 MHz, $CDCl_3$): 23.64 (PPh_2). LRMS (ESI$^+$): 489 (M^+, 100%). HRMS (ESI$^+$): 489.2894. $C_{32}H_{42}O_2P^+$ requires M^+, 489.2917.

3-(4’-Diphenylmethylphosphonio)phenylpropionic acid chloride salt 9. 1M Hydrochloric acid (1 mL) was added to a solution of ester **27** (76 mg, 0.18 mmol, 1.0 eq) in MeCN (1 mL) and the solution was heated to 90 °C for 5 h. After cooling to R.T. solution was extracted into CH_2Cl_2 (2 × 10 mL). The organic layer was then washed with NaCl (20 mL), dried over

sodium sulfate and concentrated under vacuum to give the carboxylic acid **9** as a pale yellow oil (56 mg, 79%). δ_H (400 MHz: *d*3-MeCN): 7.86-7.81 (2H, m, ArH), 7.71-7.67 (8H, m, ArH), 7.65 (1H, d, *J* = 8.4 Hz, Ar-H), 7.62-7.58 (3H, m, Ar-H), 3.02 (2H, t, *J* = 7.4 Hz, Ar*CH₂*), 2.89 (3H, d, *J* = 13.9 Hz, PCH_3), 2.71 (2H, t, *J* = 7.4 Hz, $ArCH_2$*CH_2*). δ_C (100 MHz: *d*3-MeCN): 173.99 (C), 150.49 (d, *J* = 3.1 Hz, C), 135.89 (d, *J* = 3.1 Hz, CH), 134.26 (d, *J* = 10.7 Hz, CH), 134.15 (d, *J* = 10.6 Hz, CH), 131.16 (d, *J* = 13.3 Hz, CH), 131.05 (d, *J* = 12.9 Hz, CH), 120.61 (d, *J* = 89.0 Hz, C), 117.42 (d, *J* = 90.8 Hz, C), 35.61 (CH_2), 31.45 (CH_2), 9.58 (d, *J* = 58.2 Hz, CH_3). δ_P (162 MHz: *d*3-MeCN): 21.23 (s). m/z (ESI): Found: 349.1346. $C_{22}H_{22}O_2P$ requires (M^+), 349.1352.

3-(4'-diphenylbutylphosphonio)phenylpropionic acid chloride salt 10. Folowing the same procedure as for compound **9**, ester **28** (29 mg, 0.064 mmol, 1.0 eq) gave the carboxylic acid **10** as a colourless oil (20 mg, 73%). δ_H (400 MHz: $CDCl_3$): 7.75-7.71 (2H, m, ArH), 7.67-7.61 (8H, m, ArH), 7.55-7.49 (2H, m, H- 2',H-3', H-5'+ H-6'), 3.37-3.30 (2H, m, PCH_2), 2.96 (2H, t, *J* = 7.3 Hz, ArCH_2), 2.78 (2H, t, *J* = 7.3 Hz, $ArCH_2CH_2$), 1.57-1.48 (4H, m, 2 × CH_2), 0.84 (3H, t, *J* = 6.4 Hz, CH_2CH_3). δ_C (100 MHz: $CDCl_3$): 174.15 (C), 149.89 (d, *J* = 3.0 Hz, C), 135.20 (d, *J* = 3.0 Hz, CH), 133.59 (d, *J* = 10.3 Hz, CH), 133.55 (d, *J* = 9.9 Hz, CH), 131.07 (d, *J* = 12.9 Hz, CH), 130.64 (d, *J* = 12.5 Hz, CH), 118.49 (d, *J* = 86.1 Hz, C), 114.78 (d, *J* = 88.1 Hz, C), 35.76 (CH_2), 31.22 (CH_2), 24.55 (d, *J* = 4.5 Hz, CH_2), 23.89 (d, *J* = 16.5 Hz, CH_2), 22.64 (d, *J* = 51.3 Hz, CH_2), 13.70 (d, *J* = 1.1 Hz, CH_3). δ_P (162 MHz: $CDCl_3$): 23.43 (s). m/z (ESI): Found: 391.1827. $C_{25}H_{28}O_2P$ requires (M^+), 391.1821.

3-(4'-diphenylhexylphosphonio)phenylpropionic acid chloride salt 11. Folowing the same procedure as for compound **9**, ester **29** (16 mg, 0.033 mmol, 1.0 eq) gave the carboxylic acid **11** as a colourless oil (13 mg, 86%). δ_H (400 MHz: $CDCl_3$): 7.81-7.77 (2H, m, ArH), 7.73-7.68 (8H, m, ArH), 7.59- 7.57 (3H, m, Ar-H), 7.54 (1H, d, *J* = 8.3 Hz, Ar-H), 6.25 (1H, board s, OH), 3.38-3.31 (2H, m, PCH_2), 3.02 (2H, t, *J* = 7.3 Hz, ArCH_2), 2.85 (2H, t, *J* = 7.3 Hz, $ArCH_2CH_2$), 1.57-1.48 (4H, m, 2 × CH_2), 1.65-1.51 (4H, m, 2 × CH_2), 1.29-1.17 (4H, m, 2 × CH_2), 0.81 (3H, t, *J* = 6.9 Hz, CH_2CH_3). δ_C (100 MHz: $CDCl_3$): 174.23 (C), 150.09 (d, *J* = 3.1 Hz, C), 135.25 (d, *J* = 3.0 Hz, CH), 133.56 (d, *J* = 10.3 Hz, CH), 133.54 (d, *J* = 9.9 Hz, CH), 131.15 (d, *J* = 13.0 Hz, CH), 130.68 (d, *J* = 12.5 Hz, CH), 118.49 (d, *J* = 86.0 Hz, C), 114.65 (d, *J* = 88.2 Hz, C), 35.86 (CH_2), 31.30 (CH_2), 31.22 (d, *J* = 1.1 Hz, CH_2), 30.34 (d, *J* = 15.8 Hz, CH_2), 22.92 (d, *J* = 51.1 Hz, CH_2), 22.59 (d, *J* = 4.5 Hz, CH_2), 22.40 (CH_2), 14.05 (CH_3). δ_P (162 MHz: $CDCl_3$): 23.35 (s). m/z (ESI): Found: 419.2134. $C_{27}H_{32}O_2P$ requires (M^+), 419.2134.

[4-(Ethoxycarbonyl)phenyl]diphenylmethylphosphonium chloride 12. Carboxylic acid **3** (100 mg, 0.280 mmol, 1 eq.) was dissolved in anhydrous ethanol (3 mL) while stirring and catalytic amount of concentrated sulfuric acid was added (1 drop). The reaction mixture was heated at reflux for 16 h while stirring under an inert atmosphere of argon. After cooling to RT the mixture was washed with saturated sodium bicarbonate (20 mL), extracted with dichloromethane (3 × 20 mL), washed with brine (30 mL), dried over magnesium sulfate, filtered and concentrated under reduced pressure. The resultant compound was ion exchanged to the chloride form [IRA 400 resin in chloride form, loaded and eluted in MeOH-H_2O (1:1)] giving ester **12** as a white foam (67 mg, 0.174 mmol, 62%). ν_{max} (ATR): 2986 (CH), 2886 (CH), 1713 (C=O), 1435 (PC) cm^{-1}. δ_H (400 MHz, $CDCl_3$): 8.30 (2H, dd, *J* = 8.6, 3.1 Hz, 2 × CH, Ar), 7.97 (2H, dd, *J* = 13.0, 8.6 Hz, 2 × CH, Ar), 7.86-7.75 (6H, m, 6 × CH, Ar), 7.75-7.65 (4H, m, 4 × CH, Ar), 4.42 (2H, q, *J* = 7.1 Hz, CH_2), 3.51 (3H, d, *J* = 13.4 Hz, CH_3P), 1.40 (3H, t, *J* = 7.1 Hz, C**H₃**CH₂). δ_C (101 MHz, $CDCl_3$): 164.86 (C), 136.44 (d, *J* = 3.1 Hz, C), 135.35 (d, *J* = 3.0 Hz, CH), 133.77 (d, *J* = 11.1 Hz, CH), 133.50 (d, *J* = 10.8 Hz, CH),

131.08 (d, J = 13.1 Hz, CH), 130.65 (d, J = 12.9 Hz, CH), 124.24 (d, J = 86.9 Hz, C), 119.06 (d, J = 88.5 Hz, C), 62.18 (CH_2), 14.34 (CH_3), 10.61 (d, J = 56.0 Hz, CH_3P). δ_P (162 MHz, $CDCl_3$): 22.56 (PPh_2). LRMS (ESI^+): 394 (M^+, 100%). HRMS (ESI^+): 394.1314. $C_{22}H_{22}O_2P^+$ requires M^+, 394.1352.

[4-(Ethoxycarbonyl)phenyl]diphenylhexylphosphonium chloride 13. Carboxylic acid **4** (300 mg, 0.703 mmol, 1 eq.) was dissolved in 6 mL of anhydrous ethanol while stirring and catalytic amount of sulfuric acid (1 drop) was added. The reaction mixture was heated at reflux for 16 h under an inert atmosphere of argon while stirring. After cooling to RT, the mixture was washed with saturated sodium bicarbonate (3 × 20 mL), extracted with dichloromethane (30 mL), washed with brine (3 × 20 mL) and concentrated under reduced pressure to yield ester **13** as a yellow foam (85 mg, 0.187 mmol, 27%). ν_{max} (ATR): 2955 (CH), 2861 (CH), 1717 (C=O), 1437 (PC) cm^{-1}. δ_H (400 MHz, $CDCl_3$): 8.30 (2H, dd, J = 8.5, 3.0 Hz, 2 × CH, Ar), 8.06 (2H, dd, J = 12.2, 8.6 Hz, 2 × CH, Ar), 7.93-7.83 (4H, m, 4 × CH, Ar), 7.83-7.75 (2H, m, 2 × CH, Ar), 7.75-7.65 (4H, m, 4 × CH, Ar), 4.41 (2H, q, J = 7.1 Hz, CH_2O), 4.10-3.98 (2H, m, CH_2P), 1.69-1.52 (4H, m, 2 × CH_2), 1.39 (3H, t, J = 7.1 Hz, $\mathbf{CH_3}CH_2O$), 1.30-1.14 (4H, m, 2 × CH_2), 0.80 (3H, t, J = 7.0 Hz, $\mathbf{CH_3}CH_2CH_2$). δ_C (101 MHz, $CDCl_3$): 164.93 (C), 136.23 (d, J = 3.1 Hz, C), 135.20 (d, J = 3.0 Hz, CH), 134.11 (d, J = 10.3 Hz, CH), 133.91 (d, J = 10.0 Hz, CH), 131.05 (d, J = 12.7 Hz, CH), 130.65 (d, J = 12.5 Hz, CH), 123.72 (d, J = 83.9 Hz, C), 118.21 (d, J = 85.5 Hz, C), 62.14 (CH_2), 31.52 (d, J = 1.0 Hz, CH_2), 30.18 (d, J = 15.7 Hz, CH_2), 22.81 (d, J = 4.7 Hz, CH_2), 22.50 (d, J = 48.4 Hz, CH_2), 22.37 (CH_2), 14.34 (CH_3), 14.09 (CH_3). δ_P (162 MHz, $CDCl_3$): 25.04 (s, PPh_2). LRMS (ESI^+): 419 (M^+, 100%). HRMS (ESI^+): 419.2123. $C_{27}H_{32}O_2P^+$ requires M^+, 419.2134.

[4-(Ethoxycarbonylmethyl)phenyl]diphenylmethylphosphonium chloride 14. Phosphine **22** (350 mg, 1.01 mmol, 1 eq.) and iodomethane (125 µL, 2.01 mmol, 2 eq.) were dissolved in anhydrous acetonitrile while stirring. The reaction mixture was heated at reflux under an inert atmosphere of argon while stirring for 16 h. After cooling to RT, the mixture was washed with brine (3 × 30 mL), extracted with dichloromethane (30 mL), dried over magnesium sulfate and concentrated under reduced pressure. It was then dissolved in dichloromethane (3 mL) and triturated from diethyl ether (100 mL). The solvent was decanted, the mixture purified by column chromatography [SiO_2, dichloromethane-methanol (from 10:0 to 8:2)] and ion exchanged to the chloride form [IRA 400 resin in chloride form, loaded and eluted in MeOH-H_2O (1:1)] to yield ester **14** as a yellow oil (219 mg, 0.550 mmol, 55%). R_f [SiO_2, dichloromethane-methanol (9:1)] = 0.53. ν_{max} (ATR): 1728 (C=O), 1439 (PC) cm^{-1}. δ_H (400 MHz, $CDCl_3$): 7.76-7.50 (14H, m, 14 × CH, Ar), 4.04 (2H, q, J = 7.1 Hz, $\mathbf{CH_2}CH_3$), 3.65 (2H, s, CH_2CO), 3.04 (3H, d, J = 13.2 Hz, CH_3P), 1.15 (3H, t, J = 7.1 Hz, $\mathbf{CH_3}CH_2$). δ_C (101 MHz, $CDCl_3$): 169.88 (C), 141.93 (d, J = 3.2 Hz, C), 135.08 (d, J = 3.0 Hz, CH), 133.32 (d, J = 11.1 Hz, CH), 133.06 (d, J = 10.8 Hz, CH), 131.35 (d, J = 13.3 Hz, CH), 130.35 (d, J = 12.9 Hz, CH), 118.60 (d, J = 88.8 Hz, C), 117.11 (d, J = 90.0 Hz, C), 61.19 (CH_2), 40.93 (d, J = 1.2 Hz, CH_2), 14.30 (CH_3), 11.31 (d, J = 57.3 Hz, CH_3). δ_P (162 MHz, $CDCl_3$): 21.31 (PPh_3). LRMS (ESI^+): 363 (M^+, 100%). HRMS (ESI^+): 363.1494. $C_{23}H_{24}O_2P^+$ requires M^+, 363.1508.

[4-(Ethoxycarbonylmethyl)phenyl]diphenylhexylphosphonium chloride 15.
Phosphonium salt **7** (100 mg, 0.227 mmol, 1 eq.) was dissolved in ethanol (3 mL) while stirring and catalytic amount of concentrated sulfuric acid was added (1 drop). The reaction mixture was heated at reflux while stirring under an inert atmosphere of argon for 16 h. After cooling to RT, the reaction mixture was washed with 1 M hydrochloric acid (3 × 20 mL),

saturated sodium bicarbonate (3 × 20 mL), brine (3 × 20 mL), extracted with dichloromethane (20 mL), dried over magnesium sulfate and concentrated under reduced pressure. It was then dissolved in dichloromethane (3 mL), triturated from diethyl ether (50 mL), the solvent was decanted and the ester **15** was isolated as a yellow oil (73 mg, 0.147 mmol, 65%). ν_{max} (ATR): 2936 (CH), 2868 (CH), 1732 (C=O), 1653 (C=O), 1437 (PC) cm^{-1}. δ_H (400 MHz, $CDCl_3$): 7.85-7.53 (14H, m, 14 × CH, Ar), 4.11 (2H, q, *J* = 7.1 Hz, C**H$_2$**CH_3), 3.74-3.59 (2H, m, CH_2P), 3.69 (2H, s, CH_2CO), 1.62-1.46 (4H, m, 2 × CH_2), 1.25-1.09 (4H, m, 2 × CH_2), 1.21 (3H, t, *J* = 7.1 Hz, C**H$_3$**CH_2O), 0.74 (3H, t, *J* = 6.9 Hz, C**H$_3$**CH_2CH_2). δ_C (101 MHz, $CDCl_3$): 170.18 (C), 141.91 (d, *J* = 3.1 Hz, C), 135.03 (d, *J* = 3.0 Hz, CH), 133.84 (d, *J* = 10.3 Hz, CH), 133.62 (d, *J* = 10.0 Hz, CH), 131.54 (d, *J* = 12.8 Hz, CH), 130.51 (d, *J* = 12.5 Hz, CH), 118.39 (d, *J* = 85.9 Hz, C), 116.85 (d, *J* = 87.1 Hz, C), 61.43 (CH_2), 41.11 (d, *J* = 1.0 Hz, CH_2), 31.26 (CH_2), 30.09 (d, *J* = 15.6 Hz, CH_2), 22.62 (d, *J* = 49.9 Hz, CH_2), 22.57 (d, *J* = 4.5 Hz, CH_2), 22.22 (CH_2), 14.15 (CH_3), 13.93 (CH_3). δ_P (162 MHz, $CDCl_3$): 24.07 (PPh_2). LRMS (ESI$^+$): 433 (M$^+$, 100%). HRMS (ESI$^+$): 433.2273. $C_{28}H_{34}O_2P^+$ requires M$^+$, 433.2291.

4-(2’,2’,2’-Trifluoroethoxycarbonyl)diphenylmethylphosphonium chloride 16. Phosphine **23** (150 mg, 0.386 mmol, 1 eq.) and iodomethane (48 µL, 0.773 mmol, 2 eq.) were dissolved in anhydrous acetonitrile (3 mL) while stirring. The reaction was heated at reflux for 16 h while stirring. The solution was concentrated under reduced pressure, the obtained oil was dissolved in dichloromethane (2 mL) and triturated with diethyl ether (50 mL). The solvent was decanted, the resulting oil was dissolved in dichloromethane (20 mL), washed with brine (3 × 20 mL), concentrated under reduced pressure and ion exchanged to the chloride form [IRA 400 resin in chloride form, loaded and eluted in MeOH-H_2O (1:1)], to give ester **16** as a yellow oil (133 mg, 0.303 mmol, 78%). ν_{max} (ATR): 2926 (CH), 2873 (CH), 1741 (C=O), 1439 (PC), cm^{-1}. δ_H (400 MHz, $CDCl_3$): 8.34 (2H, dd, *J* = 8.7, 3.0 Hz, 2 × CH, Ar), 8.02 (2H, dd, *J* = 12.9, 8.7 Hz, 2 × CH, Ar), 7.86-7.76 (6H, m, 6 × CH, Ar), 7.65-7.67 (4H, m, 4 × CH, Ar), 4.74 (2H, q, *J* = 8.2 Hz, CH_2), 3.46 (3H, d, *J* = 13.4, CH_3). δ_C (101 MHz, $CDCl_3$): 163.31 (C), 135.56 (d, *J* = 3.0 Hz, CH), 134.25 (C), 134.17 (d, *J* = 11.1 Hz, CH), 133.58 (d, *J* = 10.8 Hz, CH), 131.41 (d, *J* = 13.1 Hz, CH), 130.74 (d, *J* = 13.0 Hz, CH), 125.47 (d, *J* = 86.5 Hz, C), 122.85 (q, *J* = 276.2 Hz, C), 118.52 (d, *J* = 88.5 Hz, C), 61.48 (q, *J* = 37.1 Hz, CH_2), 11.31 (d, *J* = 55.9 Hz, CH_3). δ_P (162 MHz, $CDCl_3$): 22.64 (PPh_2). δ_F (376 MHz, $CDCl_3$): −73.55 (t, *J* = 8.2 Hz, CF_3). LRMS (ESI$^+$): 403 (M$^+$, 100%). HRMS (ESI$^+$): 403.1051. $C_{22}H_{19}F_3O_2P^+$ requires M$^+$, 403.1069.

4-(2’,2’,2’-Trifluoroethoxycarbonyl)diphenylhexylphosphonium chloride 17. Phosphine **23** (160 mg, 0.412 mmol, 1 eq.) and 1-bromohexane (580 µL, 4.12 mmol, 10 eq.) were dissolved in anhydrous acetonitrile (3 mL) while stirring. The reaction was conducted under an inert atmosphere of argon at reflux for 72 h while stirring. The reaction mixture was washed with brine (3 × 20 mL), extracted with dichloromethane (20 mL), concentrated under reduced pressure and ion exchanged to the chloride form [IRA 400 resin in chloride form, loaded and eluted in MeOH-H_2O (1:1)] to give ester **17** as a yellow oil (153 mg, 0.301 mmol, 73%). ν_{max} (ATR): 2926 (CH), 2857 (CH), 1741 (C=O), 1439 (PC) cm^{-1}. δ_H (400 MHz, $CDCl_3$): 8.28 (2H, dd, *J* = 8.5, 2.9 Hz, 2 × CH, Ar), 8.09 (2H, dd, *J* = 12.2, 8.4 Hz, 2 × CH, Ar), 7.93-7.74 (6H, m, 6 × CH, Ar), 7.74-7.62 (4H, m, 4 × CH, Ar), 4.69 (2H, q, *J* = 8.3 Hz, CH_2), 3.98-3.80 (2H, m, CH_2P), 1.69-1.49 (4H, m, 2 × CH_2), 1.27-1.08 (4H, m, 2 × CH_2), 0.75 (3H, t, *J* = 7.0 Hz, CH_3). δ_C (101 MHz, $CDCl_3$): 163.52 (C), 135.29 (d, *J* = 2.9 Hz, CH), 134.45 (d, *J* = 10.5 Hz, CH), 133.98 (d, *J* = 10.0 Hz, CH), 131.41 (d, *J* = 12.6 Hz, CH), 130.70 (d, *J* = 12.6 Hz, CH), 125.26 (d, *J* = 85.4 Hz, C), 118.00 (d, *J* = 85.5 Hz, C), 61.48 (q,

J = 37.1 Hz, CH_2), 31.54 (CH_2), 30.19 (d, J = 15.7 Hz, CH_2), 29.84 (CH_2), 22.85 (d, J = 4.7 Hz, CH_2), 22.38 (CH_2), 14.10 (CH_3). δ_P (162 MHz, $CDCl_3$): 25.31 (PPh_2). δ_F (376 MHz, $CDCl_3$): −73.58 (t, J = 8.2 Hz, CF_3). LRMS (ESI$^+$): 473 (M^+, 100%). HRMS (ESI$^+$): 473.1832. $C_{27}H_{29}F_3O_2P^+$ requires M^+, 473.1852.

4-(2',2',2'-Trifluoroethoxycarbonylmethyl)diphenylmethylphosphonium chloride 18. Phosphine **24** (60 mg, 0.149 mmol, 1 eq.) and iodomethane (10 μL, 0.149 mmol, 1 eq.), were dissolved in anhydrous acetonitrile (3 mL) while stirring. The reaction mixture was stirred at reflux under an inert atmosphere of argon for 16 h. After cooling to RT, the solution was concentrated under reduced pressure. The resulting oil was dissolved in dichloromethane (2 mL), triturated from diethyl ether (50 mL), the solvent was decanted and the resulting oil ion exchanged to the chloride form [IRA 400 resin in chloride form, loaded and eluted in MeOH-H_2O (1:1)] to give ester **18** as a white foam (67 mg, 0.149 mmol, quant yield). ν_{max} (ATR): 1755 (C=O), 1439 (PC) cm^{-1}. δ_H (400 MHz, $CDCl_3$): 7.87-7.73 (8H, m, 8 × CH, Ar), 7.73-7.64 (3H, m, 3 × CH, Ar), 7.64-7.58 (3H, m, 3 × CH, Ar), 4.52 (2H, q, J = 8.3 Hz, CH_2CF_3), 3.87 (2H, s, CH_2CO), 3.41 (3H, d, J = 13.4 Hz, CH_3). δ_C (101 MHz, $CDCl_3$): 168.75 (C), 140.59 (d, J = 3.2 Hz, C), 135.19 (d, J = 3.0 Hz, CH), 133.95 (d, J = 11.1 Hz, CH), 133.46 (d, J = 10.7 Hz, CH), 131.54 (d, J = 13.3 Hz, CH), 130.56 (d, J = 12.9 Hz, CH), 122.82 (d, J = 277.3 Hz, C), 119.49 (d, J = 88.6 Hz, C), 118.66 (d, J = 89.7 Hz, C), 60.99 (q, J = 36.8 Hz, CH_2), 40.45 (CH_2), 10.71 (d, J = 56.6 Hz, CH_3). δ_P (162 MHz, $CDCl_3$): 22.04 (PPh_2). δ_F (376 MHz, $CDCl_3$): −73.70 (t, J = 8.3 Hz, CF_3). LRMS (ESI$^+$): 417 (M^+, 100%). HRMS (ESI$^+$): 417.1209. $C_{23}H_{21}F_3O_2P^+$ requires M^+, 417.1226.

4-(2',2',2'-Trifluoroethoxycarbonylmethyl)diphenylhexylphosphonium chloride 19. Phosphonium salt **7** (100 mg, 0.227 mmol, 1 eq.) was dissolved in 1,1,1,-trifluoroethanol while stirring (3 mL) and a catalytic amount (1 drop) of concentrated sulfuric acid was added. The reaction mixture was heated at reflux under an inert atmosphere of argon while stirring for 16 h. After cooling to RT, the solution was washed with 1 M hydrochloric acid (3 × 20 mL), saturated sodium bicarbonate (3 × 20 mL), brine (3 × 20 mL), extracted with dichloromethane (20 mL), dried over magnesium sulfate, filtered and concentrated under reduced pressure. The resultant oil was purified by column chromatography [SiO_2, dichloromethane-methanol from (10:0) to (9:1)] to yield the ester **19** as a yellow oil (35 mg, 0.0665 mmol, 29%). R_f [SiO_2, dichloromethane-methanol (9:1)] = 0.80. ν_{max} (ATR): 2932 (CH), 2866 (CH), 1755 (C=O), 1439 (PC) cm^{-1}. δ_H (500 MHz, $CDCl_3$): 7.93-7.53 (14H, m, 14 × CH, Ar), 4.50 (2H, q, J = 8.4 Hz, CH_2CH_3), 3.87 (2H, s, CH_2CO), 3.80-3.64 (2H, m, CH_2P), 1.68-1.50 (4H, m, 2 × CH_2), 1.32-1.11 (4H, m, 2 × CH_2), 0.79 (3H, t, J = 6.6 Hz, CH_3). δ_C (126 MHz, $CDCl_3$): 168.79 (C), 140.51 (d, J = 3.1 Hz, C), 135.11 (d, J = 2.9 Hz, CH), 134.19 (d, J = 10.3 Hz, CH), 133.77 (d, J = 9.9 Hz, CH), 131.62 (d, J = 12.8 Hz, CH), 130.60 (d, J = 12.5 Hz, CH), 122.78 (q, J = 277.3 Hz, C), 118.45 (d, J = 85.8 Hz, C), 117.66 (d, J = 86.9 Hz, C), 60.91 (q, J = 36.8 Hz, C), 40.38 (CH_2), 31.39 (CH_2), 30.20 (d, J = 15.6 Hz, CH_2), 29.75 (CH_2), 22.75 (d, J = 49.6 Hz, CH_2), 22.70 (d, J = 4.5 Hz, CH_2), 22.32 (CH_2), 14.02 (CH_3). δ_P (202 MHz, $CDCl_3$): 24.24 (PPh_2). δ_F (471 MHz, $CDCl_3$): −73.69 (t, J = 8.3 Hz, CF_3). LRMS (ESI$^+$): 487 (M^+, 100%). HRMS (ESI$^+$): 487.1991. $C_{28}H_{31}F_3O_2P^+$ requires M^+, 487.2008.

4-(Diphenylphosphino)phenylacetic acid 21. Following the procedure reported by Dydio *et al.*, (Dydio et al., 2014) on a 3.82 mmol scale the phenylacetic acid derivative **21** was obtained as a white amorphous solid (1.20 g, 3.82 mmol, quant yield). R_f [SiO_2, CH_2Cl_2-MeOH (85:15)] = 0.75. ν_{max} (ATR): 3071 (OH), 3051 (OH), 1709 (C=O), 1601 (Ph), 1586 (Ph), 1435 (PC) cm^{-1}. δ_H (400 MHz, DMSO-d_6): 12.37 (1H, s, OH), 7.46-7.35 (6H, m, 6 ×

CH, Ar), 7.34-7.15 (8H, m, 8 × CH, Ar), 3.58 (2H, s, CH_2). δ_C (101 MHz, DMSO-d_6): 172.39 (C), 136.75 (d, J = 11.3 Hz, CH), 136.02 (C), 134.60 (d, J = 10.8 Hz, CH), 133.29 (d, J = 20.0 Hz, C), 133.16 (d, J = 19.4 Hz, C), 129.88 (d, J = 7.1 Hz, CH), 128.94 (C), 128.76 (d, J = 6.8 Hz, CH), 40.35 (CH_2). δ_P (162 MHz, DMSO-d_6): -7.44 (PPh_2). LRMS (EI^+): 320 ($M^{\cdot+}$, 100%). HRMS (EI^+): 320.0980. $C_{20}H_{17}O_2P$ requires $M^{\cdot+}$, 320.0966. ^1H and ^{13}C NMR data agree with literature (Dydio et al., 2014)

[4-(Ethoxycarbonylmethyl)phenyl]diphenylmethylphosphine 22. 4-(Diphenylphosphino)phenylacetic **21** (500 mg, 1.56 mmol, 1 eq.), *N,N'*-dicyclohexylcarbodiimide (354 mg, 1.72 mmol, 1.1 eq.), ethanol (100 µL, 1.72 mmol, 1.1 eq.) and 4-dimethylaminopyridine (catalytic amount) were dissolved in anhydrous and degassed acetonitrile (15 mL) at 0 °C. The reaction mixture was warmed up to RT and stirred under an inert atmosphere of argon for 16 h. The urea was filtered off, the liquid phase was washed with 1 M hydrochloric acid (3 × 50 mL), saturated sodium bicarbonate (3 × 50 mL), brine (3 × 50 mL), extracted with ethyl acetate, dried over magnesium sulfate, filtered and concentrated under reduced pressure. The crude was purified by column chromatography [SiO_2, hexane-ethyl acetate from (10:0) to (9:1)] to yield the phosphine **22** as a colourless oil (359 mg, 1.032 mmol, 66%). R_f [SiO_2, hexane-ethyl acetate (9.5:0.5)] = 0.28. δ_H (400 MHz, $CDCl_3$): 7.40-7.24 (14H, m, 14 × CH, Ar), 4.16 (2H, q, J = 7.1 Hz, C**H₂**CH₃), 3.61 (2H, s, CH_2O), 1.26 (3H, t, J = 7.1 Hz, CH_3). δ_C (126 MHz, $CDCl_3$): 171.49 (C), 137.29 (d, J = 10.6 Hz, C), 135.96 (d, J = 10.8 Hz, C), 134.90 (C), 134.11 (d, J = 19.5 Hz, CH), 133.86 (d, J = 19.4 Hz, CH), 129.55 (d, J = 6.8 Hz, CH), 128.86 (CH), 128.63 (d, J = 6.9 Hz, CH), 61.08 (CH_2), 41.30 (CH_2), 14.31 (CH_3). δ_P (162 MHz, $CDCl_3$): –6.04 (s, PPh_3). The compound is prone to oxidation to form phosphine oxide, so only NMR was used for and the compound was quickly used in the next step.

[4-(2’,2’,2’-Trifluoroethoxycarbonyl)phenyl]diphenylmethylphosphine 23. 4-(Diphenylphosphino)benzoic acid **20** (500 mg, 1.63 mmol, 1 eq.), 1,1,1-trifluoroethanol (131 µL, 1.80 mmol, 1.1 eq.), 4-dimethylaminopyridine (catalytic amount) were dissolved in 10 mL of anhydrous dichloromethane at 0 °C while stirring. A solution of *N,N'*-diisopropylcarbodiimide (358 µL, 1.80 mmol, 1.1 eq.) in 5 mL of anhydrous dichloromethane was added dropwise and the reaction mixture was allowed to warm to RT and stirred at RT for 16 h. the mixture was filtered then concentrated under reduced pressure. The resultant oil was purified by column chromatography [SiO_2, hexane-ethyl acetate from (10:0) to (9.5:0.5)] to yield the phosphine **23** as a colourless oil (338 mg, 0.870 mmol, 53%). R_f [SiO_2, hexane-ethyl acetate (9:1)] = 0.29. ν_{max} (ATR): 3073 (CH), 1738 (C=O), 1597 (C=O), 1435 (PC) cm^{-1}. δ_H (500 MHz, $CDCl_3$): 8.00 (2H, dd, J = 8.5, 1.5 Hz, 2 × CH, Ar), 7.43-7.29 (12H, m, 12 × CH, Ar), 4.69 (2H, q, J = 8.4 Hz, CH_2). δ_C (101 MHz, $CDCl_3$): 164.89 (C), 145.87 (d, J = 15.2 Hz, C), 136.03 (d, J = 10.5 Hz, C), 134.17 (d, J = 20.1 Hz, CH), 133.41 (d, J = 18.5 Hz, CH), 129.80 (d, J = 6.3 Hz, CH), 129.44 (CH), 128.91 (d, J = 7.3 Hz, CH), 128.27 (C), 123.22 (q, J = 277.2 Hz, C), 60.98 (q, J = 36.7 Hz, CH_2). δ_P (162 MHz, $CDCl_3$): –4.74 (PPh_2). δ_F (376 MHz, $CDCl_3$): –73.66 (t, J = 8.4 Hz, CF_3). LRMS (ESI^+): 391 (M^+, 100%). HRMS (ESI^+): 389.0896. $C_{21}H_{17}F_3O_2P^+$ requires $(M+H)^+$, 389.0913.

[4-(2’,2’,2’-Trifluoroethoxycarbonylmethyl)phenyl]diphenylmethylphosphine 24. 4-(Diphenylphosphino)phenylacetic **21** (200 mg, 0.624 mmol, 1 eq.), 1,1,1-trifluoroethanol (45 µL, 0.687 mmol, 1.1 eq.), *N,N'*-dicyclohexylcarbodiimide (142 mg, 0.687 mmol, 1.1 eq.) and 4-dimethylaminopyridine (catalytic amount) were dissolved in anhydrous acetonitrile (10 mL) at 0 °C while stirring. The reaction mixture was allowed to warm to RT and stirred under an inert atmosphere of argon for 72 h. The mixture was filtered and the concentrated

under reduced pressure. The crude was purified by column chromatography [SiO_2, hexane-ethyl acetate (from 10:0 to 9.5:0.5)] to yield the phosphine **24** as a colourless oil (147 mg, 0.365 mmol, 59%). R_f [SiO_2, hexane-ethyl acetate (9.5:0.5)] = 0.24. δ_H (400 MHz, $CDCl_3$): 7.38-7.27 (14H, m, 14 × CH, Ar), 4.48 (2H, q, *J* = 8.4 Hz, CH_2CF_3), 3.72 (2H, s, CH_2CO). δ_C (101 MHz, $CDCl_3$): 169.90 (C), 137.12 (d, *J* = 10.8 Hz, C), 136.69 (C), 134.22 (d, *J* = 19.5 Hz, CH), 133.88 (d, *J* = 19.5 Hz, CH), 133.45 (C), 129.54 (d, *J* = 7.0 Hz, CH), 128.94 (CH), 128.68 (d, *J* = 7.0 Hz, CH), 122.99 (q, *J* = 277.6 Hz, C), 60.79 (q, *J* = 36.7, CH_2), 40.45 (s, CH_2). δ_P (162 MHz, $CDCl_3$): −6.04 (PPh_3). The compound is prone to oxidation to form phosphine oxide, so only NMR was used for and the compound was quickly used in the next step.

3-(4'-Iodophenyl)propanoic acid 25. The iodoarene **25** was synthesised by the method of Qin *et al* (Qin et al., 2015) on a 40.00 mmol scale to give the acid as a white crystals (3.00 g, 27%). δ_H (400 MHz: $CDCl_3$): 7.61 (2H, d, *J* = 8.4 Hz, H-3 + H-5), 6.97 (2H, d, *J* = 8.4 Hz, H-2 + H-6), 2.90 (2H, t, *J* = 7.6 Hz, ArCH_2), 2.68-2.64 (2H, m, $ArCH_2CH_2$).

Ethyl 3-(4-iodophenyl)propionate 26. Concentrated sulfuric acid (2 drops) was added to a solution of 3-(4'-iodophenyl)propanoic acid **25** (1.00 g, 3.62 mmol, 1.0 eq) in ethanol (20 mL) and the solution heated to 80 °C overnight. After cooling to R.T. the solution was concentrated under vacuum, redissolved in diethyl ether (~ 50 mL) and washed with water (2 × 50mL). The organic layer was dried over magnesium sulfate and concentrated under vacuum to give the ester **26** as a colourless oil (960 mg, 87%). δ_H (400 MHz: $CDCl_3$): 7.60 (2H, d, *J* = 8.3 Hz, H-3 + H-5), 6.96 (2H, d, *J* = 8.3 Hz, H-2 + H-6), 4.12 (2H, q, *J* = 7.1 Hz, OCH2), 2.89 (2H, t, *J* = 7.7 Hz, ArCH_2), 2.59 (2H, t, *J* = 7.7 Hz, $ArCH_2CH_2$), 1.23 (3H, t, *J* = 7.1 Hz, CH_3). δ_C (100 MHz: $CDCl_3$): 172.70 (C), 140.36 (C), 137.65 (CH), 130.57 (CH), 91.50 (C), 60.65 (CH_2), 35.75 (CH_2), 30.57 (CH_2), 14.35 (CH_3). m/z (ESI): Found: 326.9846. $C_{11}H_{13}INaO_2$ requires $(M+Na)^+$, 326.9852.

Ethyl 3-[4'-(diphenylmethylphosphonio)phenyl]propionate chloride salt 27. Iodoarene **26** (214 mg, 0.70 mmol, 1.0 eq) and triethylamine (213 µL, 1.54 mmol, 2.2 eq) were dissolved in dry toluene (4 mL). The resulting solution was degassed by bubbling argon through the solution for 5 min, then palladium acetate (3.0 mg, 0.014 mmol, 0.02 eq) was added followed by diphenylphosphine (122 µL, 0.70 mmol, 1.0 eq). The resulting red solution was heated to 100 °C for 2.5 h. After cooling to 80°C iodomethane (435 µL, 7.00 mmol, 10.0 eq) was added and the solution stirred at 80 °C for a further 30 min. The solution was cooled to R.T., concentrated under vacuum then redissolved in CH_2Cl_2 (20 mL). The solution was then washed with 1M hydrochloric acid (2 × 100 mL), dried over sodium sulfate and concentrated under vacuum. The residue was purified by column chromatography using an Agela 20 g column eluting a gradient of CH_2Cl_2:MeOH (100:0) increasing to (92:8) to give the phosphonium salt **27** as a viscous pale yellow oil (283 mg, 97%). δ_H (400 MHz: $CDCl_3$): 7.72-7.65 (2H, m, ArH), 7.63-7.52 (10H, m, ArH + H-3' + H-5'), 7.43 (2H, dd, *J* = 8.3, 3.2 Hz, H-2' + H-6'), 3.98 (2H, q, *J* = 7.1 Hz, OCH_2), 3.00 (3H, d, *J* = 13.2 Hz, PCH_3), 2.93 (2H, t, *J* = 7.5 Hz, ArCH_2), 2.55 (2H, t, *J* = 7.5 Hz, $ArCH_2CH_2$), 1.10 (3H, t, *J* = 7.1 Hz, OCH_2CH_3). δ_C (100 MHz: $CDCl_3$): 171.85 (C), 148.72 (d, *J* = 3.1 Hz, C), 134.97 (d, *J* = 3.1 Hz, CH), 133.25 (d, *J* = 11.1 Hz, CH), 132.94 (d, *J* = 10.7 Hz, CH), 130.34 (d, *J* = 13.3 Hz, CH), 130.27 (d, *J* = 12.9 Hz, CH), 118.73 (d, *J* = 88.8 Hz, C), 115.95 (d, *J* = 90.5 Hz, C), 60.42 (CH_2), 34.46 (CH_2), 30.50 (d, *J* = 1.4 Hz, CH_2), 13.95 (CH_3), 11.16 (d, *J* = 57.4 Hz, CH_3). δ_P (162 MHz: $CDCl_3$): 21.06 (s). m/z (ESI): Found: 377.1660. $C_{24}H_{26}O_2P$ requires (M^+), 377.1665.

Ethyl 3-[4'-(butyldiphenylphosphonio)phenyl]propionate chloride salt 28. Following the same procedure as for compound **27**, iodoarene **26** (271 mg, 0.89 mmol, 1.0 eq), triethylamine (271 μL, 1.96 mmol, 2.2 eq), palladium acetate (4.0 mg, 0.018 mmol, 0.02 eq), diphenylphosphine (155 μL, 0.89 mmol, 1.0 eq) and 1-bromobutane (960 μL, 8.90 mmol, 10.0 eq) gave the phosphonium salt **28** as a viscous pale yellow oil (59 mg, 14%). δ_H (400 MHz: $CDCl_3$): 7.77-7.61 (8H, m, ArH), 7.66-7.61 (4H, m, ArH + H-3' + H-5'), 7.49 (2H, dd, *J* = 8.3, 3.1 Hz, H-2' + H-6'), 4.06 (2H, q, *J* = 7.1 Hz, OCH_2), 3.68-3.57 (2H, m, PCH_2), 3.00 (2H, t, *J* = 7.5 Hz, ArCH_2), 2.62 (2H, t, *J* = 7.5 Hz, ArCH_2CH_2), 1.64-1.43 (4H, m, 2 × CH_2), 1.16 (3H, t, *J* = 7.2 Hz, OCH_2CH_3), 0.83 (3H, t, *J* = 7.0 Hz, CH_2CH_3). δ_C (100 MHz: $CDCl_3$): 172.17 (C), 148.74 (d, *J* = 3.0 Hz, C), 134.97 (d, *J* = 3.1 Hz, CH), 133.82 (d, *J* = 10.3 Hz, CH), 133.55 (d, *J* = 9.9 Hz, CH), 130.56 (d, *J* = 12.8 Hz, CH), 130.47 (d, *J* = 12.5 Hz, CH), 118.52 (d, *J* = 85.9 Hz, C), 115.71 (d, *J* = 87.6 Hz, C), 60.72 (CH_2), 34.69 (CH_2), 30.75 (d, *J* = 1.4 Hz, CH_2), 24.58 (d, *J* = 4.6 Hz, CH_2), 23.70 (d, *J* = 16.4 Hz, CH_2), 22.40 (d, *J* = 50.4 Hz, CH_2), 14.19 (CH_3), 13.71 (d, *J* = 1.2 Hz, CH_3). δ_P (162 MHz: $CDCl_3$): 23.90 (s). m/z (ESI): Found: 419.2129. $C_{27}H_{32}O_2P$ requires (M^+), 419.2134.

Ethyl 3-[4'-(diphenylhexylphosphonio)phenyl]propionate chloride salt 29. Following the same procedure as for compound **27**, iodoarene **26** (253 mg, 0.83 mmol, 1.0 eq), triethylamine (253 μL, 1.82 mmol, 2.2 eq), palladium acetate (4.0 mg, 0.018 mmol, 0.02 eq), diphenylphosphine (144 μL, 0.89 mmol, 1.0 eq) and 1-bromohexane (1.16 mL, 8.3 mmol, 10.0 eq) gave phosphonium salt **29** as a colourless oil (34 mg, 7%) after further purified by reverse phase HPLC using a Luna Omega 5μm PS C18 250×10.0 mm column eluting an isocratic gradient of 41% MeCN:water (0.1% TFA) at 5 mL/min. δ_H (400 MHz: $CDCl_3$): 7.77-7.71 (6H, m, ArH), 7.69-7.63 (6H, m, ArH + H-3' + H-5'), 7.50 (2H, dd, *J* = 8.3, 3.1 Hz, H-2' + H-6'), 4.07 (2H, q, *J* = 7.1 Hz, OCH_2), 3.63-3.58 (2H, m, PCH_2), 3.01 (2H, t, *J* = 7.5 Hz, ArCH_2), 2.64 (2H, t, *J* = 7.5 Hz, ArCH_2CH_2), 1.59-1.52 (4H, m, 2 × CH_2), 1.21-1.14 (4H, m, 2CH_2), 1.18 (3H, t, *J* = 7.1 Hz, OCH_2CH_3), 0.77 (3H, t, *J* = 7.1 Hz, CH_2CH_3). δ_C (100 MHz: $CDCl_3$): 172.22 (C), 148.82 (d, *J* = 3.0 Hz, C), 135.03 (d, *J* = 3.0 Hz, CH), 133.82 (d, *J* = 10.3 Hz, CH), 133.56 (d, *J* = 9.9 Hz, CH), 130.60 (d, *J* = 12.9 Hz, CH), 130.51 (d, *J* = 12.5 Hz, CH), 118.53 (d, *J* = 85.9 Hz, C), 115.71 (d, *J* = 87.7 Hz, C), 60.77 (CH_2), 34.72 (CH_2), 31.28 (d, *J* = 1.2 Hz, CH_2), 30.78 (d, *J* = 1.5 Hz, CH_2) 30.12 (d, *J* = 15.6 Hz, CH_2), 22.59 (d, *J* = 50.2 Hz, CH_2), 22.57 (d, *J* = 4.5 Hz, CH_2), 22.29 (CH_2), 14.21 (CH_3), 13.96 (CH_3). δ_P (162 MHz: $CDCl_3$): 23.85 (s). m/z (ESI): Found: 447.2437. $C_{29}H_{36}O_2P$ requires (M^+), 447.2447.

1.1.3 Preparation of Zwitterion for X-ray Crystallography Studies

(4-Carboxyphenyl)diphenylmethylphosphonium iodide was prepared as for compound **3** above, but not ion exchanged to the chloride salt **3**. The iodide salt, $TPMP^+$-COOH was recrystallised from a minimum volume of MeCN affording single crystals suitable for x-ray diffraction. The iodide salt (200 mg, 0.45 mmol) and Ag_2CO_3 (246 mg, 0.89 mmol) in MeCN (10 mL) were stirred at RT for 3 h. The solids were removed by filtration and washed with MeCN. The combined MeCN solution was concentrated under reduced pressure to give a solid, which was recrystallised from a minimum volume of MeCN affording the pure $TPMP^+$-COO^- as large colourless blocks suitable for single crystal X-ray diffraction studies. (120 mg, 0.37 mmol, 83% yield). ν_{max} (ATR): 2953 (CH) 2887(CH) 1641 (CO) 1600 (CO) 1543 (Ph) 1438 (PC) cm^{-1}. δ_H (400 MHz, MeOD): 8.18 (2H, dd, *J* = 8.2, 3.3 Hz, H-3and H-5), 7.91–7.86 (2H, m, H-2 and H-4), 7.79–7.71 (10H, m, ArH), 3.01 (3H, d, *J* = 14.1 Hz, PMe). δ_C (126 MHz, MeOD):172.51 (C), 146.01 (d, *J* = 3.0 Hz, CH), 136.21 (d, *J* = 3.0 Hz, CH), 134.44 (d, *J* = 10.7 Hz, CH), 134.09 (d, *J* = 11.1 Hz, CH), 131.49 (d, *J* =13.3 Hz, CH),

131.42 (d, J = 12.9 Hz, CH), 121.98 (d J =88.9 Hz, C), 121.13 (d, J =89.0 Hz, C), 8.67 (d J = 57.9 Hz, CH_3). δ_P (202 MHz, MeOD): 21.87 (s).

1.1.4 Crystal data

$C_{20}H_{17}O_2P$, M_r = 320.30, monoclinic, a = 12.1945 (6), b = 10.0034 (4), c = 13.9635 (7) Å, □ = 100.177 (2)°, V = 1676.56 (14) Å^3, T=295K, space group $P2_1/n$ (no. 14), Z = 4, colourless, block-shaped crystal 0.34 × 0.3 × 0.11 mm. 19629 reflections measured, 4146 unique (R_{int} = 0.040), which were used in all calculations. The final $R[F^2 > 2\sigma(F^2)$ 3663 reflections] = 0.057, $wR(F^2)$ = 0.172, 209 parameters.

I·$C_{20}H_{18}O_2P$, M_r = 448.21, orthorhombic, a = 14.9745 (7), b = 14.6269 (6), c = 17.2993 (8) Å V = 3789.1 (3) Å^3, T=150K, $Pbca$ (no. 64), Z = 8, colourless, tablet shaped crystal 0.25 × 0.17 × 0.14 mm. 90476 reflections measured, 4701 unique (R_{int} = 0.047), which were used in all calculations. The final $R[F^2 > 2\sigma(F^2)$ 4509 reflections] = 0.022, $wR(F^2)$ = 0.060, 222 parameters.

Single Crystal x-ray data were collected using APEX3 Ver. 2016.9-0 (Bruker-AXS, 2016) and cell refinement: SAINT V8.37A (Bruker-AXS, 2016); data reduction: APEX3 Ver. 2016.9-0 (Bruker-AXS, 2016). Program(s) used to solve structure: ShelXT (Sheldrick, 2015); program(s) used to refine structure: XL (Sheldrick, 2008); molecular graphics: Olex2 (Dolomanov et al., 2009); software used to prepare material for publication: Olex2 (Dolomanov et al., 2009). Full details are contained in CCDC 1998454 [TPMP$^+$-COO$^-$] and 1998455 (TPMP$^+$-COOH.I-] and can be obtained free of charge from The Cambridge Crystallographic Data Centre via www.ccdc.cam.ac.uk/structure.

Supplemental References

Dolomanov, O. V., Bourhis, L. J., Gildea, R. J., Howard, J. A. K., Puschmann, H. (2009). OLEX2: a complete structure solution, refinement and analysis program. *J. Appl. Cryst*. 42, 339–341.

Dydio, P., Detz, R. J., de Bruin, B., Reek, J. N. (2014). Beyond classical reactivity patterns: hydroformylation of vinyl and allyl arenes to valuable beta- and gamma-aldehyde intermediates using supramolecular catalysis. *J. Am. Chem. Soc*. 136, 8418–8429. doi: 10.1021/ja503033q.

Le Page, M. D., Patrick, B. O., Rettig, S. J., and James, B. R. (2015). 2-Pyridyl-phosphine and -diphosphine complexes of nickel(0), their reactivity (including aqueous solution chemistry), and some related, incidental methylphosphonium iodides. *Inorg. Chim. Acta*. 431, 276–288. doi: 10.1016/j.ica.2015.04.003.

Qin, L., Hu, B., Neumann, K. D., Linstad, E. J., McCauley, K., Veness, J., et al. (2015). A Mild and General One-Pot Synthesis of Densely Functionalized Diaryliodonium Salts. *Eur. J. Org. Chem*. 2015, 5919–5924. doi: 10.1002/ejoc.201500986.

Shabana, R., Amer, A., Mark, H. B., Zimmers, H. (1990). A Convenient Synthesis of long-chain 3-n-alkylthiophenes and 3-n- alkylfurans. *Phosphorus Sulfur Silicon Relat. Elem*. 53, 299–306. doi: 10.1080/10426509008038039.

Sheldrick, G. M. (2008). SHELTX-integrated space-group and crystal structure determination. *Acta Cryst*. A64, 112–122.

Sheldrick, G. M. (2015). Crystal structure refinement with SHELX. *Acta Cryst*. A71, 3–8.
