# Supplementary material for: Enhancing the Mitochondrial Uptake of Phosphonium Cations by Carboxylic Acid Incorporation
Source: Front Chem. 2020 Sep 9;8:783. doi: 10.3389/fchem.2020.00783 (PMC7509049; doi:10.3389/fchem.2020.00783)
Supplement: Supplementary file 2 [file Data_Sheet_2.PDF]

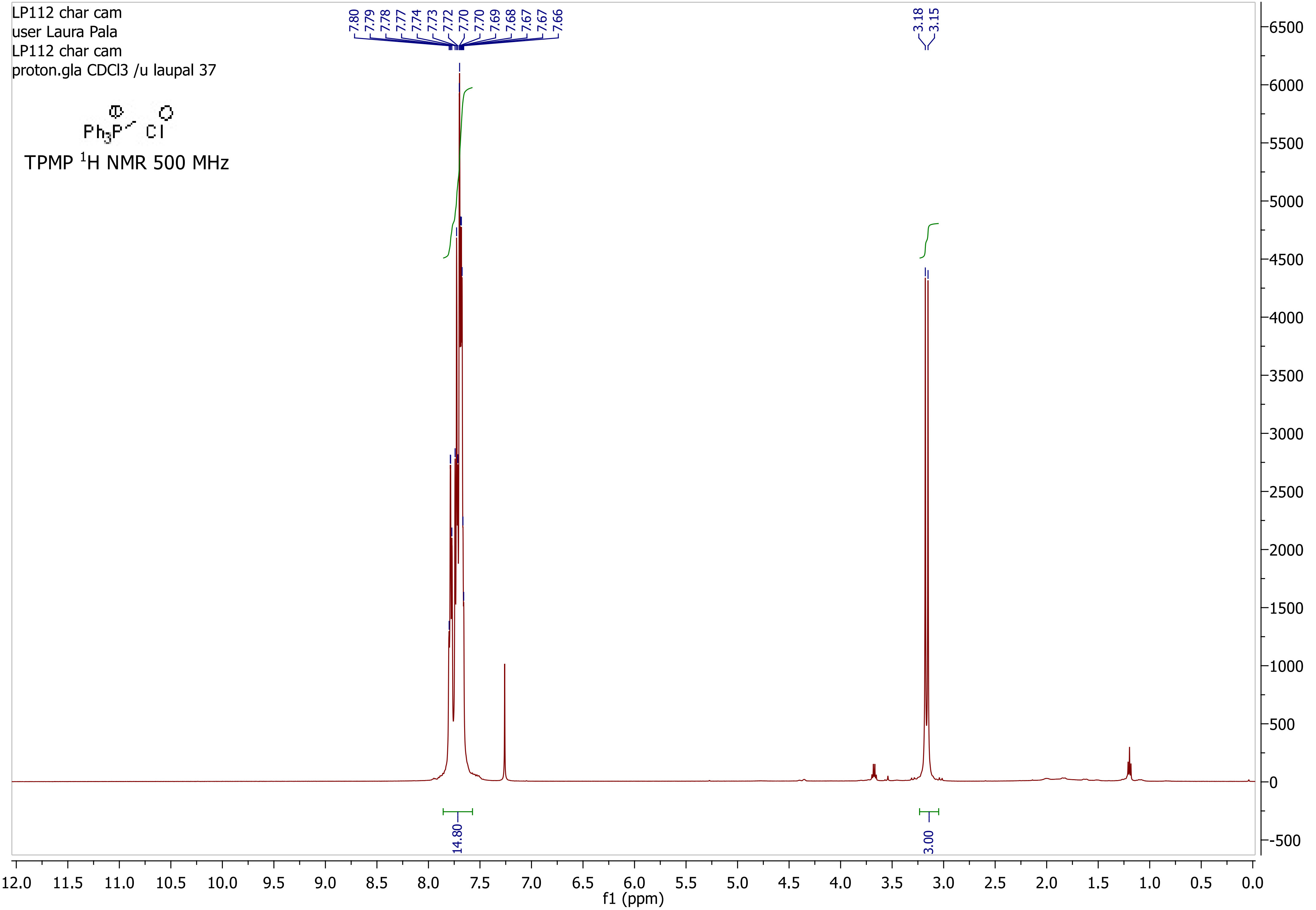
LP112 char cam
user Laura Pala
LP112 char cam
proton.gla CDCl3 /u laupal 37
Ph3P Cl
TPMP 1H NMR 500 MHz
7.80 7.79 7.78 7.77 7.74 7.73 7.72 7.70 7.70 7.69 7.68 7.67 7.67 7.66
3.18 3.15
14.80
3.00
f1 (ppm)
12.0 11.5 11.0 10.5 10.0 9.5 9.0 8.5 8.0 7.5 7.0 6.5 6.0 5.5 5.0 4.5 4.0 3.5 3.0 2.5 2.0 1.5 1.0 0.5 0.0
6500 6000 5500 5000 4500 4000 3500 3000 2500 2000 1500 1000 500 0 -500

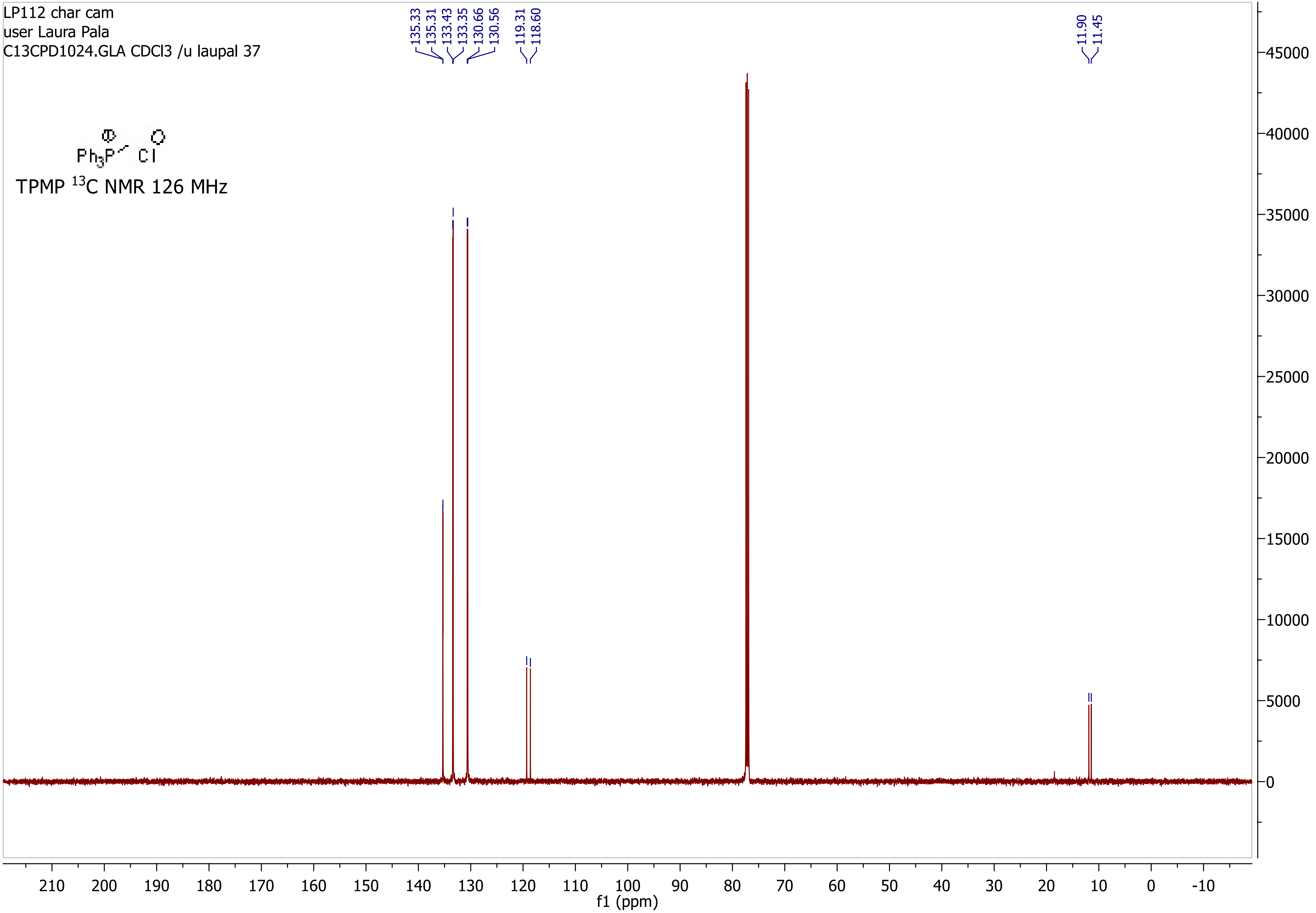
LP112 char cam
user Laura Pala
C13CPD1024.GLA CDCl3 /u laupal 37
Ph3P Cl
TPMP 13C NMR 126 MHz
135.33
135.31
133.43
133.35
130.66
130.56
119.31
118.60
11.90
11.45
45000
40000
35000
30000
25000
20000
15000
10000
5000
0
210
200
190
180
170
160
150
140
130
120
110
100
90
80
70
60
50
40
30
20
10
0
-10
f1 (ppm)

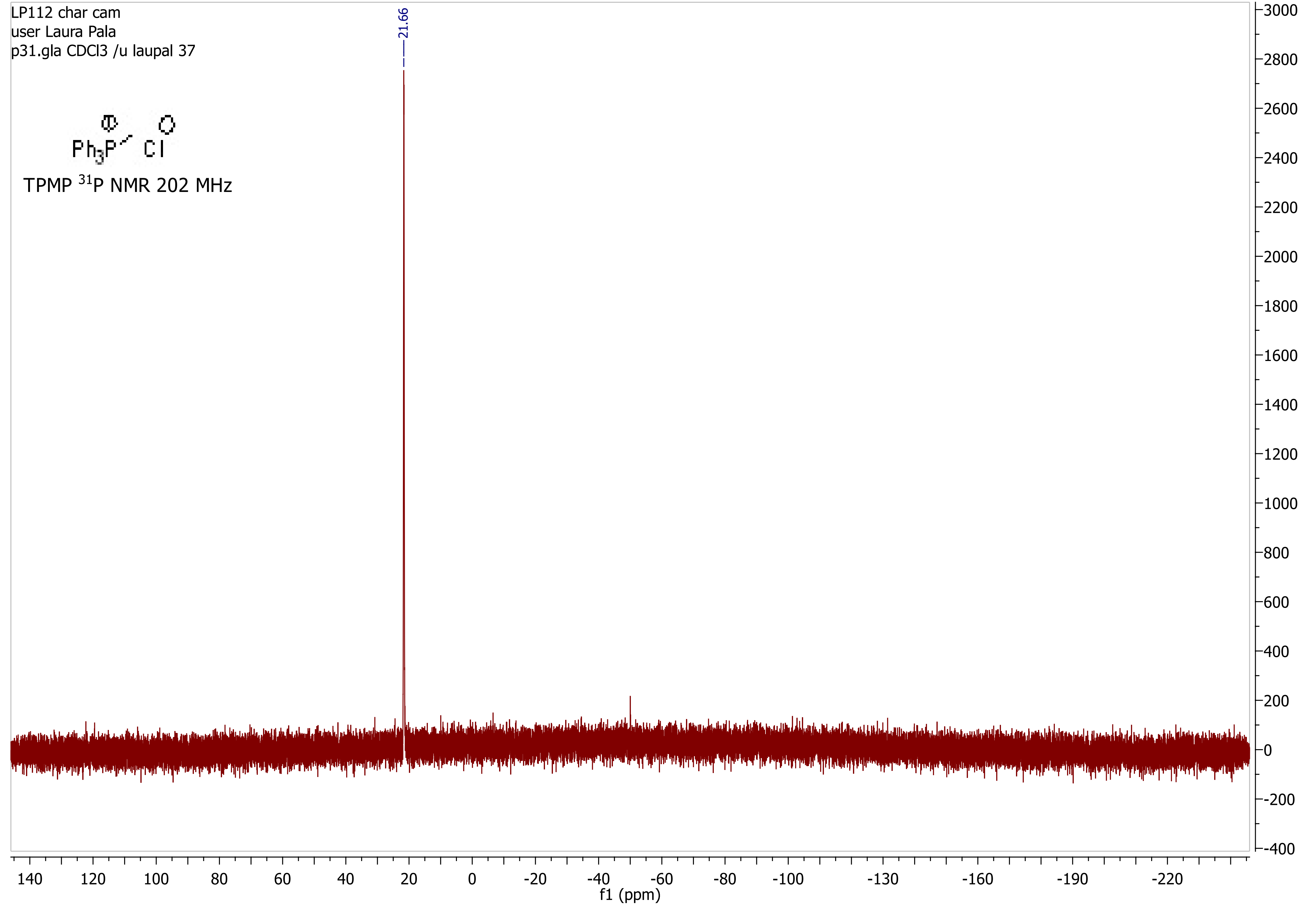

LP112 char cam
user Laura Pala
p31.gla CDCl3 /u laupal 37
TPMP ^{31}P NMR 202 MHz
21.66
f1 (ppm)

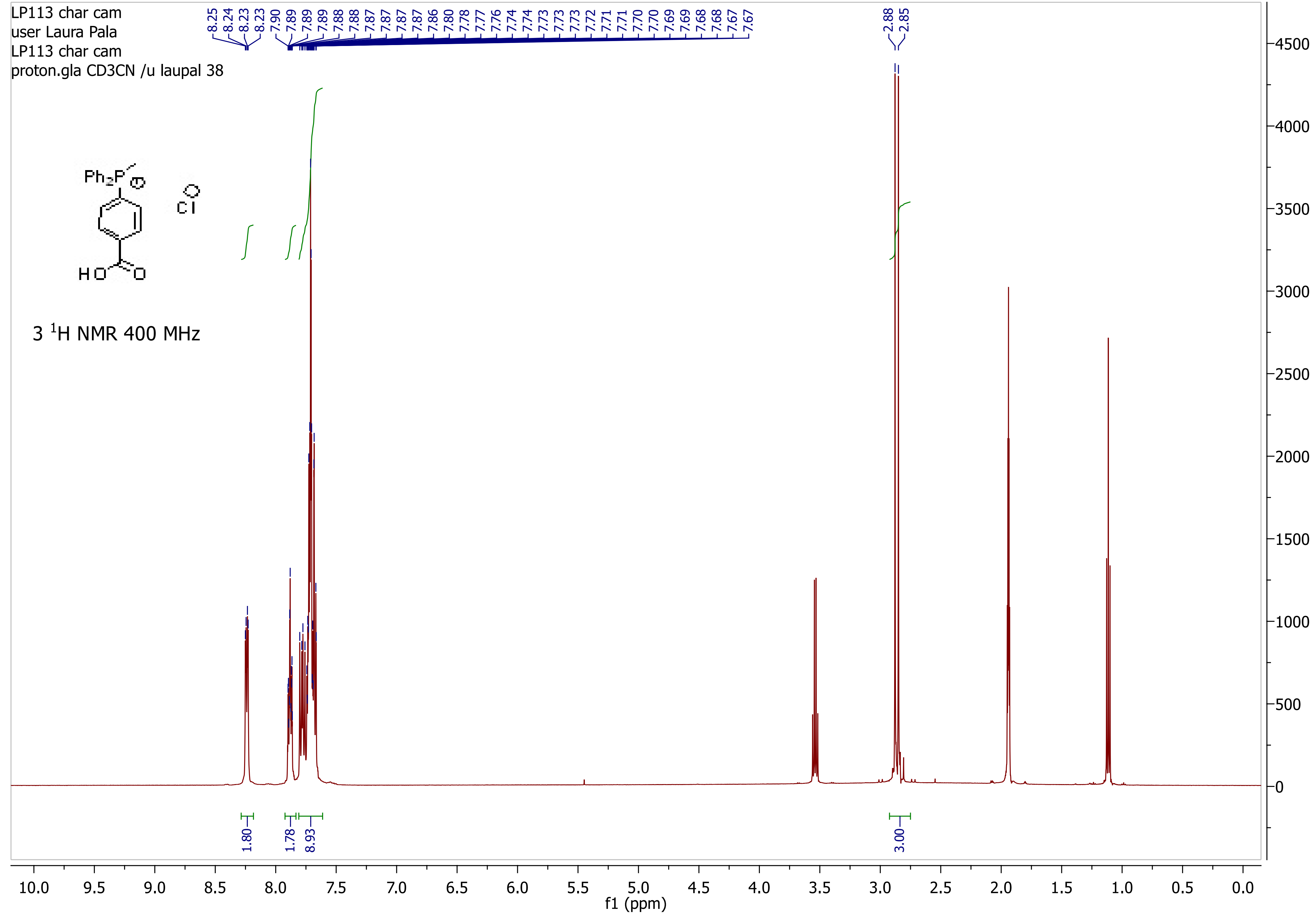
LP113 char cam
user Laura Pala
LP113 char cam
proton.gla CD3CN /u laupal 38
8.25
8.24
8.23
8.23
7.90
7.89
7.89
7.89
7.88
7.88
7.87
7.87
7.87
7.87
7.86
7.80
7.78
7.77
7.76
7.74
7.74
7.73
7.73
7.73
7.72
7.71
7.71
7.70
7.70
7.69
7.69
7.68
7.68
7.67
7.67
2.88
2.85
Ph2P
Cl
HO
O
3 1H NMR 400 MHz
1.80
1.78
8.93
3.00
f1 (ppm)

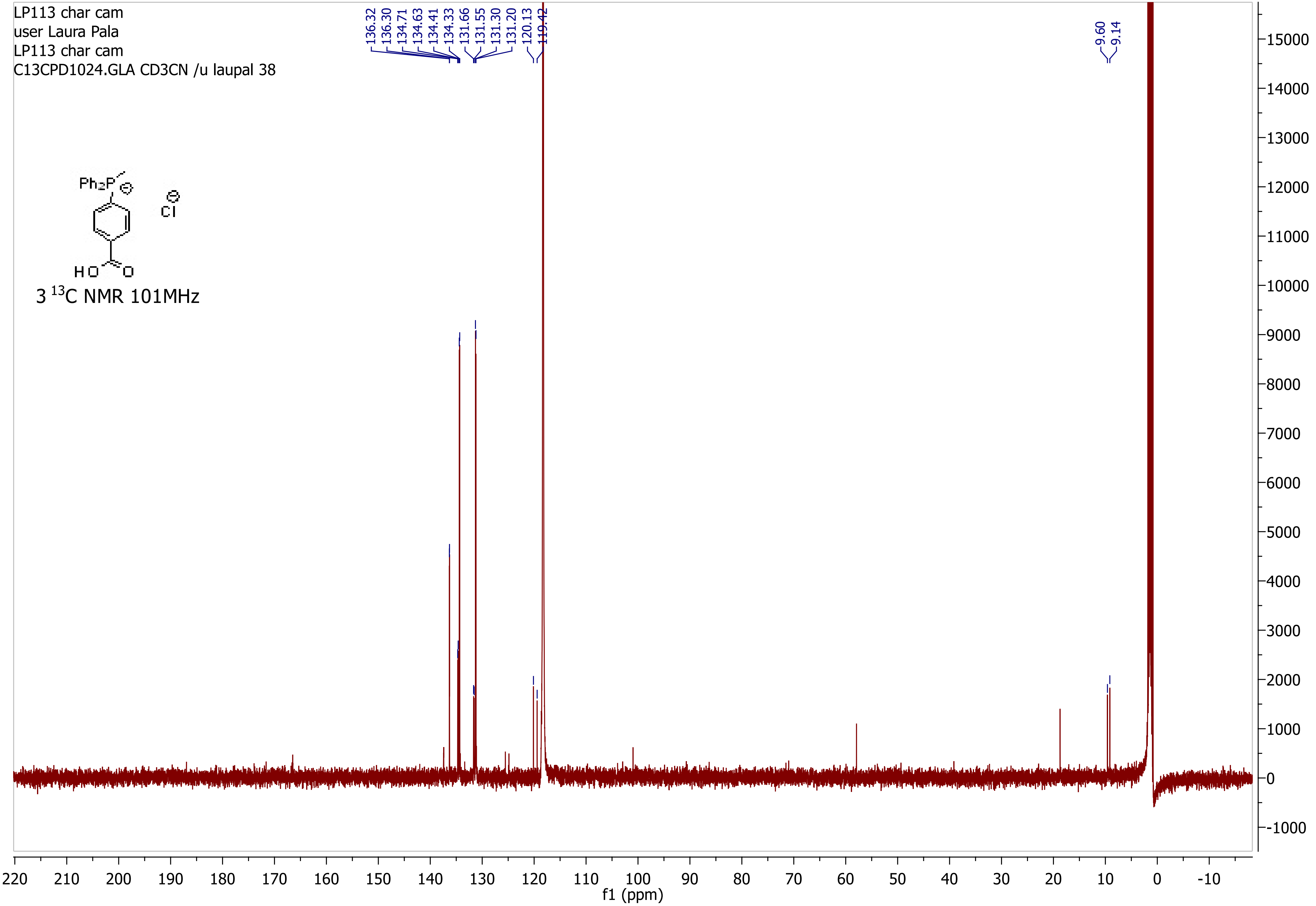
LP113 char cam
user Laura Pala
LP113 char cam
C13CPD1024.GLA CD3CN /u laupal 38
136.32
136.30
134.71
134.63
134.41
134.33
131.66
131.55
131.30
131.20
120.13
119.42
9.60
9.14
Ph2P
Cl
HO
O
3 13C NMR 101MHz
f1 (ppm)

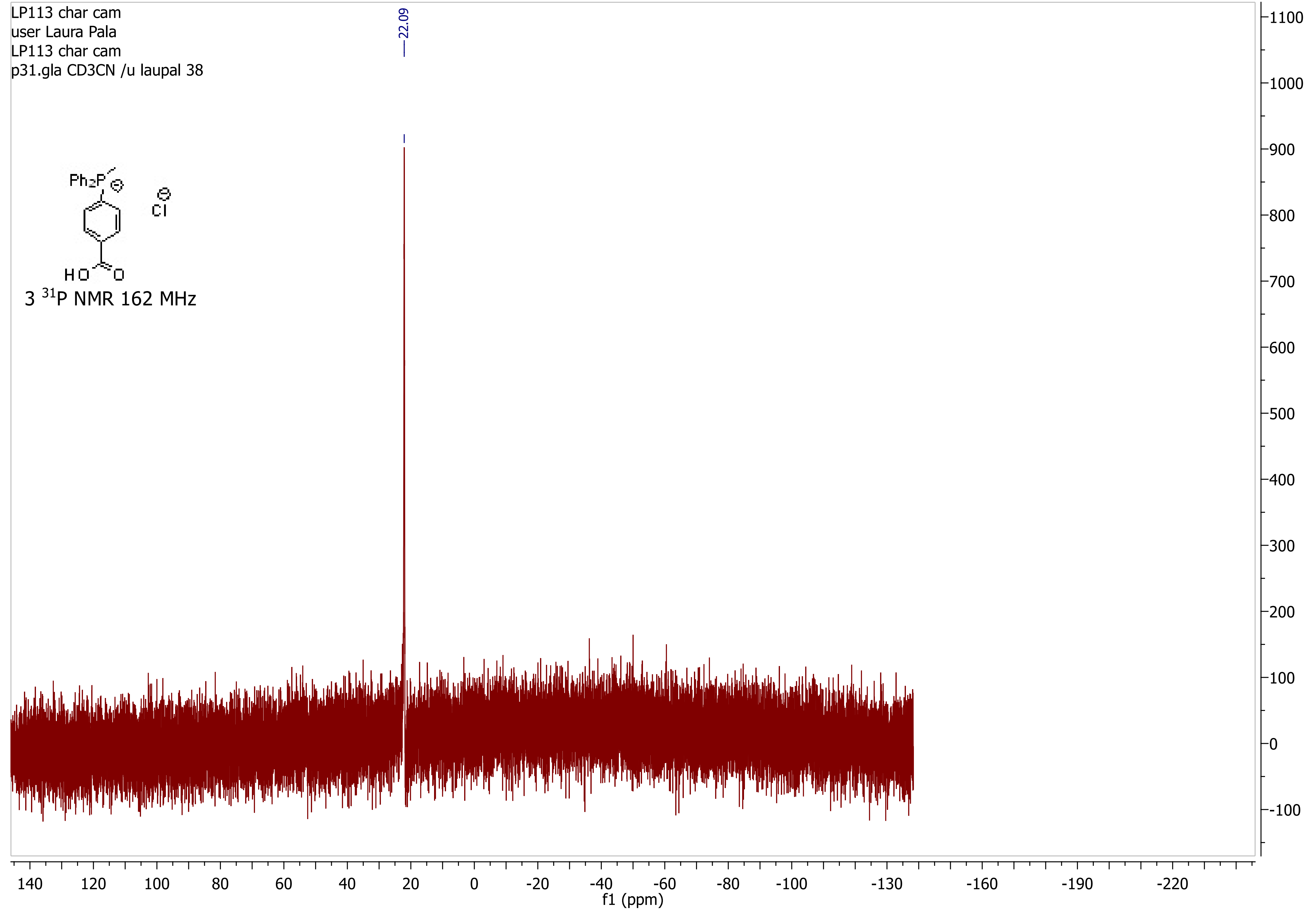
LP113 char cam
user Laura Pala
LP113 char cam
p31.gla CD3CN /u laupal 38
22.09
Ph2P
Cl
HO
O
3 31P NMR 162 MHz
1100
1000
900
800
700
600
500
400
300
200
100
0
-100
140
120
100
80
60
40
20
0
-20
-40
-60
-80
-100
-130
-160
-190
-220
f1 (ppm)

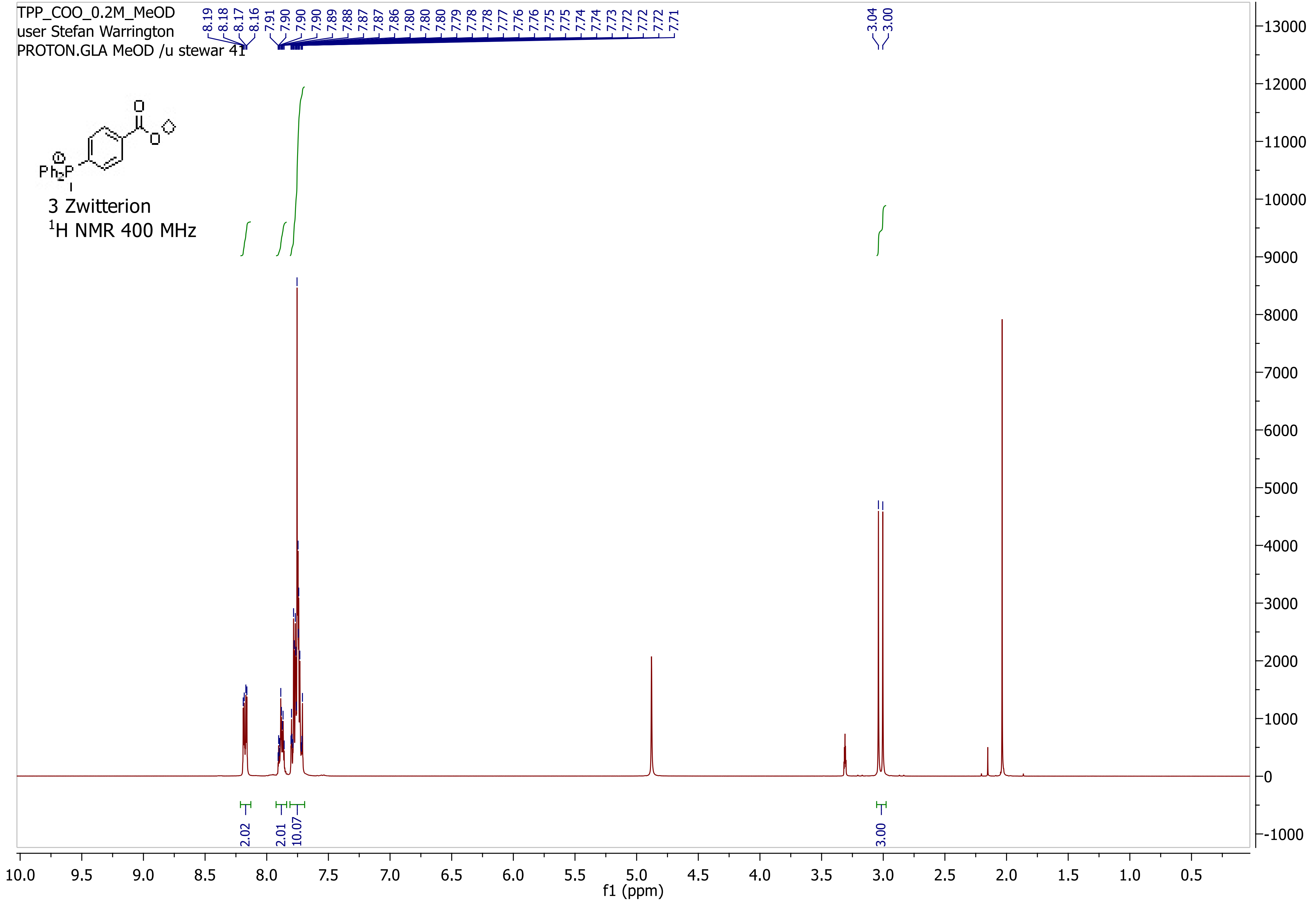

TPP_COO_0.2M_MeOD
user Stefan Warrington
PROTON.GLA MeOD /u stewar 41
8.19 8.18 8.17 8.16 7.91 7.90 7.90 7.90 7.89 7.88 7.87 7.87 7.86 7.80 7.80 7.80 7.79 7.78 7.78 7.77 7.76 7.76 7.75 7.75 7.74 7.74 7.73 7.72 7.72 7.72 7.71
3.04 3.00
Ph2P
3 Zwitterion
1H NMR 400 MHz
2.02 2.01 10.07 3.00
f1 (ppm)

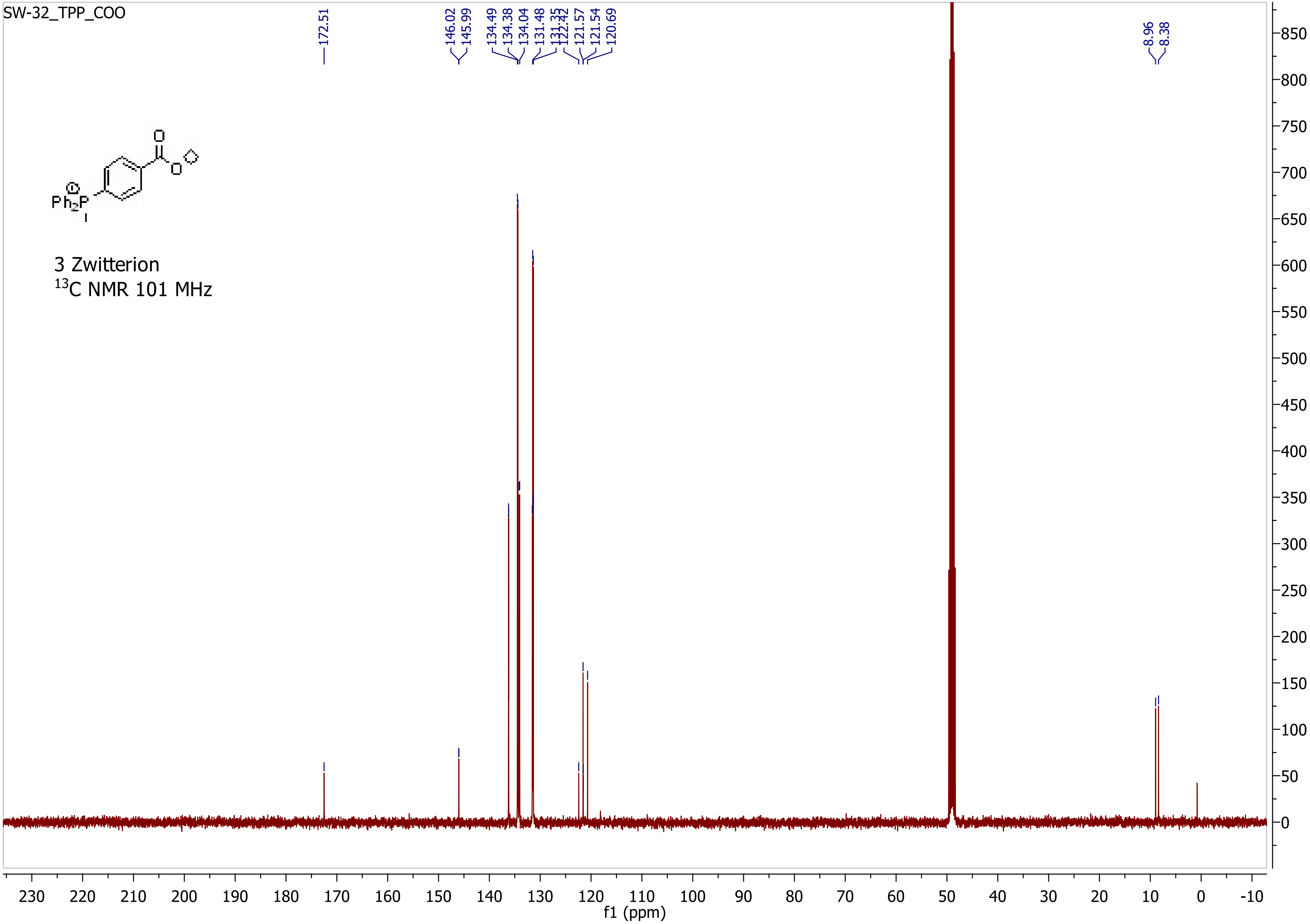

SW-32_TPP_COO
172.51
146.02
145.99
134.49
134.38
134.04
131.48
131.35
122.42
121.57
121.54
120.69
8.96
8.38
Ph2P
3 Zwitterion
13C NMR 101 MHz
850
800
750
700
650
600
550
500
450
400
350
300
250
200
150
100
50
0
230
220
210
200
190
180
170
160
150
140
130
120
110
100
90
80
70
60
50
40
30
20
10
0
-10
f1 (ppm)

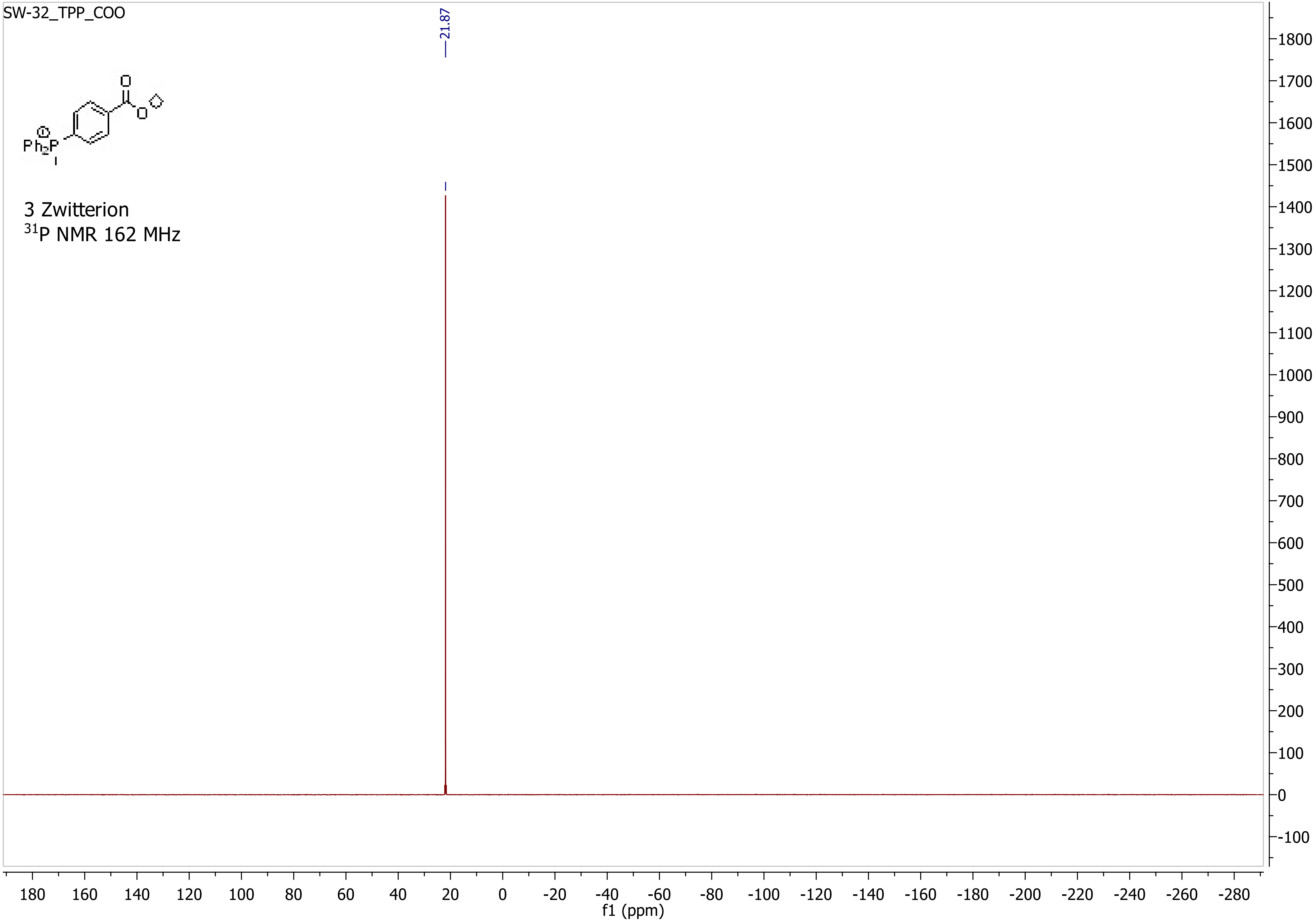
SW-32_TPP_COO
3 Zwitterion
^{31}P NMR 162 MHz
21.87
f1 (ppm)

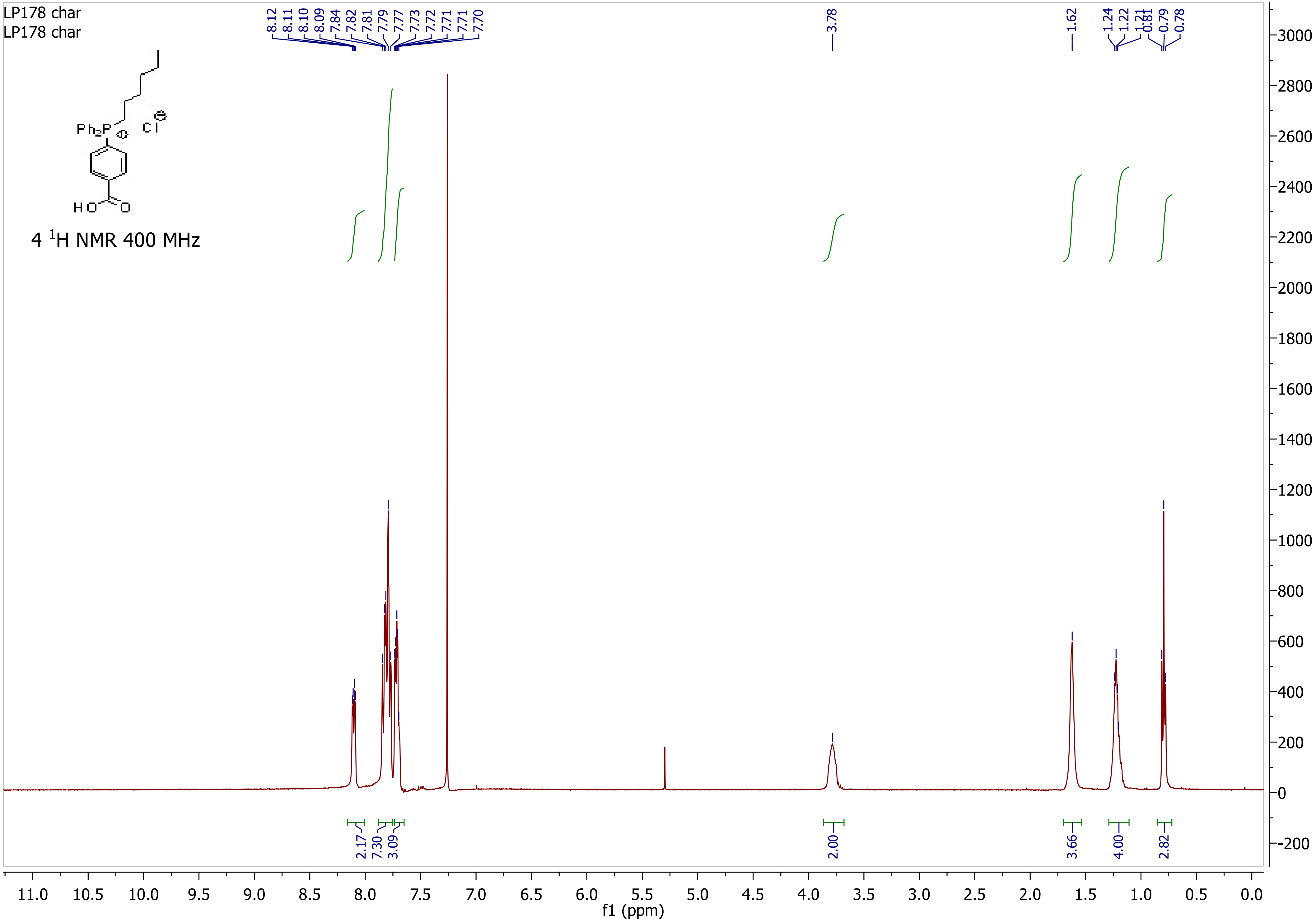

LP178 char
LP178 char
8.12 8.11 8.10 8.09 7.84 7.82 7.81 7.79 7.77 7.73 7.72 7.71 7.71 7.70
3.78
1.62
1.24 1.22 1.21 0.81 0.79 0.78
Ph2P
Cl
HO
O
4 1H NMR 400 MHz
2.17 7.30 3.09
2.00
3.66 4.00 2.82
f1 (ppm)

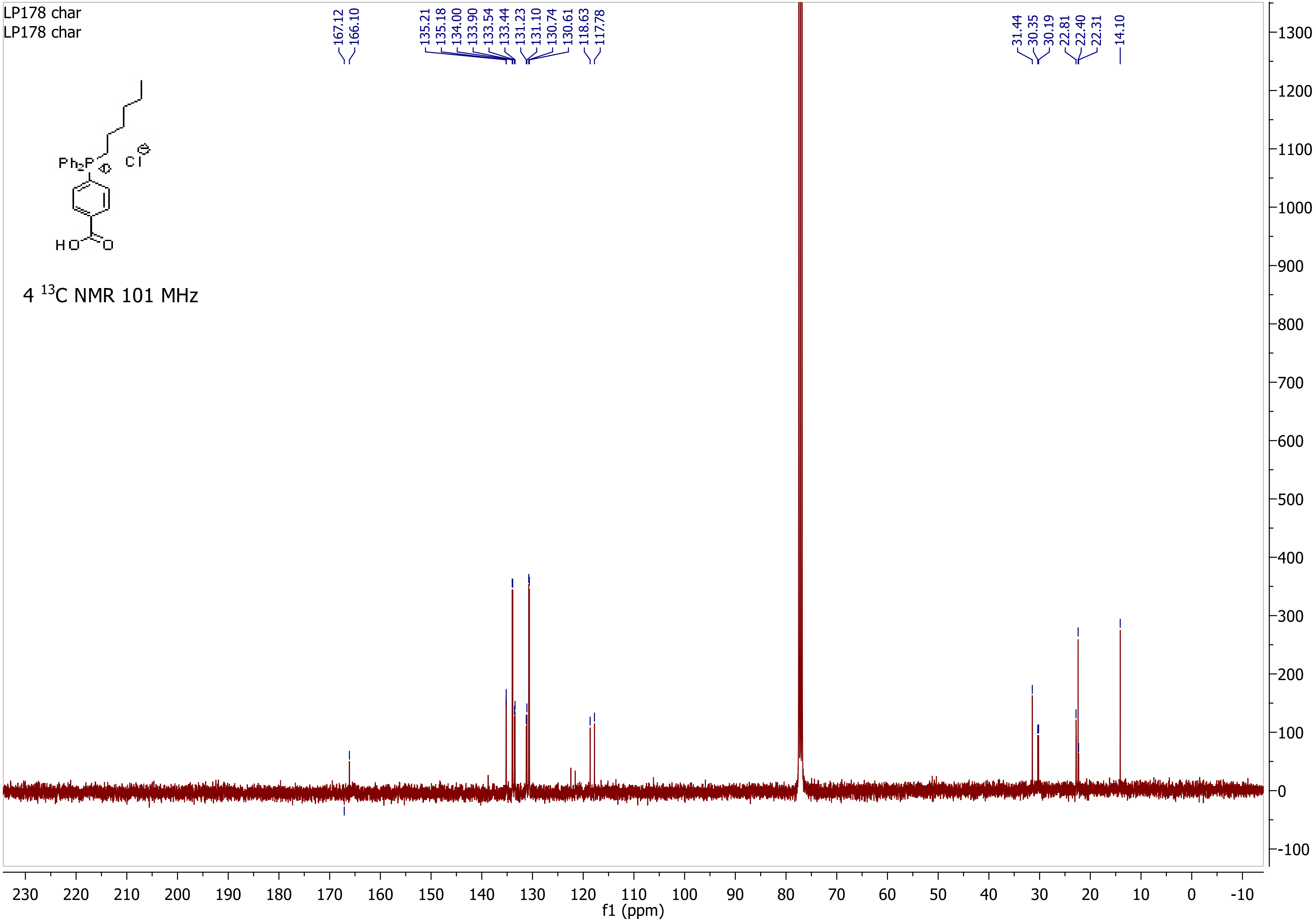
LP178 char
LP178 char
4 ^{13}C NMR 101 MHz
167.12
166.10
135.21
135.18
134.00
133.90
133.54
133.44
131.23
131.10
130.74
130.61
118.63
117.78
31.44
30.35
30.19
22.81
22.40
22.31
14.10
Ph2P
Cl
HO
O
f1 (ppm)
230 220 210 200 190 180 170 160 150 140 130 120 110 100 90 80 70 60 50 40 30 20 10 0 -10
1300 1200 1100 1000 900 800 700 600 500 400 300 200 100 0 -100

LP178 char
LP178 char

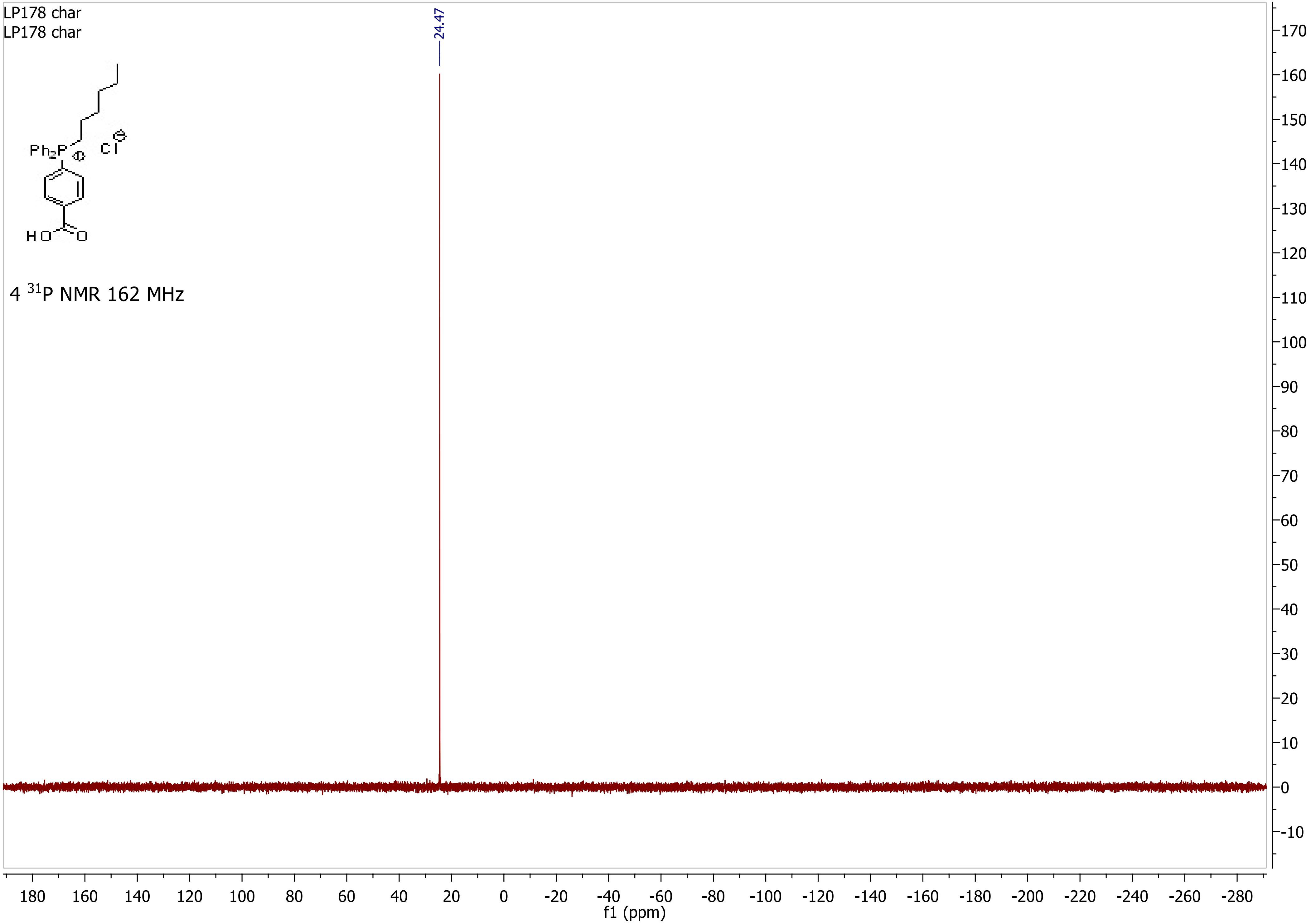

4 ^{31}P NMR 162 MHz

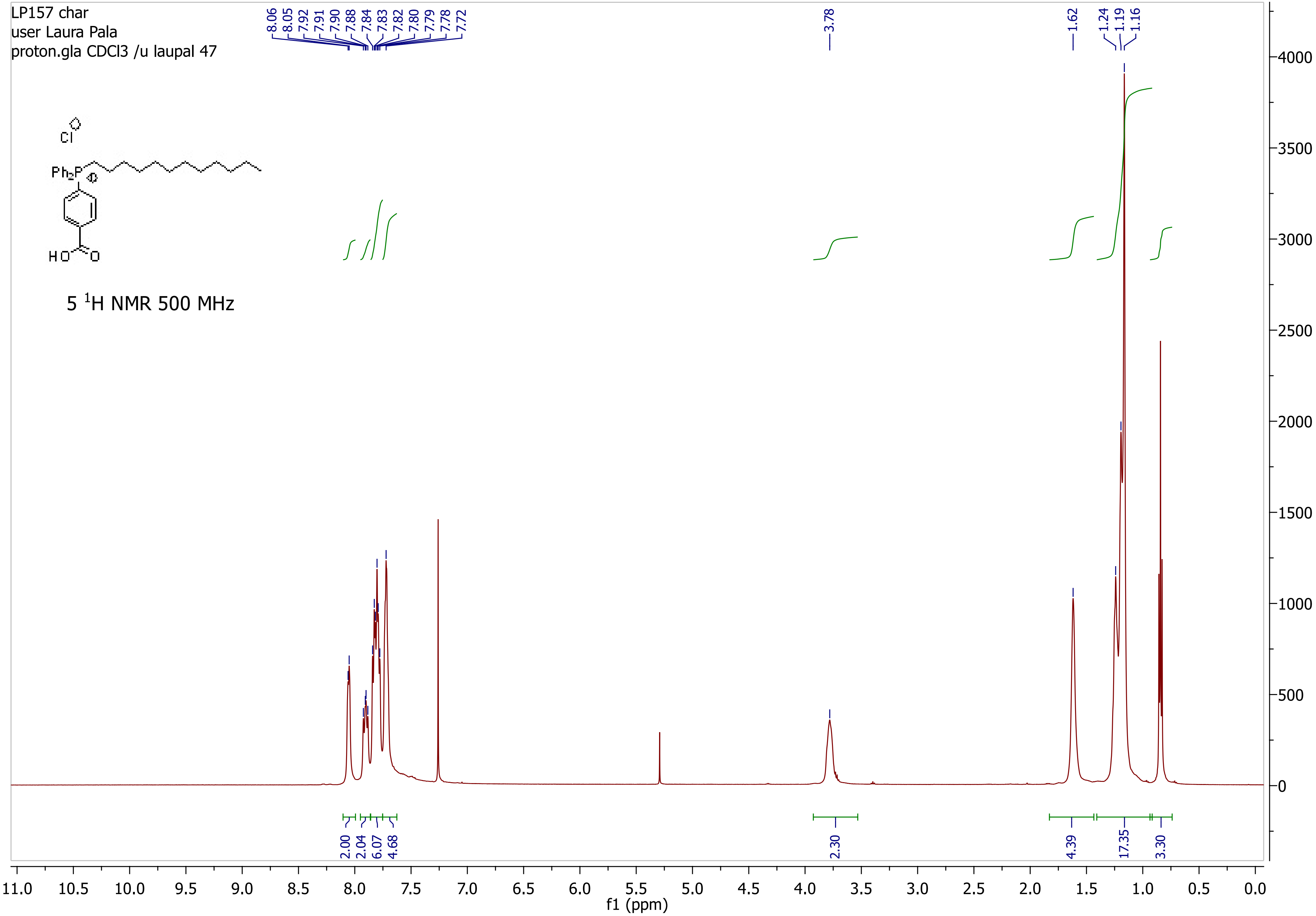

LP157 char
user Laura Pala
proton.gla CDCl3 /u laupal 47
8.06 8.05 7.92 7.91 7.90 7.88 7.84 7.83 7.82 7.80 7.79 7.78 7.72
3.78
1.62
1.24 1.19 1.16
Cl
Ph2P
HO
O
5 1H NMR 500 MHz
2.00 2.04 6.07 4.68
2.30
4.39 17.35 3.30
f1 (ppm)

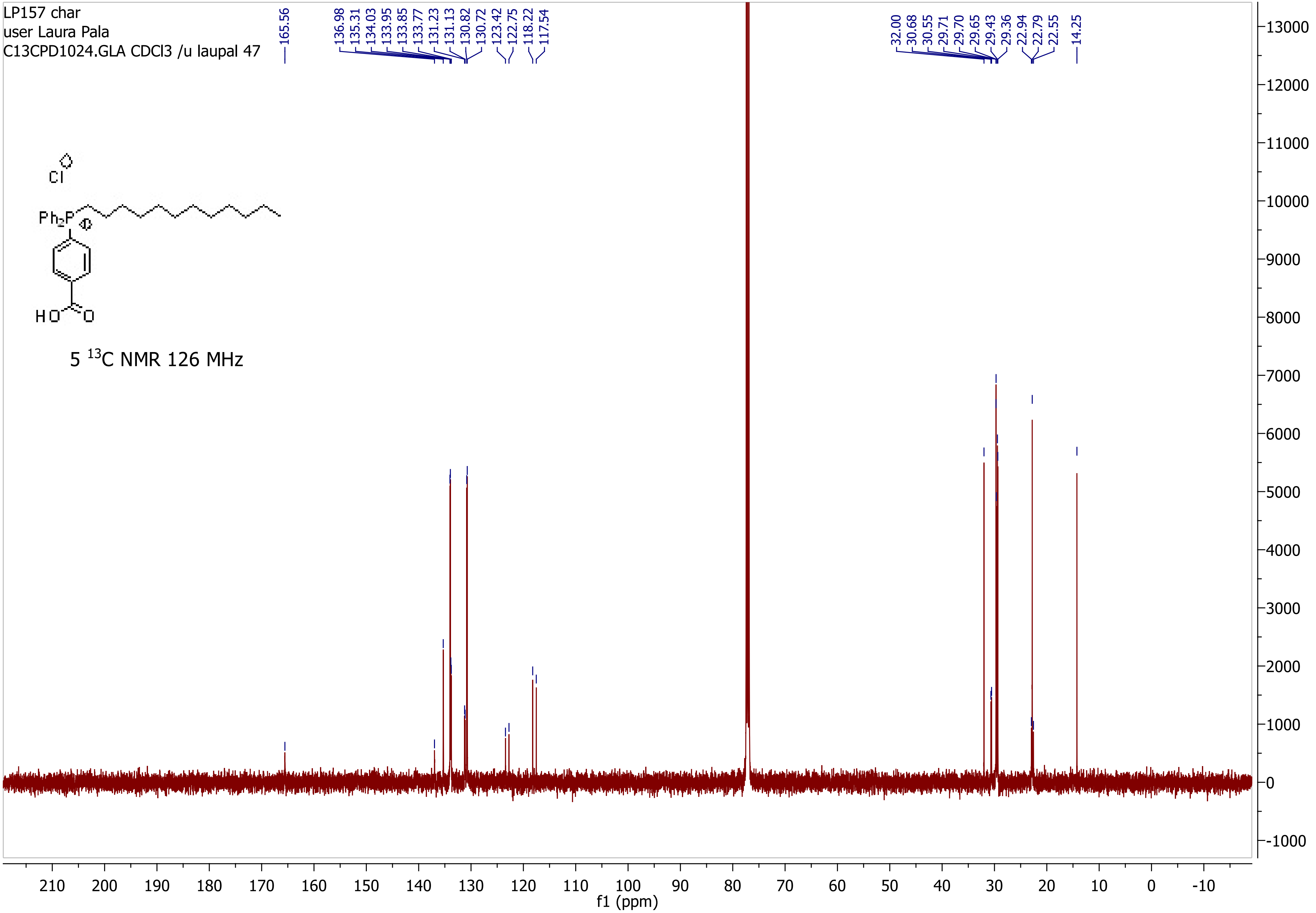
LP157 char
user Laura Pala
C13CPD1024.GLA CDCl3 /u laupal 47
165.56
136.98
135.31
134.03
133.95
133.85
133.77
131.23
131.13
130.82
130.72
123.42
122.75
118.22
117.54
32.00
30.68
30.55
29.71
29.70
29.65
29.43
29.36
22.94
22.79
22.55
14.25
Cl
Ph2P
HO
O
5 13C NMR 126 MHz
13000
12000
11000
10000
9000
8000
7000
6000
5000
4000
3000
2000
1000
0
-1000
210
200
190
180
170
160
150
140
130
120
110
100
90
80
70
60
50
40
30
20
10
0
-10
f1 (ppm)

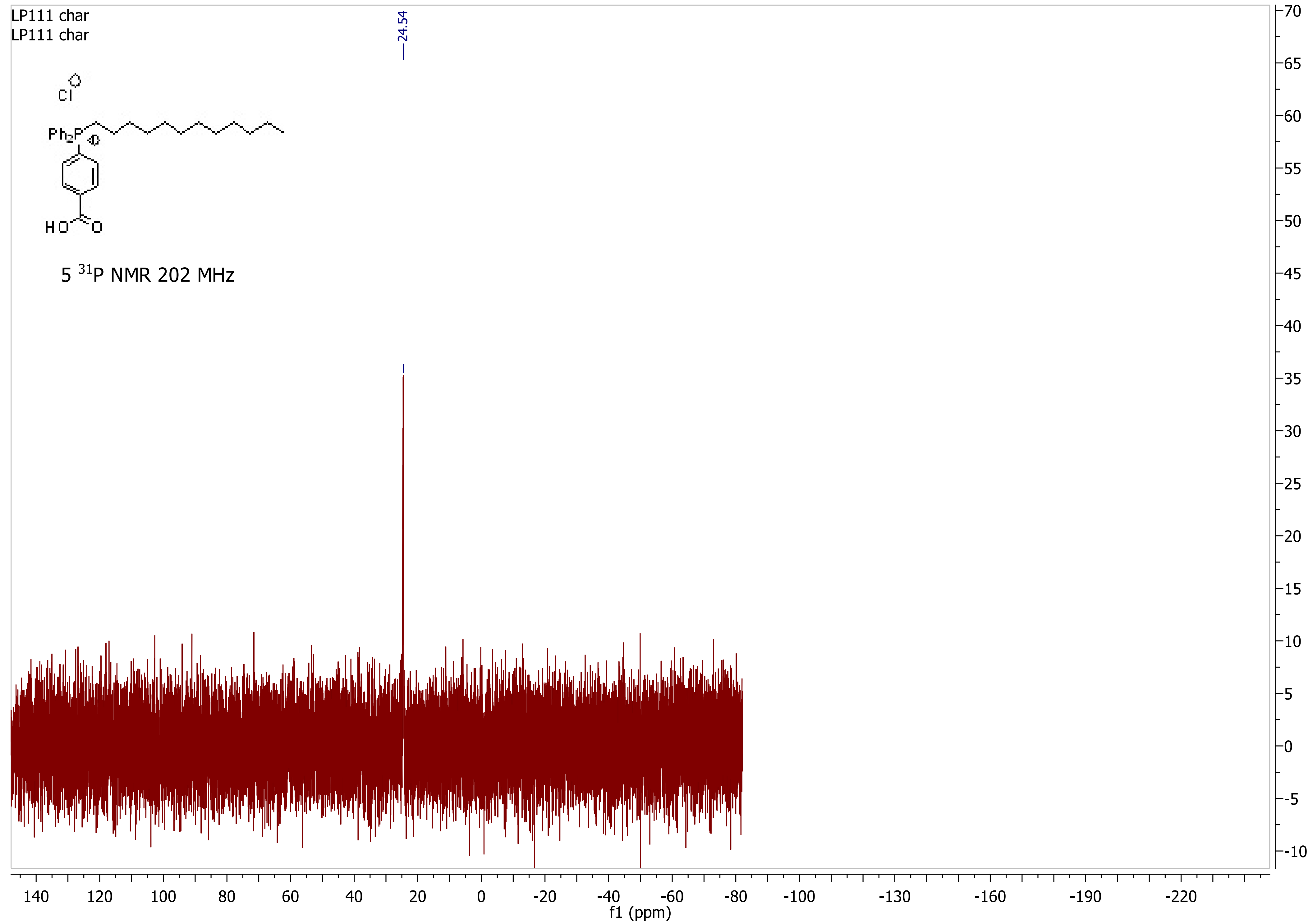
LP111 char
LP111 char
5 31P NMR 202 MHz
24.54
f1 (ppm)

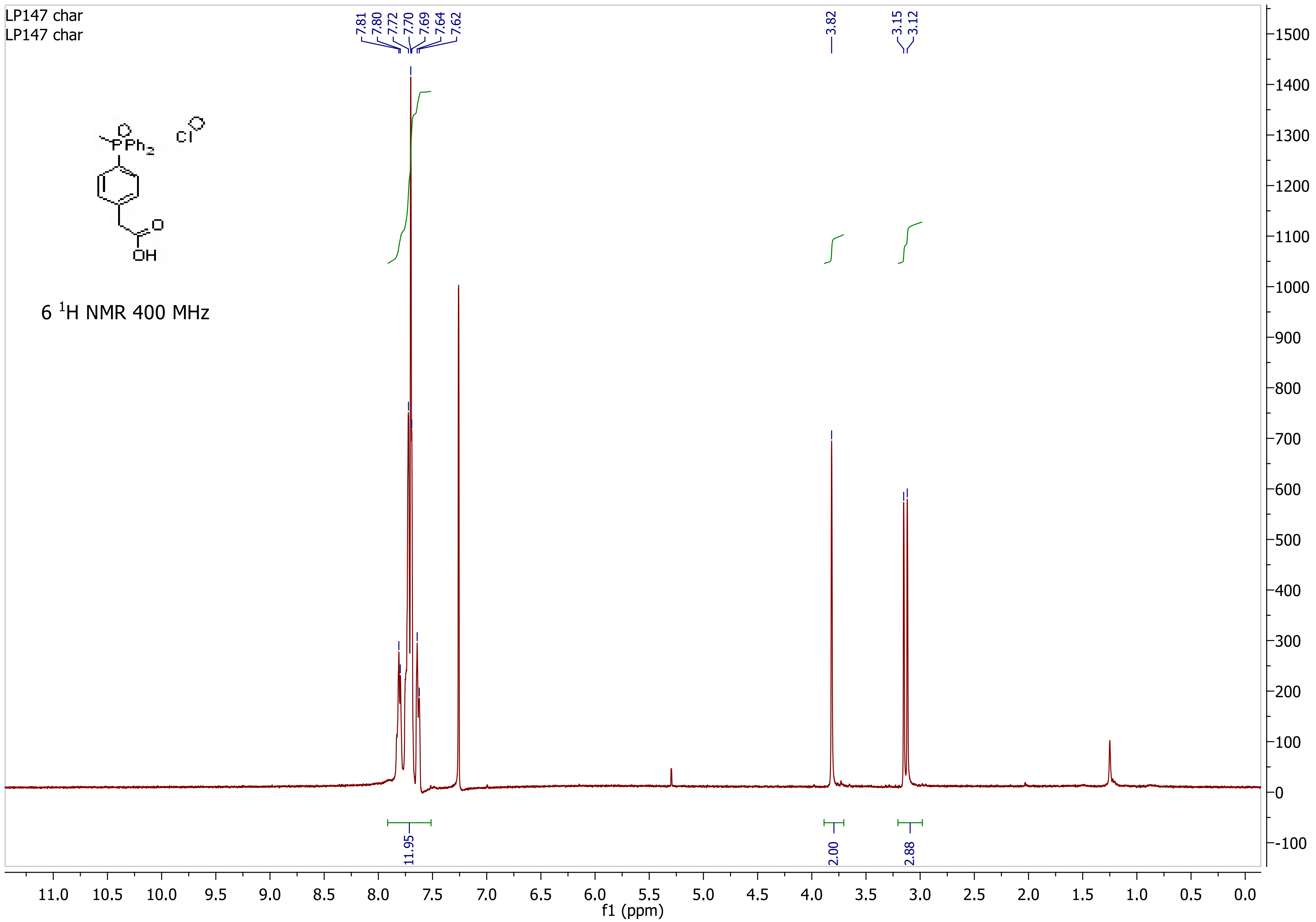

LP147 char
LP147 char
7.81
7.80
7.72
7.70
7.69
7.64
7.62
3.82
3.15
3.12
6 1H NMR 400 MHz
11.95
2.00
2.88
f1 (ppm)

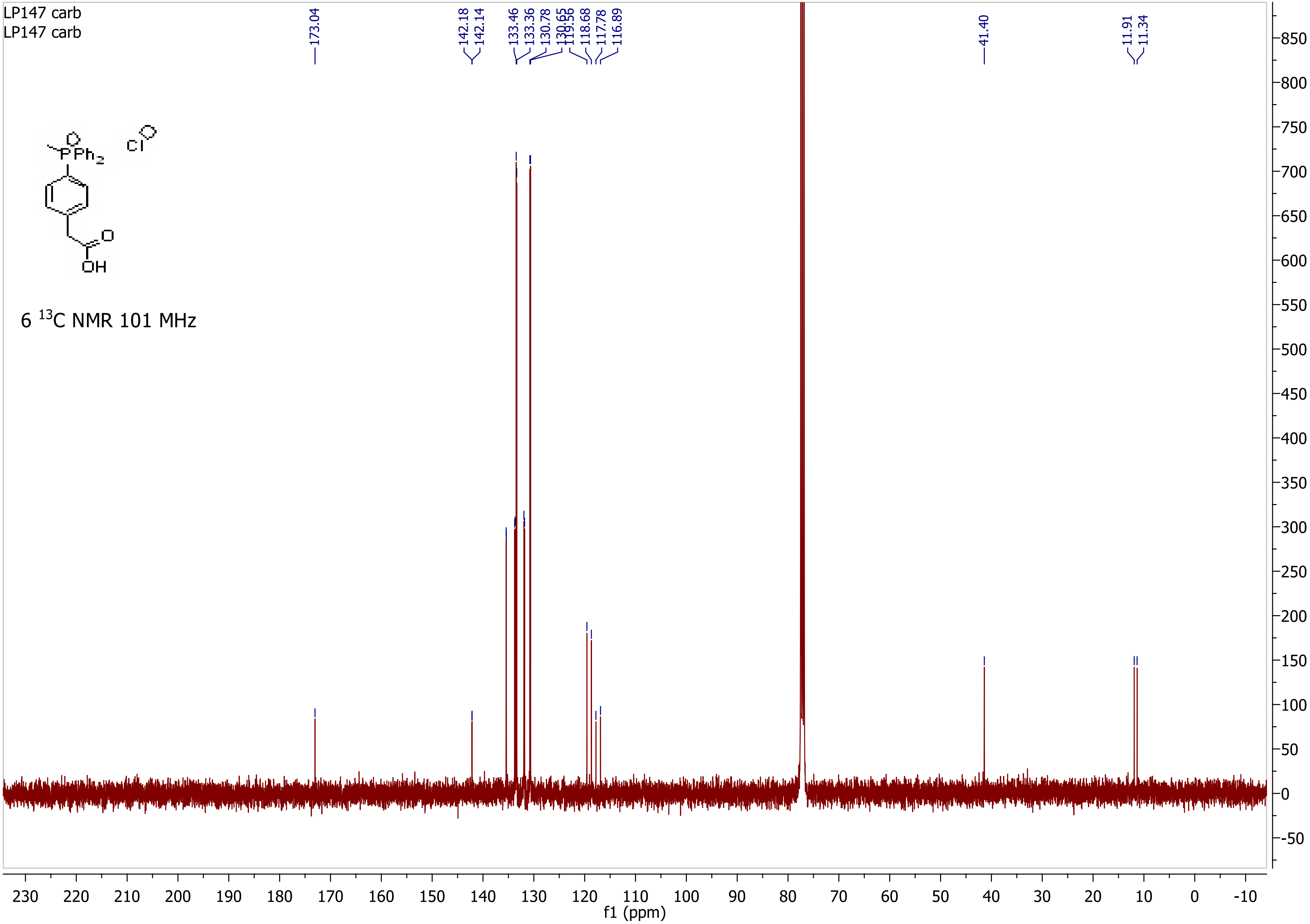

LP147 carb
LP147 carb
173.04
142.18
142.14
133.46
133.36
130.78
130.65
119.56
118.68
117.78
116.89
41.40
11.91
11.34
6 ^{13}C NMR 101 MHz
f1 (ppm)

LP147 char
LP147 char

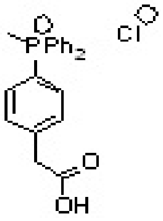

6 ^{31}P NMR 162 MHz

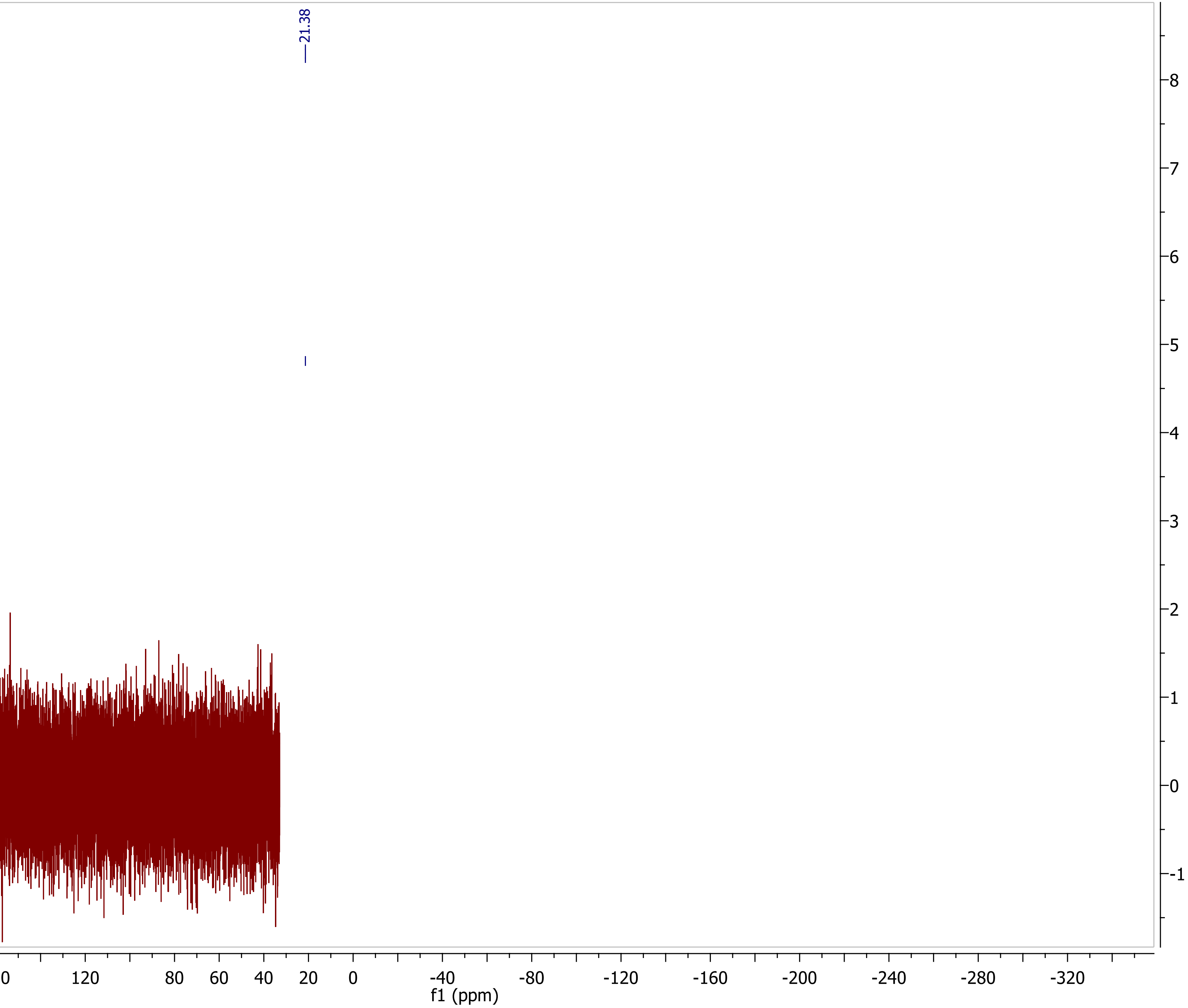

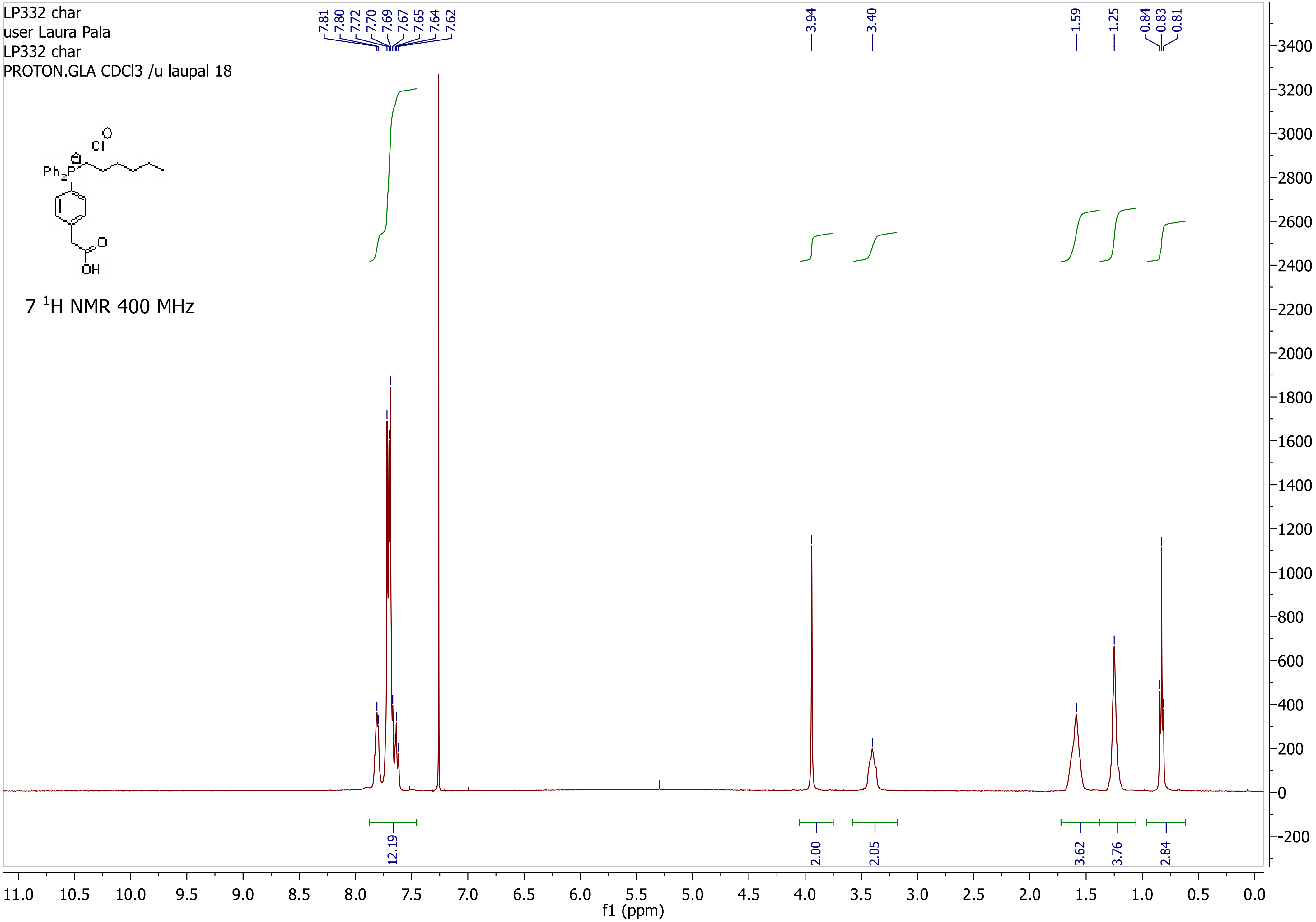

LP332 char
user Laura Pala
LP332 char
PROTON.GLA CDCl3 /u laupal 18
7 1H NMR 400 MHz
7.81
7.80
7.72
7.70
7.69
7.67
7.65
7.64
7.62
3.94
3.40
1.59
1.25
0.84
0.83
0.81
12.19
2.00
2.05
3.62
3.76
2.84
f1 (ppm)
Ph2P
Cl
O
OH

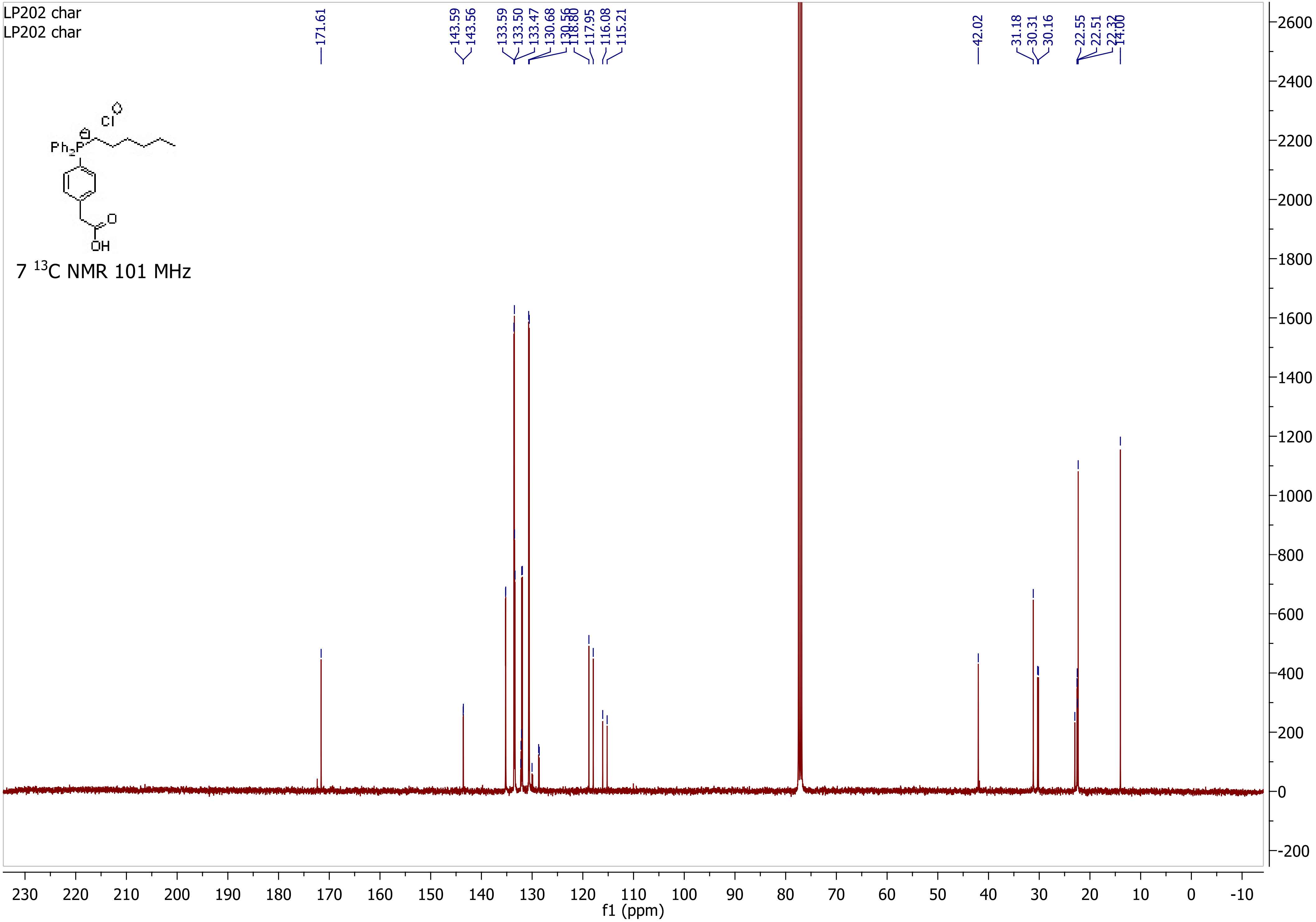
LP202 char
LP202 char
171.61
143.59
143.56
133.59
133.50
133.47
130.68
130.56
118.80
117.95
116.08
115.21
42.02
31.18
30.31
30.16
22.55
22.51
22.32
14.00
Cl
O
Ph2P
O
OH
7 13C NMR 101 MHz
f1 (ppm)

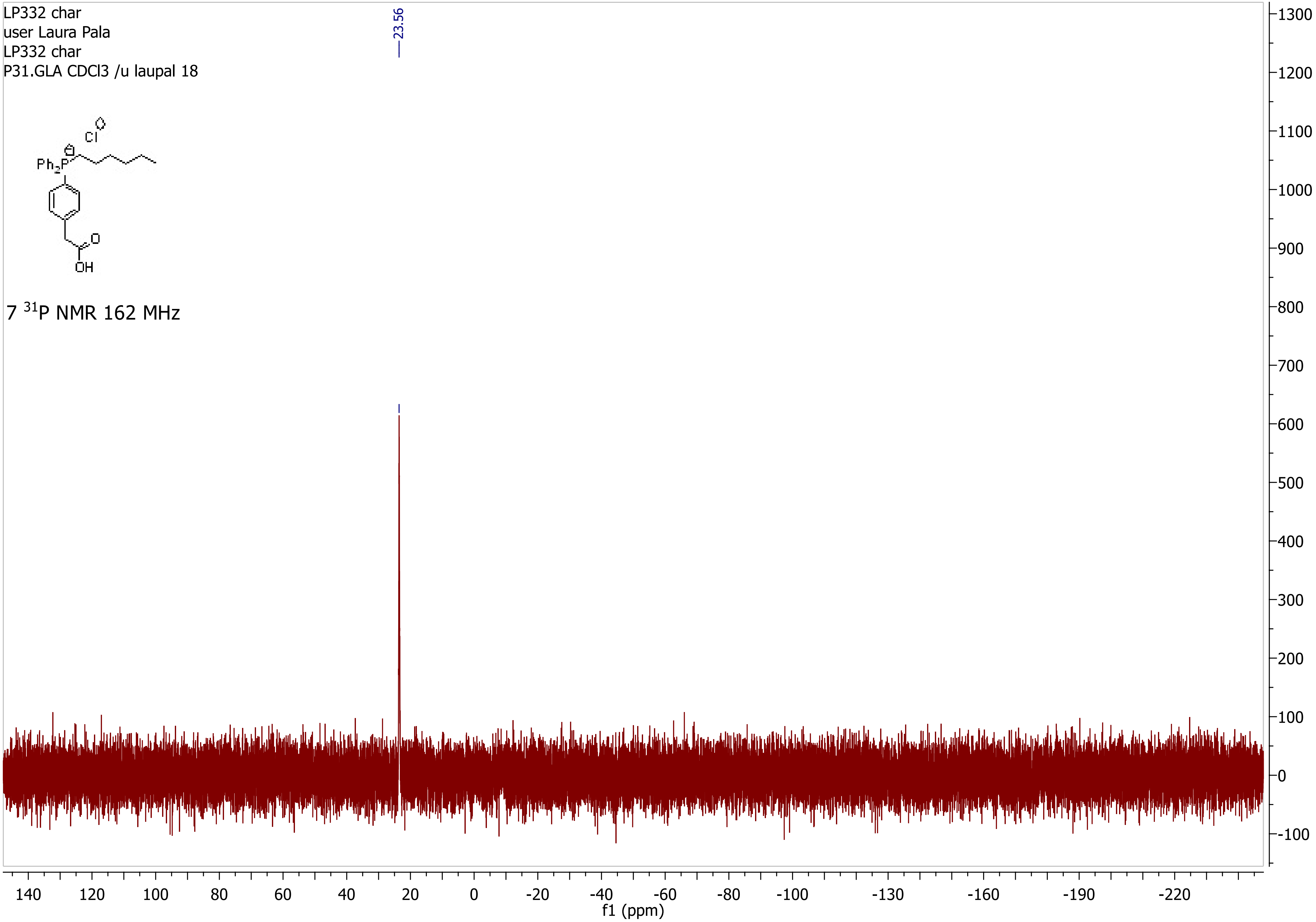
LP332 char
user Laura Pala
LP332 char
P31.GLA CDCl3 /u laupal 18
Cl
Ph2P
OH
O
7 31P NMR 162 MHz
23.56
f1 (ppm)

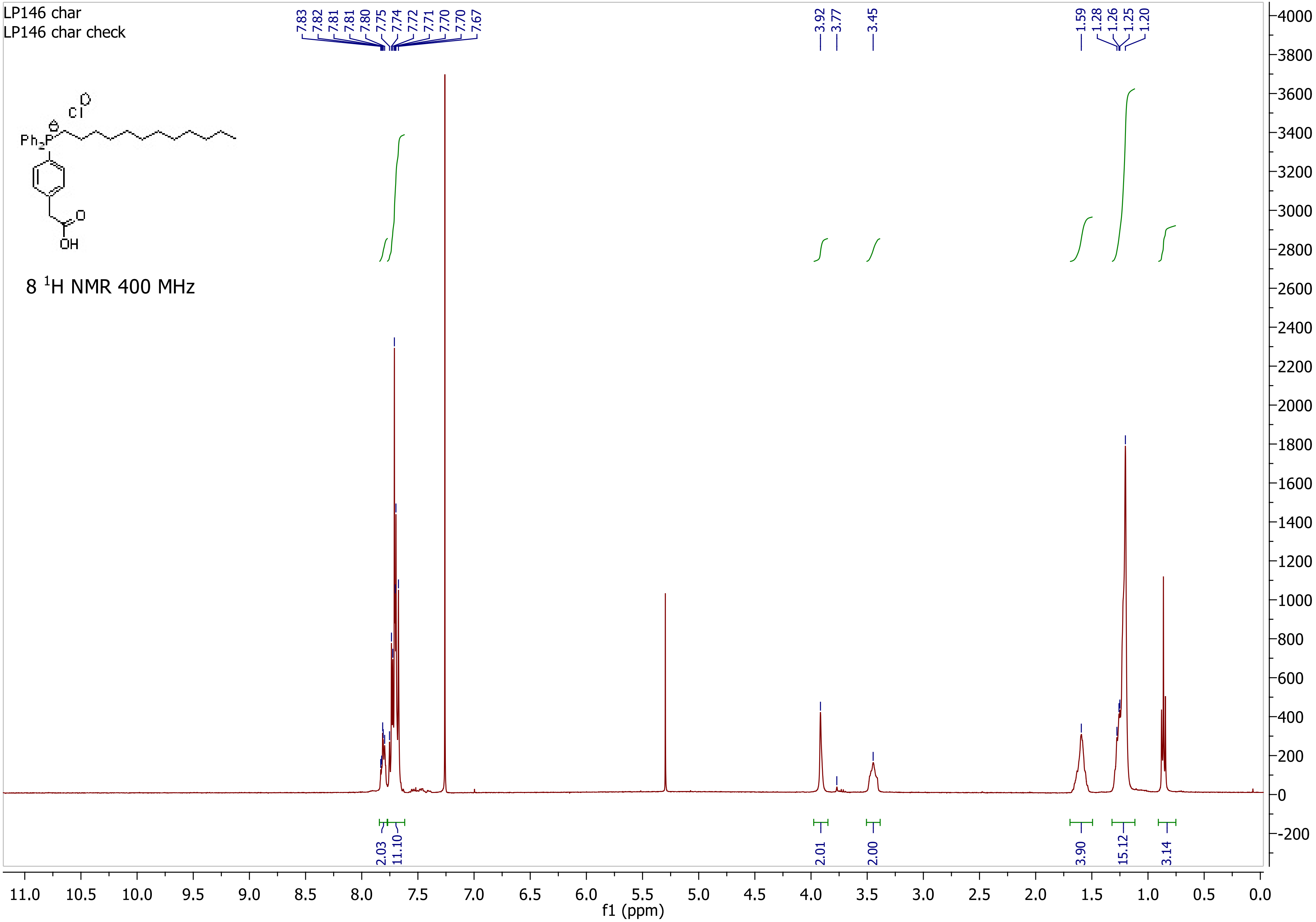
LP146 char
LP146 char check
8 1H NMR 400 MHz
7.83 7.82 7.81 7.81 7.80 7.75 7.74 7.72 7.71 7.70 7.70 7.67
3.92 3.77 3.45
1.59 1.28 1.26 1.25 1.20
2.03 11.10 2.01 2.00 3.90 15.12 3.14
Ph2P
OH
f1 (ppm)

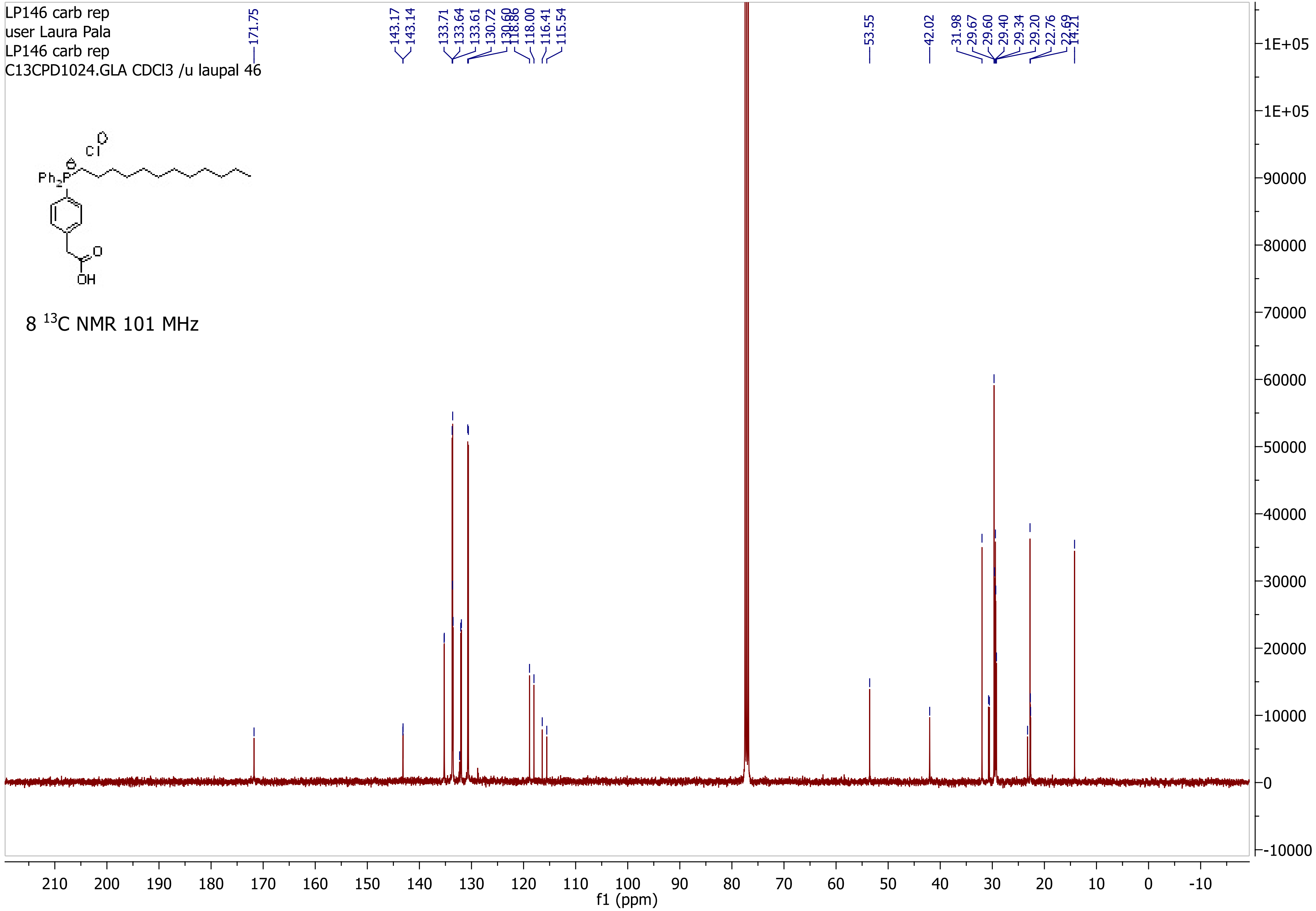

LP146 carb rep
user Laura Pala
LP146 carb rep
C13CPD1024.GLA CDCl3 /u laupal 46
8 13C NMR 101 MHz
171.75
143.17
143.14
133.71
133.64
133.61
130.72
118.00
116.41
115.54
53.55
42.02
31.98
29.67
29.60
29.40
29.34
29.20
22.76
f1 (ppm)

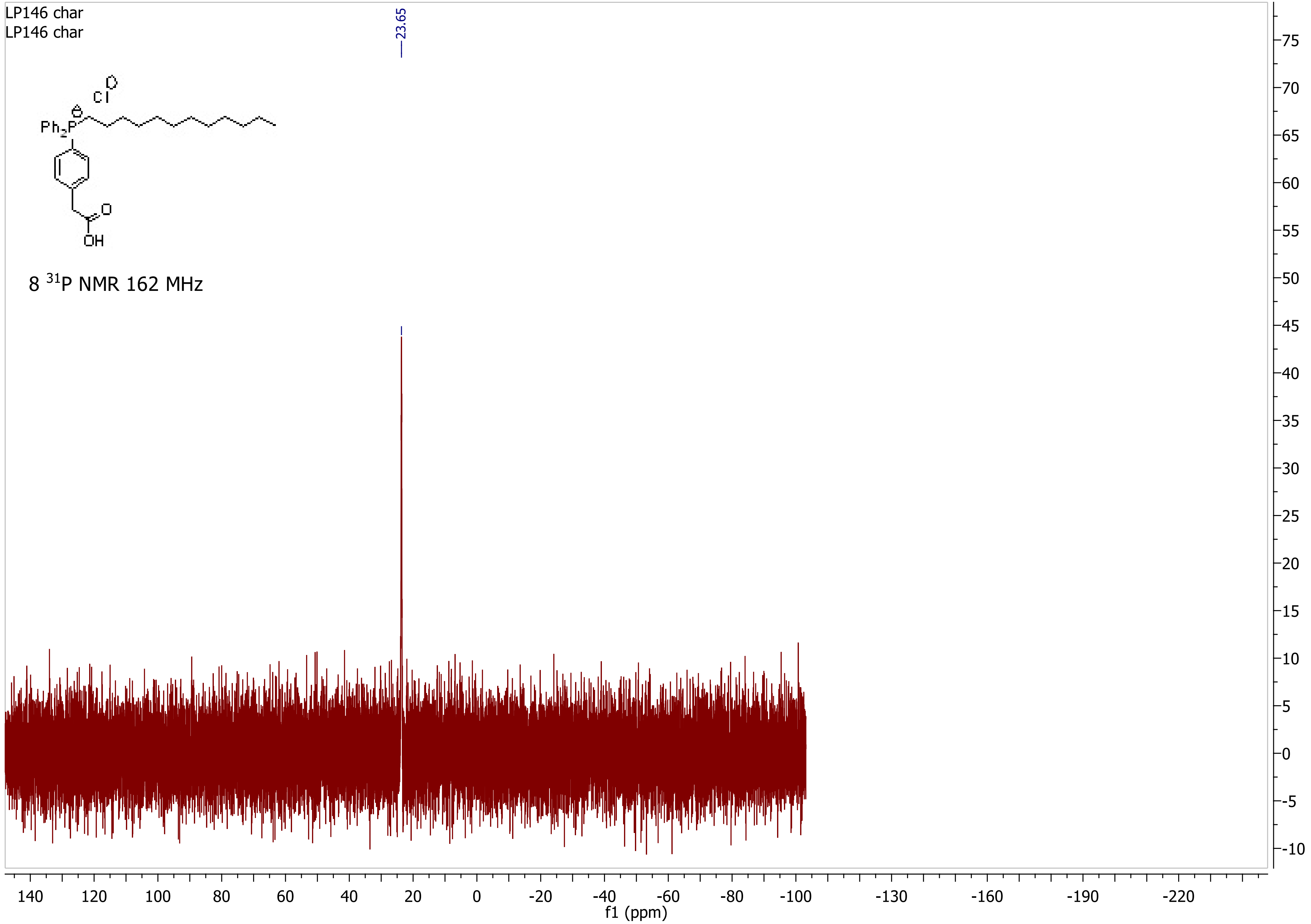
LP146 char
LP146 char
Ph2P
Cl
O
OH
8 31P NMR 162 MHz
23.65
f1 (ppm)
140 120 100 80 60 40 20 0 -20 -40 -60 -80 -100 -130 -160 -190 -220
75 70 65 60 55 50 45 40 35 30 25 20 15 10 5 0 -5 -10

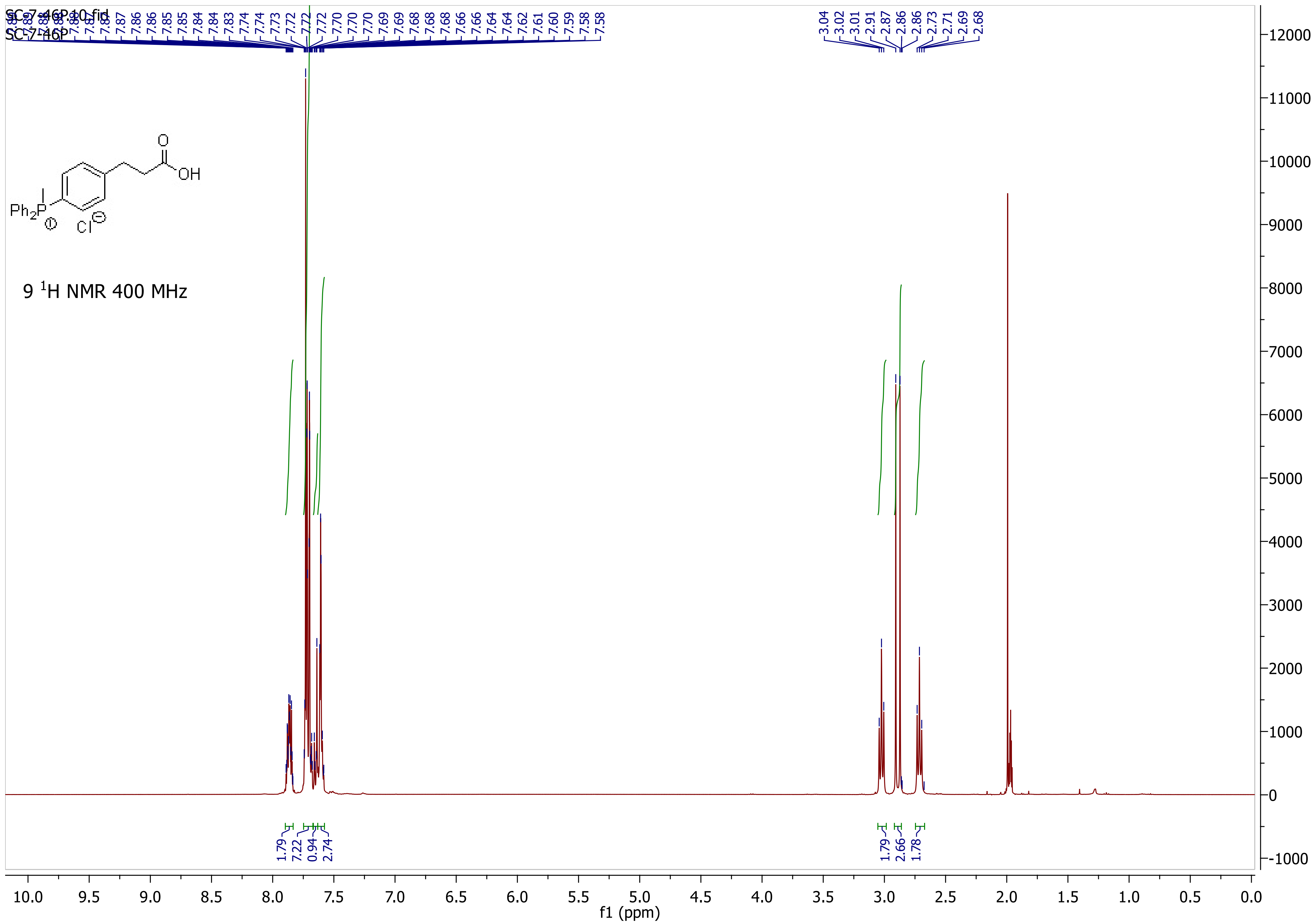
SC-7-46P.10.fid
SC-7-46P
9 1H NMR 400 MHz
OH
O
Ph2P
Cl
7.87
7.86
7.86
7.85
7.85
7.84
7.84
7.83
7.74
7.74
7.73
7.72
7.72
7.72
7.70
7.70
7.70
7.69
7.69
7.68
7.68
7.68
7.66
7.66
7.64
7.64
7.62
7.61
7.60
7.59
7.58
7.58
3.04
3.02
3.01
2.91
2.87
2.86
2.86
2.73
2.71
2.69
2.68
1.79
7.22
0.94
2.74
1.79
2.66
1.78
f1 (ppm)

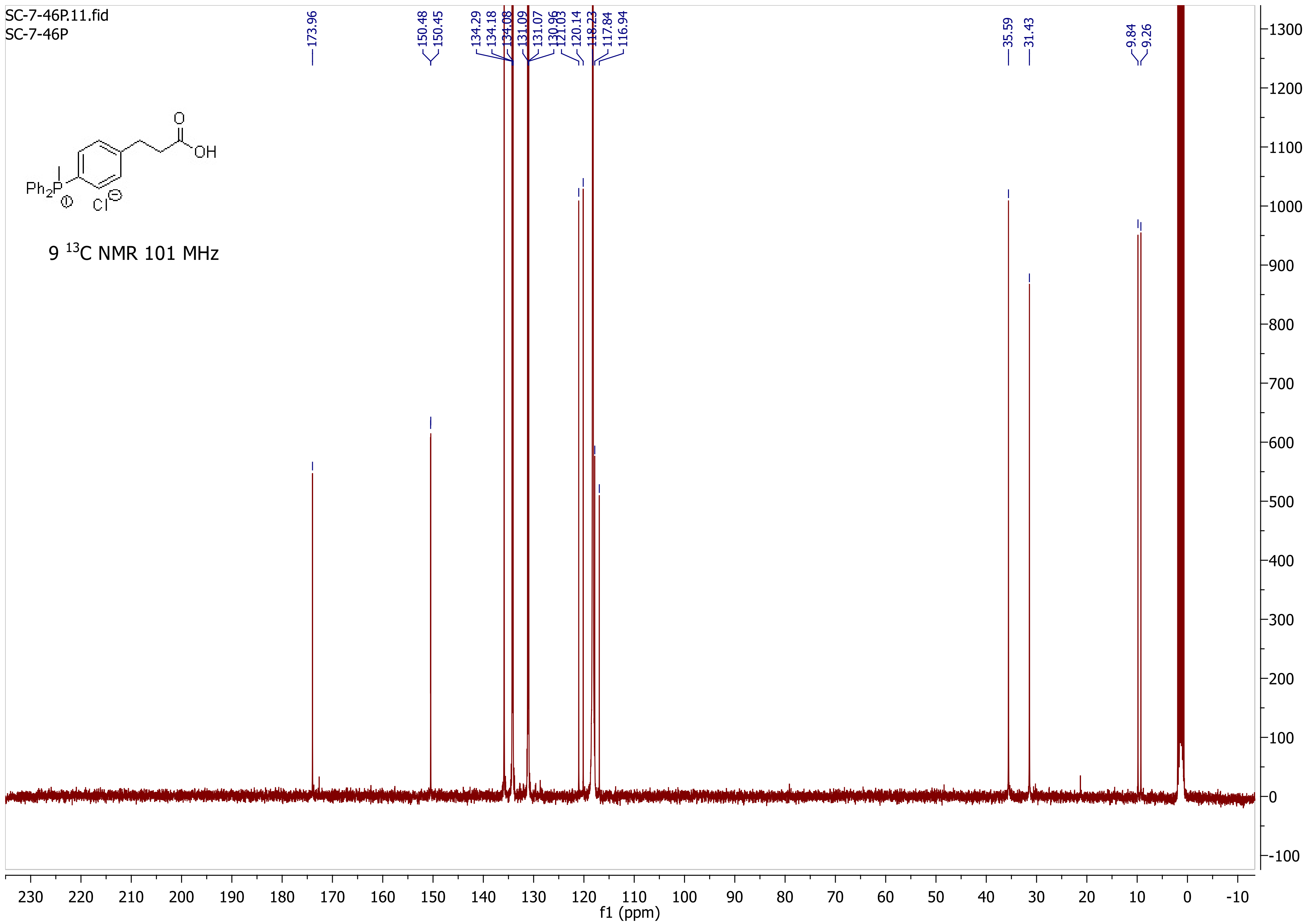

SC-7-46P.11.fid
SC-7-46P
173.96
150.48
150.45
134.29
134.18
134.08
131.09
131.07
130.96
121.03
120.14
118.23
117.84
116.94
35.59
31.43
9.84
9.26
O
OH
Ph2P
Cl
9 13C NMR 101 MHz
f1 (ppm)

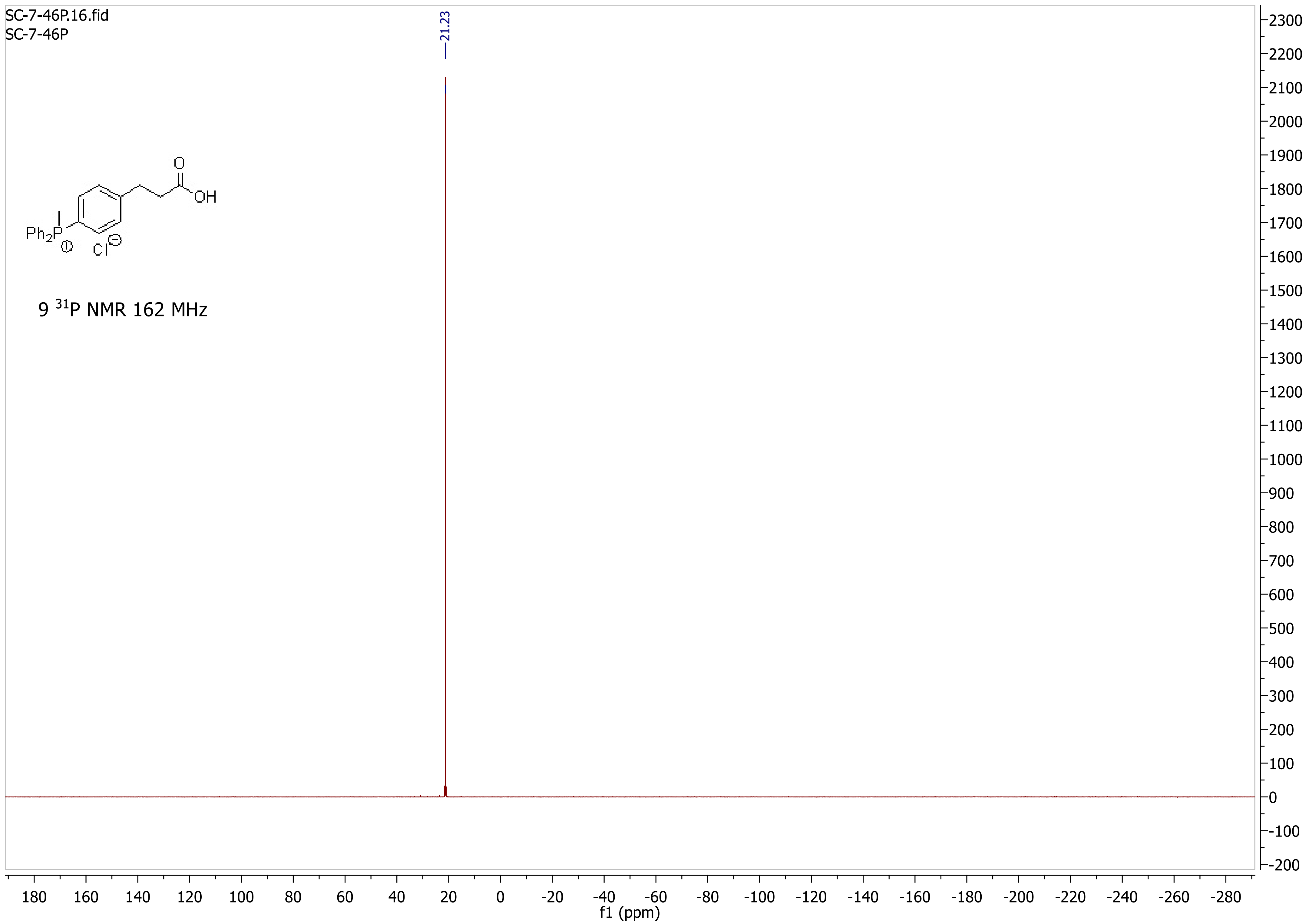
SC-7-46P.16.fid
SC-7-46P
21.23
O
OH
Ph_2P
Cl
9 ^{31}P NMR 162 MHz
f1 (ppm)

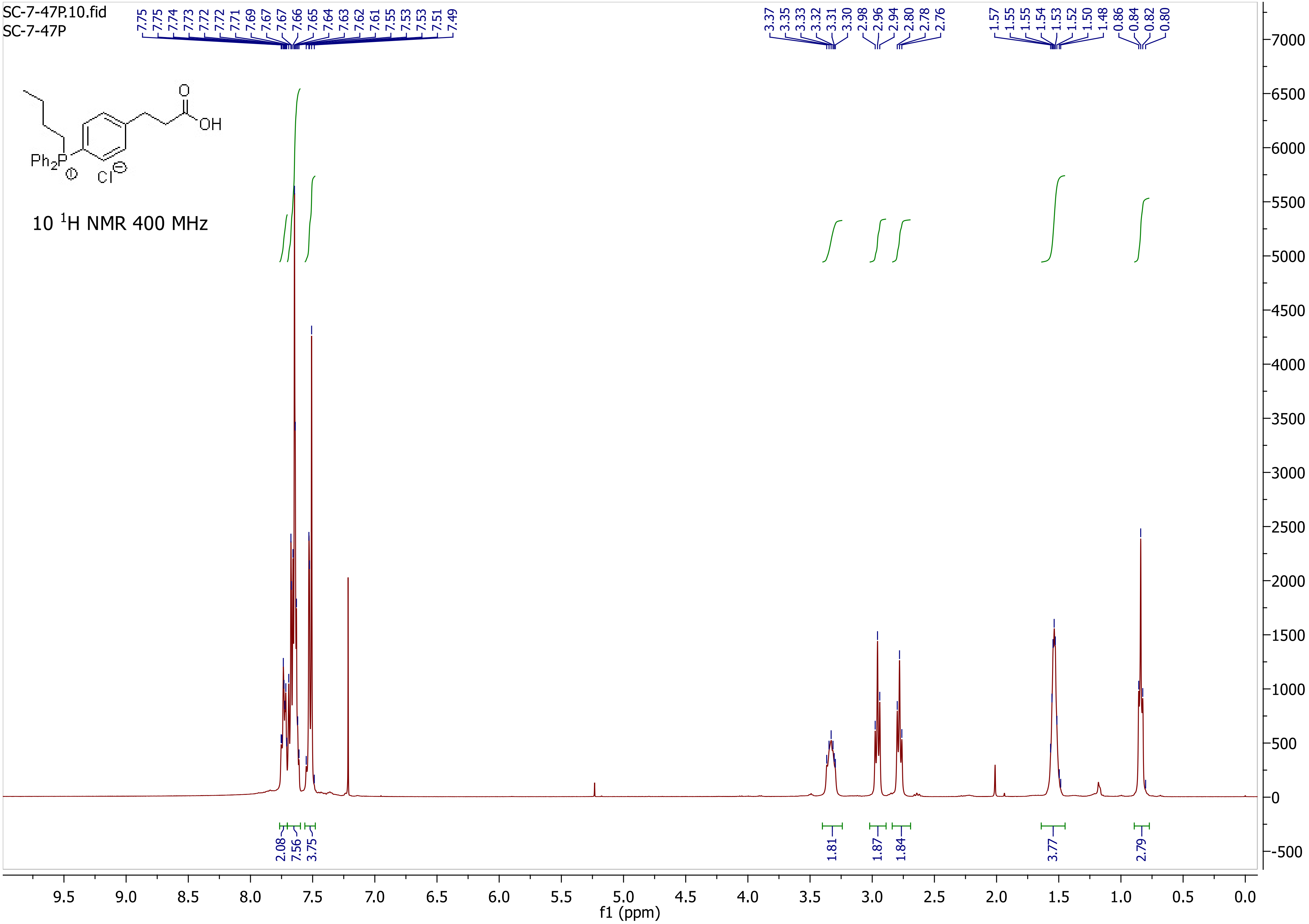
SC-7-47P.10.fid
SC-7-47P
7.75 7.75 7.74 7.73 7.72 7.72 7.71 7.69 7.67 7.67 7.66 7.65 7.64 7.63 7.62 7.61 7.55 7.53 7.53 7.51 7.49
3.37 3.35 3.33 3.32 3.31 3.30 2.98 2.96 2.94 2.80 2.78 2.76
1.57 1.55 1.55 1.54 1.53 1.52 1.50 1.48 0.86 0.84 0.82 0.80
O
OH
Ph2P
Cl
10 1H NMR 400 MHz
2.08 7.56 3.75
1.81 1.87 1.84
3.77 2.79
7000 6500 6000 5500 5000 4500 4000 3500 3000 2500 2000 1500 1000 500 0 -500
9.5 9.0 8.5 8.0 7.5 7.0 6.5 6.0 5.5 5.0 4.5 4.0 3.5 3.0 2.5 2.0 1.5 1.0 0.5 0.0
f1 (ppm)

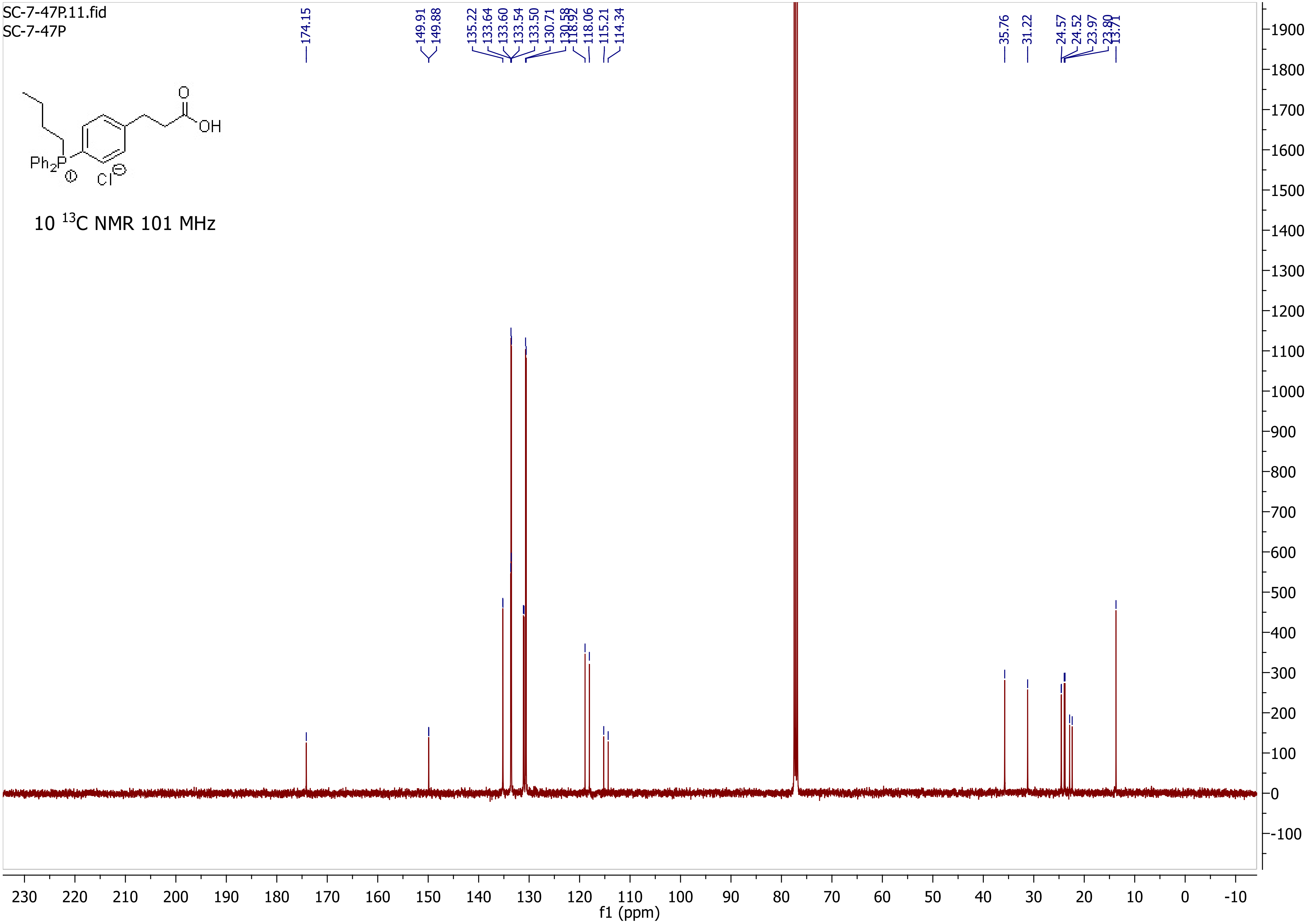
SC-7-47P.11.fid
SC-7-47P
O
OH
Ph2P
Cl
10 13C NMR 101 MHz
174.15
149.91
149.88
135.22
133.64
133.60
133.54
133.50
130.71
130.58
118.92
118.06
115.21
114.34
35.76
31.22
24.57
24.52
23.97
23.80
13.71
f1 (ppm)

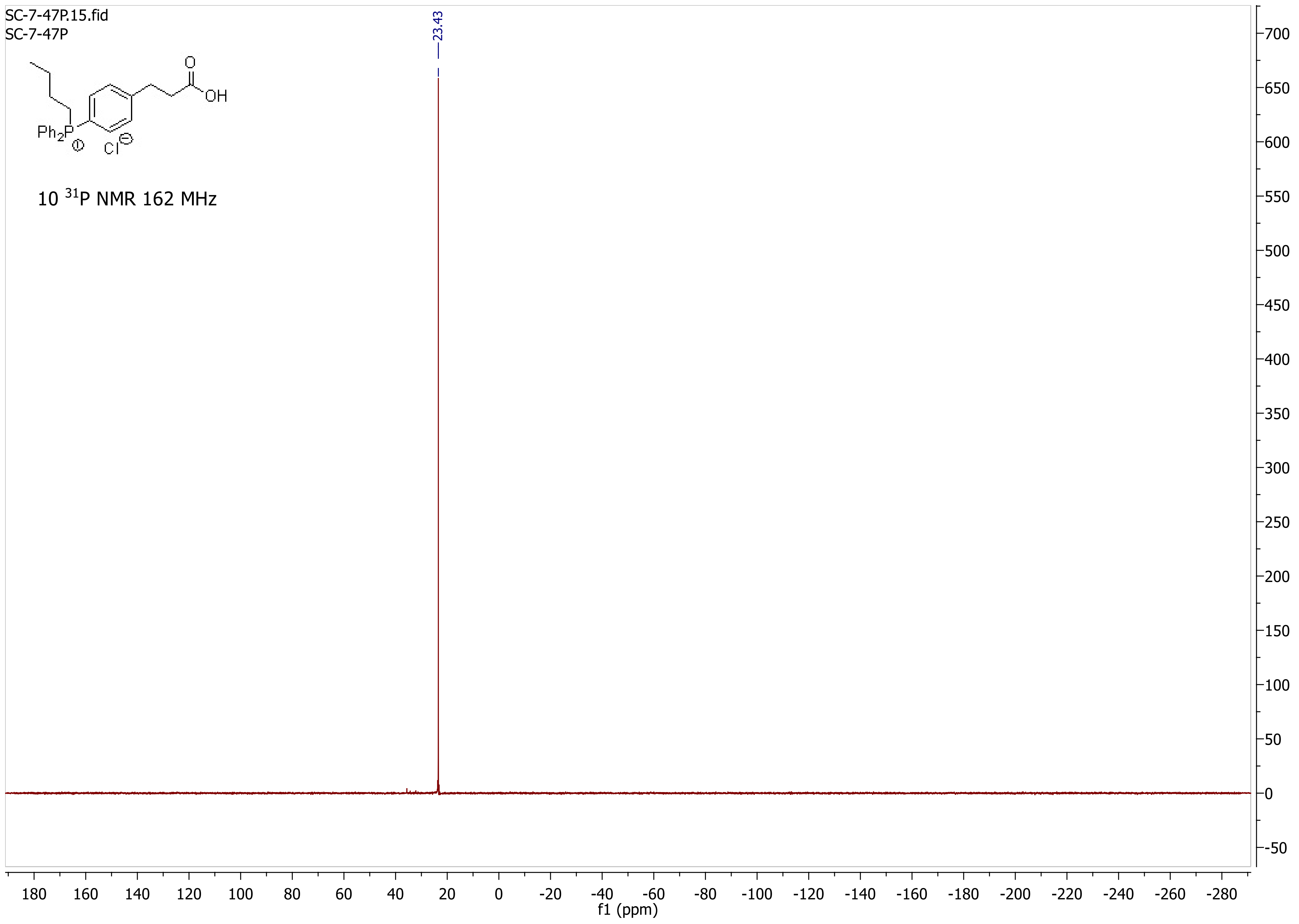
SC-7-47P.15.fid
SC-7-47P
O
OH
Ph2P
Cl
10 31P NMR 162 MHz
23.43
700
650
600
550
500
450
400
350
300
250
200
150
100
50
0
-50
180
160
140
120
100
80
60
40
20
0
-20
-40
-60
-80
-100
-120
-140
-160
-180
-200
-220
-240
-260
-280
f1 (ppm)

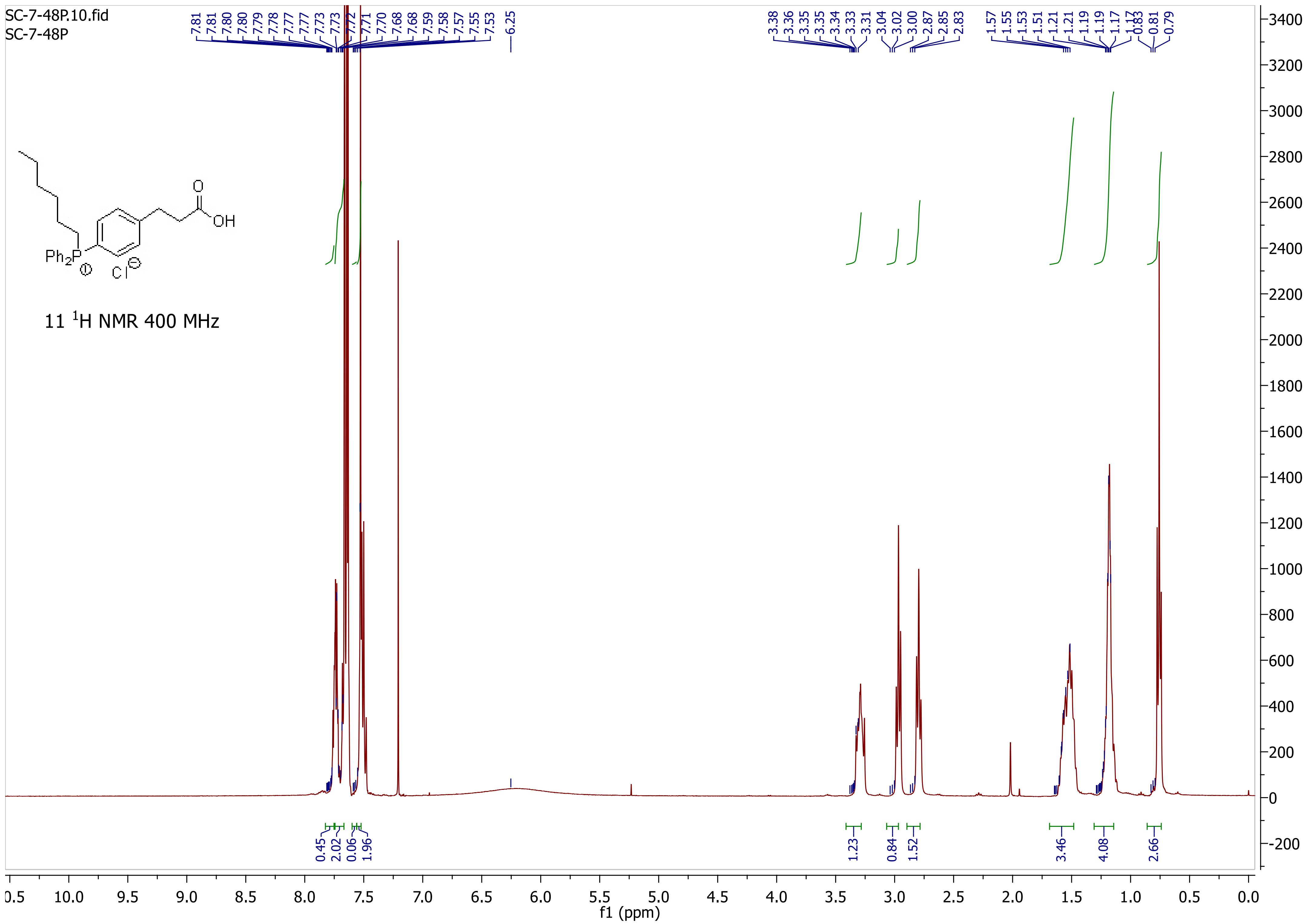
SC-7-48P.10.fid
SC-7-48P
Ph2P
Cl
O
OH
11 1H NMR 400 MHz
f1 (ppm)

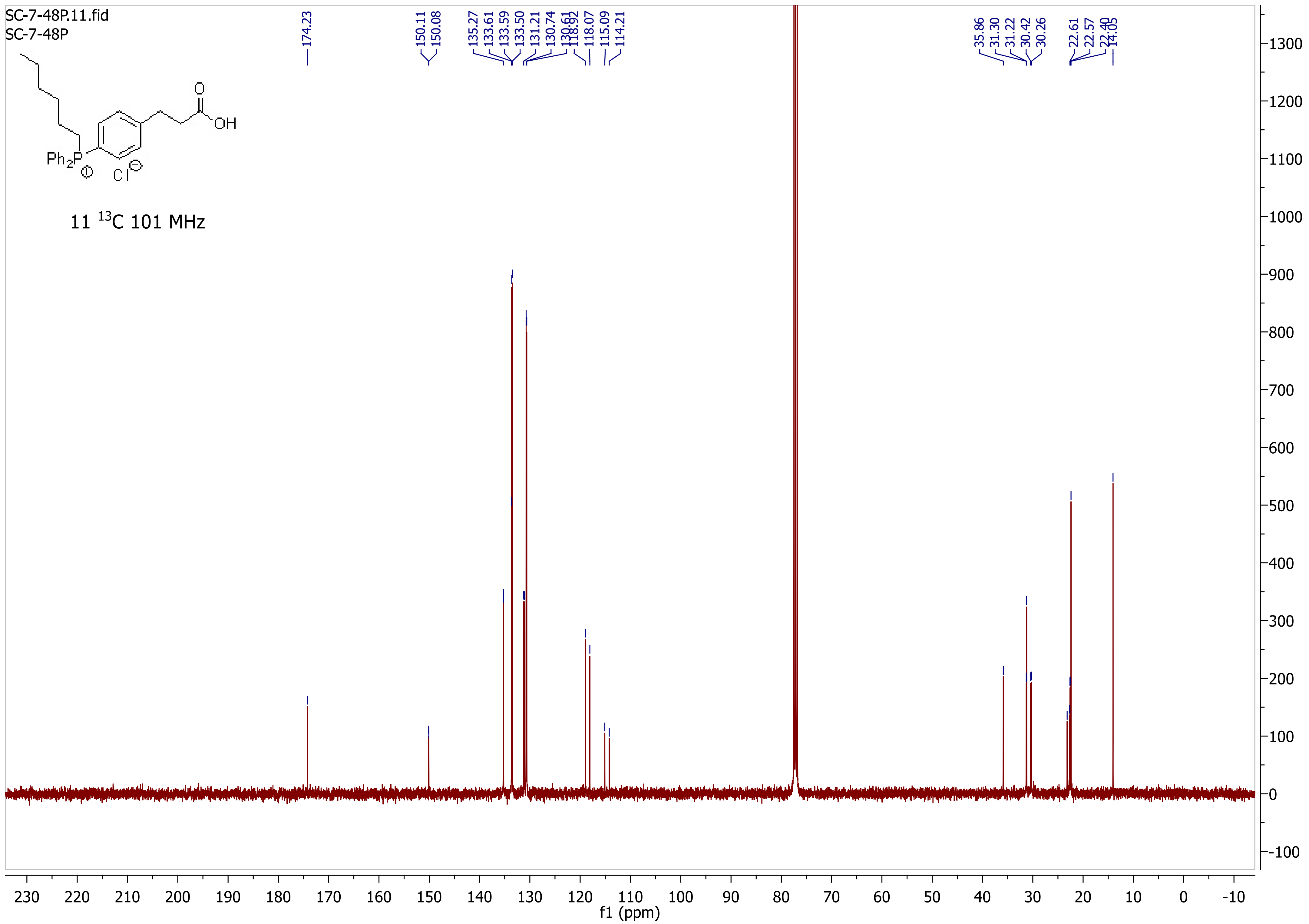
SC-7-48P.11.fid
SC-7-48P
174.23
150.11
150.08
135.27
133.61
133.59
133.50
131.21
130.74
130.61
118.92
118.07
115.09
114.21
35.86
31.30
31.22
30.42
30.26
22.61
22.57
22.40
14.05
O
OH
Ph2P
Cl
11 13C 101 MHz
f1 (ppm)

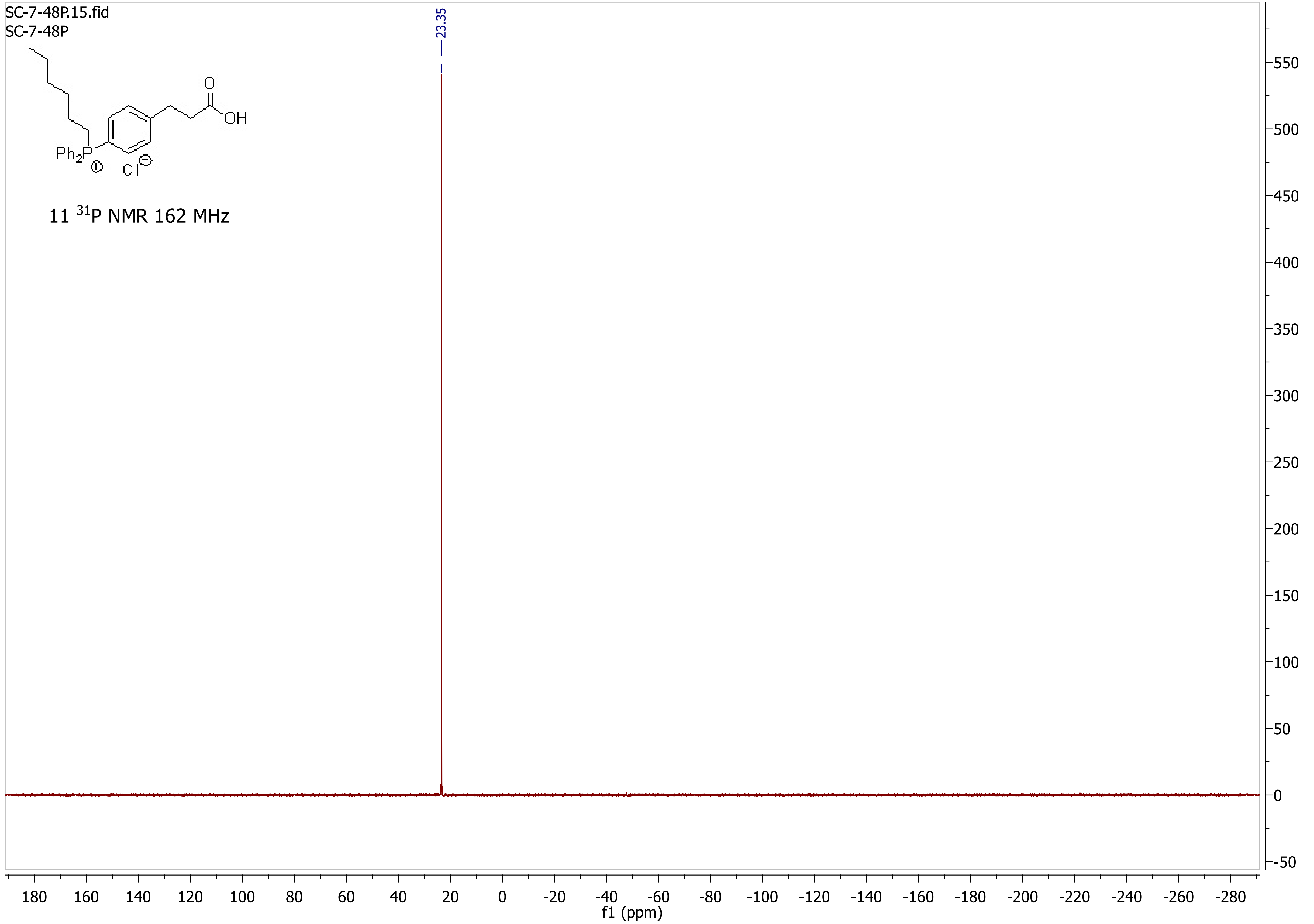
SC-7-48P.15.fid
SC-7-48P
O
OH
Ph2P
Cl
11 31P NMR 162 MHz
23.35
550
500
450
400
350
300
250
200
150
100
50
0
-50
180
160
140
120
100
80
60
40
20
0
-20
-40
-60
-80
-100
-120
-140
-160
-180
-200
-220
-240
-260
-280
f1 (ppm)

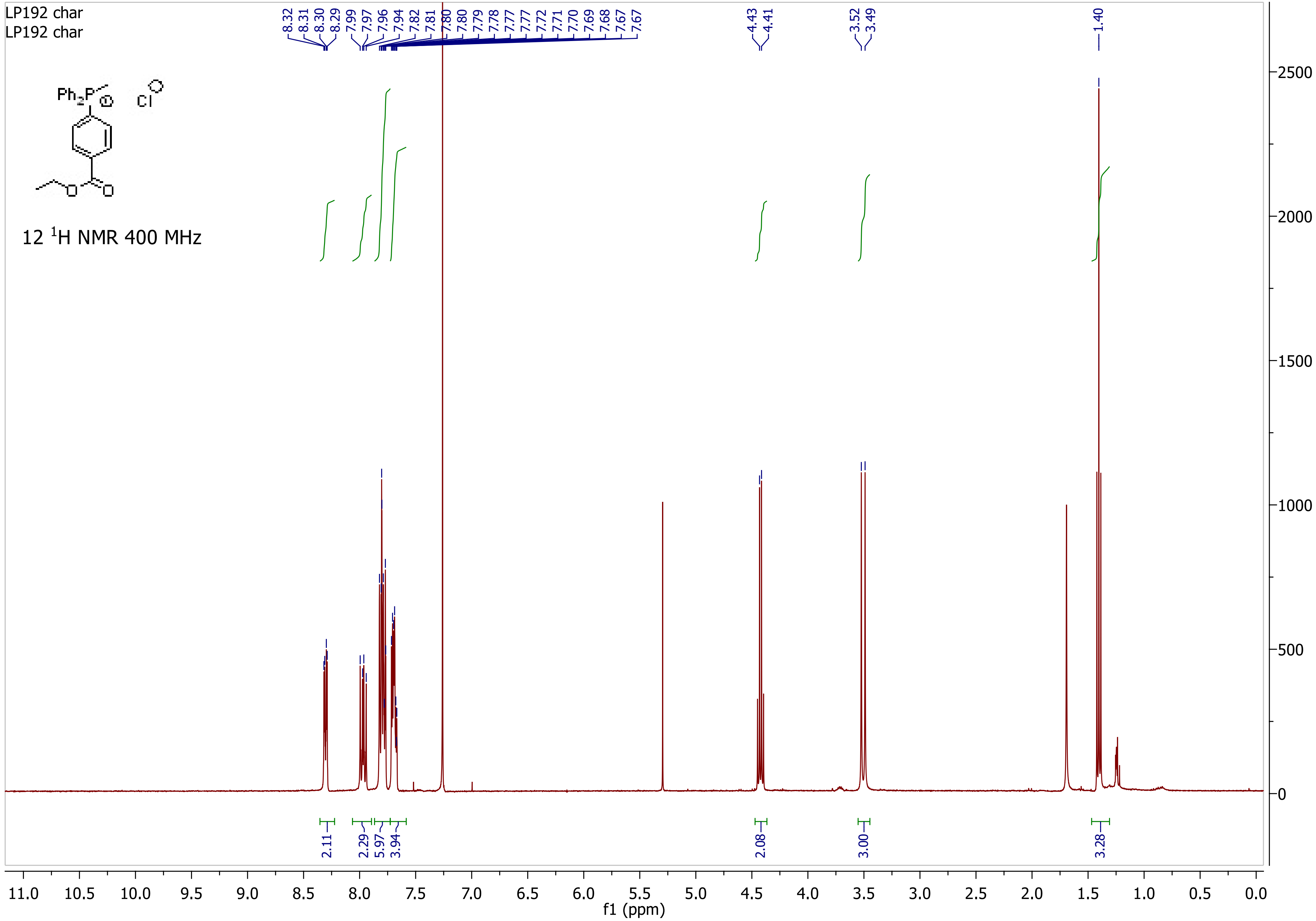
LP192 char
LP192 char
Ph2P
Cl
12 1H NMR 400 MHz
8.32
8.31
8.30
8.29
7.99
7.97
7.96
7.94
7.82
7.81
7.80
7.80
7.79
7.78
7.77
7.77
7.72
7.71
7.70
7.69
7.68
7.67
7.67
4.43
4.41
3.52
3.49
1.40
2.11
2.29
5.97
3.94
2.08
3.00
3.28
2500
2000
1500
1000
500
0
11.0
10.5
10.0
9.5
9.0
8.5
8.0
7.5
7.0
6.5
6.0
5.5
5.0
4.5
4.0
3.5
3.0
2.5
2.0
1.5
1.0
0.5
0.0
f1 (ppm)

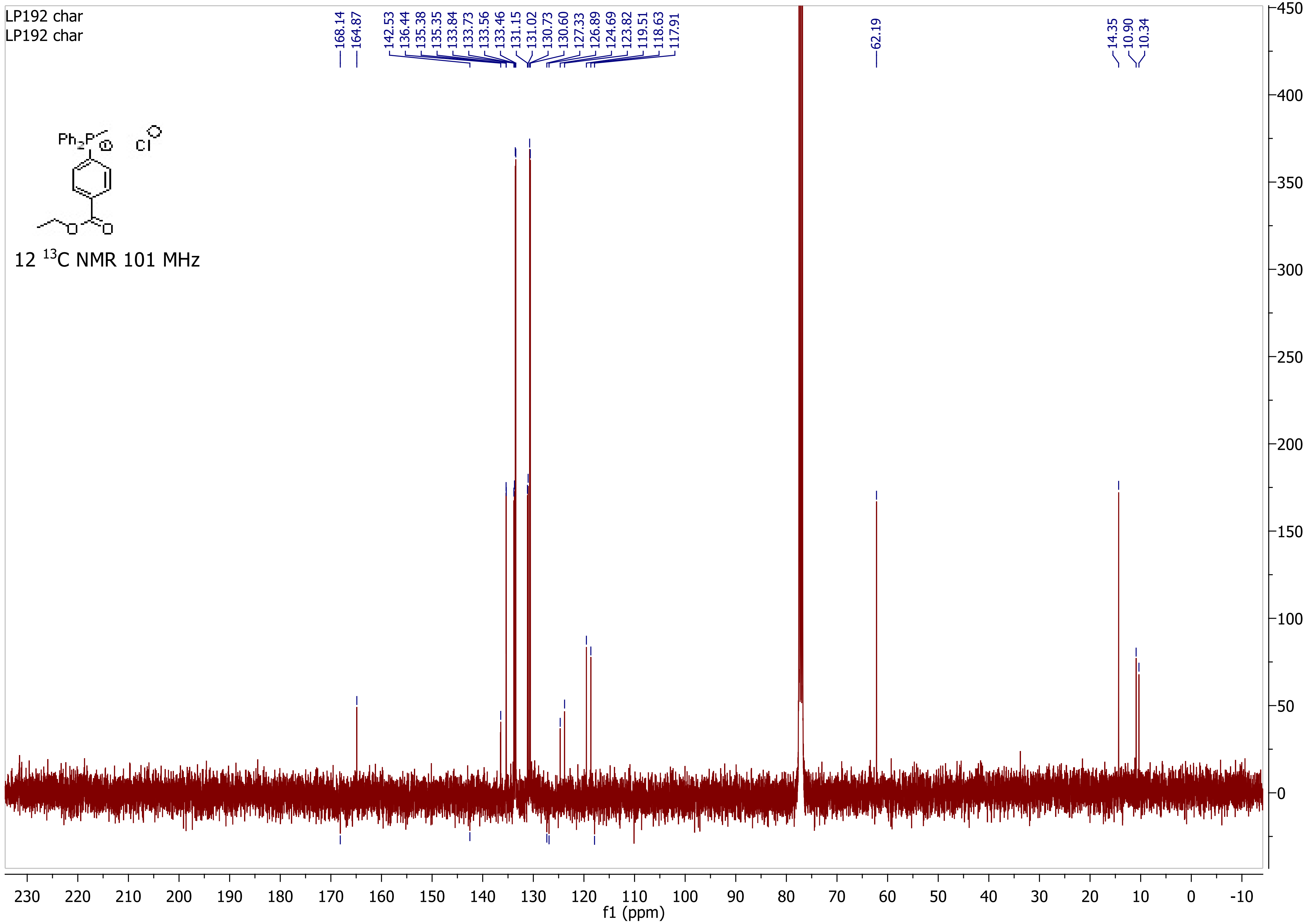
LP192 char
LP192 char
168.14
164.87
142.53
136.44
135.38
135.35
133.84
133.73
133.56
133.46
131.15
131.02
130.73
130.60
127.33
126.89
124.69
123.82
119.51
118.63
117.91
62.19
14.35
10.90
10.34
Ph2P
Cl
12 13C NMR 101 MHz
f1 (ppm)

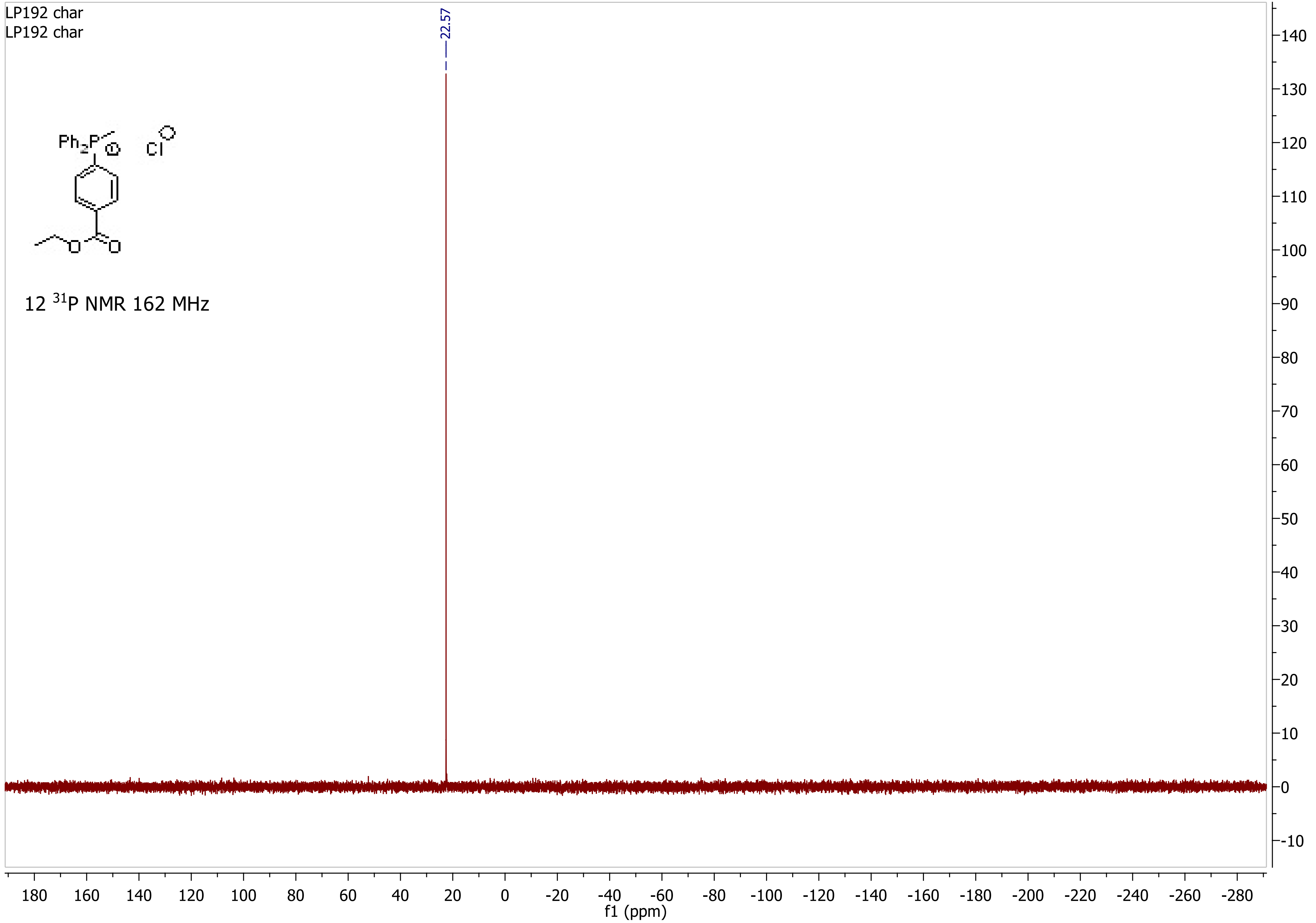

LP192 char
LP192 char
22.57
Ph2P
Cl
12 31P NMR 162 MHz
f1 (ppm)

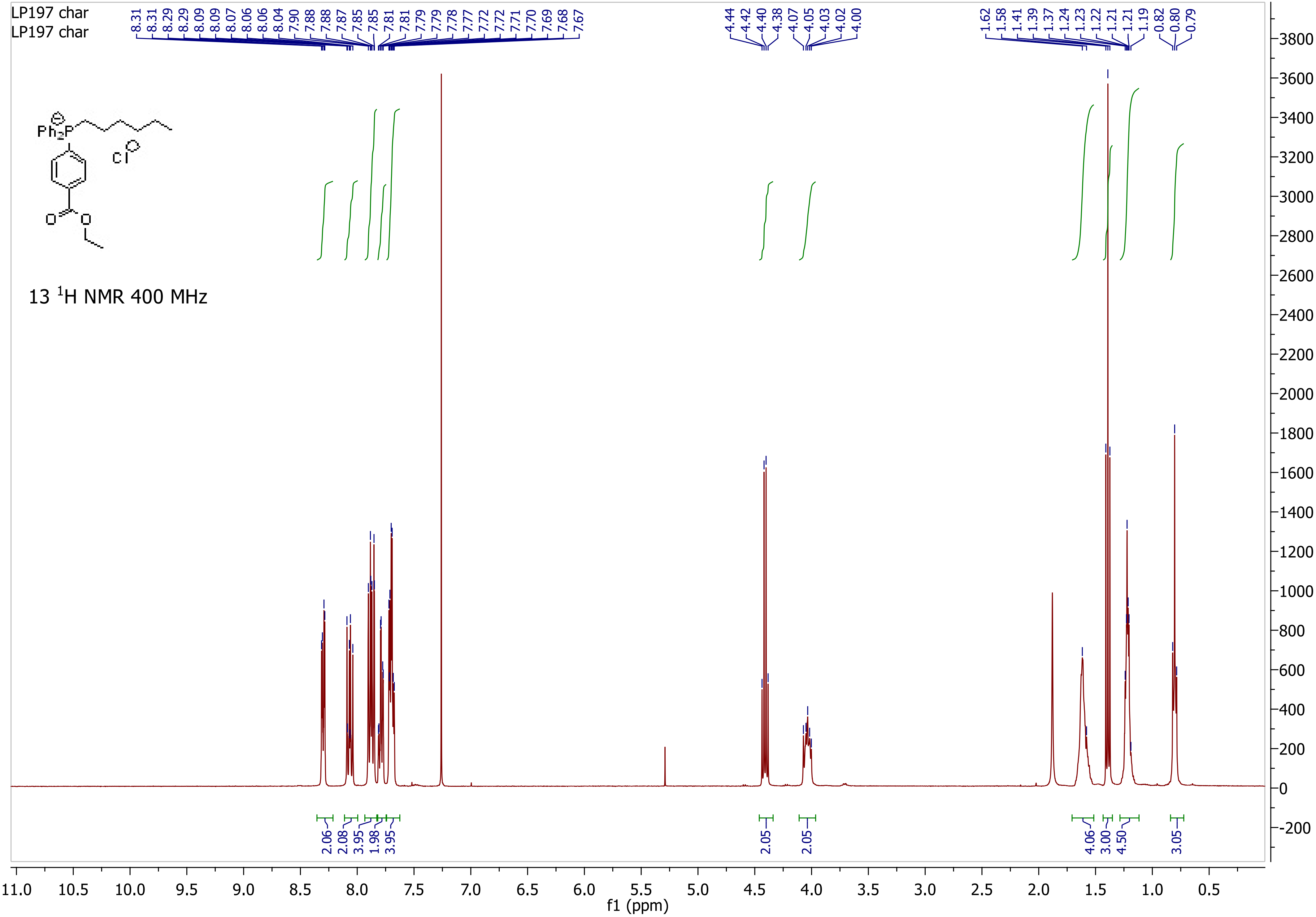

LP197 char
LP197 char
8.31 8.31 8.29 8.29 8.09 8.09 8.07 8.06 8.06 8.04 7.90 7.88 7.88 7.87 7.85 7.85 7.81 7.81 7.79 7.79 7.78 7.77 7.72 7.72 7.71 7.70 7.69 7.68 7.67
4.44 4.42 4.40 4.38 4.07 4.05 4.03 4.02 4.00
1.62 1.58 1.41 1.39 1.37 1.24 1.23 1.22 1.21 1.21 1.19 0.82 0.80 0.79
Ph2P
Cl
O
O
13 1H NMR 400 MHz
2.06 2.08 3.95 1.98 3.95
2.05 2.05
4.06 3.00 4.50 3.05
f1 (ppm)

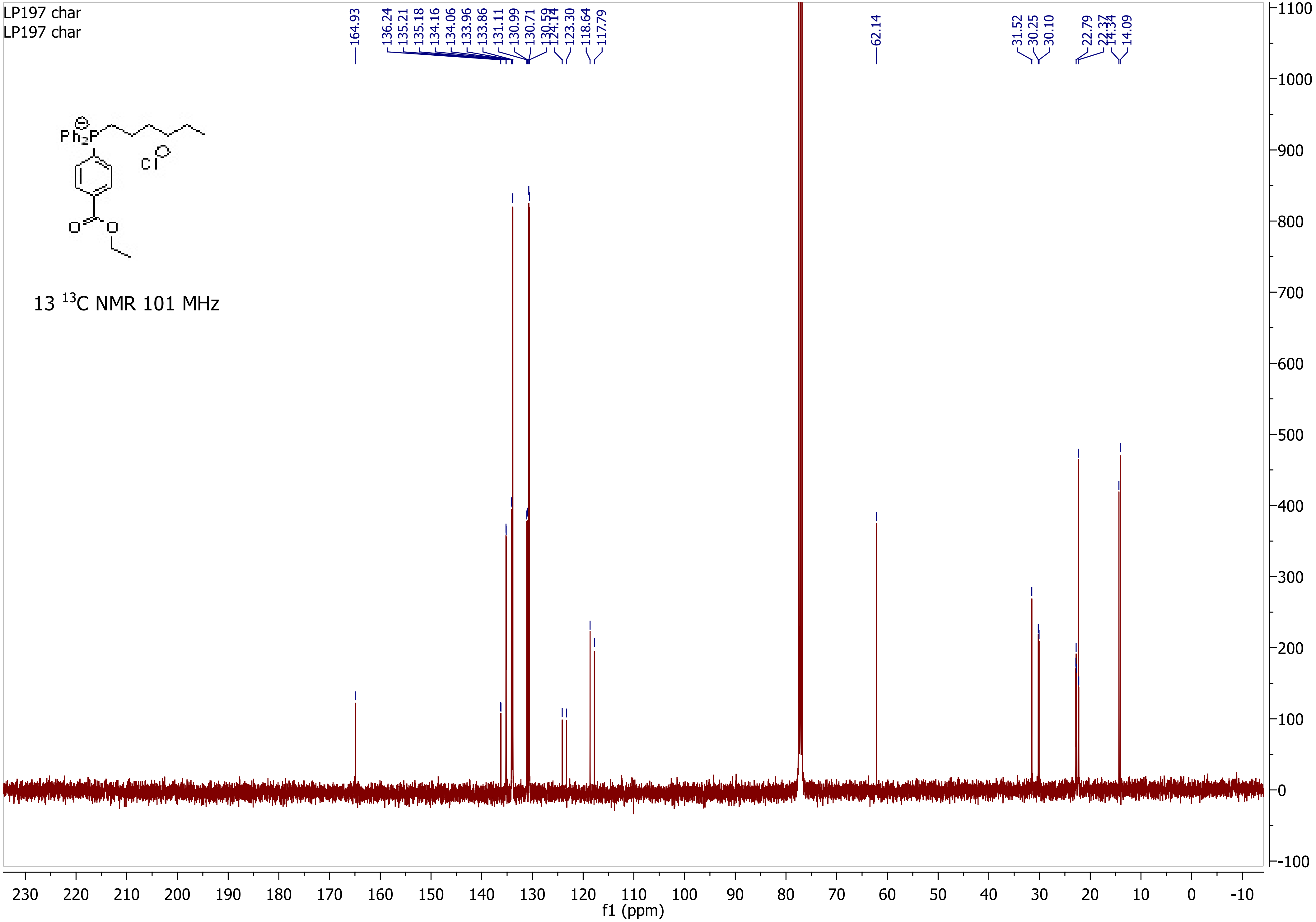
LP197 char
LP197 char
164.93
136.24
135.21
135.18
134.16
134.06
133.96
133.86
131.11
130.99
130.71
130.59
124.14
123.30
118.64
117.79
62.14
31.52
30.25
30.10
22.79
22.37
14.34
14.09
Ph2P
Cl
O
O
13 13C NMR 101 MHz
f1 (ppm)

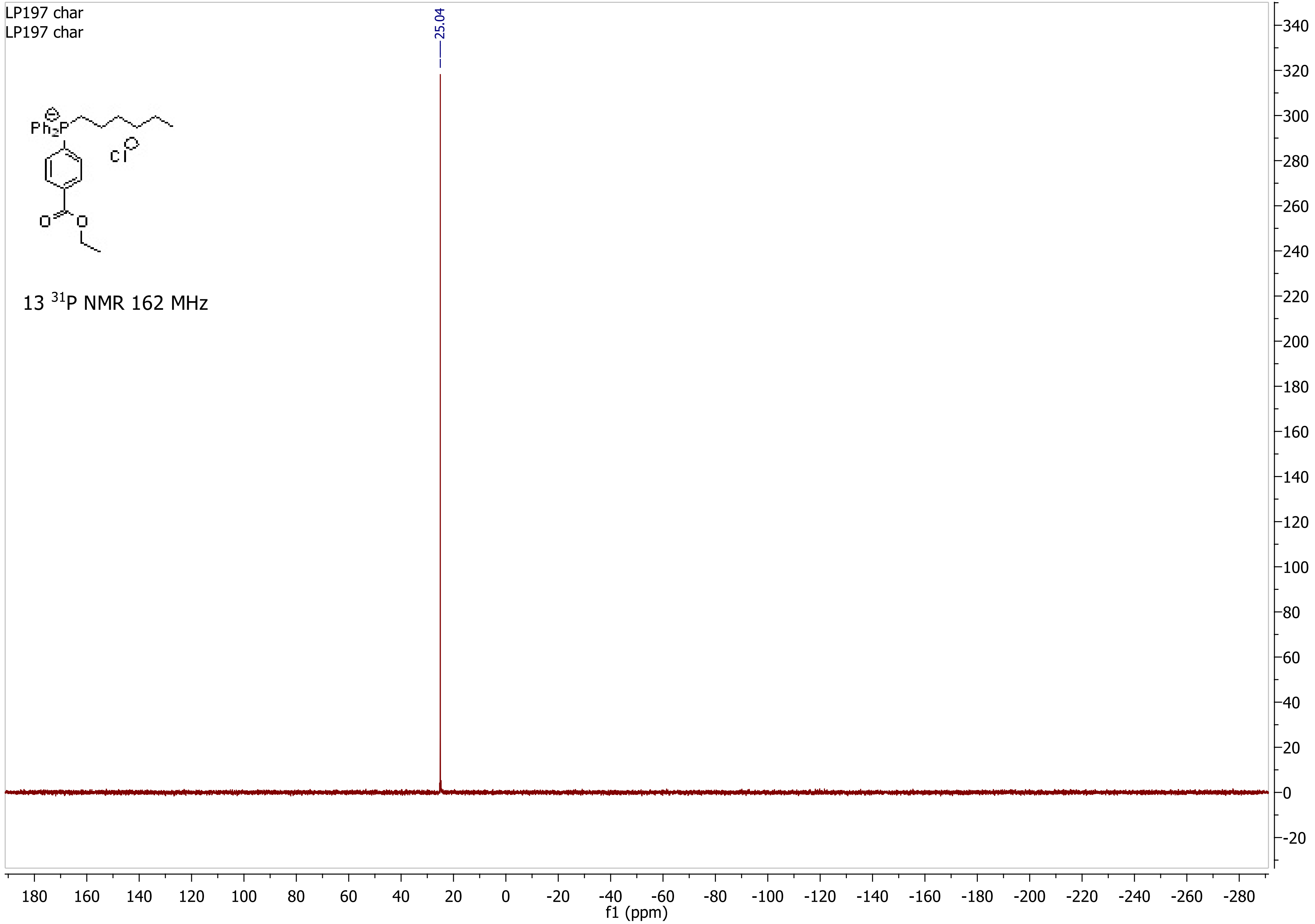
LP197 char
LP197 char
13 31P NMR 162 MHz
25.04
f1 (ppm)

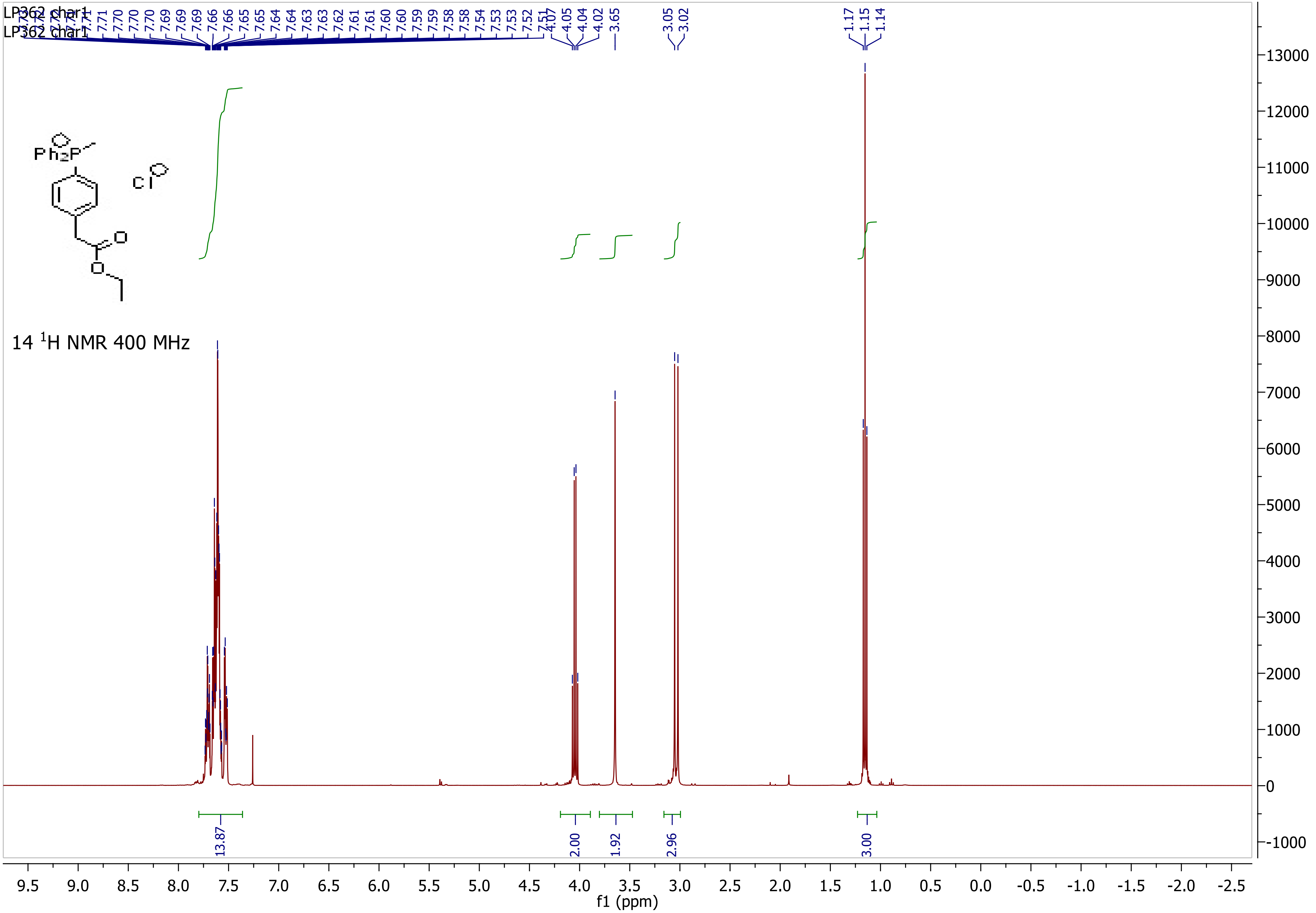
LP362 char1
LP362 char1
7.71
7.70
7.70
7.70
7.69
7.69
7.69
7.66
7.66
7.65
7.65
7.64
7.64
7.63
7.63
7.62
7.61
7.61
7.60
7.59
7.59
7.58
7.58
7.54
7.53
7.53
7.52
7.51
4.07
4.05
4.04
4.02
3.65
3.05
3.02
1.17
1.15
1.14
Ph2P
Cl
14 1H NMR 400 MHz
13.87
2.00
1.92
2.96
3.00
f1 (ppm)

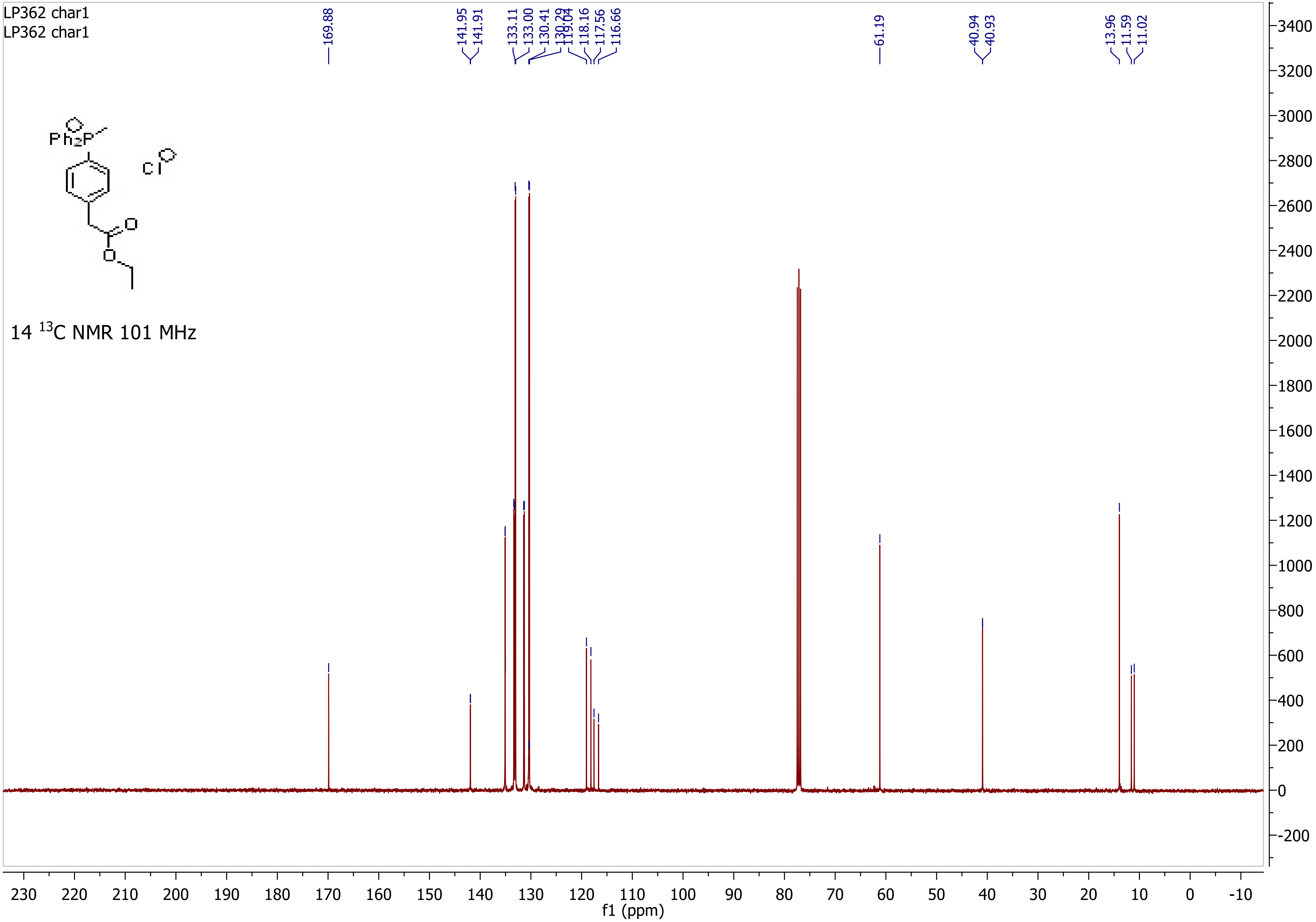

LP362 char1
LP362 char1
169.88
141.95
141.91
133.11
133.00
130.41
130.29
119.04
118.16
117.56
116.66
61.19
40.94
40.93
13.96
11.59
11.02
Ph2P
Cl
O
14 13C NMR 101 MHz
f1 (ppm)

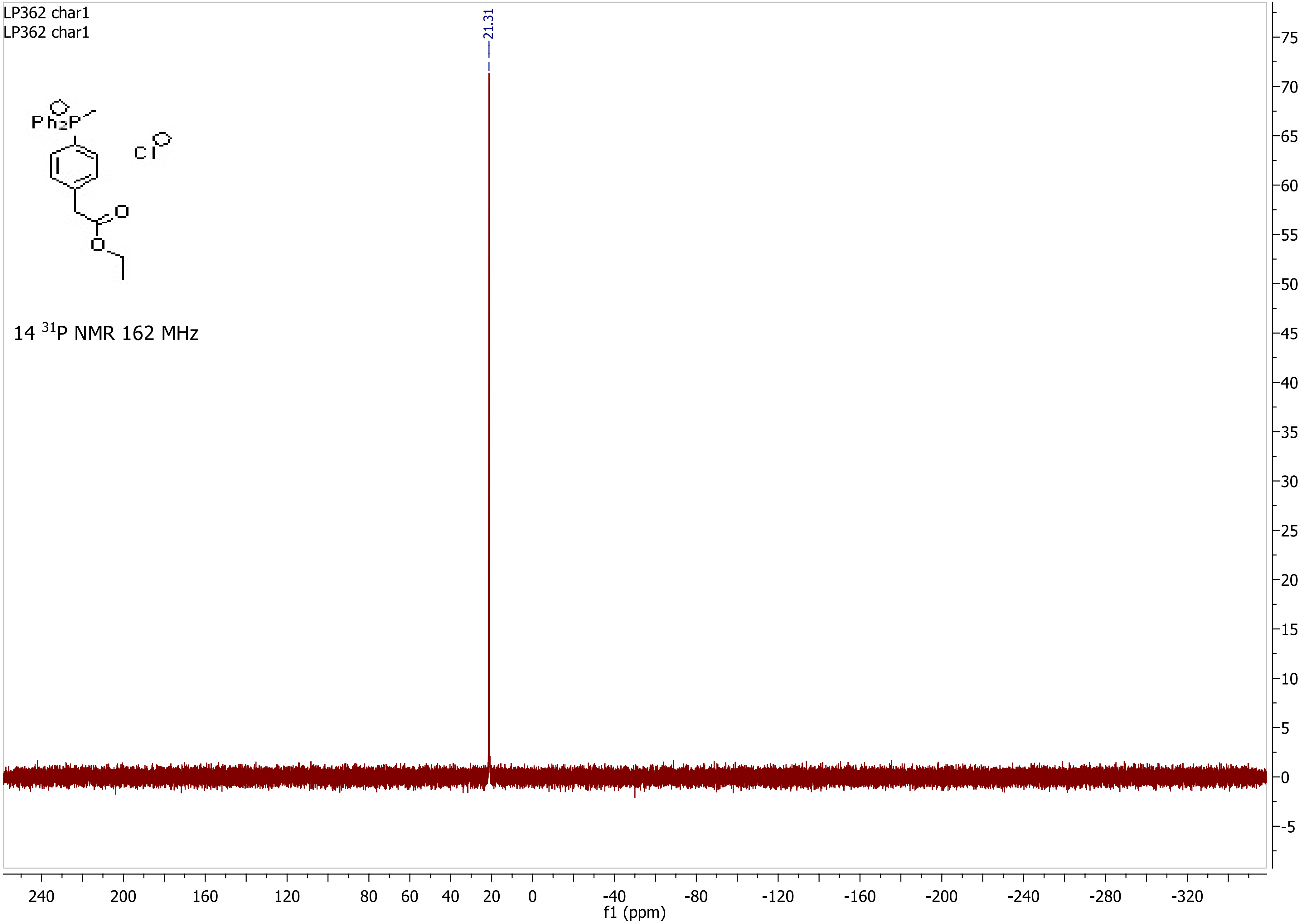
LP362 char1
LP362 char1
21.31
Ph2P
Cl
O
14 31P NMR 162 MHz
f1 (ppm)

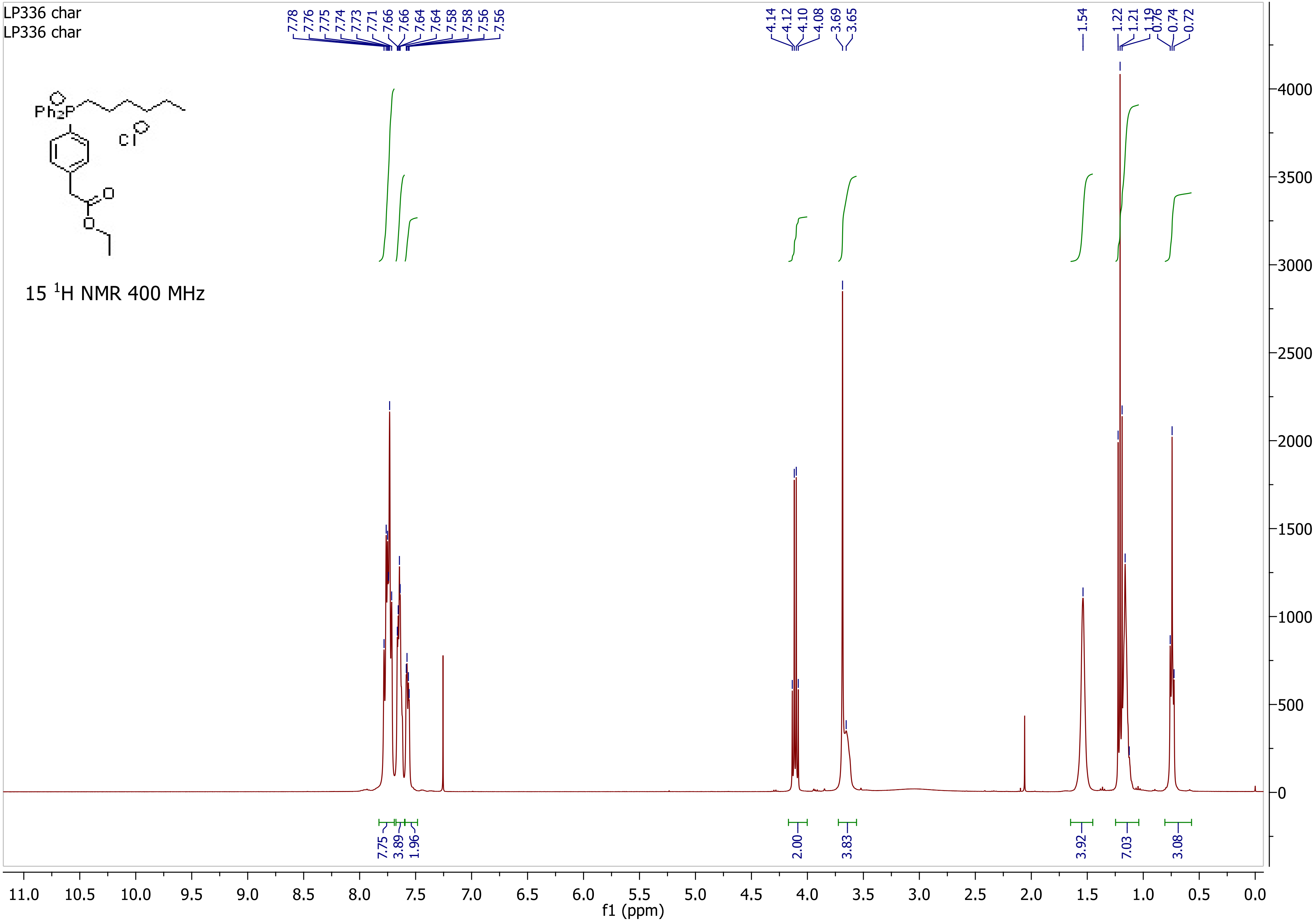
LP336 char
LP336 char
15 ¹H NMR 400 MHz
Ph2P
Cl
7.78
7.76
7.75
7.74
7.73
7.71
7.66
7.66
7.64
7.64
7.58
7.58
7.56
7.56
4.14
4.12
4.10
4.08
3.69
3.65
1.54
1.22
1.21
1.19
0.76
0.74
0.72
7.75
3.89
1.96
2.00
3.83
3.92
7.03
3.08
4000
3500
3000
2500
2000
1500
1000
500
0
11.0
10.5
10.0
9.5
9.0
8.5
8.0
7.5
7.0
6.5
6.0
5.5
5.0
4.5
4.0
3.5
3.0
2.5
2.0
1.5
1.0
0.5
0.0
f1 (ppm)

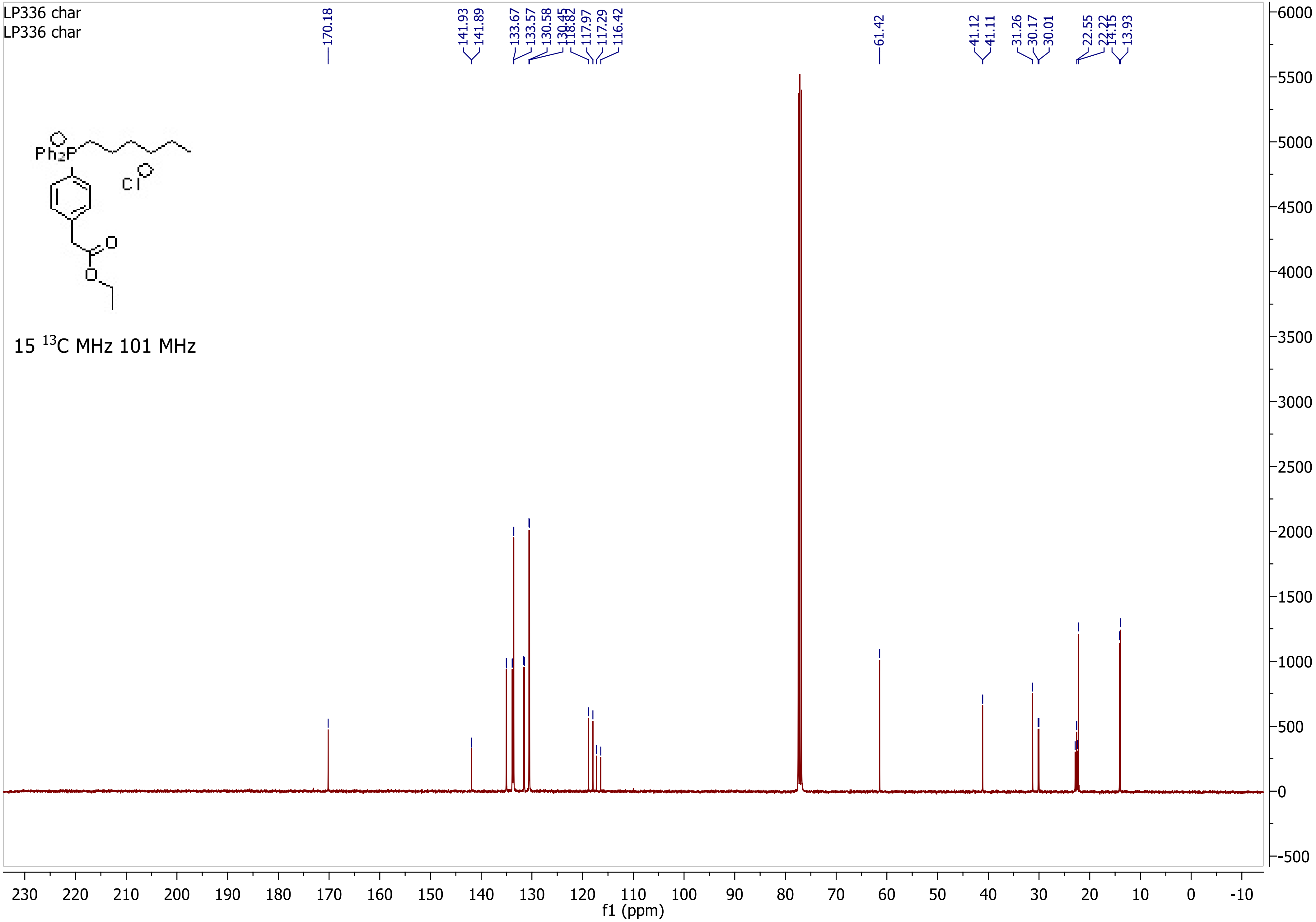

LP336 char
LP336 char
170.18
141.93
141.89
133.67
133.57
130.58
130.45
118.82
117.97
117.29
116.42
61.42
41.12
41.11
31.26
30.17
30.01
22.55
22.22
14.15
13.93
Ph2P
O
Cl
15 13C MHz 101 MHz
f1 (ppm)

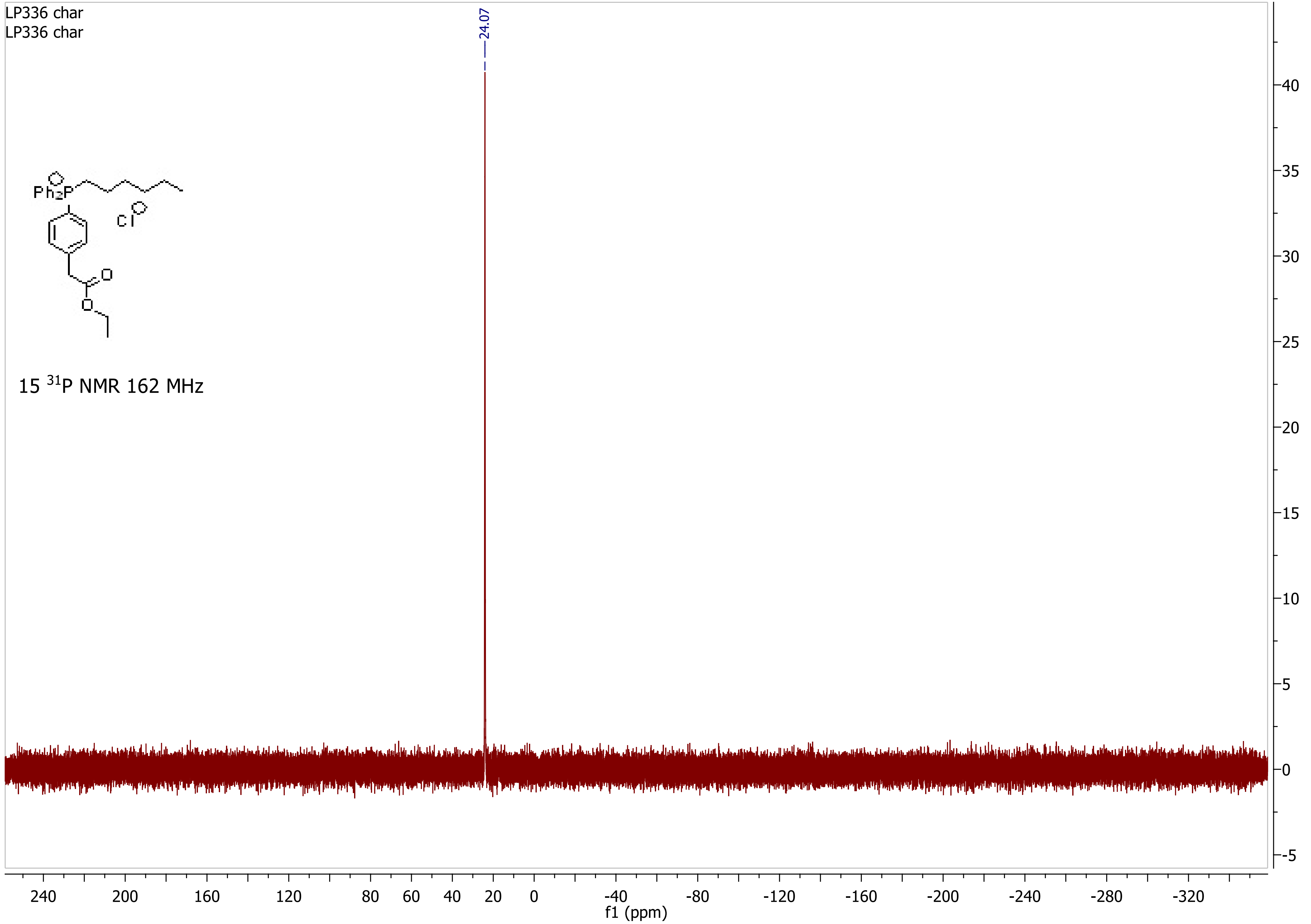
LP336 char
LP336 char
24.07
Ph2P
Cl
15 31P NMR 162 MHz
240 200 160 120 80 60 40 20 0 -40 -80 -120 -160 -200 -240 -280 -320
f1 (ppm)
40 35 30 25 20 15 10 5 0 -5

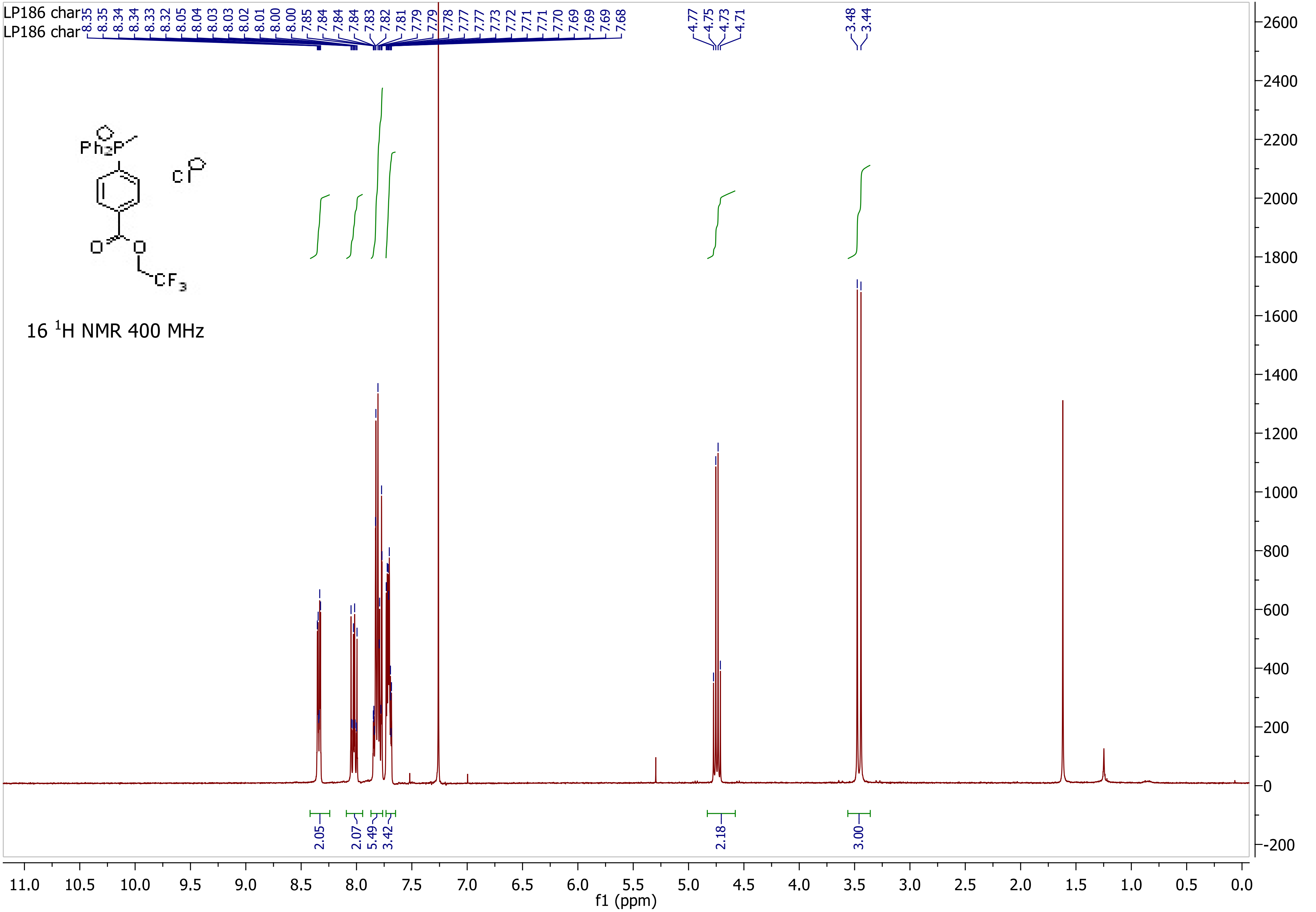
LP186 char
LP186 char
8.35 8.35 8.34 8.34 8.33 8.32 8.05 8.04 8.03 8.03 8.02 8.01 8.00 8.00 7.85 7.84 7.84 7.83 7.82 7.81 7.79 7.79 7.78 7.77 7.77 7.73 7.72 7.71 7.71 7.70 7.69 7.69 7.69 7.68
4.77 4.75 4.73 4.71
3.48 3.44
Ph2P
Cl
O
O
CF3
16 1H NMR 400 MHz
2.05
2.07
5.49
3.42
2.18
3.00
f1 (ppm)

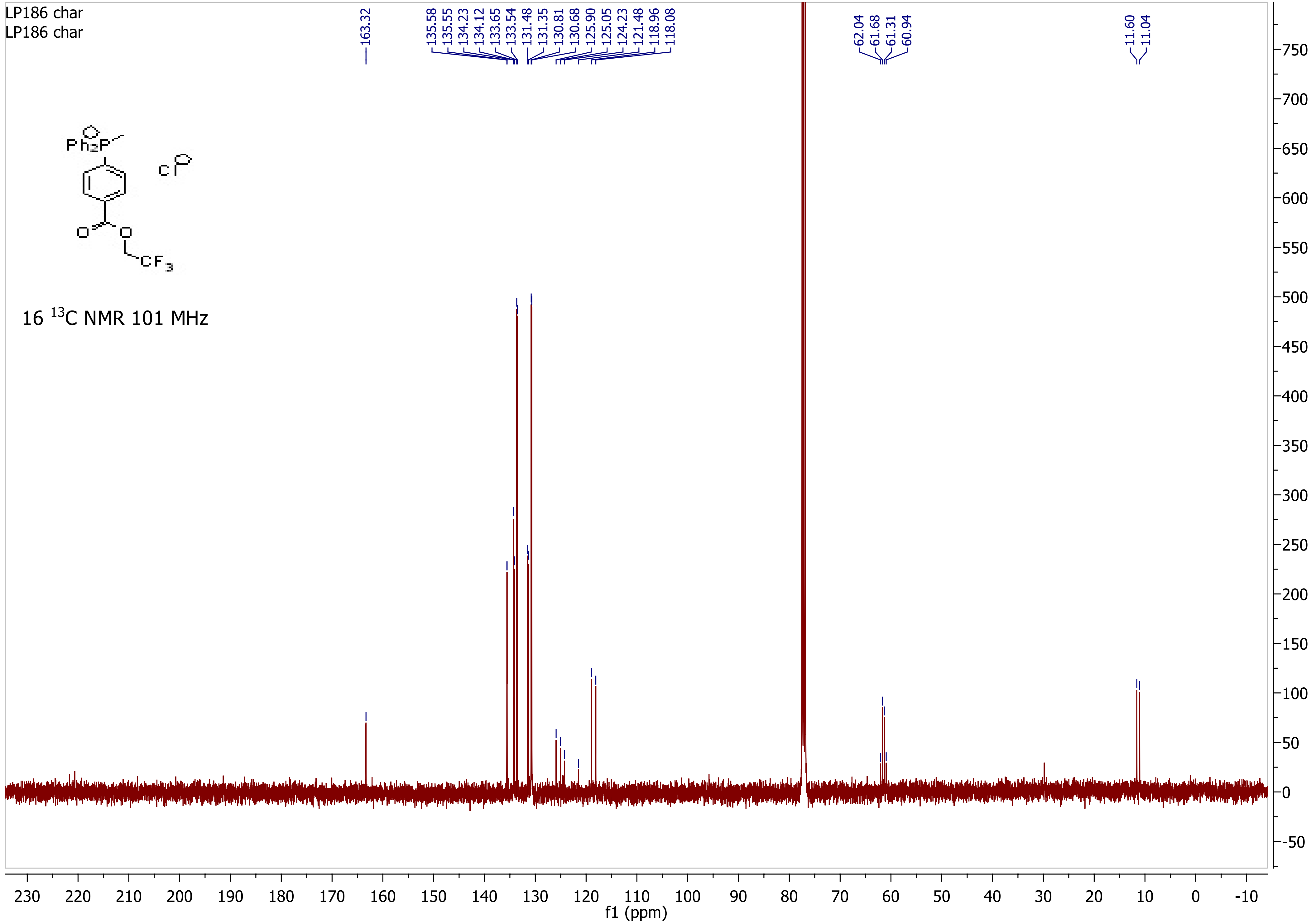
LP186 char
LP186 char
16 13C NMR 101 MHz
163.32
135.58
135.55
134.23
134.12
133.65
133.54
131.48
131.35
130.81
130.68
125.90
125.05
124.23
121.48
118.96
118.08
62.04
61.68
61.31
60.94
11.60
11.04
Ph2P
Cl
O
O
CF3
f1 (ppm)

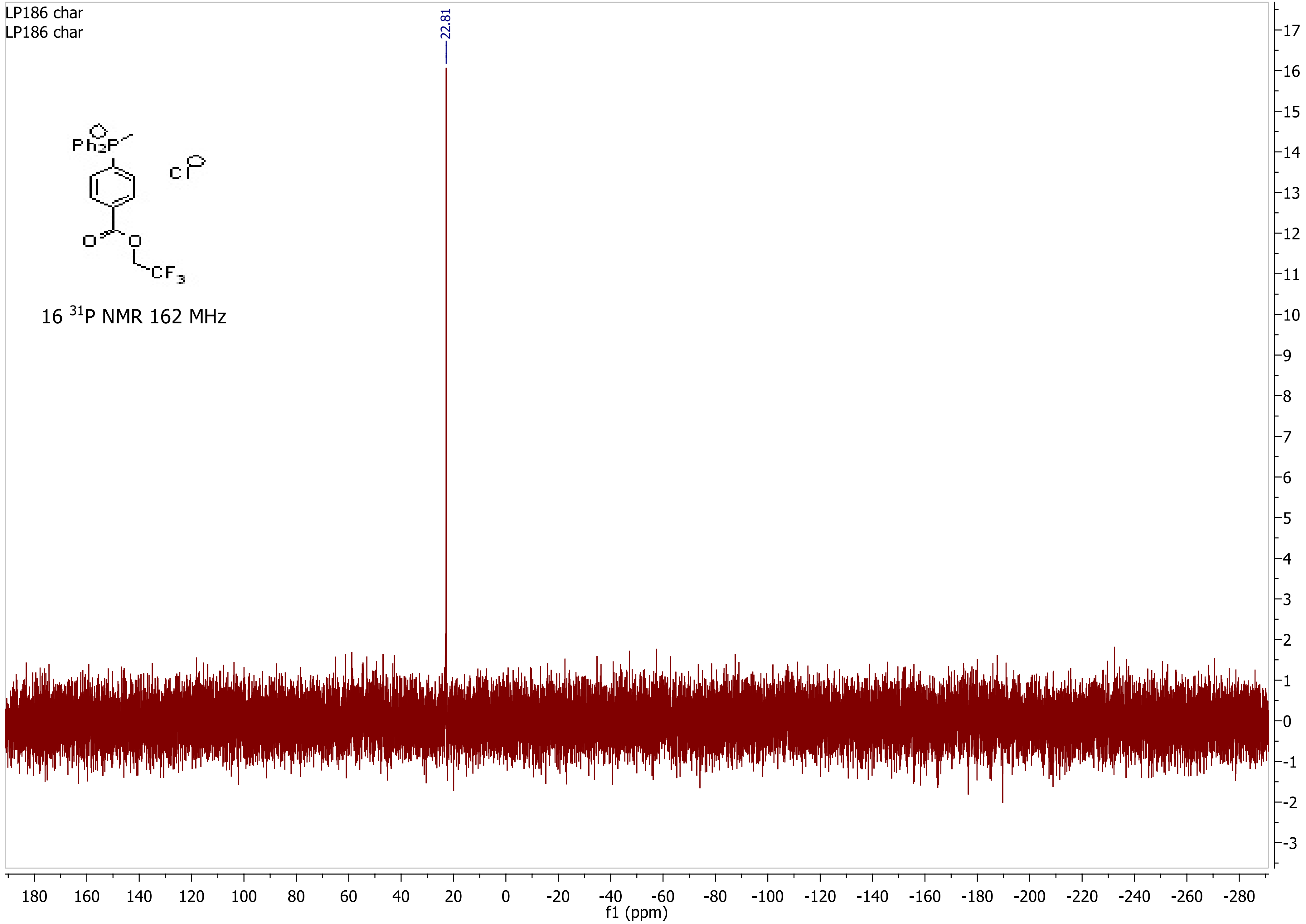
LP186 char
LP186 char
22.81
Ph2P
Cl
O
CF3
16 31P NMR 162 MHz
f1 (ppm)
180 160 140 120 100 80 60 40 20 0 -20 -40 -60 -80 -100 -120 -140 -160 -180 -200 -220 -240 -260 -280
17 16 15 14 13 12 11 10 9 8 7 6 5 4 3 2 1 0 -1 -2 -3

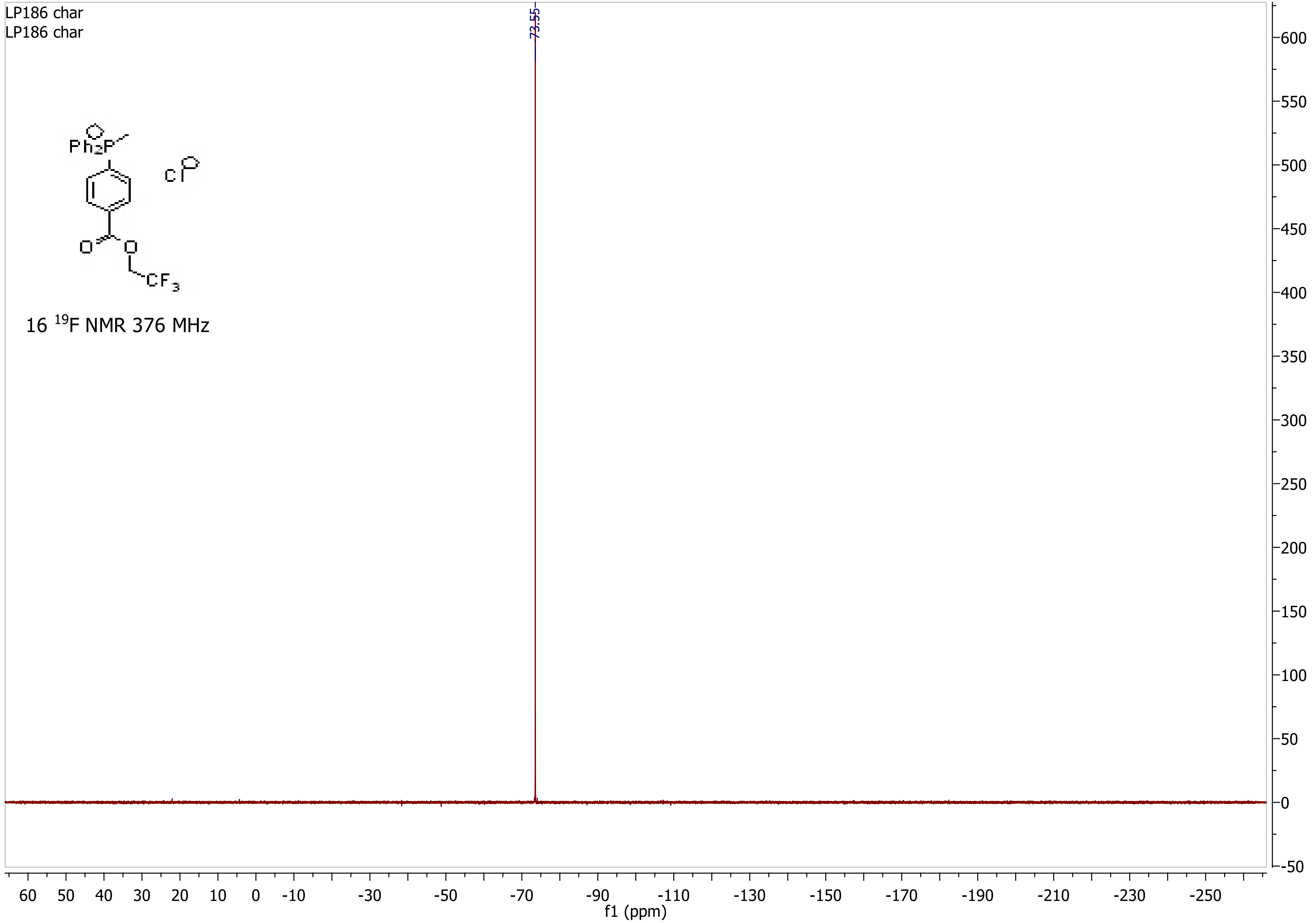
LP186 char
LP186 char
-73.55
16 ^19F NMR 376 MHz
f1 (ppm)

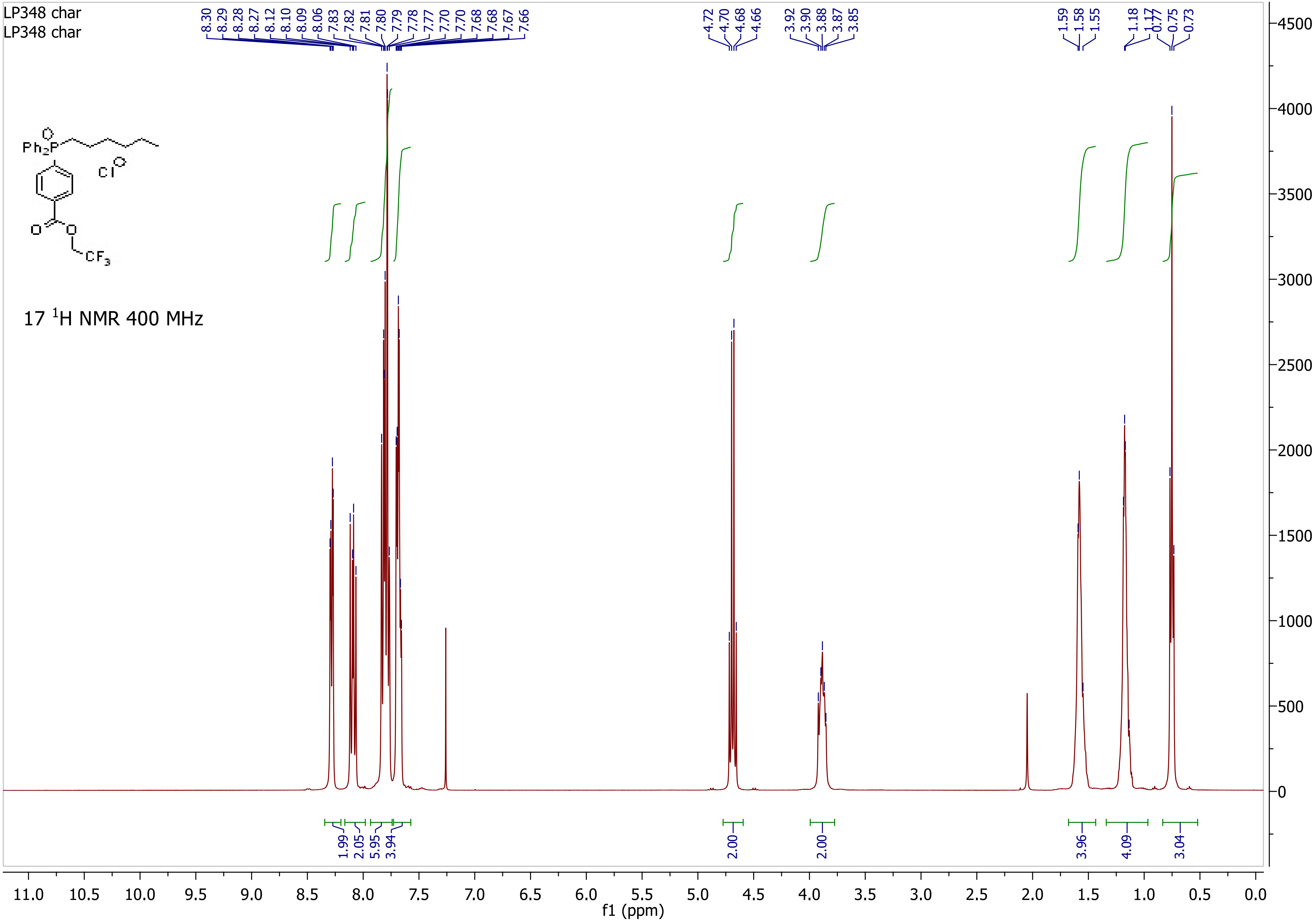

LP348 char
LP348 char
17 ¹H NMR 400 MHz
8.30 8.29 8.28 8.27 8.12 8.10 8.09 8.06 7.83 7.82 7.81 7.80 7.79 7.78 7.77 7.70 7.70 7.68 7.68 7.67 7.66
4.72 4.70 4.68 4.66
3.92 3.90 3.88 3.87 3.85
1.59 1.58 1.55
1.18 1.17 0.77 0.75 0.73
1.99 2.05 5.95 3.94 2.00 2.00 3.96 4.09 3.04
f1 (ppm)

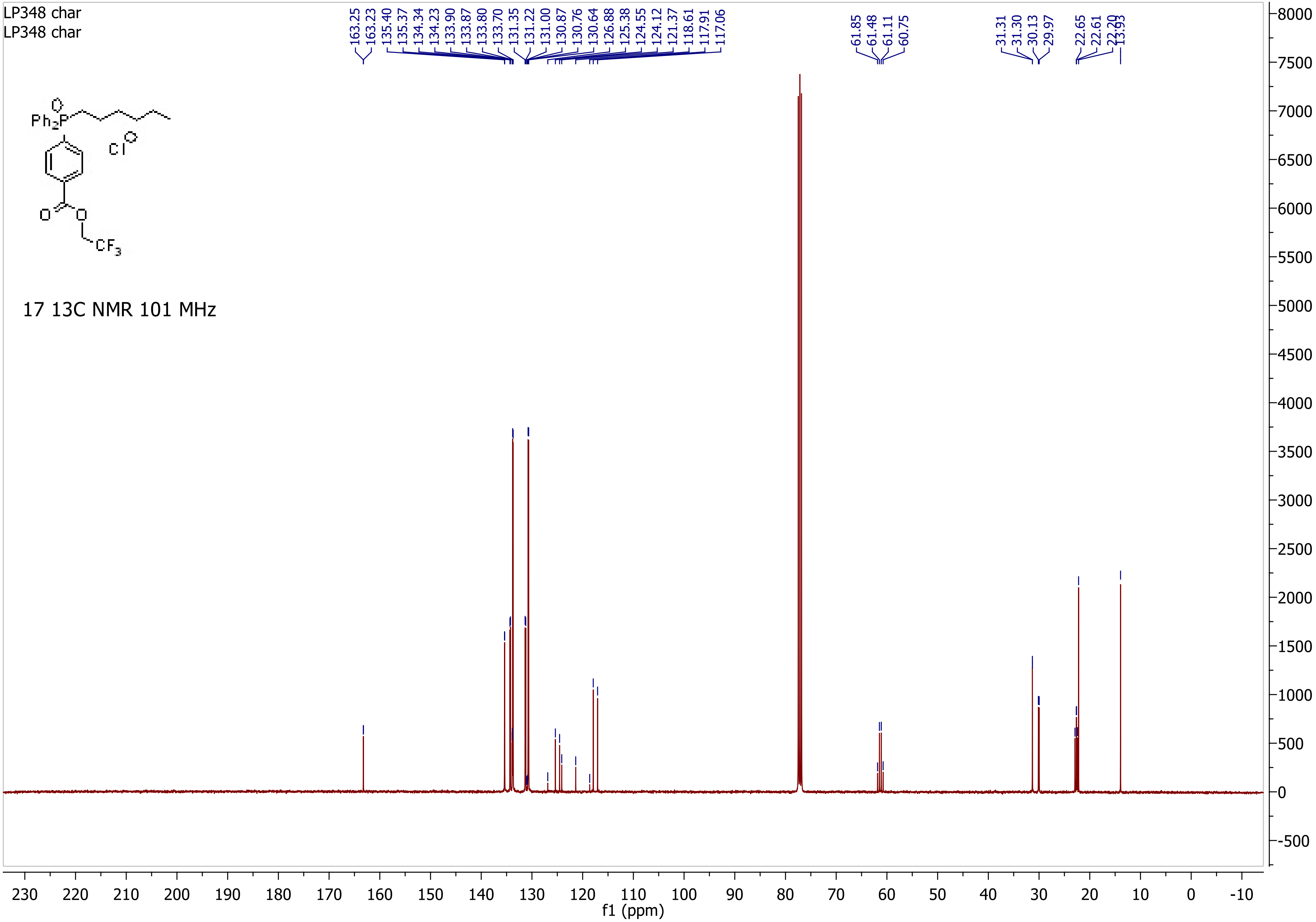
LP348 char
LP348 char
17 13C NMR 101 MHz
Ph2P
O
Cl
O
O
O
CF3
163.25
163.23
135.40
135.37
134.34
134.23
133.90
133.87
133.80
133.70
131.35
131.22
131.00
130.87
130.76
130.64
126.88
125.38
124.55
124.12
121.37
118.61
117.91
117.06
61.85
61.48
61.11
60.75
31.31
31.30
30.13
29.97
22.65
22.61
22.20
13.93
f1 (ppm)

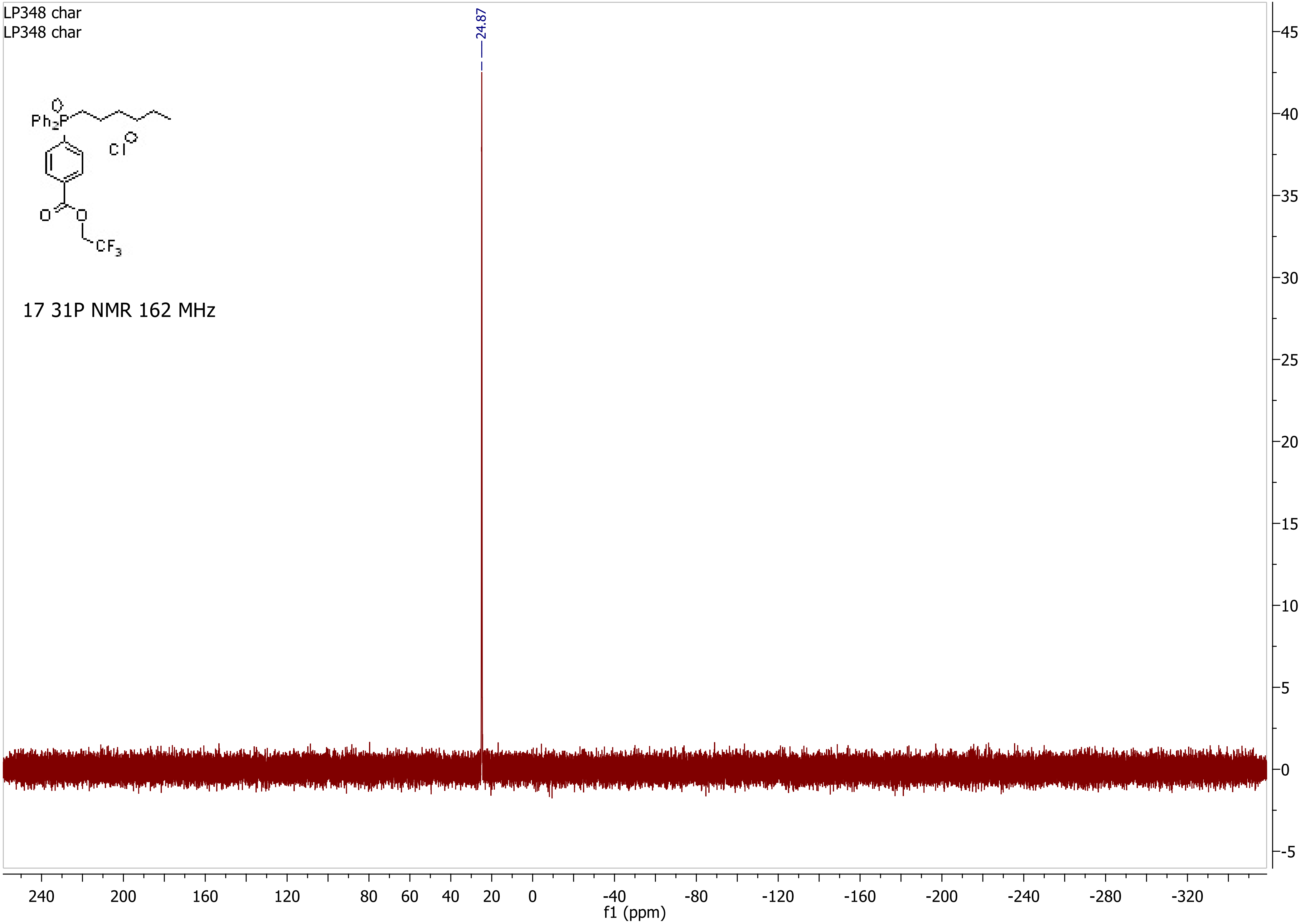
LP348 char
LP348 char
24.87
Ph2P
O
Cl
O
O
CF3
17 31P NMR 162 MHz
f1 (ppm)

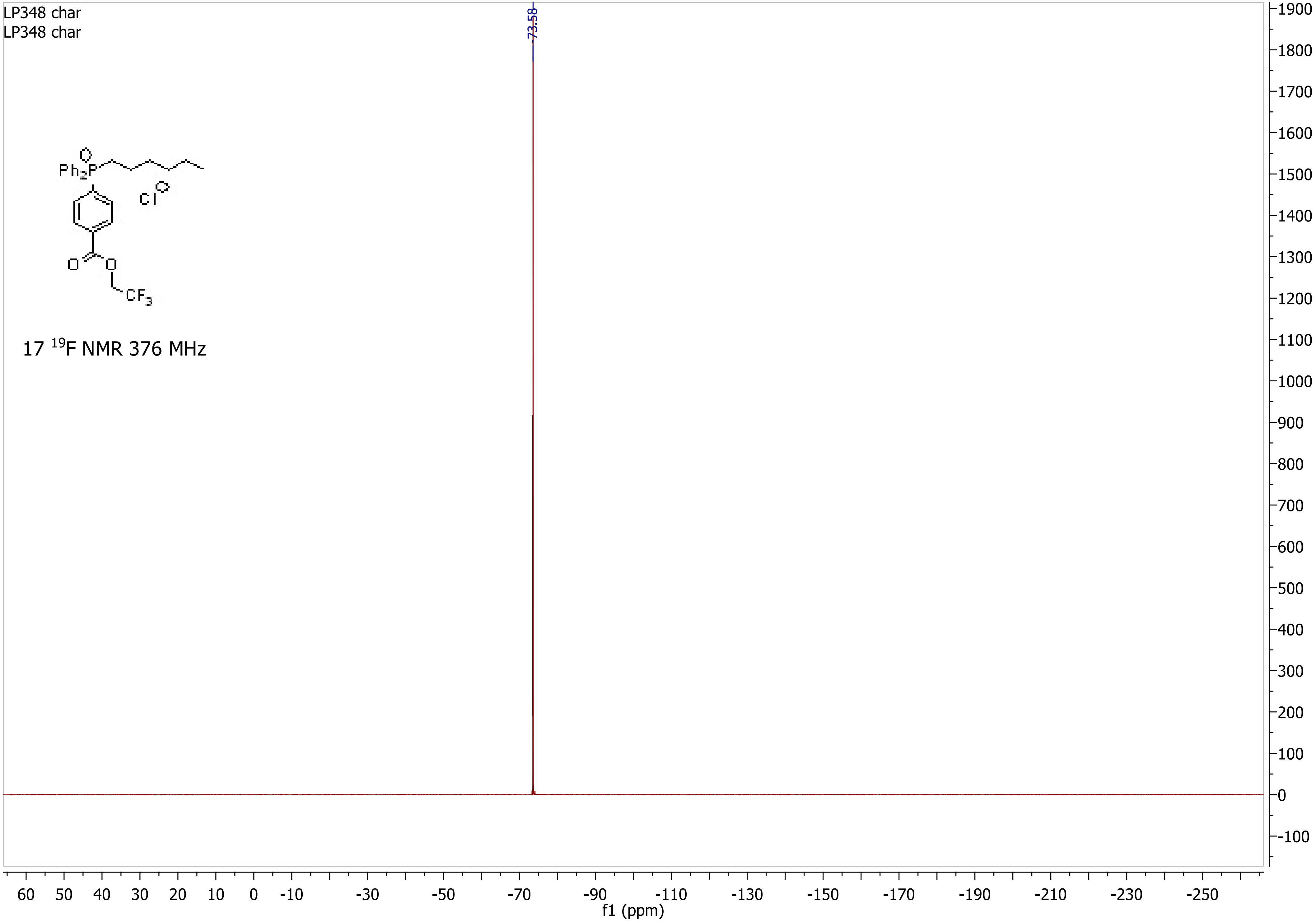

LP348 char
LP348 char
73.58
Ph2P
O
Cl
O
O
CF3
17 19F NMR 376 MHz
1900
1800
1700
1600
1500
1400
1300
1200
1100
1000
900
800
700
600
500
400
300
200
100
0
-100
60 50 40 30 20 10 0 -10 -30 -50 -70 -90 -110 -130 -150 -170 -190 -210 -230 -250
f1 (ppm)

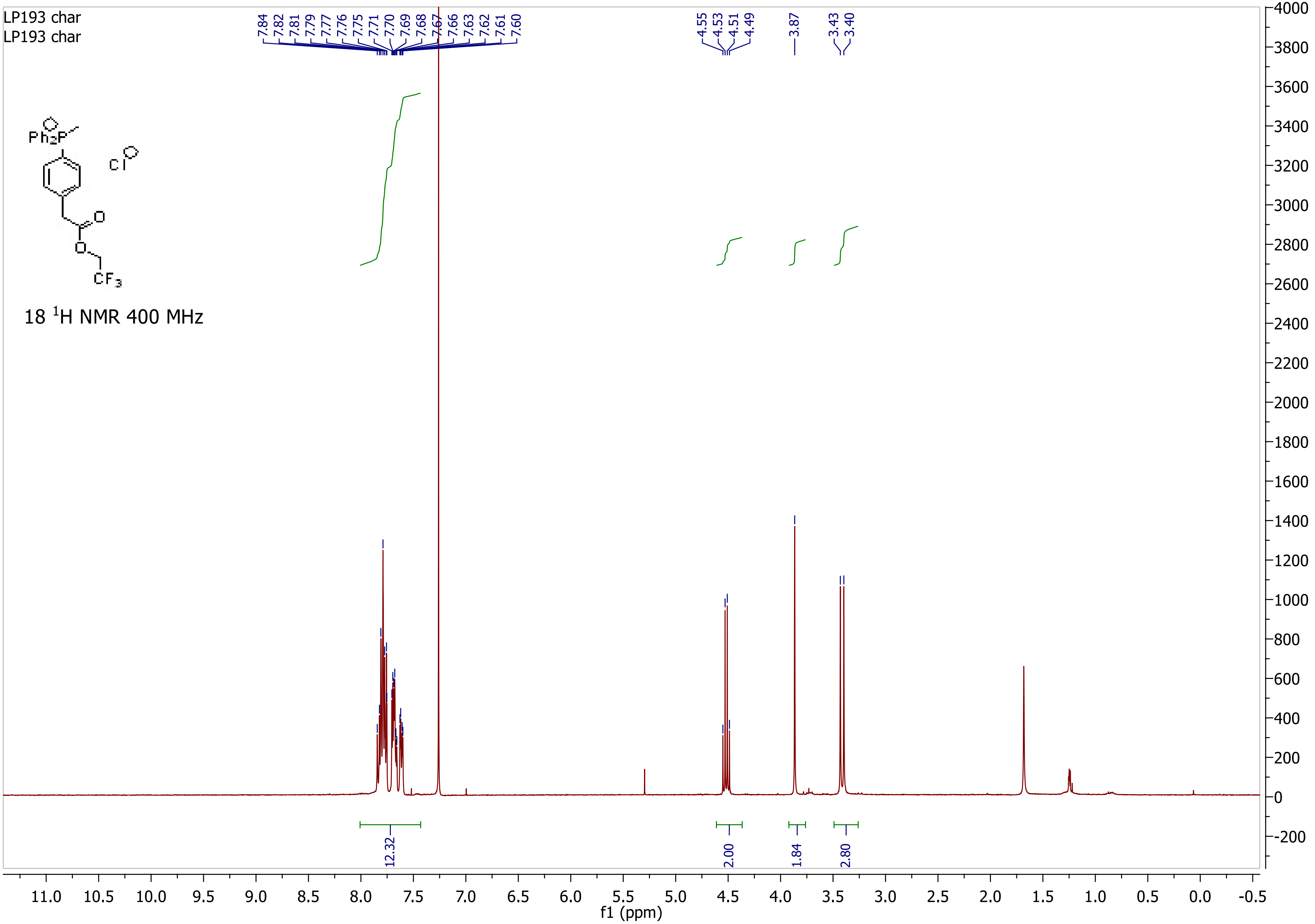
LP193 char
LP193 char
18 ¹H NMR 400 MHz
Ph2P
Cl
O
O
CF3
7.84 7.82 7.81 7.79 7.77 7.76 7.75 7.71 7.70 7.69 7.68 7.67 7.66 7.63 7.62 7.61 7.60
4.55 4.53 4.51 4.49
3.87
3.43 3.40
12.32
2.00
1.84
2.80
f1 (ppm)

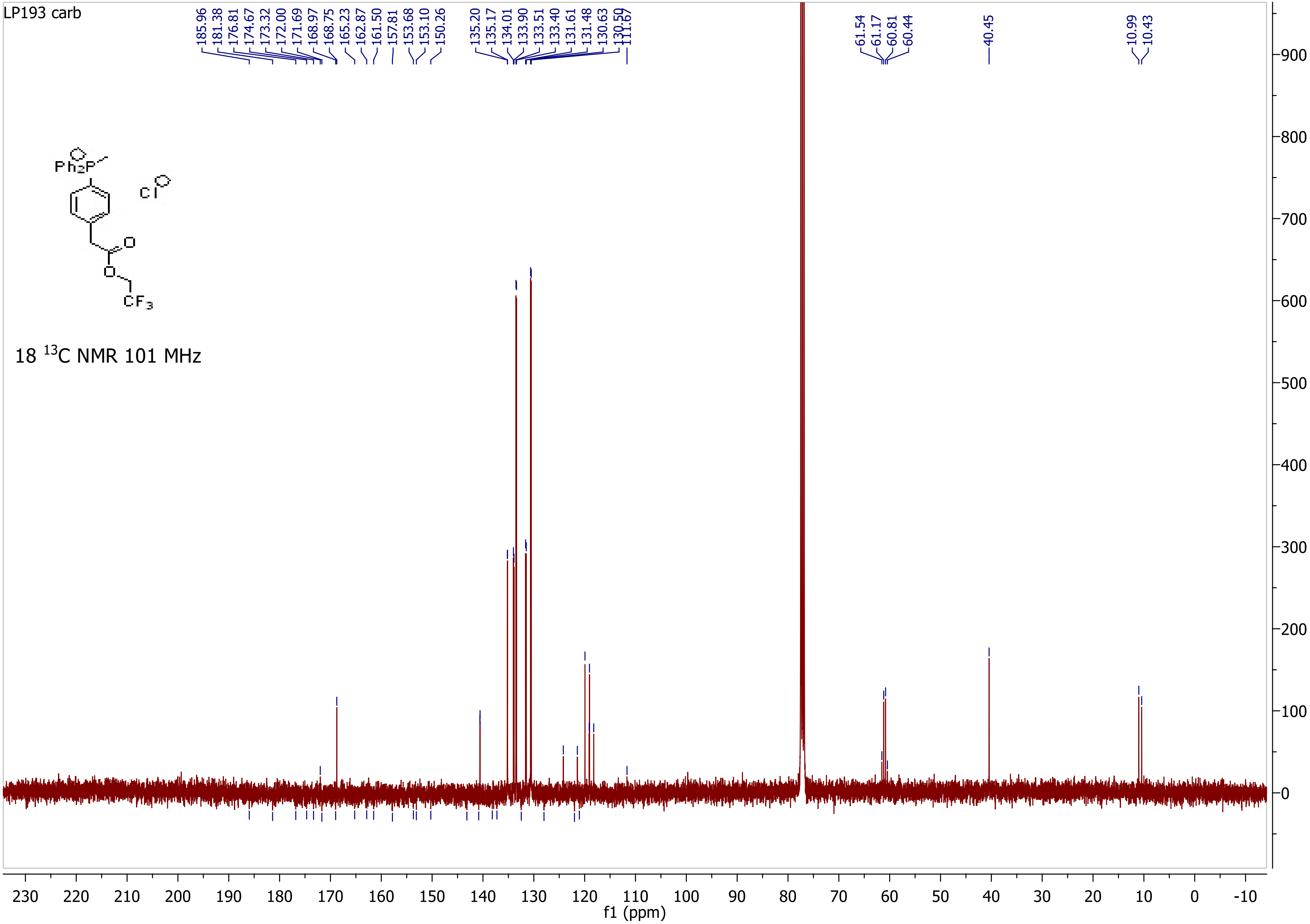

LP193 carb
185.96
181.38
176.81
174.67
173.32
172.00
171.69
168.97
168.75
165.23
162.87
161.50
157.81
153.68
153.10
150.26
135.20
135.17
134.01
133.90
133.51
133.40
131.61
131.48
130.63
130.59
111.67
61.54
61.17
60.81
60.44
40.45
10.99
10.43
Ph2P
Cl
O
CF3
18 13C NMR 101 MHz
900
800
700
600
500
400
300
200
100
0
230 220 210 200 190 180 170 160 150 140 130 120 110 100 90 80 70 60 50 40 30 20 10 0 -10
f1 (ppm)

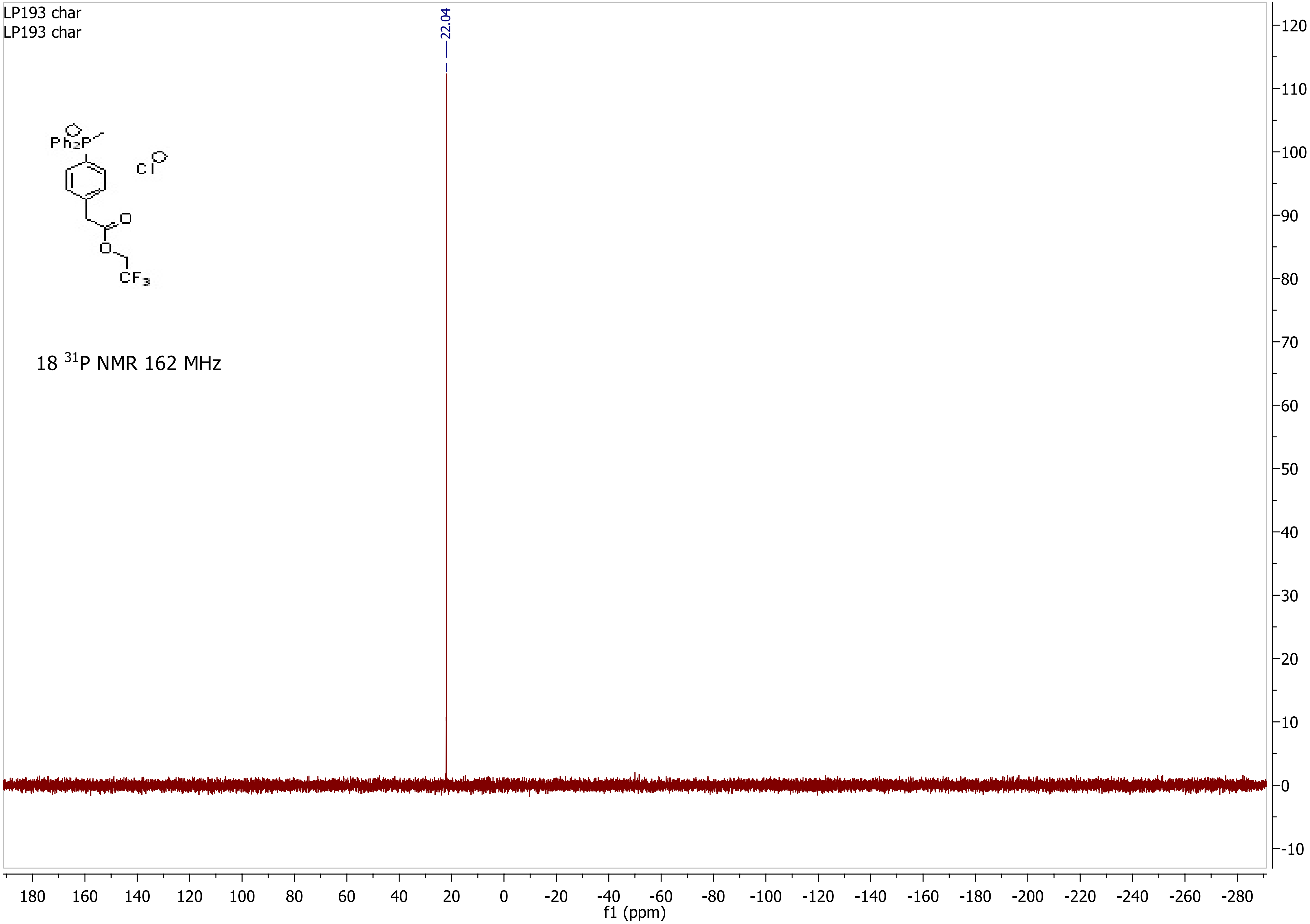

LP193 char
LP193 char
22.04
Ph2P
Cl
O
CF3
18 31P NMR 162 MHz
f1 (ppm)

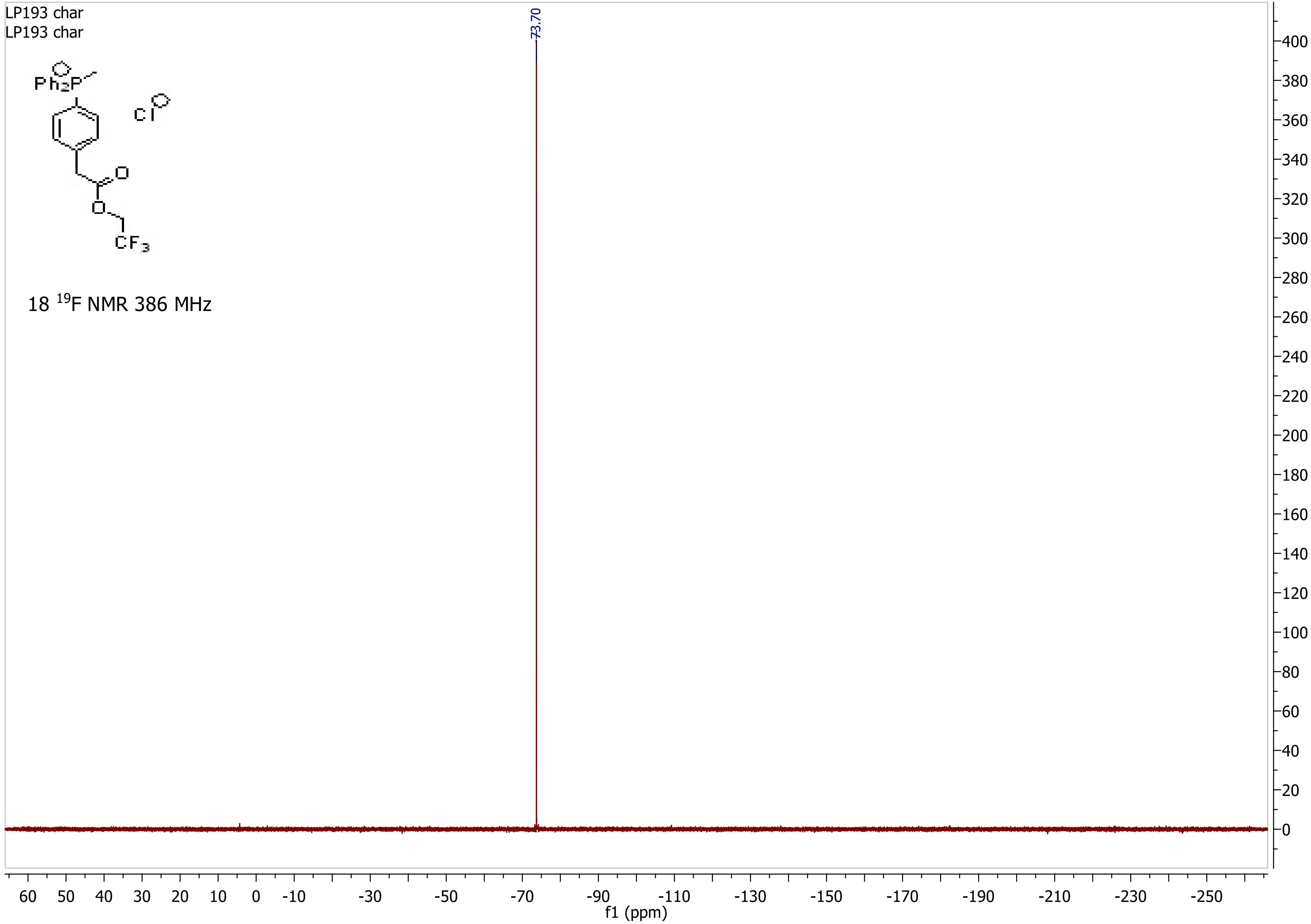
LP193 char
LP193 char
Ph2P
Cl
O
O
CF3
18 19F NMR 386 MHz
-73.70
f1 (ppm)
60 50 40 30 20 10 0 -10 -30 -50 -70 -90 -110 -130 -150 -170 -190 -210 -230 -250
400 380 360 340 320 300 280 260 240 220 200 180 160 140 120 100 80 60 40 20 0

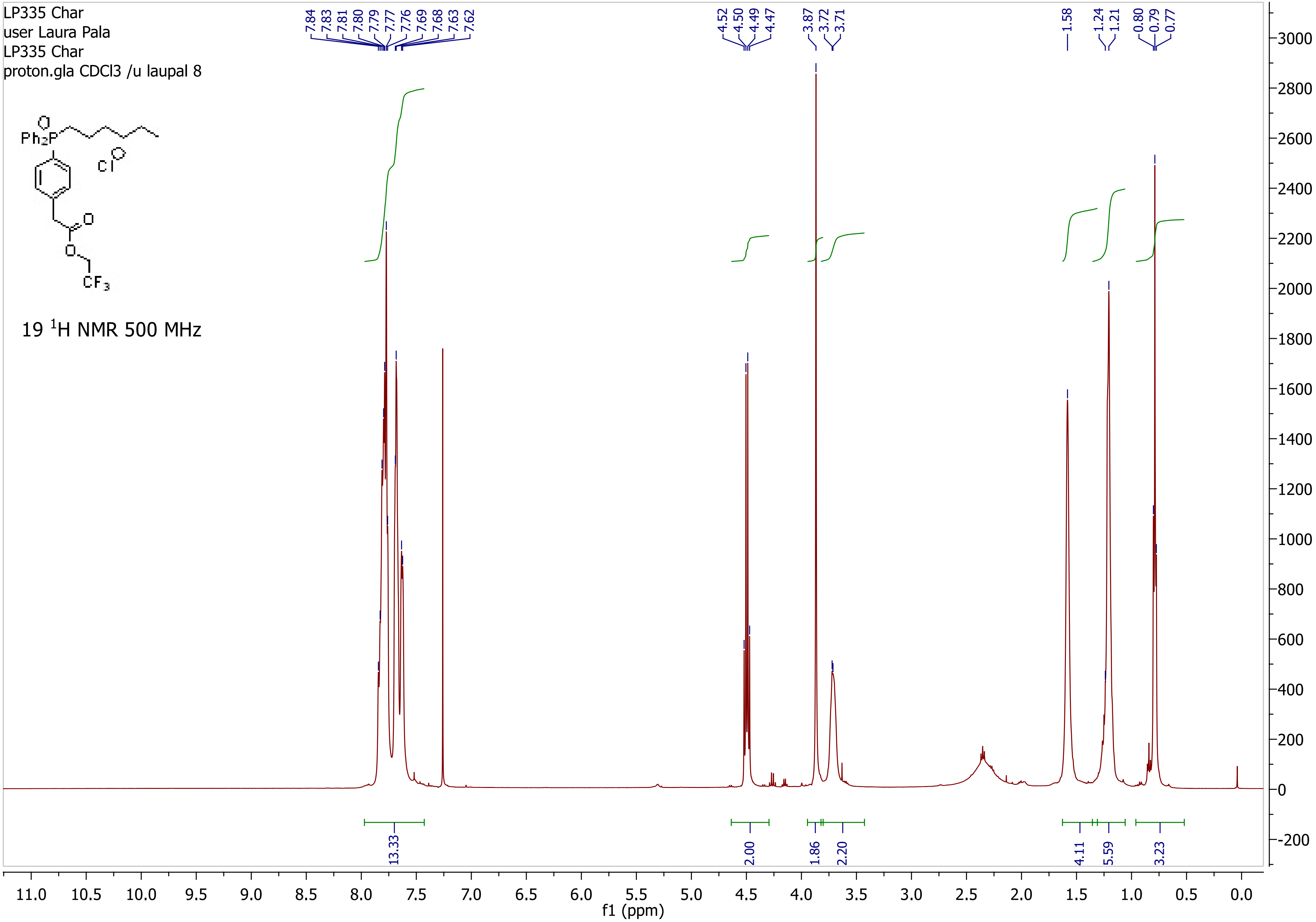

LP335 Char
user Laura Pala
LP335 Char
proton.gla CDCl3 /u laupal 8
19 ¹H NMR 500 MHz
7.84 7.83 7.81 7.80 7.79 7.77 7.76 7.69 7.68 7.63 7.62
4.52 4.50 4.49 4.47
3.87 3.72 3.71
1.58
1.24 1.21
0.80 0.79 0.77
13.33
2.00
1.86
2.20
4.11
5.59
3.23
f1 (ppm)

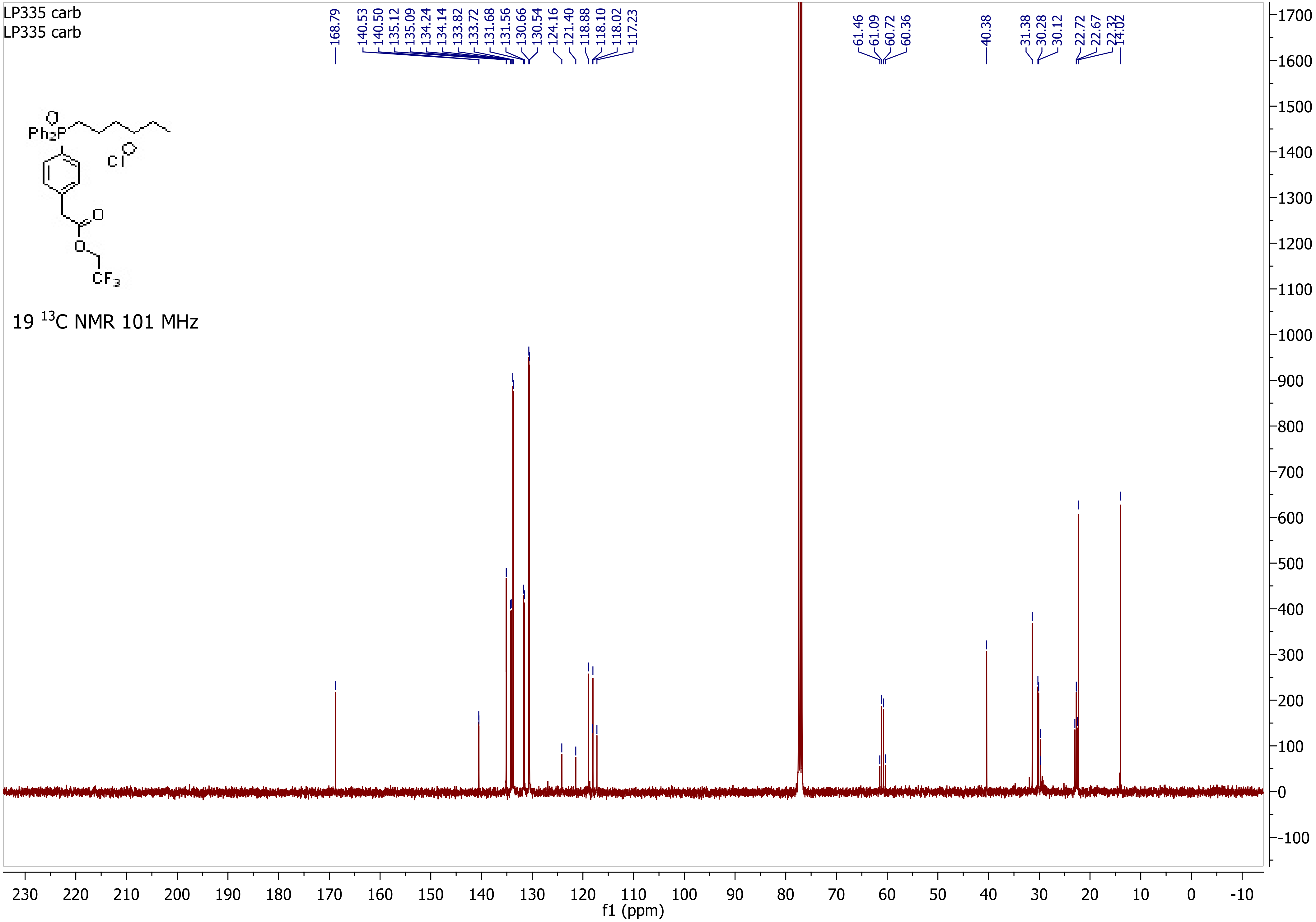

LP335 carb
LP335 carb
168.79
140.53
140.50
135.12
135.09
134.24
134.14
133.82
133.72
131.68
131.56
130.66
130.54
124.16
121.40
118.88
118.10
118.02
117.23
61.46
61.09
60.72
60.36
40.38
31.38
30.28
30.12
22.72
22.67
22.32
14.02
Ph2P
O
ClO
O
O
CF3
19 13C NMR 101 MHz
f1 (ppm)

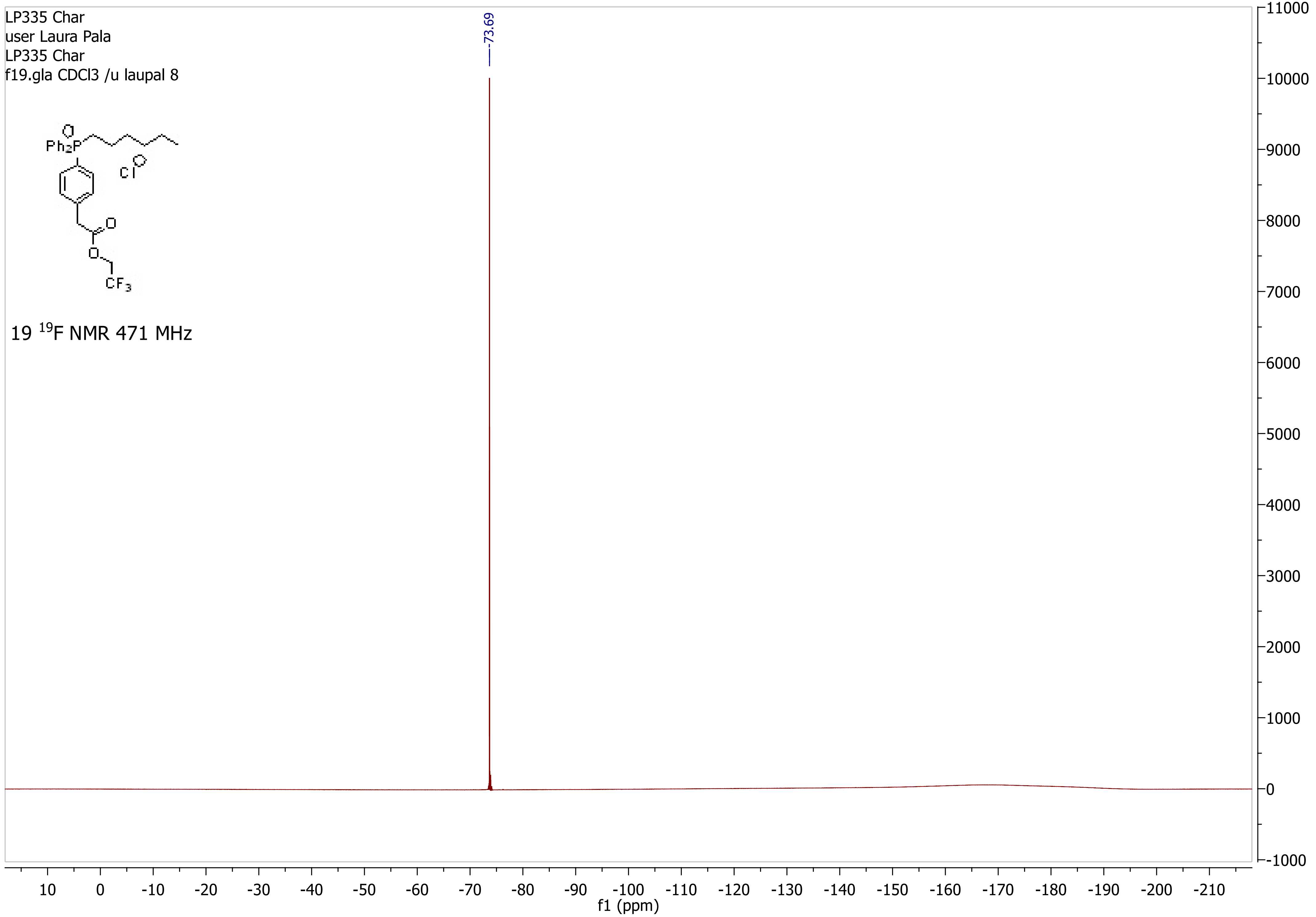

LP335 Char
user Laura Pala
LP335 Char
f19.gla CDCl3 /u laupal 8
-73.69
19 19F NMR 471 MHz
f1 (ppm)

LP335 Char
user Laura Pala
LP335 Char
p31.gla CDCl3 /u laupal 8

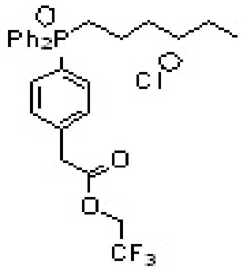

19 ^{31}P NMR 202 MHz

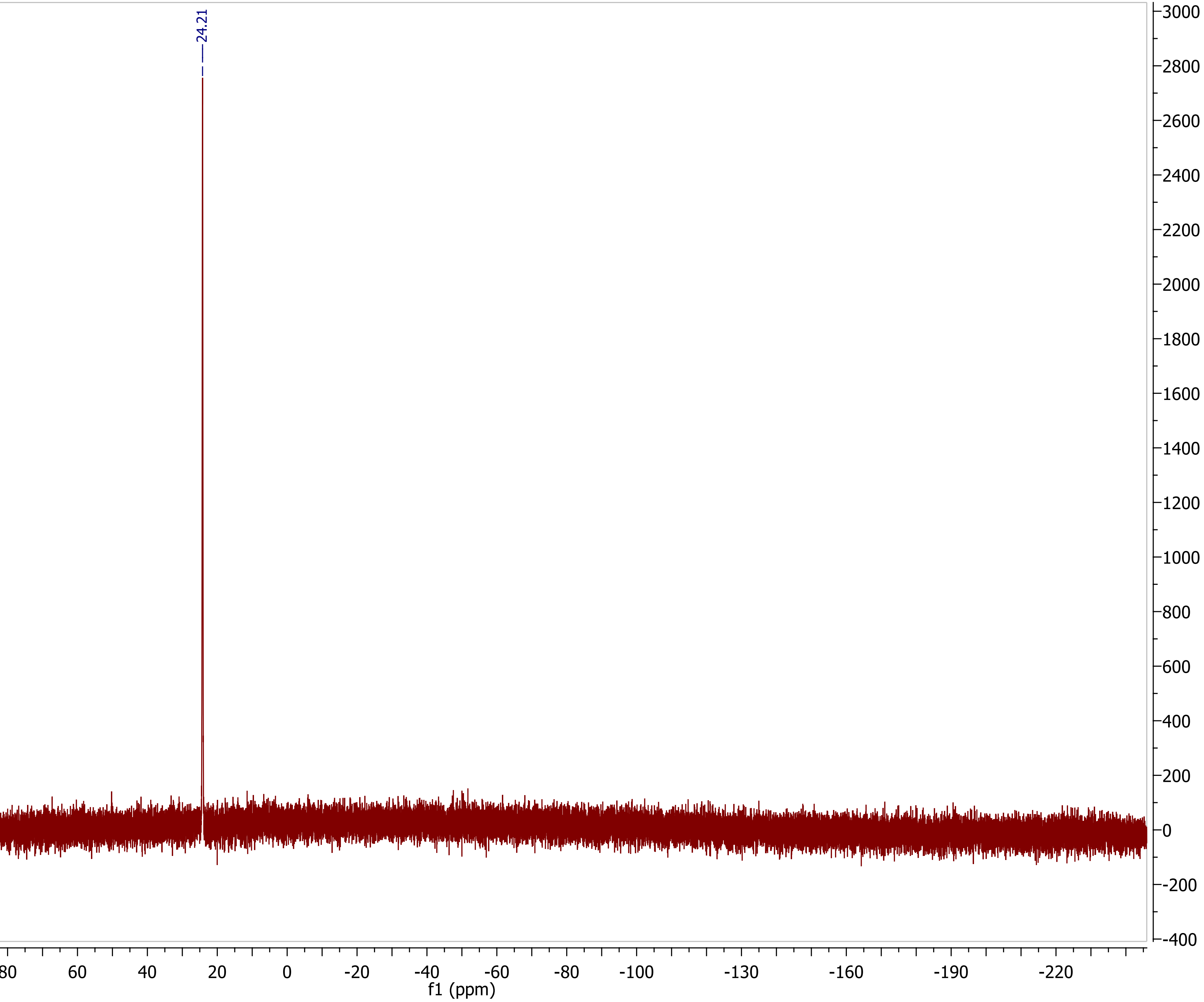

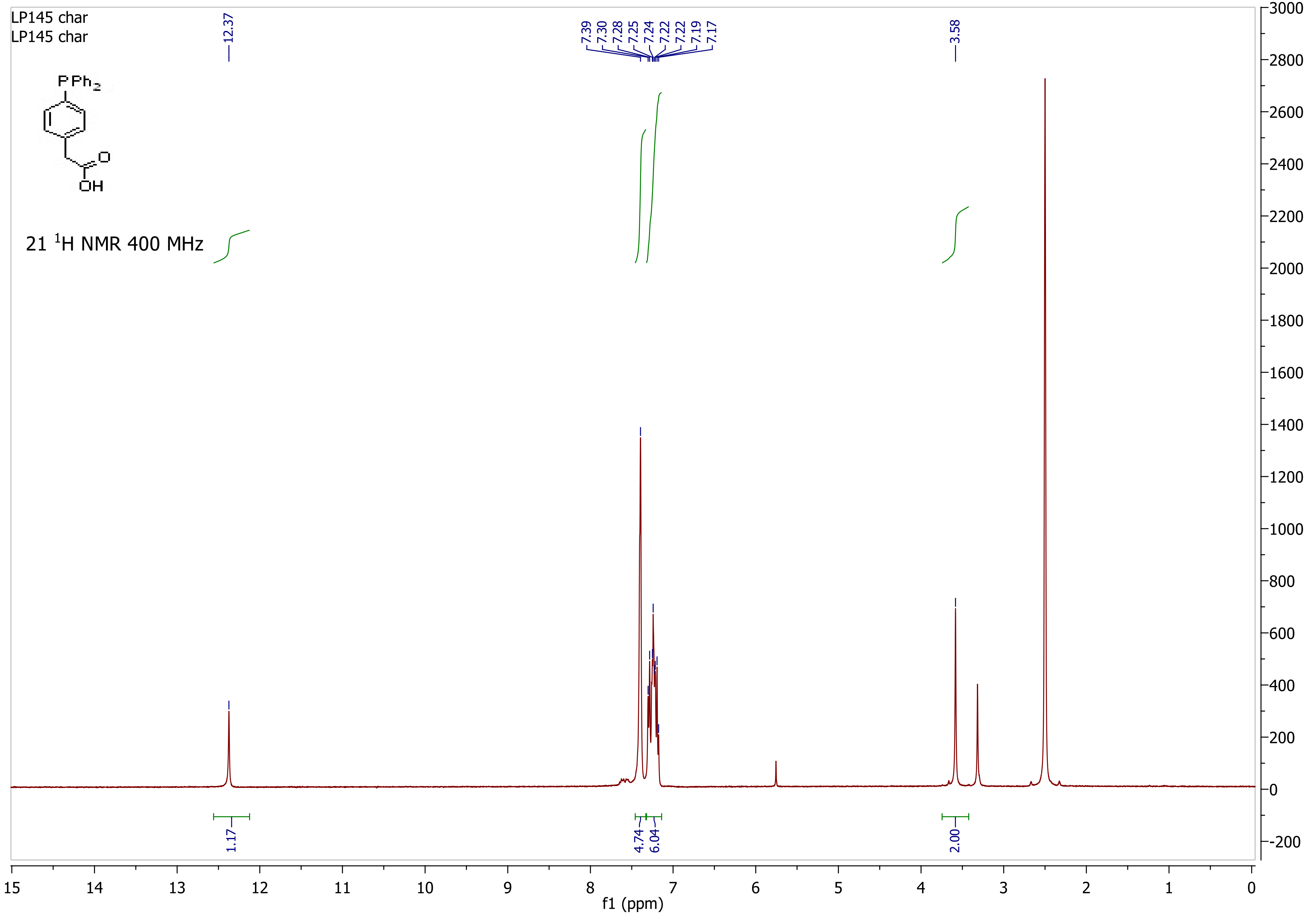

LP145 char
LP145 char
PPh2
O
OH
21 1H NMR 400 MHz
12.37
7.39
7.30
7.28
7.25
7.24
7.22
7.22
7.19
7.17
3.58
1.17
4.74
6.04
2.00
f1 (ppm)

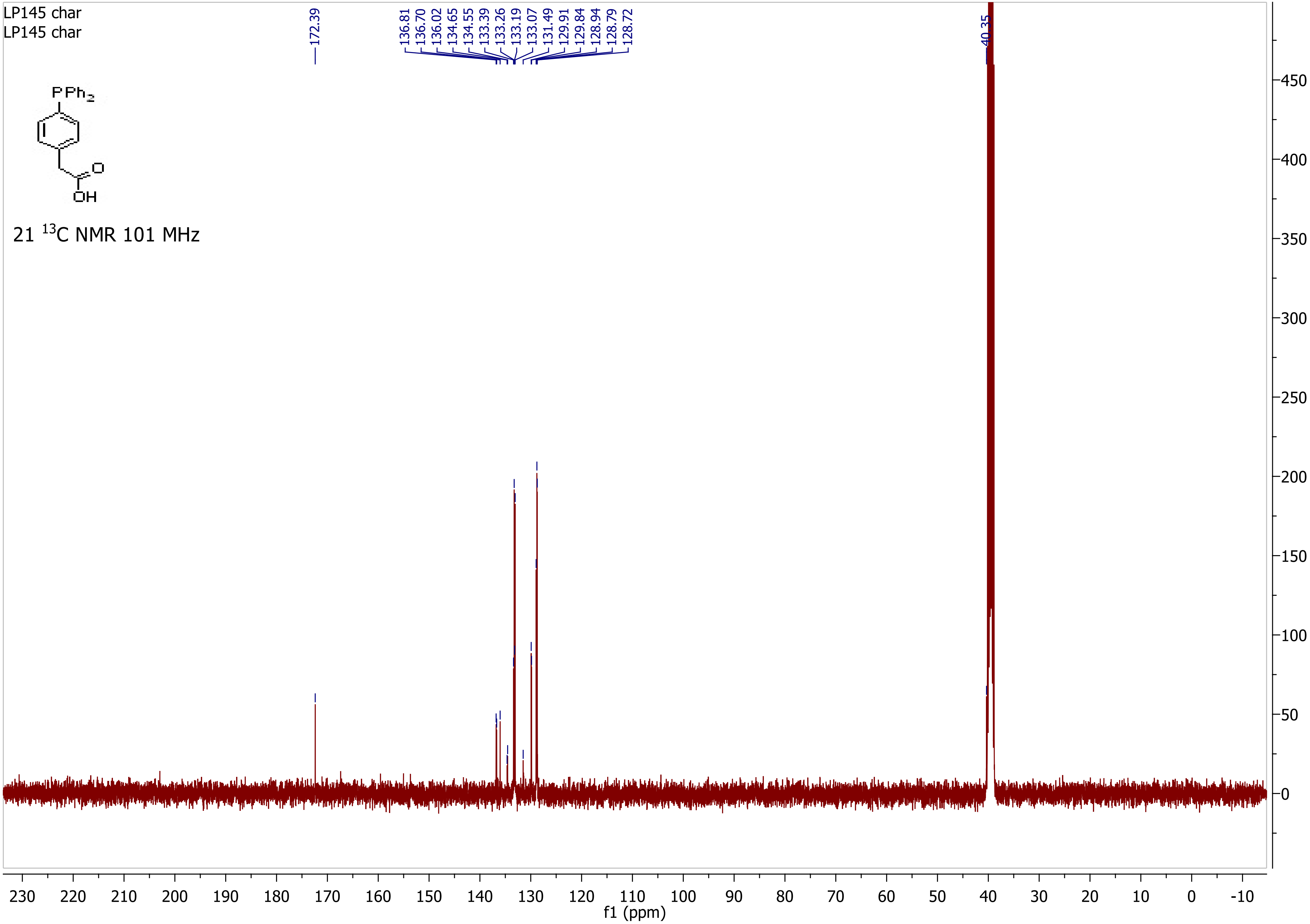
LP145 char
LP145 char
PPh2
O
OH
21 13C NMR 101 MHz
172.39
136.81
136.70
136.02
134.65
134.55
133.39
133.26
133.19
133.07
131.49
129.91
129.84
128.94
128.79
128.72
40.35
f1 (ppm)
230
220
210
200
190
180
170
160
150
140
130
120
110
100
90
80
70
60
50
40
30
20
10
0
-10
450
400
350
300
250
200
150
100
50
0

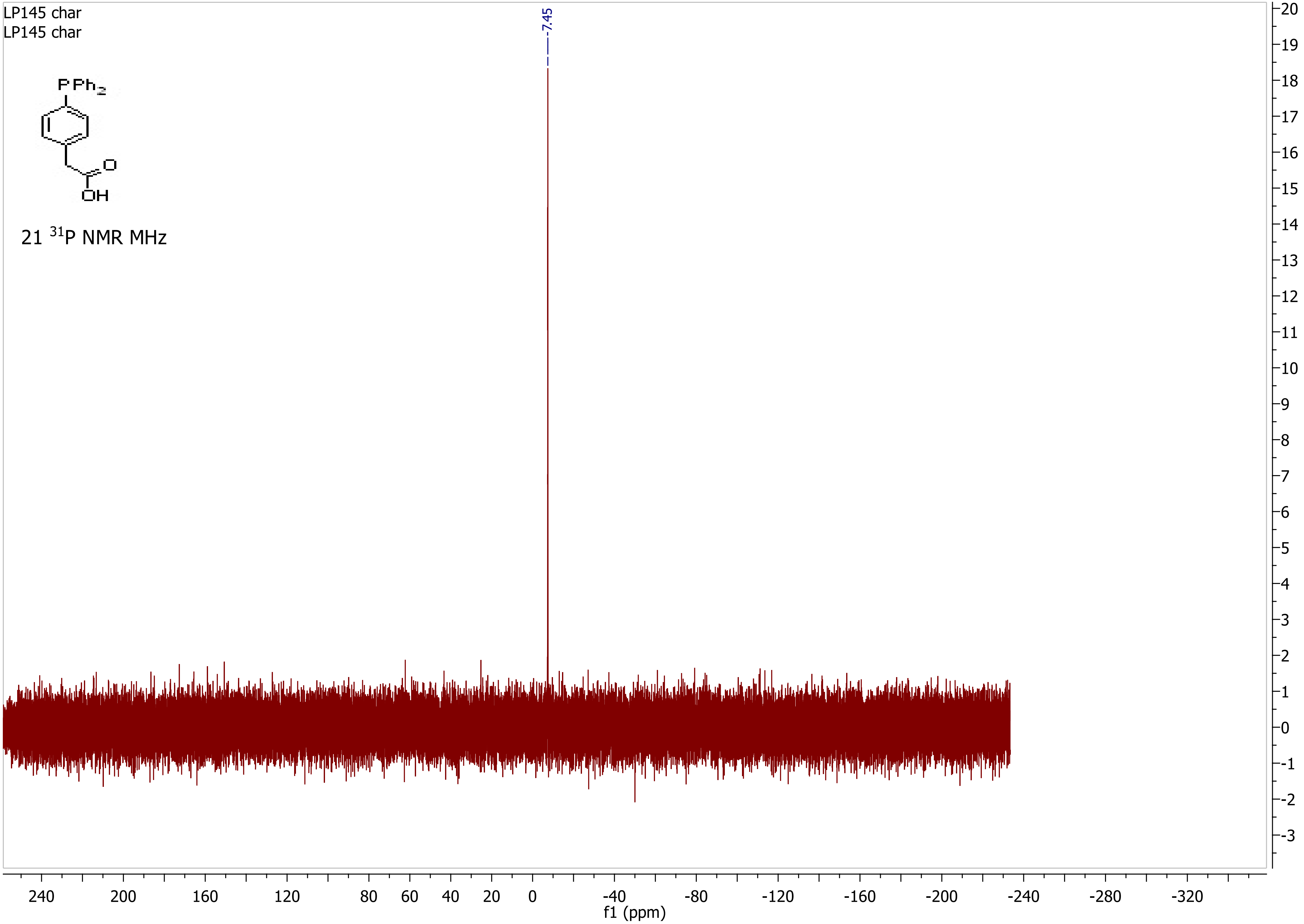
LP145 char
LP145 char
PPh2
O
OH
21 31P NMR MHz
-7.45
f1 (ppm)

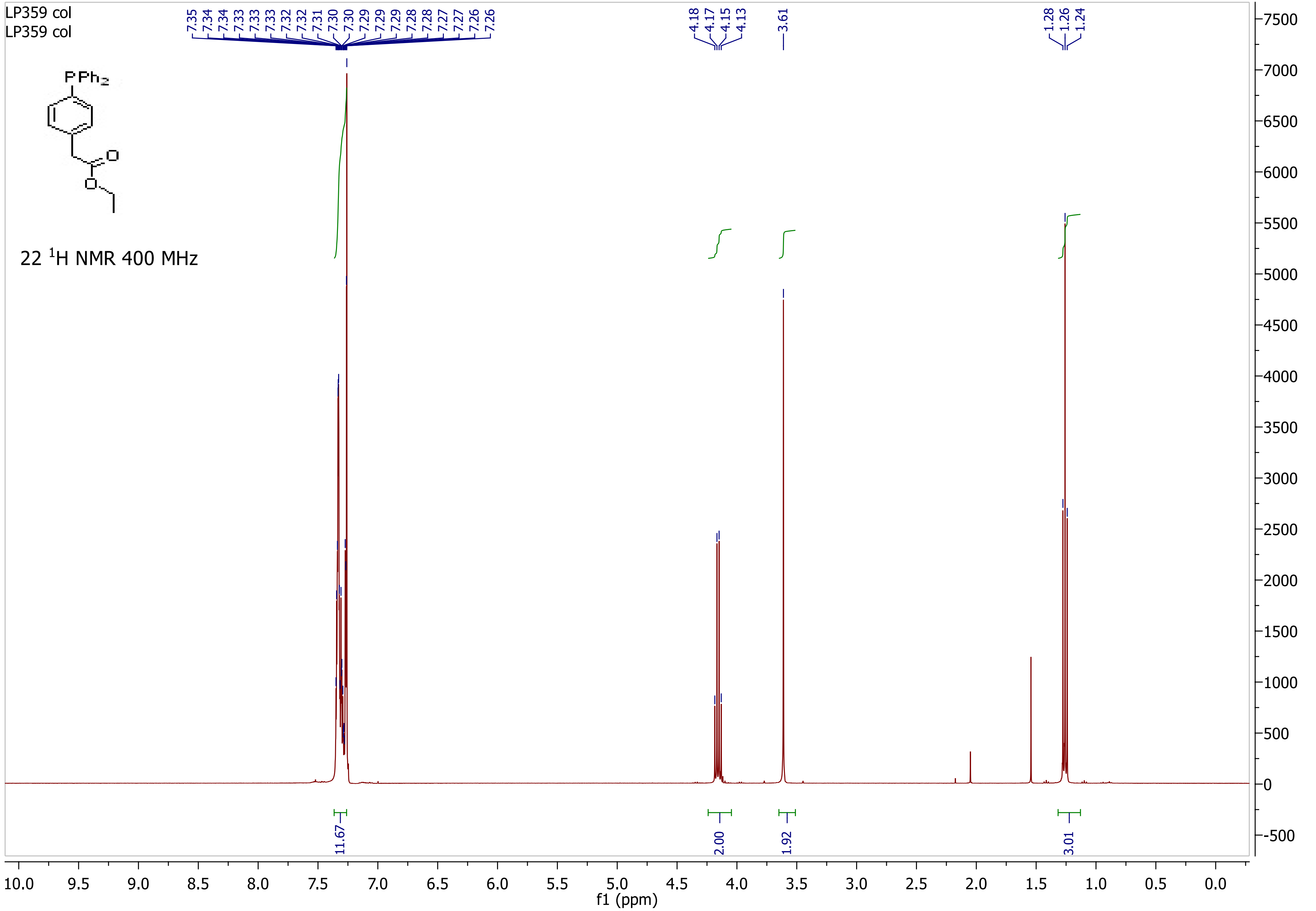

LP359 col
LP359 col
PPh2
O
O
22 1H NMR 400 MHz
7.35
7.34
7.34
7.33
7.33
7.33
7.32
7.32
7.31
7.30
7.30
7.29
7.29
7.29
7.28
7.28
7.27
7.27
7.26
7.26
4.18
4.17
4.15
4.13
3.61
1.28
1.26
1.24
11.67
2.00
1.92
3.01
f1 (ppm)

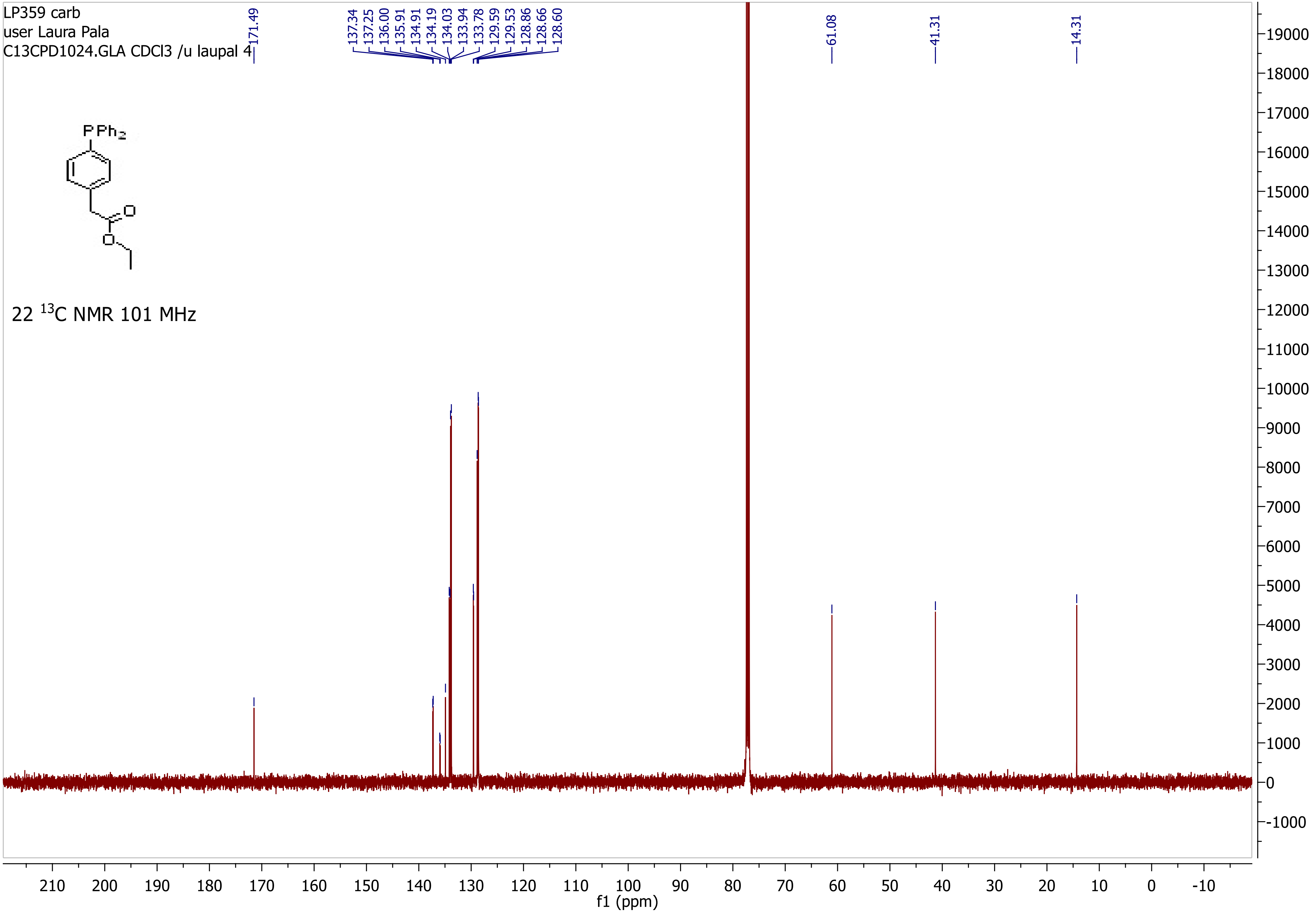
LP359 carb
user Laura Pala
C13CPD1024.GLA CDCl3 /u laupal 4
171.49
137.34
137.25
136.00
135.91
134.91
134.19
134.03
133.94
133.78
129.59
129.53
128.86
128.66
128.60
61.08
41.31
14.31
PPh2
O
O
22 13C NMR 101 MHz
f1 (ppm)

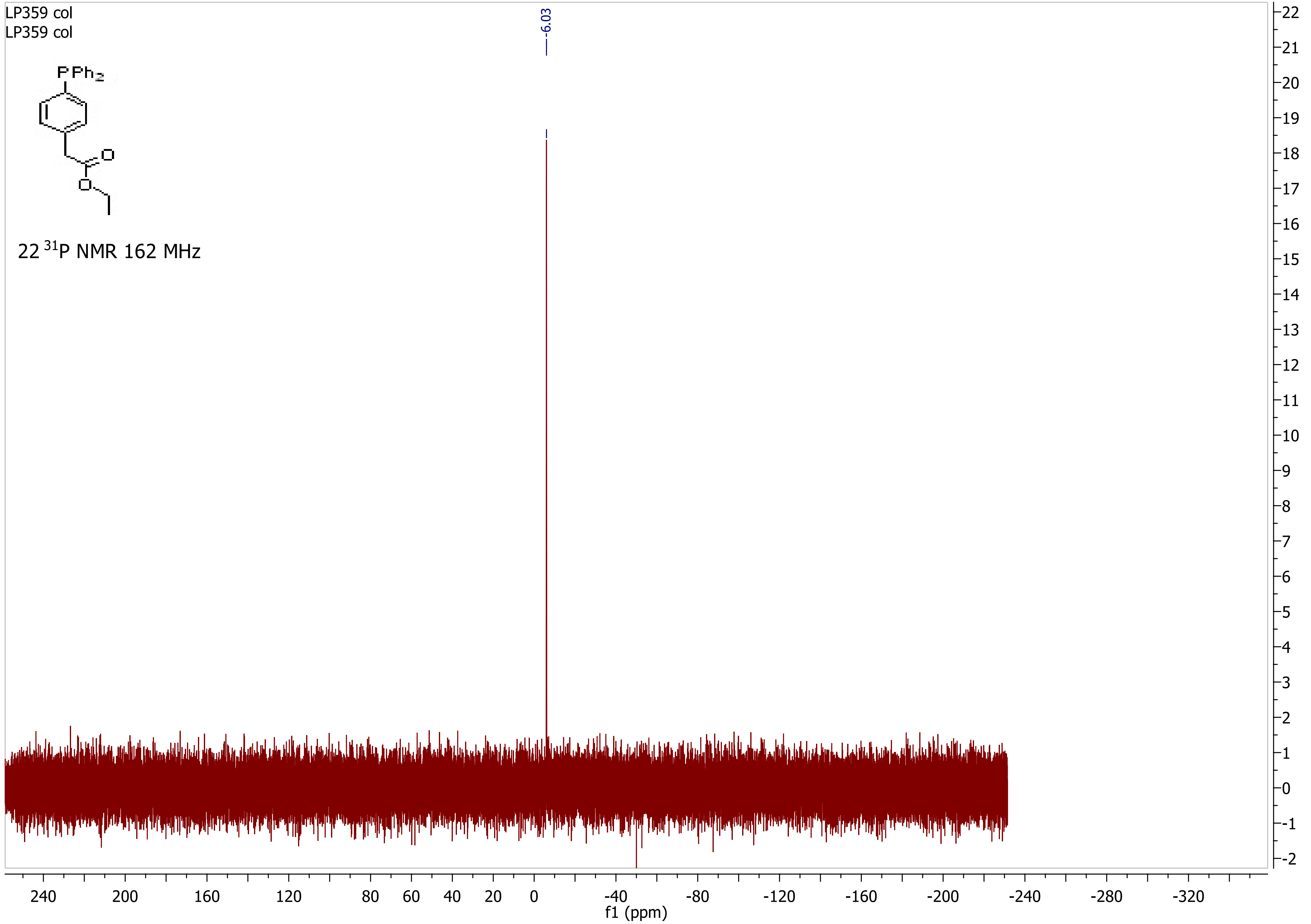
LP359 col
LP359 col
PPh2
O
O
22 31P NMR 162 MHz
-6.03
f1 (ppm)

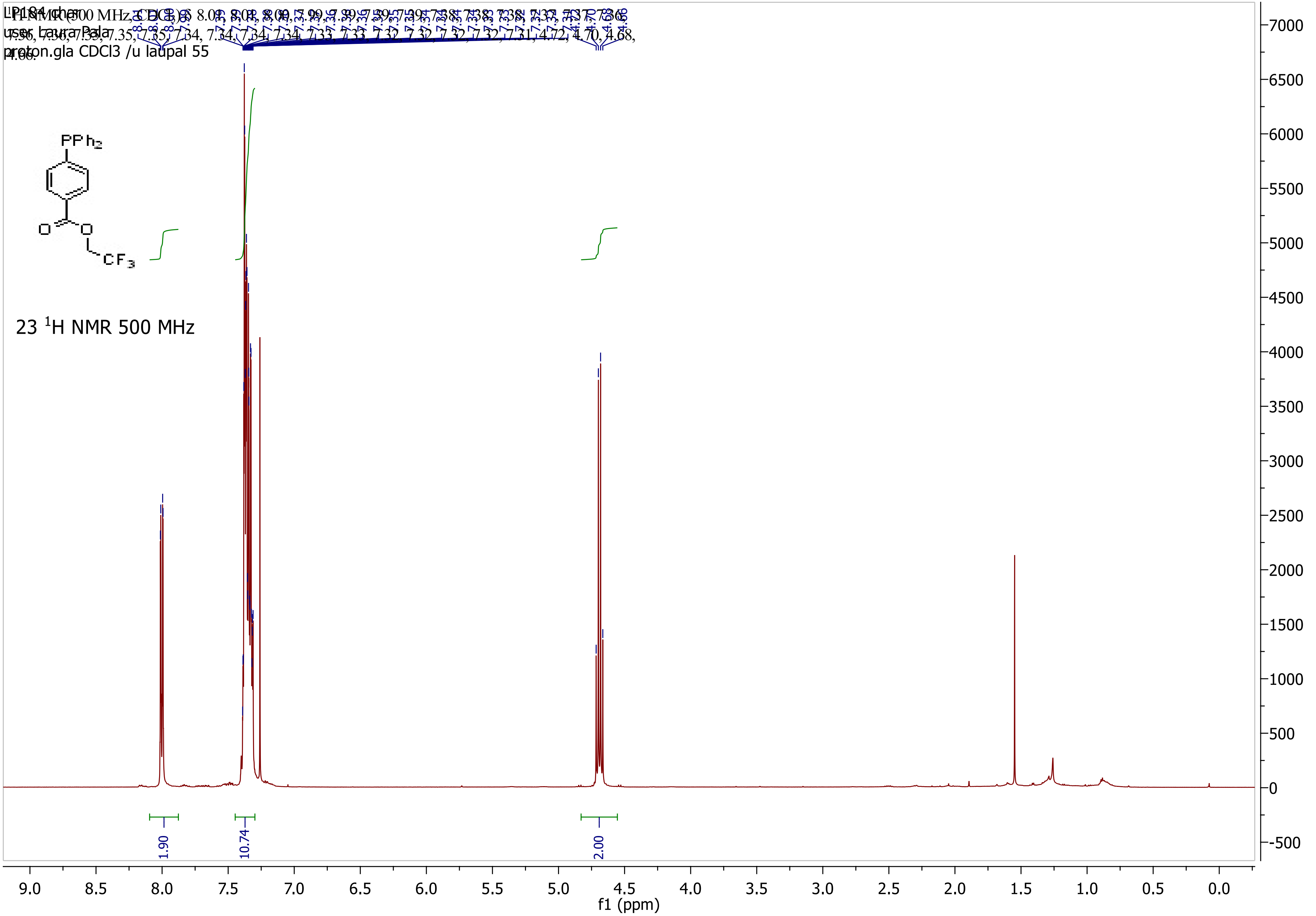

LP184 char
user Laura Pala
proton.gla CDCl3 /u laupal 55
PPh2
O
O
CF3
23 1H NMR 500 MHz
1.90
10.74
2.00
f1 (ppm)

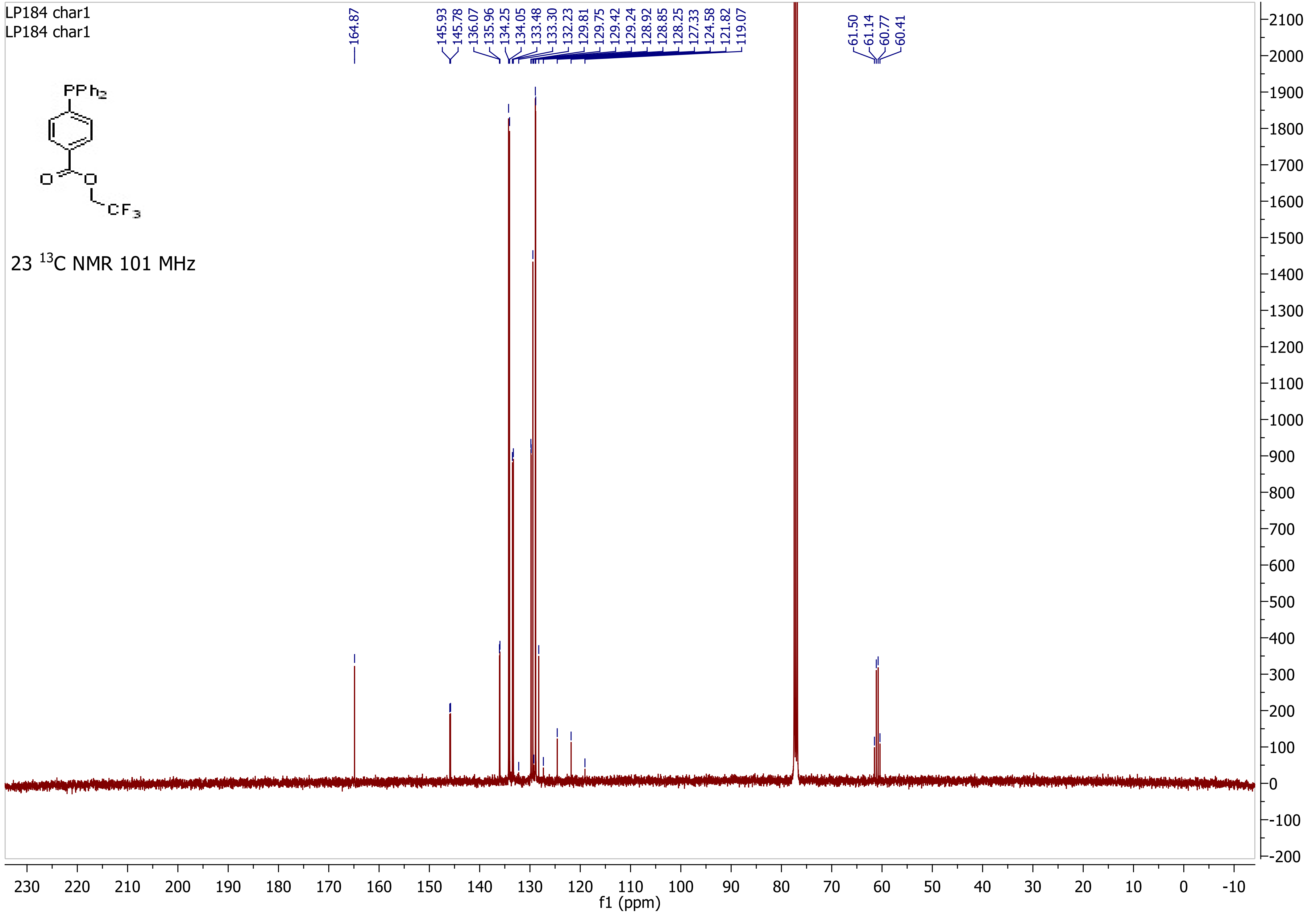
LP184 char1
LP184 char1
PPh2
O
O
CF3
23 13C NMR 101 MHz
164.87
145.93
145.78
136.07
135.96
134.25
134.05
133.48
133.30
132.23
129.81
129.75
129.42
129.24
128.92
128.85
128.25
127.33
124.58
121.82
119.07
61.50
61.14
60.77
60.41
f1 (ppm)

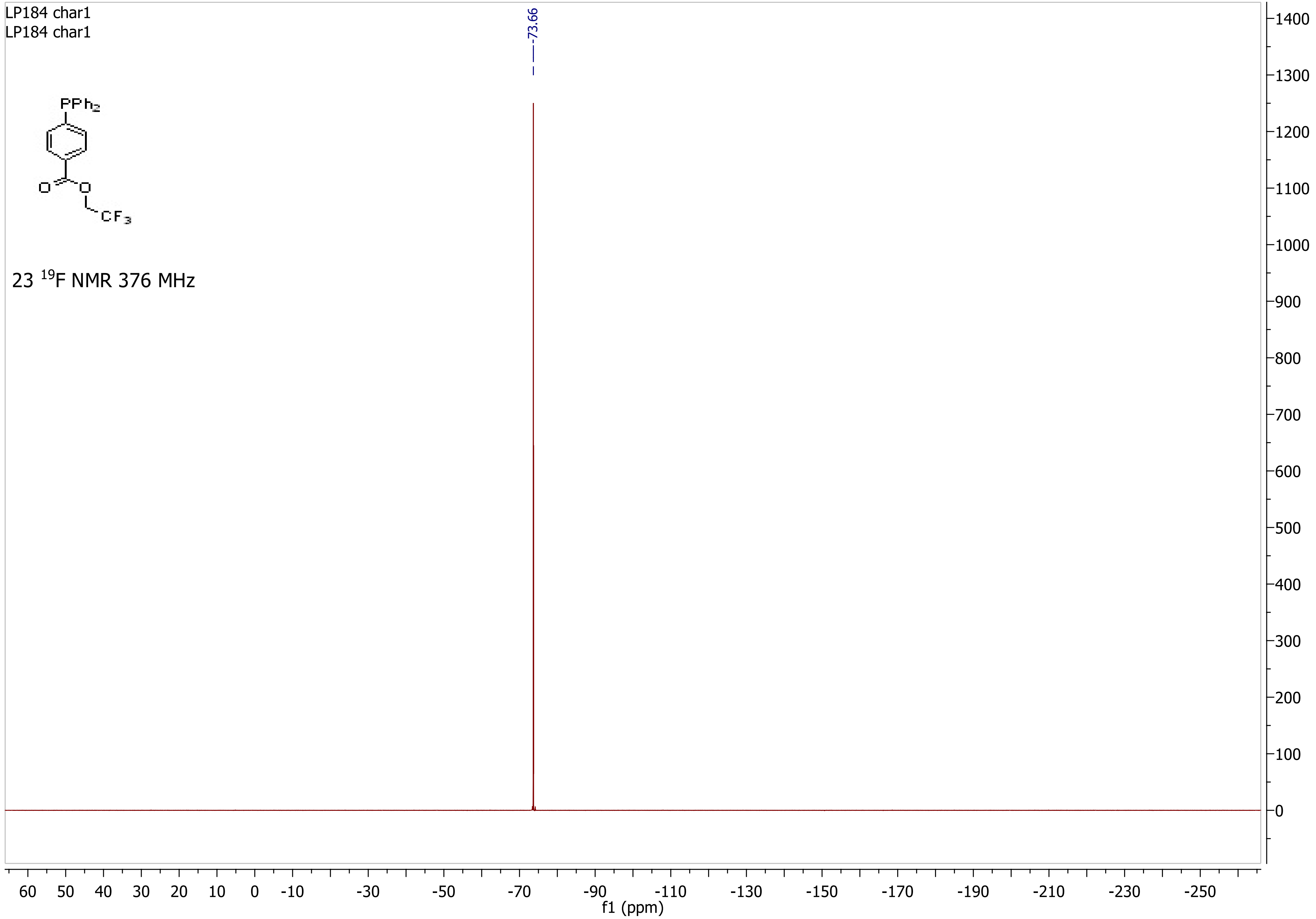
LP184 char1
LP184 char1
-73.66
PPh2
O
O
CF3
23 19F NMR 376 MHz
1400
1300
1200
1100
1000
900
800
700
600
500
400
300
200
100
0
60 50 40 30 20 10 0 -10 -30 -50 -70 -90 -110 -130 -150 -170 -190 -210 -230 -250
f1 (ppm)

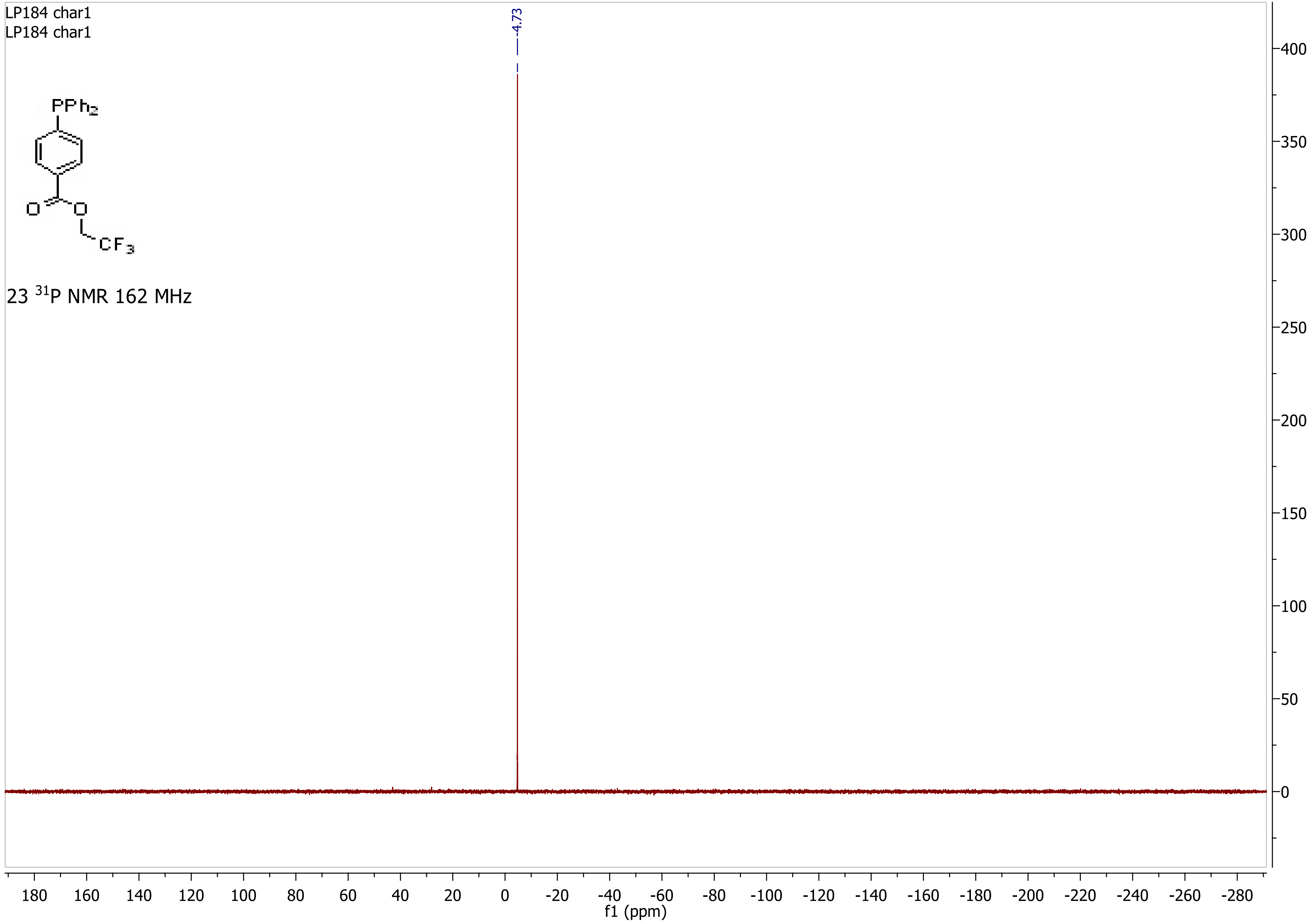
LP184 char1
LP184 char1
PPh2
O
O
CF3
23 31P NMR 162 MHz
-4.73
f1 (ppm)

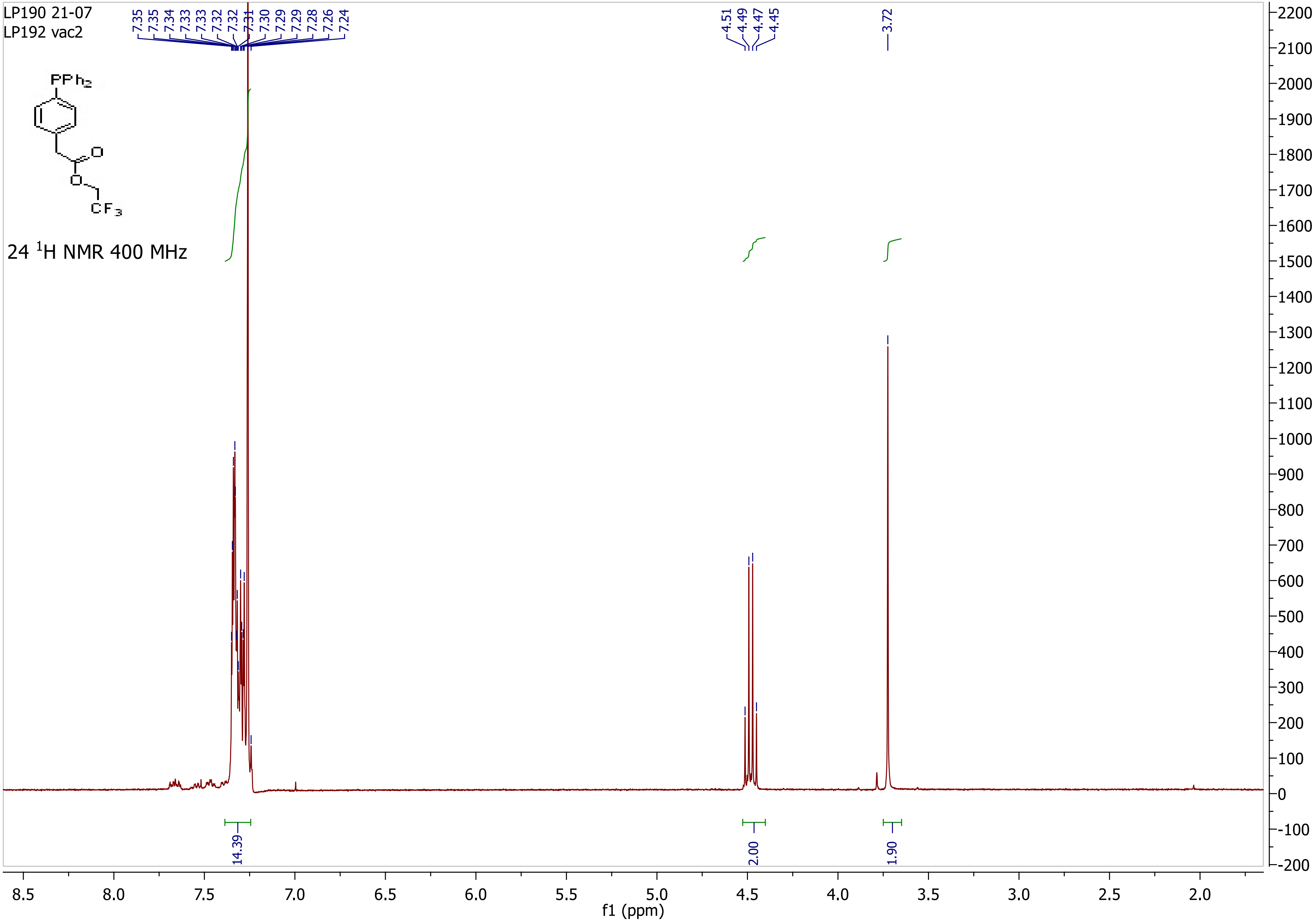

LP190 21-07
LP192 vac2
PPh2
O
O
CF3
24 1H NMR 400 MHz
7.35
7.35
7.34
7.33
7.33
7.32
7.32
7.31
7.30
7.29
7.29
7.28
7.26
7.24
4.51
4.49
4.47
4.45
3.72
14.39
2.00
1.90
f1 (ppm)

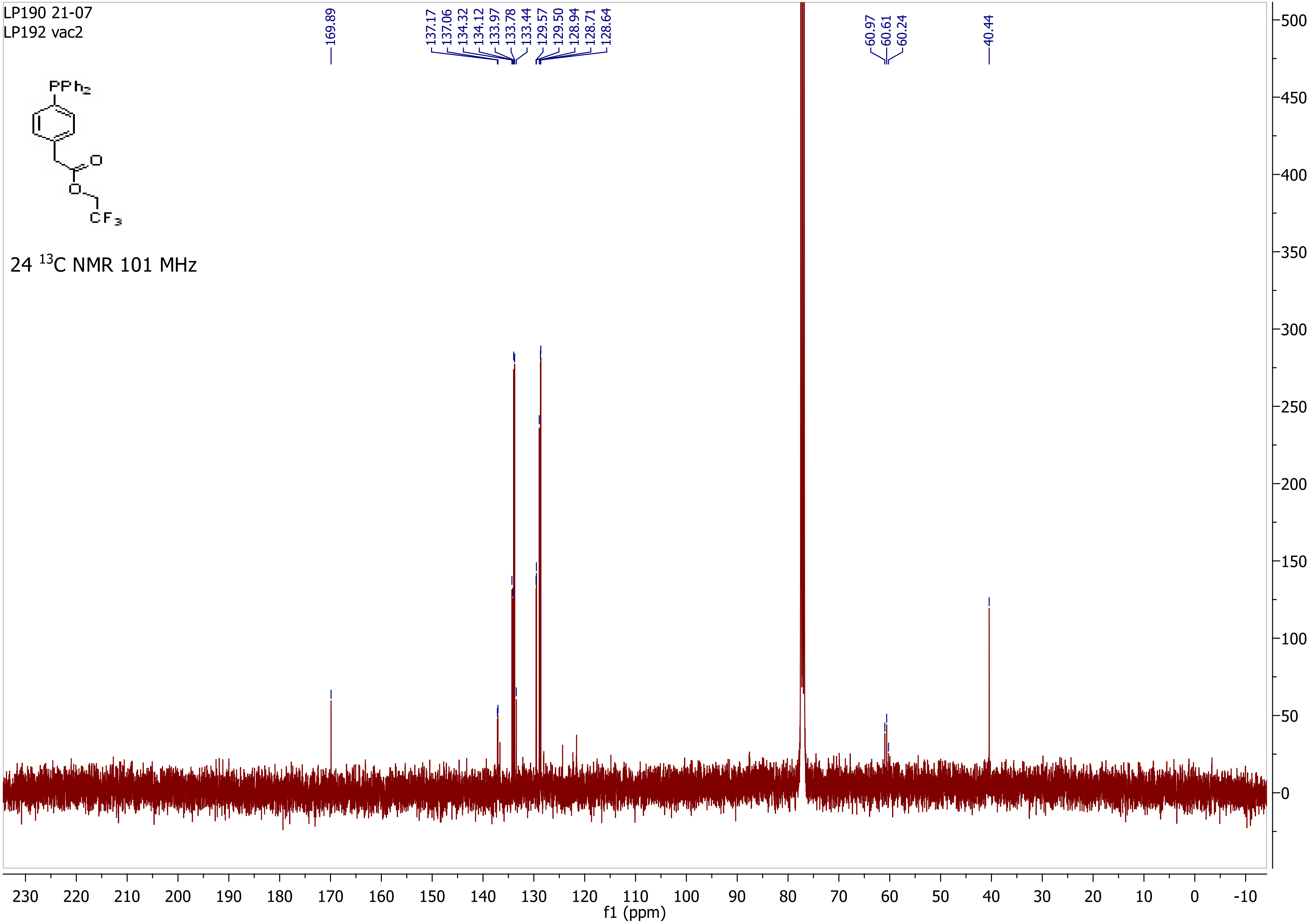

LP190 21-07
LP192 vac2
PPh2
O
O
CF3
24 13C NMR 101 MHz
169.89
137.17
137.06
134.32
134.12
133.97
133.78
133.44
129.57
129.50
128.94
128.71
128.64
60.97
60.61
60.24
40.44
f1 (ppm)

LP190 21-07
LP192 vac2

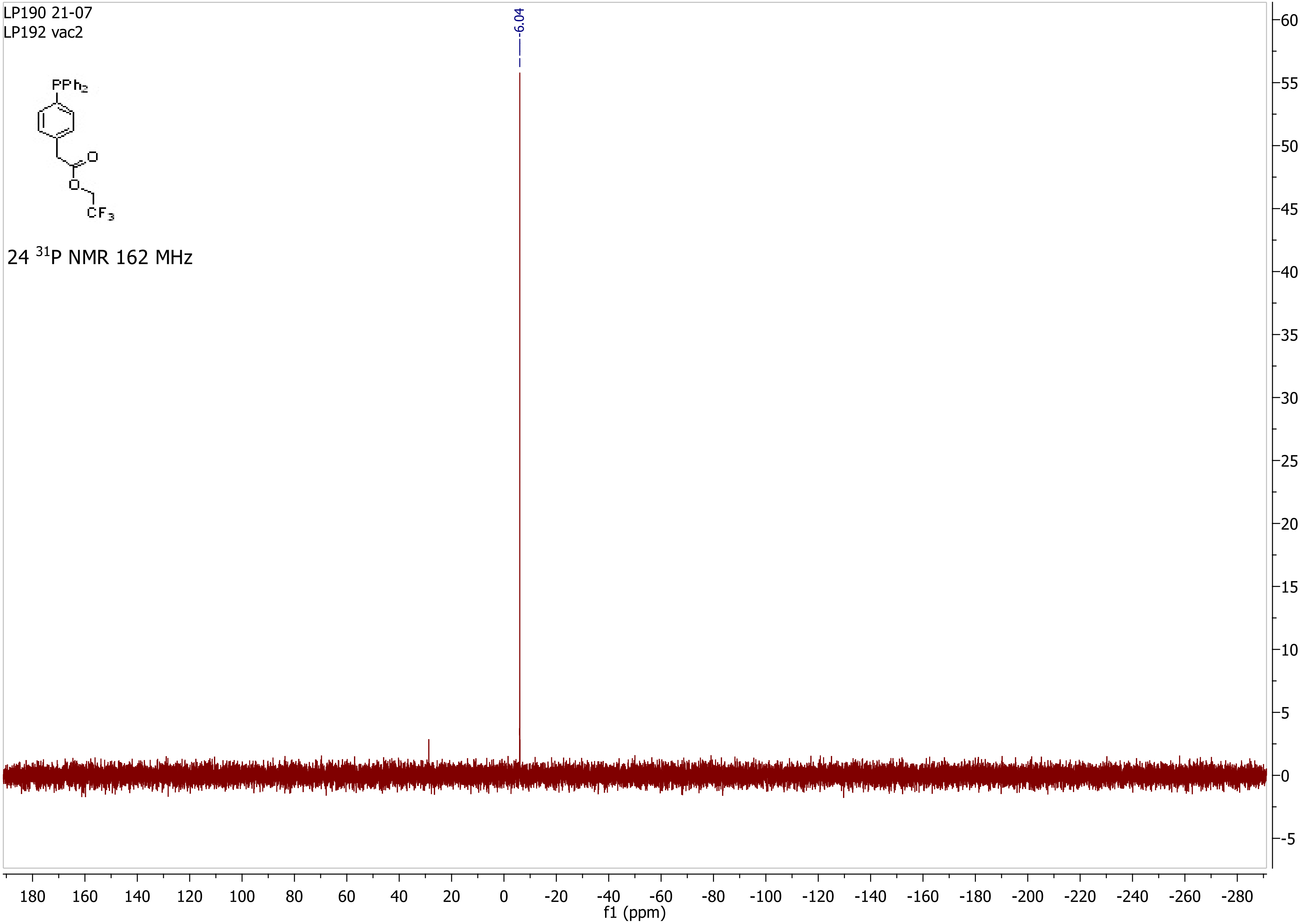

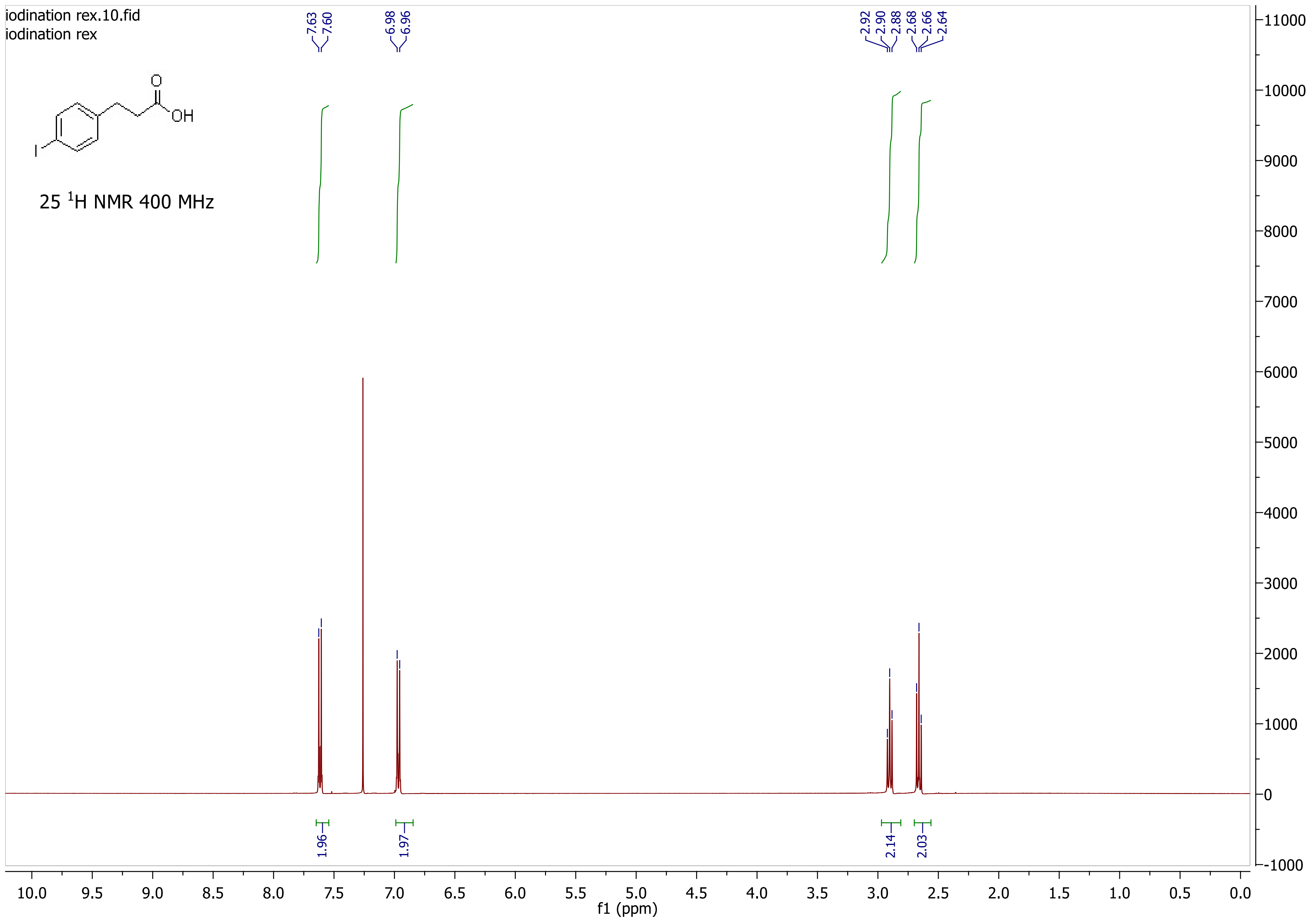

iodination rex.10.fid
iodination rex
OH
O
I
25 1H NMR 400 MHz
7.63
7.60
6.98
6.96
2.92
2.90
2.88
2.68
2.66
2.64
1.96
1.97
2.14
2.03
f1 (ppm)

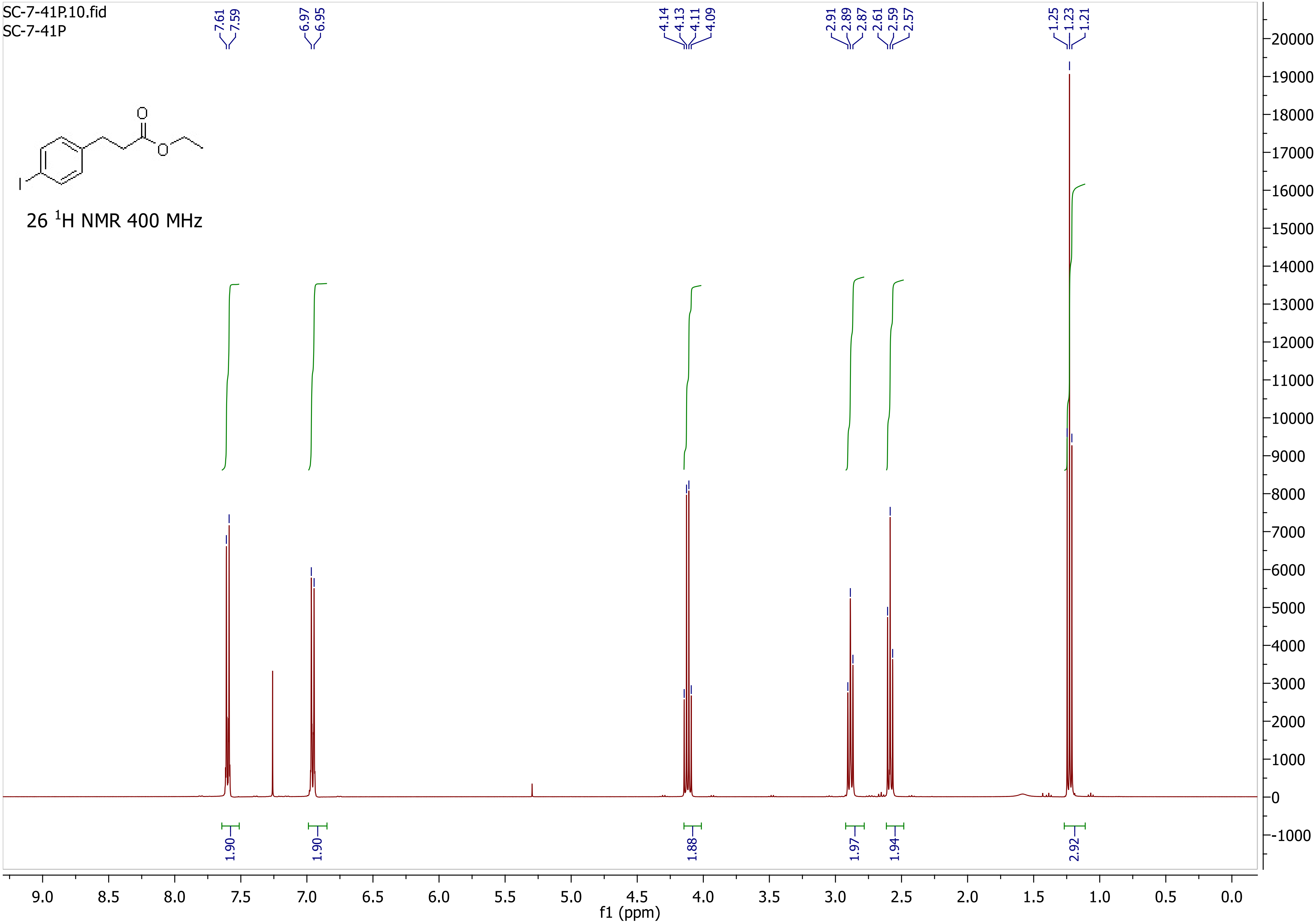
SC-7-41P.10.fid
SC-7-41P
26 ¹H NMR 400 MHz
O
O
I
7.61
7.59
6.97
6.95
4.14
4.13
4.11
4.09
2.91
2.89
2.87
2.61
2.59
2.57
1.25
1.23
1.21
1.90
1.90
1.88
1.97
1.94
2.92
9.0 8.5 8.0 7.5 7.0 6.5 6.0 5.5 5.0 4.5 4.0 3.5 3.0 2.5 2.0 1.5 1.0 0.5 0.0
f1 (ppm)
20000 19000 18000 17000 16000 15000 14000 13000 12000 11000 10000 9000 8000 7000 6000 5000 4000 3000 2000 1000 0 -1000

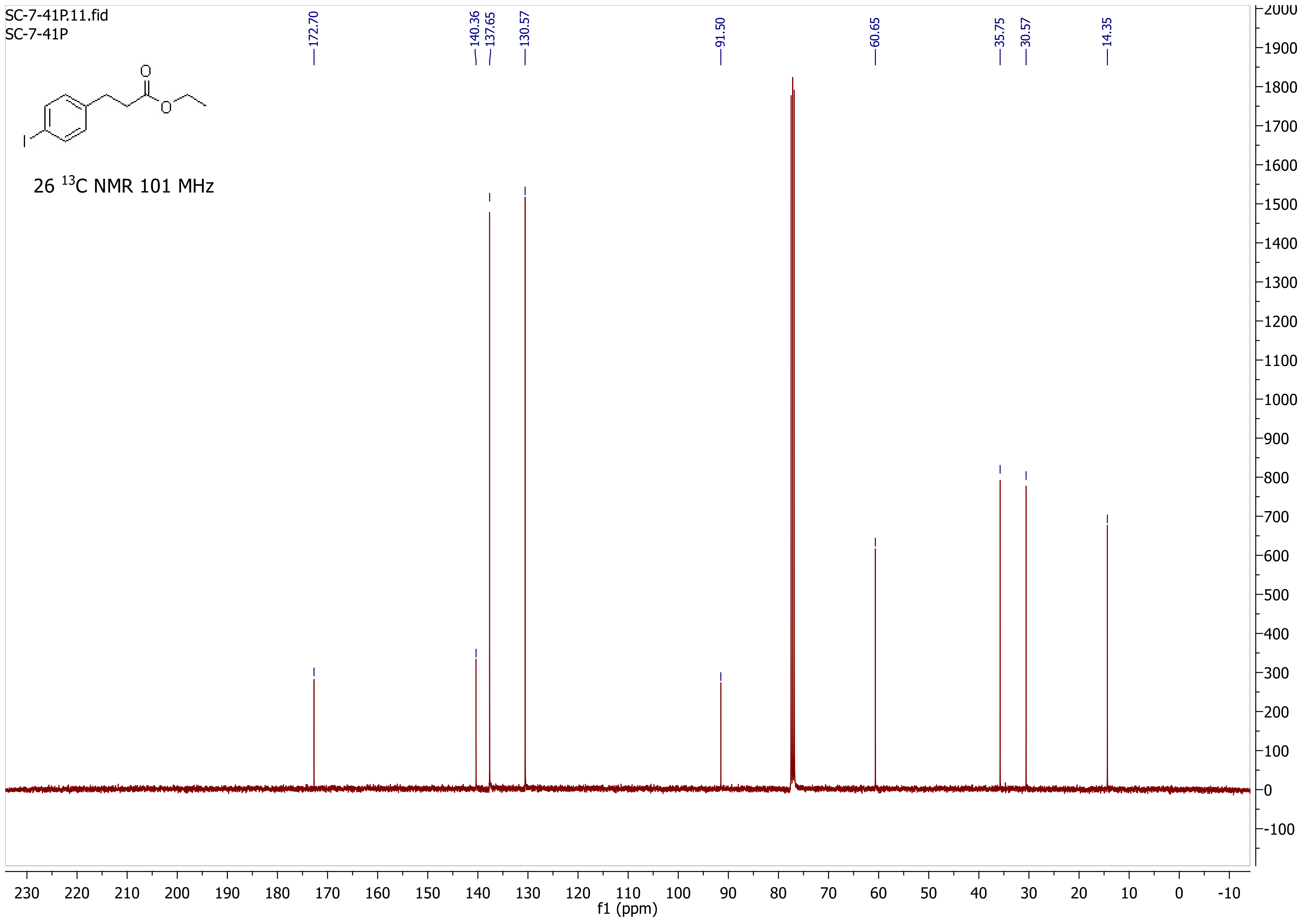
SC-7-41P.11.fid
SC-7-41P
I
O
O
26 13C NMR 101 MHz
172.70
140.36
137.65
130.57
91.50
60.65
35.75
30.57
14.35
f1 (ppm)

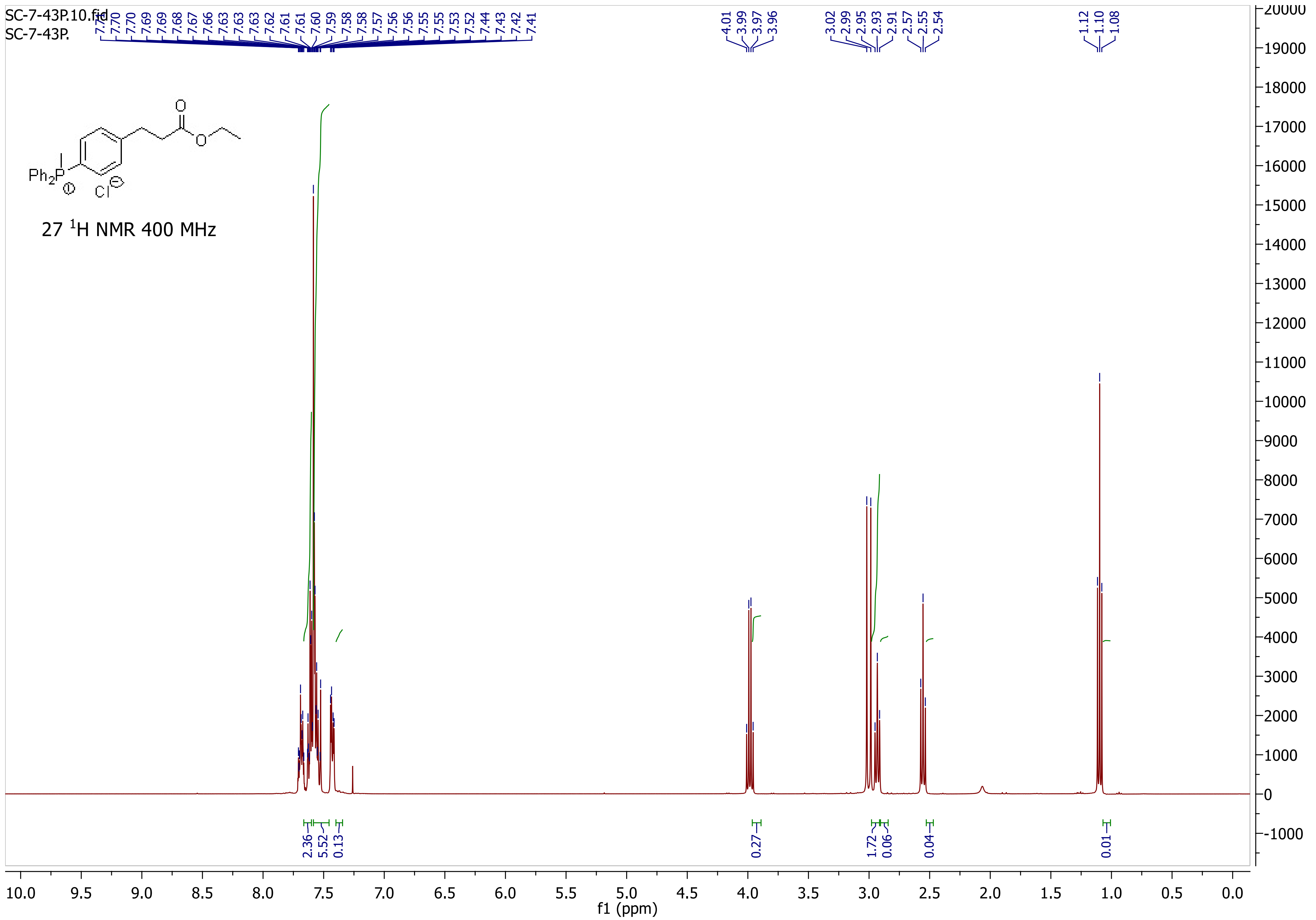

SC-7-43P.10.fid
SC-7-43P.
Ph2P
Cl
27 1H NMR 400 MHz
7.70
7.70
7.69
7.69
7.68
7.67
7.66
7.63
7.63
7.63
7.62
7.61
7.61
7.60
7.59
7.58
7.58
7.57
7.56
7.56
7.55
7.55
7.53
7.52
7.44
7.43
7.42
7.41
4.01
3.99
3.97
3.96
3.02
2.99
2.95
2.93
2.91
2.57
2.55
2.54
1.12
1.10
1.08
2.36
5.52
0.13
0.27
1.72
0.06
0.04
0.01
f1 (ppm)

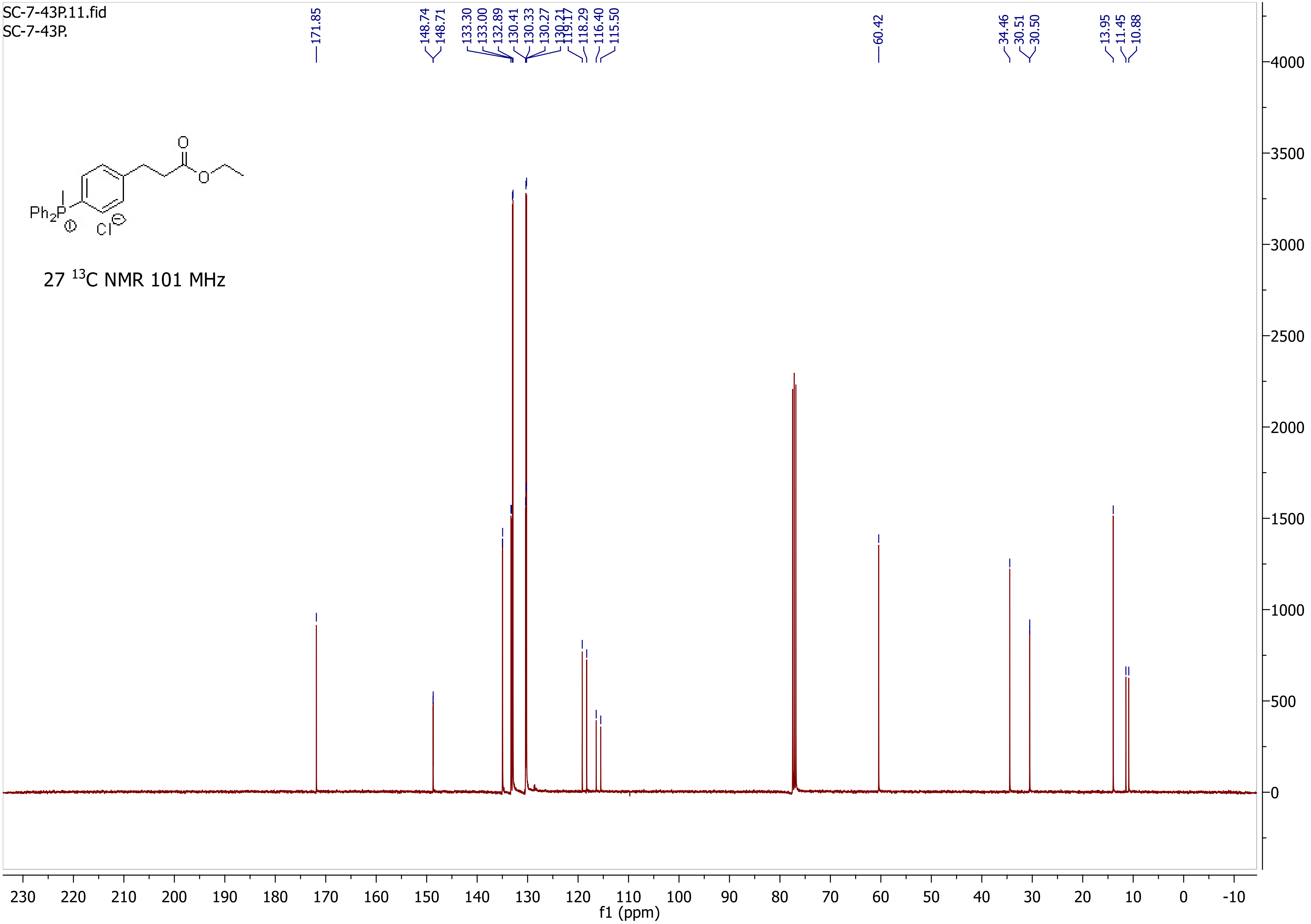

SC-7-43P.11.fid
SC-7-43P.
171.85
148.74
148.71
133.30
133.00
132.89
130.41
130.33
130.27
130.21
119.17
118.29
116.40
115.50
60.42
34.46
30.51
30.50
13.95
11.45
10.88
O
O
Ph2P
Cl
27 13C NMR 101 MHz
f1 (ppm)

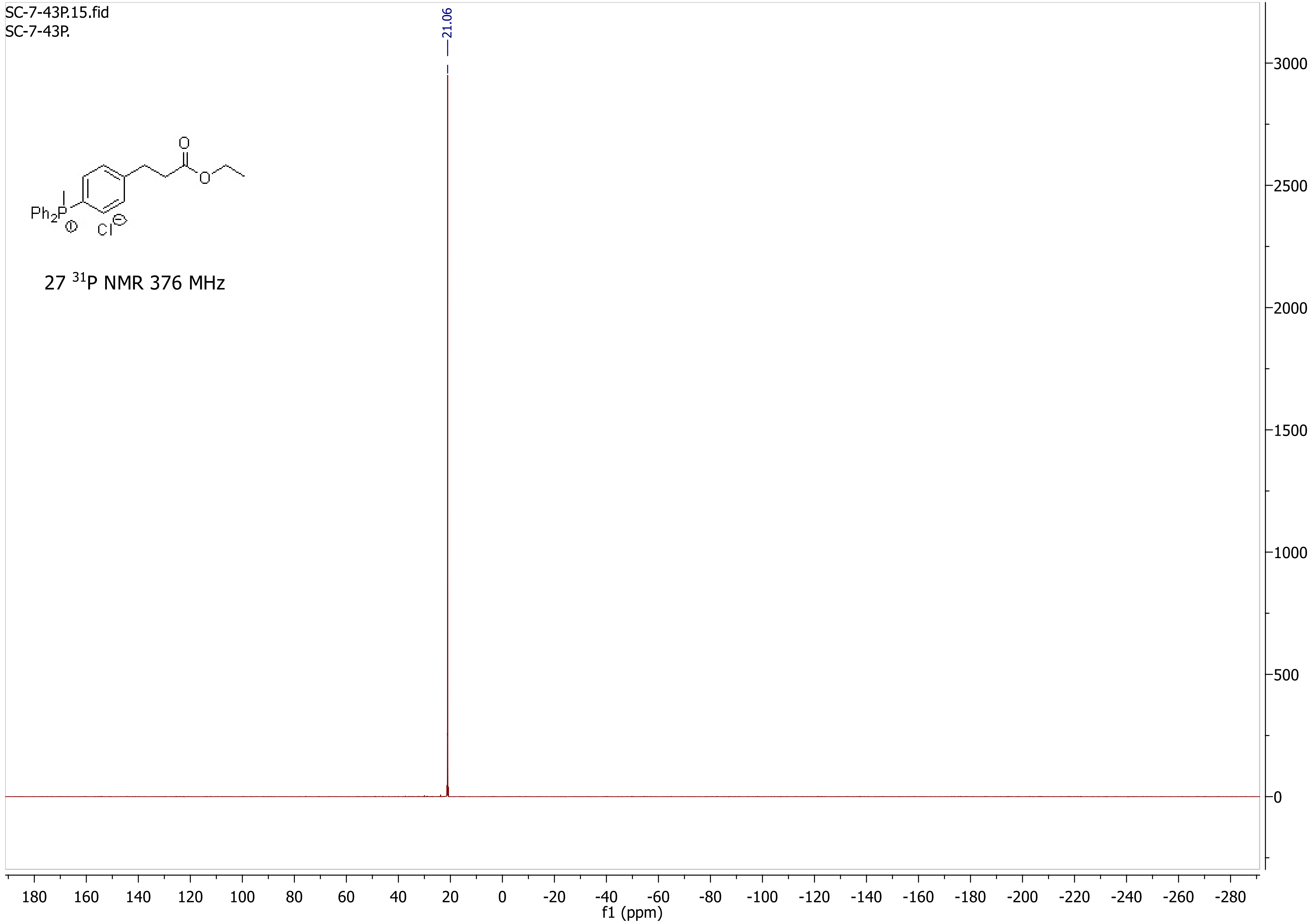
SC-7-43P.15.fid
SC-7-43P.
21.06
O
O
Ph2P
Cl
27 31P NMR 376 MHz
3000
2500
2000
1500
1000
500
0
180 160 140 120 100 80 60 40 20 0 -20 -40 -60 -80 -100 -120 -140 -160 -180 -200 -220 -240 -260 -280
f1 (ppm)

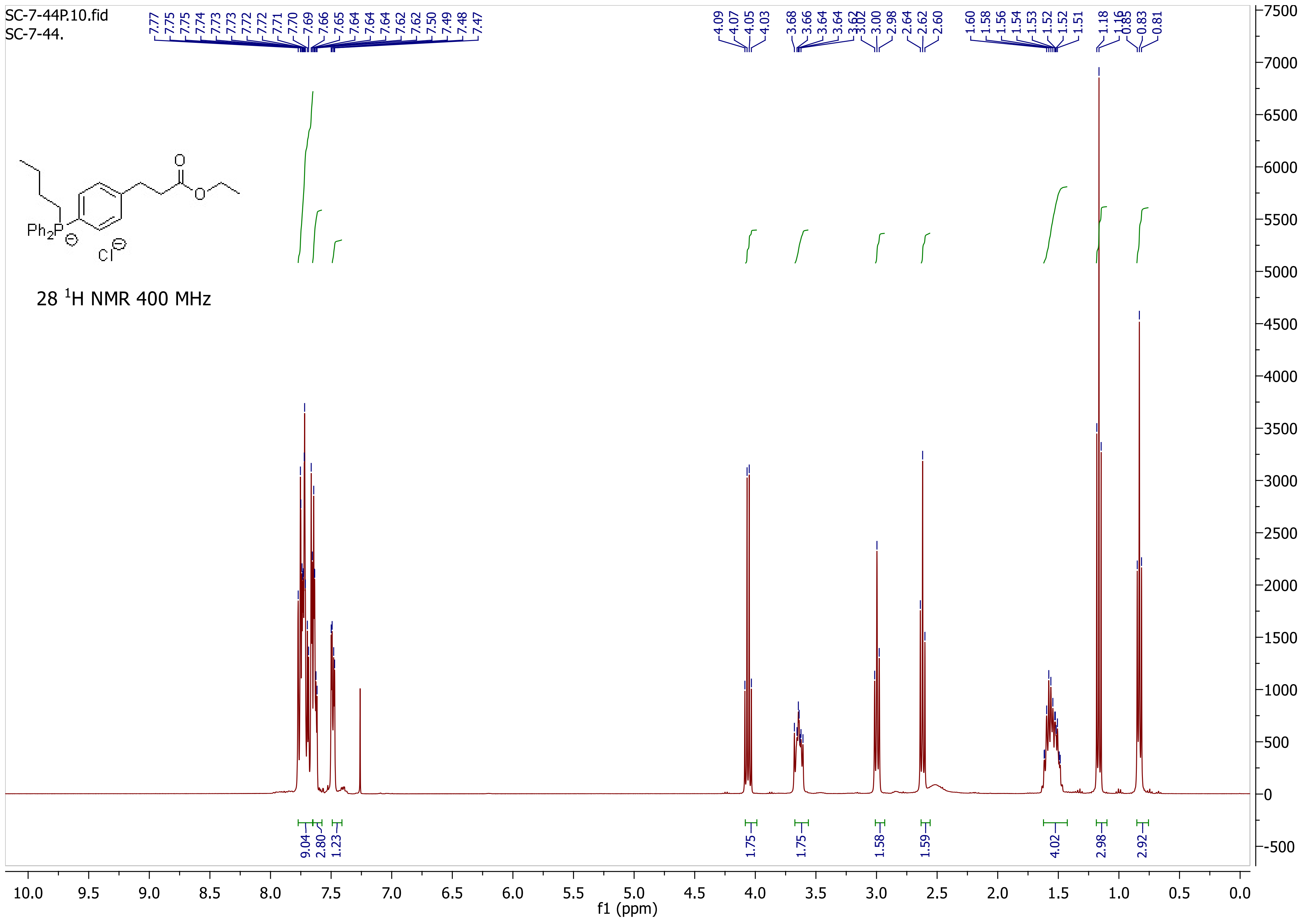

SC-7-44P.10.fid
SC-7-44.
7.77 7.75 7.75 7.74 7.73 7.73 7.72 7.72 7.71 7.70 7.69 7.66 7.65 7.64 7.64 7.64 7.62 7.62 7.50 7.49 7.48 7.47
4.09 4.07 4.05 4.03
3.68 3.66 3.64 3.64 3.62 3.02 3.00 2.98
2.64 2.62 2.60
1.60 1.58 1.56 1.54 1.53 1.52 1.52 1.51
1.18 1.16 0.85 0.83 0.81
O
O
Ph2P
Cl
28 1H NMR 400 MHz
9.04 2.80 1.23 1.75 1.75 1.58 1.59 4.02 2.98 2.92
10.0 9.5 9.0 8.5 8.0 7.5 7.0 6.5 6.0 5.5 5.0 4.5 4.0 3.5 3.0 2.5 2.0 1.5 1.0 0.5 0.0
f1 (ppm)
7500 7000 6500 6000 5500 5000 4500 4000 3500 3000 2500 2000 1500 1000 500 0 -500

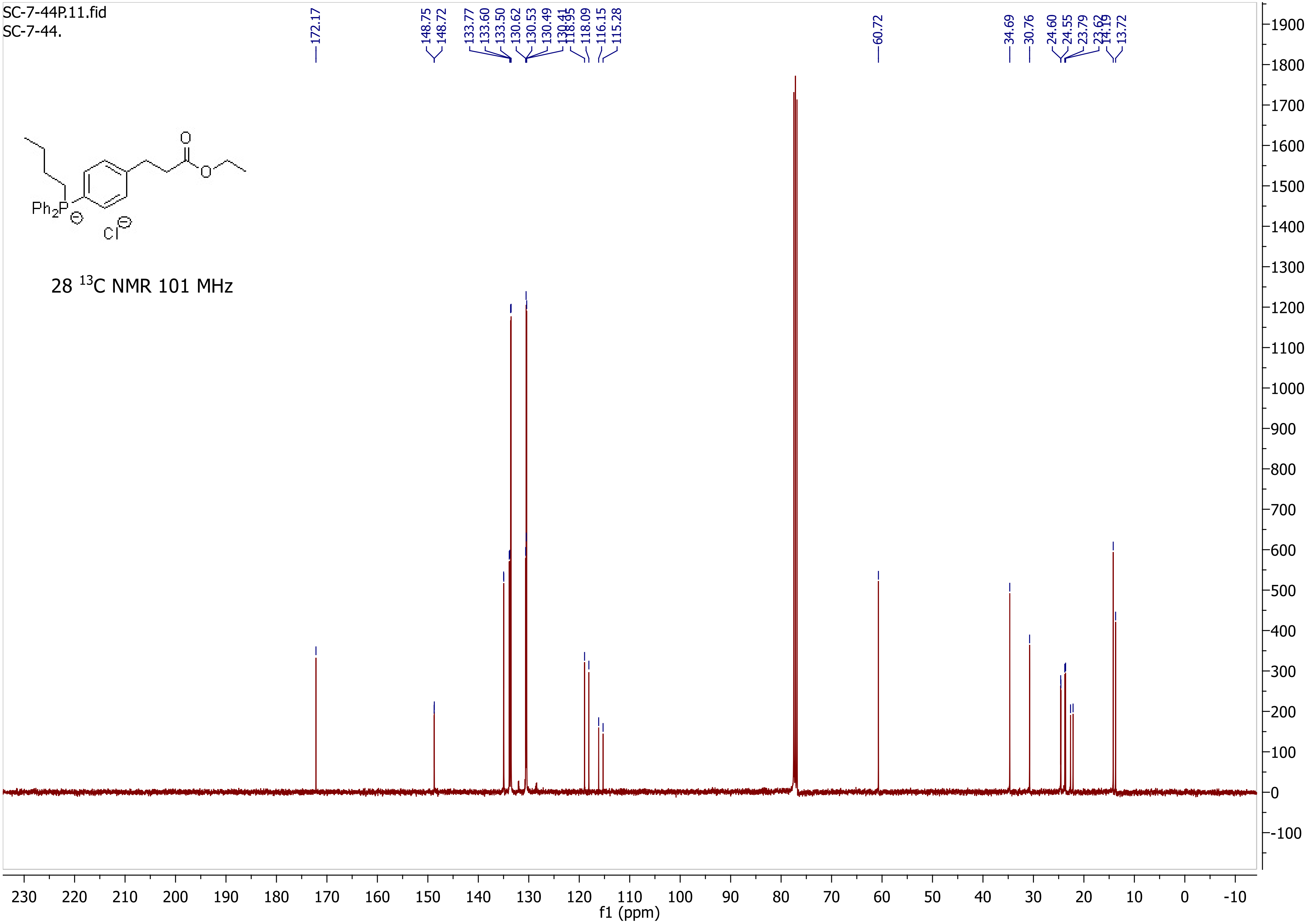

SC-7-44P.11.fid
SC-7-44.
172.17
148.75
148.72
133.77
133.60
133.50
130.62
130.53
130.49
130.41
118.95
118.09
116.15
115.28
60.72
34.69
30.76
24.60
24.55
23.79
23.62
14.19
13.72
O
O
Ph2P
Cl
28 13C NMR 101 MHz
f1 (ppm)

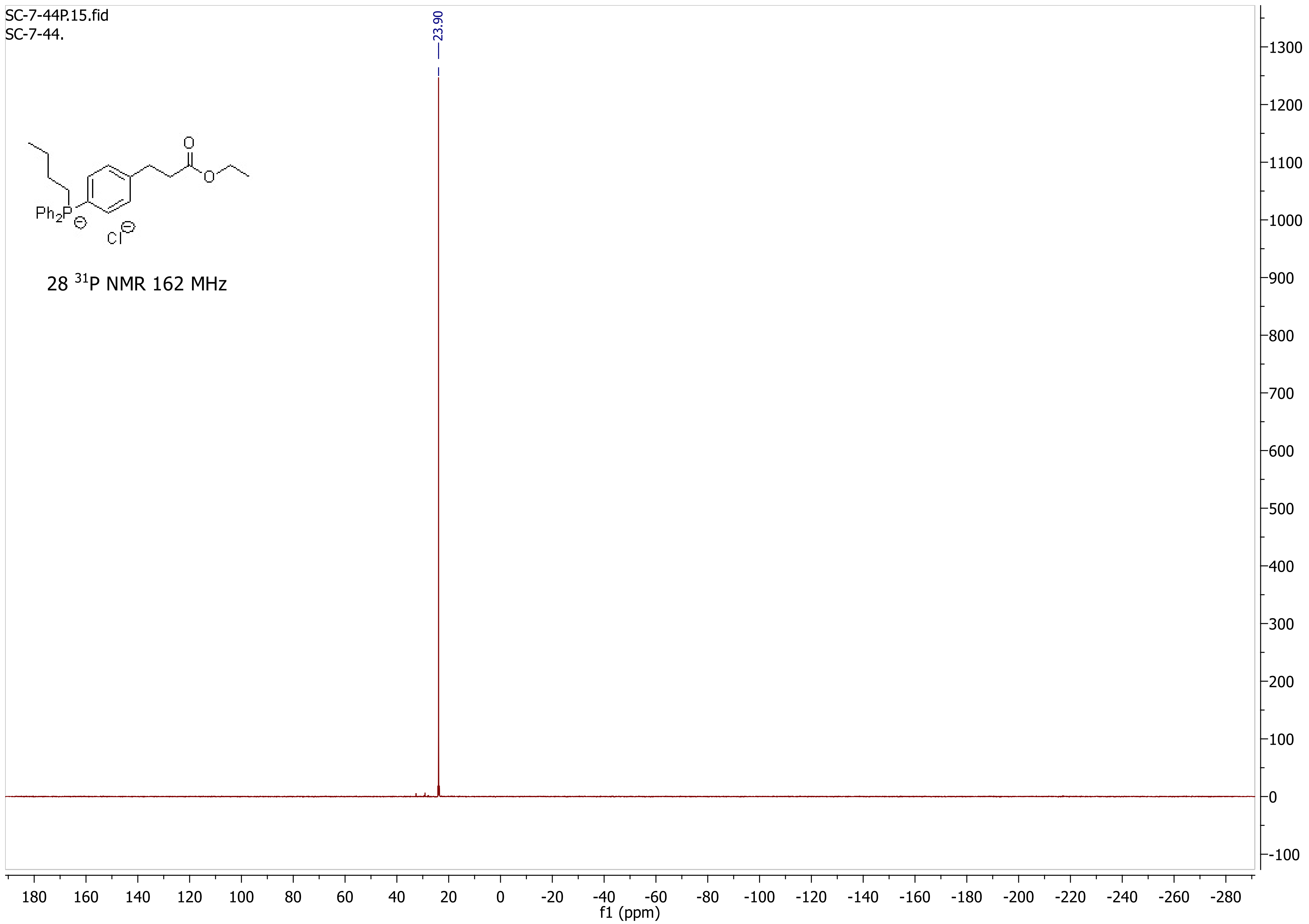
SC-7-44P.15.fid
SC-7-44.
23.90
Ph2P
Cl
O
28 31P NMR 162 MHz
1300
1200
1100
1000
900
800
700
600
500
400
300
200
100
0
-100
180 160 140 120 100 80 60 40 20 0 -20 -40 -60 -80 -100 -120 -140 -160 -180 -200 -220 -240 -260 -280
f1 (ppm)

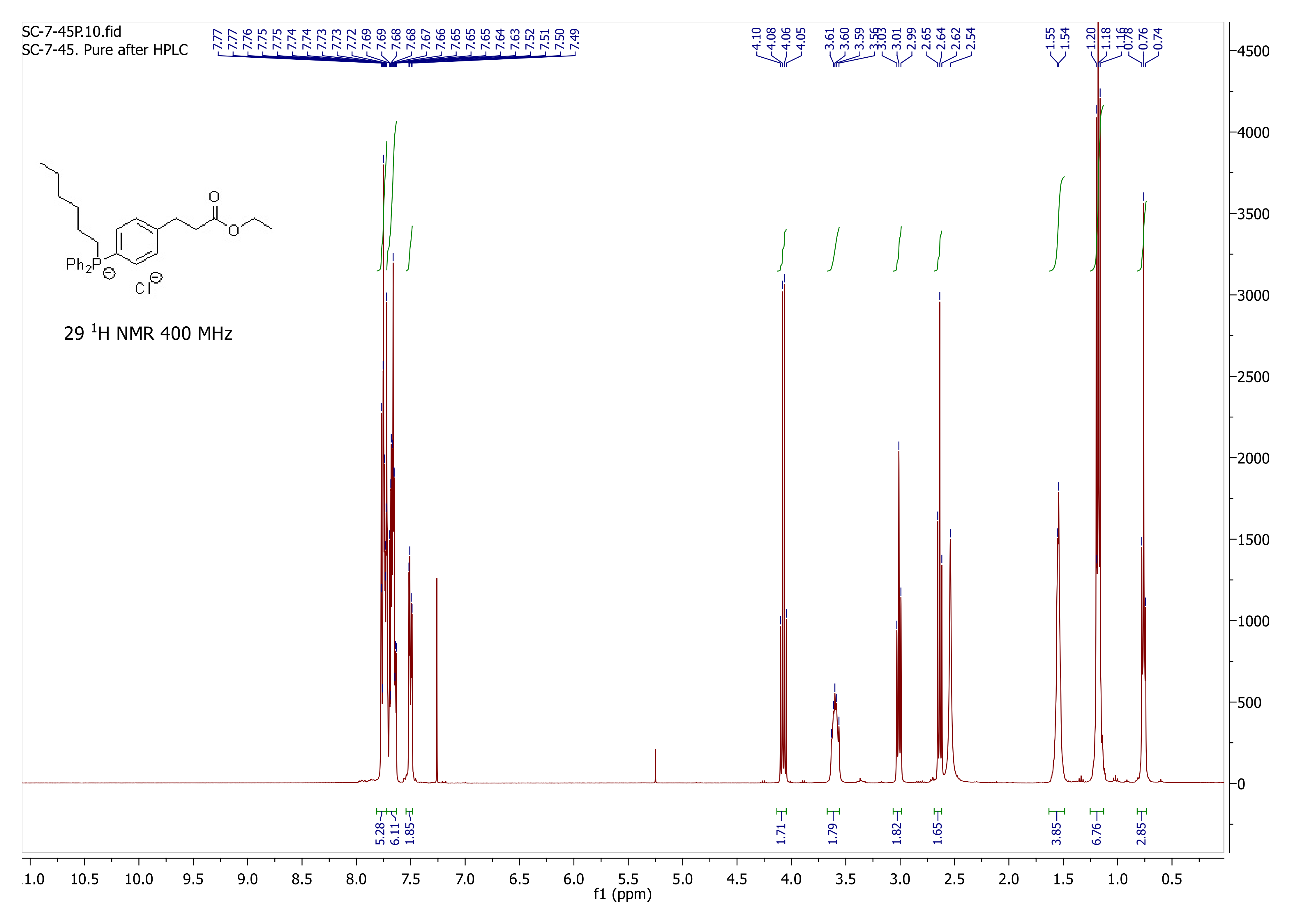
SC-7-45P.10.fid
SC-7-45. Pure after HPLC
7.77 7.77 7.76 7.75 7.75 7.74 7.74 7.73 7.73 7.72 7.69 7.69 7.68 7.68 7.67 7.66 7.65 7.65 7.65 7.64 7.63 7.52 7.51 7.50 7.49
4.10 4.08 4.06 4.05
3.61 3.60 3.59 3.56 3.03 3.01 2.99 2.65 2.64 2.62 2.54
1.55 1.54
1.20 1.18 1.16 0.78 0.76 0.74
Ph2P
O
O
Cl
29 1H NMR 400 MHz
5.28 6.11 1.85 1.71 1.79 1.82 1.65 3.85 6.76 2.85
4500 4000 3500 3000 2500 2000 1500 1000 500 0
11.0 10.5 10.0 9.5 9.0 8.5 8.0 7.5 7.0 6.5 6.0 5.5 5.0 4.5 4.0 3.5 3.0 2.5 2.0 1.5 1.0 0.5
f1 (ppm)

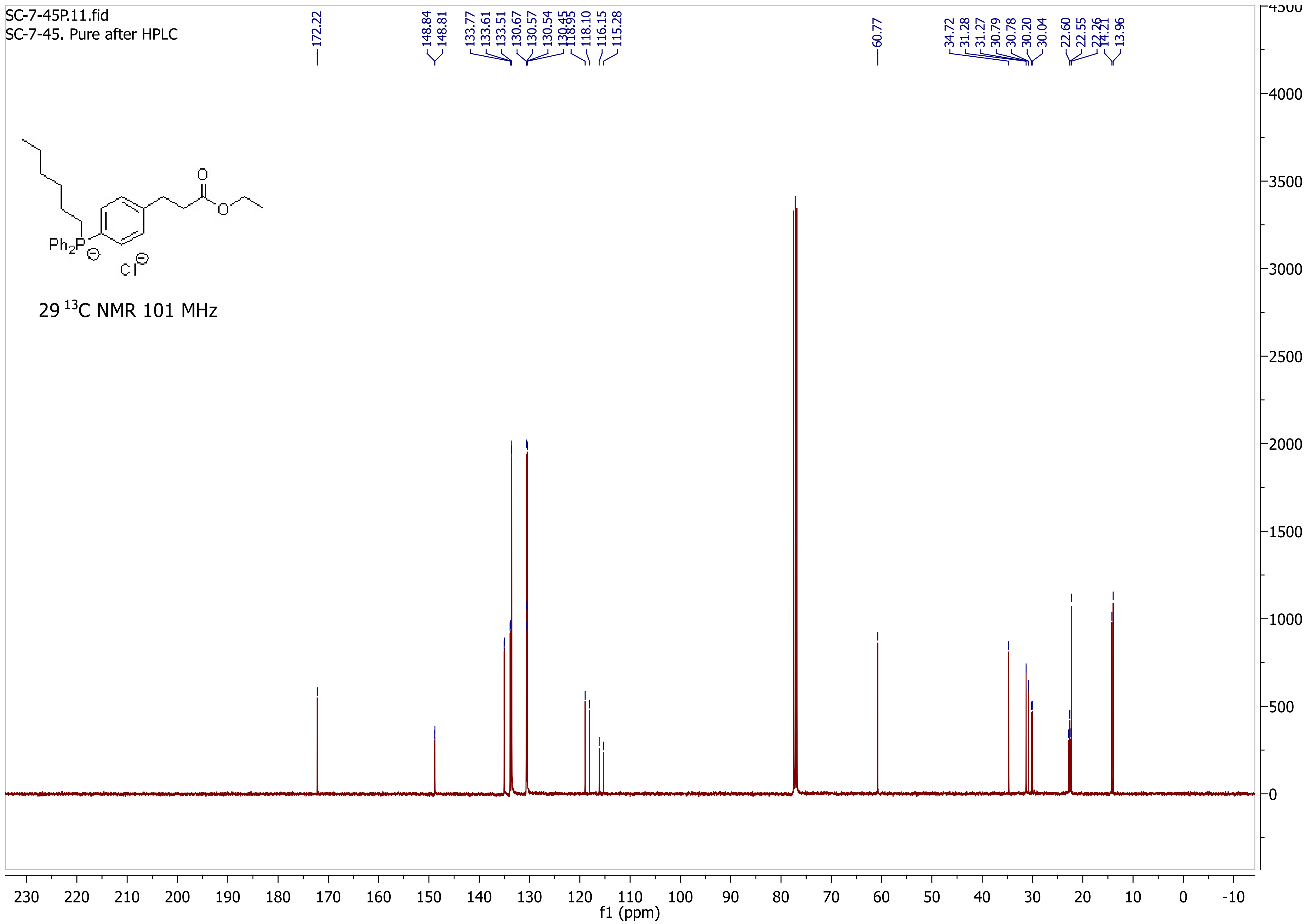
SC-7-45P.11.fid
SC-7-45. Pure after HPLC
172.22
148.84
148.81
133.77
133.61
133.51
130.67
130.57
130.54
130.45
118.95
118.10
116.15
115.28
60.77
34.72
31.28
31.27
30.79
30.78
30.20
30.04
22.60
22.55
22.26
14.21
13.96
O
O
Ph2P
Cl
29 13C NMR 101 MHz
f1 (ppm)

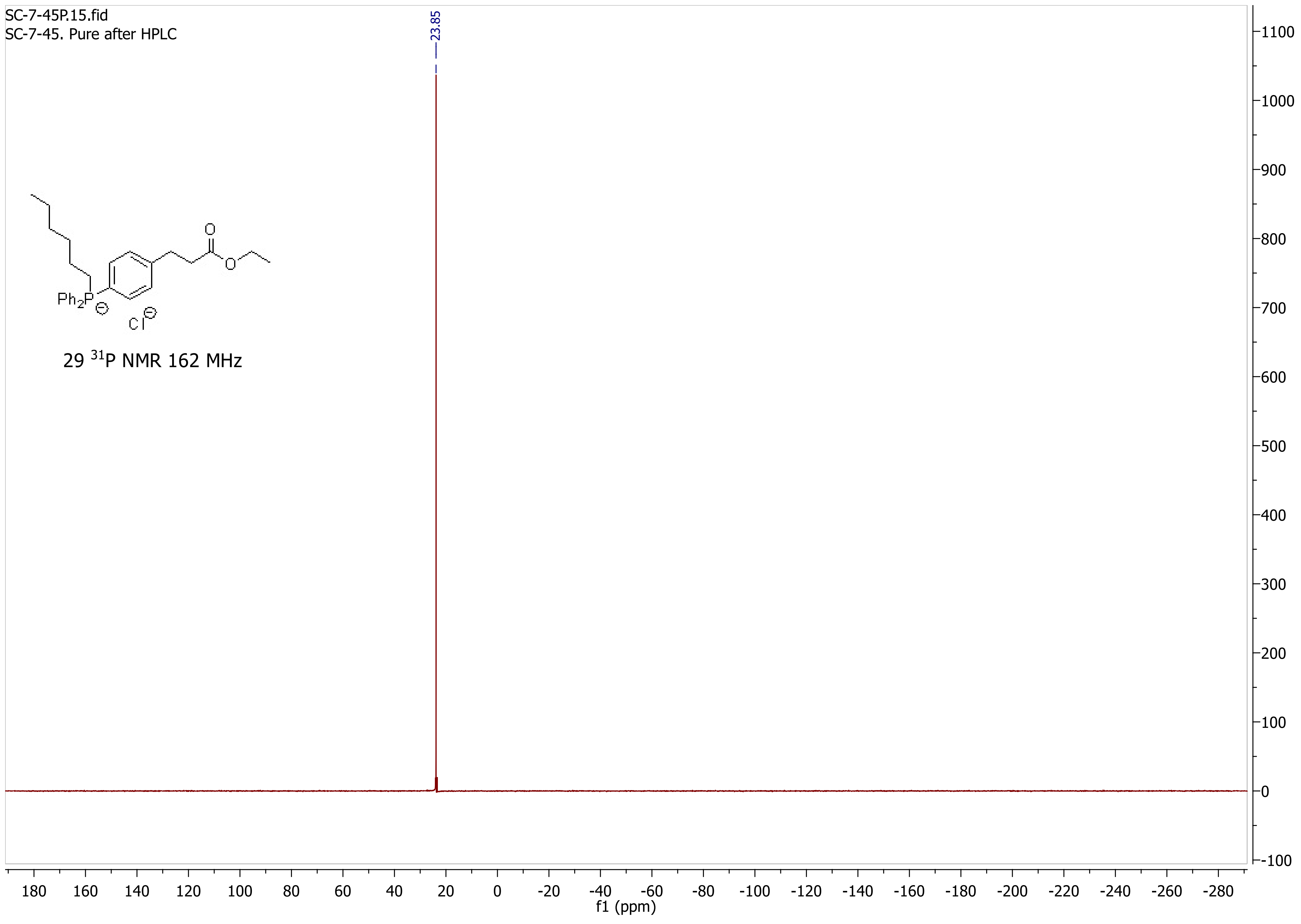
SC-7-45P.15.fid
SC-7-45. Pure after HPLC
23.85
O
O
Ph$_2$P
Cl
29 ^{31}P NMR 162 MHz
1100
1000
900
800
700
600
500
400
300
200
100
0
-100
180 160 140 120 100 80 60 40 20 0 -20 -40 -60 -80 -100 -120 -140 -160 -180 -200 -220 -240 -260 -280
f1 (ppm)
